# New 1,2,4-oxadiazole derivatives with positive mGlu_4_ receptor modulation activity and antipsychotic-like properties

**DOI:** 10.1080/14756366.2021.1998022

**Published:** 2021-12-11

**Authors:** Anna Stankiewicz, Katarzyna Kaczorowska, Ryszard Bugno, Aneta Kozioł, Maria H. Paluchowska, Grzegorz Burnat, Barbara Chruścicka, Paulina Chorobik, Piotr Brański, Joanna M. Wierońska, Beata Duszyńska, Andrzej Pilc, Andrzej J. Bojarski

**Affiliations:** a Department of Medicinal Chemistry, Maj Institute of Pharmacology, Polish Academy of Sciences, Kraków, Poland; b Department of Neurobiology, Maj Institute of Pharmacology, Polish Academy of Sciences, Kraków, Poland

**Keywords:** Metabotropic glutamate receptor 4 (mGlu_4_ receptor), positive allosteric modulator (PAM), 1,2,4-oxadiazoles, antipsychotic properties, anxiolytics

## Abstract

Considering the allosteric regulation of mGlu receptors for potential therapeutic applications, we developed a group of 1,2,4-oxadiazole derivatives that displayed mGlu_4_ receptor positive allosteric modulatory activity (EC_50_ = 282–656 nM). Selectivity screening revealed that they were devoid of activity at mGlu_1_, mGlu_2_ and mGlu_5_ receptors, but modulated mGlu_7_ and mGlu_8_ receptors, thus were classified as group III-preferring mGlu receptor agents. None of the compounds was active towards hERG channels or in the mini-AMES test. The most potent in vitro mGlu_4_ PAM derivative **52** (N-(3-chloro-4-(5-(2-chlorophenyl)-1,2,4-oxadiazol-3-yl)phenyl)picolinamide) was readily absorbed after i.p. administration (male Albino Swiss mice) and reached a maximum brain concentration of 949.76 ng/mL. Five modulators (**34**, **37**, **52**, **60** and **62**) demonstrated significant anxiolytic- and antipsychotic-like properties in the SIH and DOI-induced head twitch test, respectively. Promising data were obtained, especially for N-(4-(5-(2-chlorophenyl)-1,2,4-oxadiazol-3-yl)-3-methylphenyl)picolinamide (**62**), whose effects in the DOI-induced head twitch test were comparable to those of clozapine and better than those reported for the selective mGlu_4_ PAM ADX88178.

## Graphical Abstract

**Figure:**
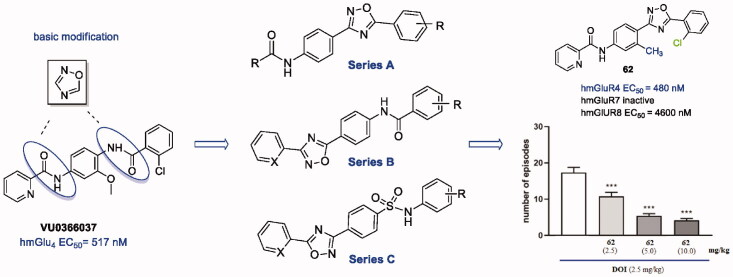

## Introduction

Targeting the allosteric regulation of G protein-coupled receptors (GPCRs) has introduced a new paradigm for drug discovery. Among the metabotropic glutamate receptors (mGlu receptors), the first developed mGlu receptor ligands influenced the receptor response by competing with endogenous glutamate at the orthosteric binding site^1^^,^^2^. However, the high level of conservation at the glutamate binding pocket hampered the discovery of truly selective ligands for receptors belonging to groups I–III of the mGlu receptors family. As improvement of receptor selectivity was pointed out among the main advantages of the allosteric mode of action, screening campaigns into the identification of new selective mGlu receptors ligands were oriented towards molecules that bind to sites other than the orthosteric region of the receptor^3–6^. These compounds, identified as allosteric modulators, affected mGlu receptor activity either positively by potentiating the response of the receptor (i.e. positive allosteric modulators – PAMs) or negatively by antagonising the activity of orthosteric agonists (negative allosteric modulators – NAMs)^6^^,^^7^.

From a historical point of view, interest in mGlu receptor ligand discovery has concentrated mostly on group I (mGlu_1_ and mGlu_5_) and II (mGlu_2_ and mGlu_3_) receptors, leading to several potential drug candidates in clinical trials for various central nervous system (CNS) diseases^6^^,^^8–14^. However, in recent years, the therapeutic potential of group III (mGlu_4_, mGlu_6–8_) receptors, especially type 4 receptors, has become a focus of research at a number of pharmaceutical companies, such as Addex^15–18^, Merck^17^^,^^19^^,^^20^, Lundbeck^21–24^, Prexton Therapeutics^7^^,^^25^, Domain Therapeutics^26^, Hoffmann-La Roche^27^, Boehringer Ingelheim/Evotec^28^^,^^29^ as well as Vanderbilt University^23^^,^^25^^,^^30–36^, which holds leading position in the development of mGlu receptor allosteric modulators.

**Figure 1.**
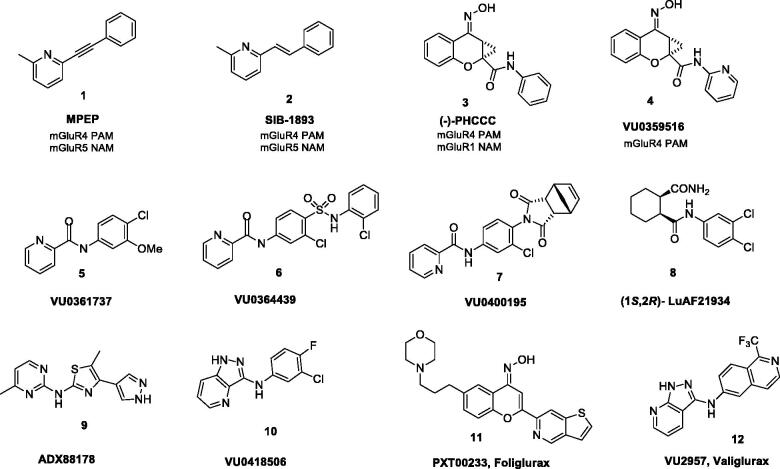
Chemical structures of various classes of mGlu_4_ PAMs. Compounds **1** and **2** were the first ligands identified as PAMs for mGlu_4_ receptor, **11** is currently in phase II clinical trials, and **12** has advanced as a preclinical development candidate.

The first ligands identified as PAMs for mGlu_4_ receptor, MPEP (**1**) and SIB-1893 (**2**)^34^^,^^37^ (Figure 1), were weak and not selective, as they showed cross-reactivity with mGlu_5_ receptor and mGlu_1_ receptor. Similarly, the ligand (–)-PHCCC (**3**, mGlu_4_ EC_50_ = 1.4 µM), originally thought to be a breakthrough, proved to be non-selective^20^, showing partial antagonist activity towards mGlu_1_ receptor as well as agonist activity towards mGlu_6_^34^. However, further pharmacological studies with the use of (–)–PHCCC (**3**) revealed its efficacy in animal models of Parkinson’s disease^7^^,^^20^^,^^33^^,^^35^^,^^38–44^, depression^45^^,^^46^, anxiety^47^^,^^48^, epilepsy^49^^,^^50^, neuroprotection^51^ and oncology^52^ but also showed a poor pharmacokinetic profile, limited brain exposure and low aqueous solubility^2^^,^^53^^,^^54^.

Modification of the (–)-PHCCC (**3**) structure by replacing the phenyl amide with a 2-pyridyl amide led to VU0359516 (**4**, EC_50_ = 0.38 µM) (Figure 1), a more potent, efficacious and highly selective mGlu_4_ receptor PAM devoid of mGlu_1_ receptor activity^55^. This discovery was a starting point for extensive structure-activity relationship (SAR) studies initiated at Vanderbilt University and continued by others for the identification of a number of new PAMs of mGlu_4_ receptor of various chemotypes^53^^,^^56^^,^^57^.

The reported PAMs of mGlu_4_ receptor represent various chemical classes of compounds and different scaffolds^33^, such as picolinamides (e.g. VU0361737 (**5**))^9^^,^^15^^,^^31^^,^^58^, sulphonamides (e.g. VU0364439 (**6**))^19^^,^^59^, phthalimides (e.g. VU0400195 (**7**))^60^, cyclohexyl amides (e.g. (1S,2R)-Lu AF21934 (**8**))^21^, triaryl amines (ADX88178 (**9**))^61–63^, pyrazolo[4,3-*b*] pyridines (e.g. VU0418506 (**10**))^31^, benzisoxazoles^64^^,^^65^, and other polyheterocycles^16^^,^^22^^,^^66^^,^^67^.

Although most of the developed mGlu_4_ PAMs suffered from poor physicochemical and pharmacokinetic properties, limited CNS exposure and/or CYP inhibition/induction issues, their pharmacological investigation did provide *in vivo* target validation and further increased interest in the mechanism of mGlu_4_ modulation.

The first mGlu_4_ PAM clinical candidate, PXT002331 (**11**), a chemical analogue of (–)-PHCCC (**3**) developed by Prexton/Domain Therapeutics^7^ had very good mGlu_4_ PAM potency (EC_50_ = 46 nM), improved pharmacokinetics, high CNS penetration with preferential exposure in the brain, and significant anti-Parkinson’s activity in vivo in rodent models of motor symptoms of the disease, however, Lundbeck has announced in April 2020 that the phase IIa study (AMBLED) of its novel selective positive allosteric modulator of the glutamate 4 receptor, Foliglurax, for the treatment of Parkinson’s disease did not meet the primary study endpoint and the study was stopped. The second potential clinical candidate has been recently reported by Vanderbilt researches^35^. Valiglurax (**12**) represents an isoquinoline-based series and has shown mGlu_4_ receptor PAM potency (EC_50_ = 64.6 nM) comparable to PXT002331. Moreover, **12** exhibits excellent pharmacokinetic properties, an acceptable CYP profile and affords robust oral efficacy in a pharmacodynamic model.

Considering the importance of mGlu_4_ receptor modulation for potential therapeutic applications, we focussed on searching for mGlu4 receptor PAM activity among the group of 1,2,4-oxadiazole derivatives. In addition to identifying new PAMs with high activity for mGlu_4_ receptor, we also evaluated their mGlu receptor selectivity profile and therapeutic potential with respect to anxiolytic, antipsychotic and antidepressant properties in preliminary *in vivo* tests. The presented results also include a determination of the cardiotoxic risk and potential mutagenic properties as well as a preliminary metabolic and pharmacokinetic profile for the most active mGlu_4_ receptor PAMs (**34**, **37**, **49**, **52**, **60** and **62**).

## Results and discussion

### Chemistry and *in vitro* activity

**Figure 2.**
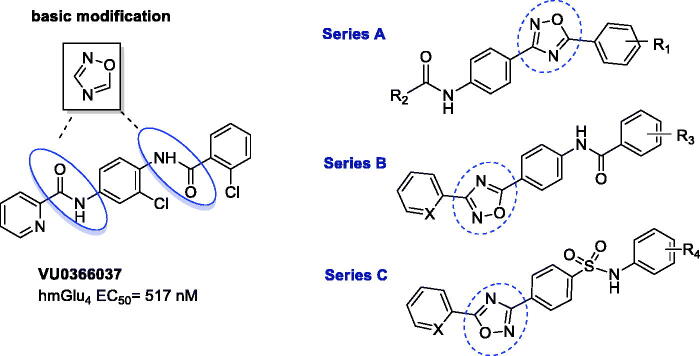
Design of three new series of compounds generated *via* bioisosteric replacement.

The bioisosteric approach was used to design a pilot series of compounds, and VU0366037 (EC_50_ = 517 nM, Figure 2), the PAM of mGlu_4_ receptor discovered at Vanderbilt University, was selected as the parent derivative for structural modifications. The basic bioisosteric replacement focussed on exchanging one of the amide groups for the 1,2,4-oxadiazole system^68^ and coupling it with various substituted aryl or heteroaryl moieties, in such a way that a distinct series of compounds (A–C) were developed (Figure 2).

**Scheme 1.**
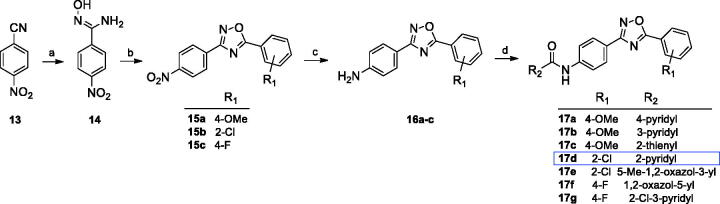
Reagents and conditions: (a) NH_2_OH·HCl, NaOH_aq_, EtOH, reflux, 1–5 h; (b) R_1_COCl, toluene, K_2_CO_3_, MW 170 °C, 10 min; (c) Fe, CH_3_COOH, EtOH, water, 60 °C, 1–2 h or SnCl_2_, 5 N HCl, EtOH, reflux, 2 h; (d) R_2_COCl, py, rt, overnight.

**Scheme 2.**
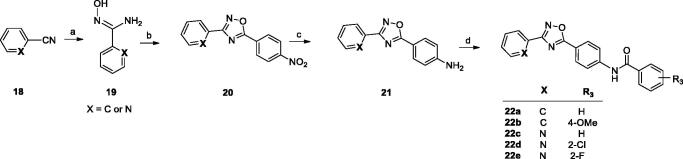
Reagents and conditions: (a) NH_2_OH·HCl, NaOH_aq_, EtOH, reflux, 1–5 h; (b) 4-nitrobenzoyl chloride, toluene, K_2_CO_3_, MW 170 °C, 10 min; (c) Fe, CH_3_COOH, EtOH, water, 60 °C, 1–2 h or Raney Ni, NH_2_-NH_2_ aq, MeOH/THF, 60 °C, 30 min; (d) R_2_COCl, py, rt, overnight.

**Scheme 3.**
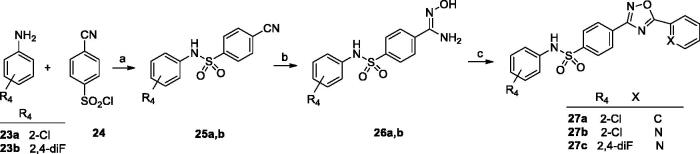
Reagents and conditions: (a) py, rt, overnight; (b) NH_2_OH·HCl, NaOH_aq_, EtOH, reflux, 1–5 h; (c) R_2_COCl, toluene, K_2_CO_3_, MW 170 °C, 10 min or rt, 30 min than reflux, 6 h.

The designed compounds representing all three chemotypes were synthesised according to the procedures outlined in Schemes 1–3.

Series A ligands were synthesised by a four-step pathway (Scheme 1) starting from the nucleophilic addition of hydroxylamine hydrochloride to 4-nitrobenzonitrile (**13**). The obtained benzamide oxime (**14**) was coupled with the corresponding acyl chloride to give 1,2,4-oxadiazoles (**15**). Further nitro group reduction led to amines (**16**), which in the last step were reacted with the appropriate acyl chlorides, resulting in final amides formation (**17a**–**g**).

The second type of ligands (series B) were prepared in a similar way starting from benzonitrile or pyridine-2-carbonitrile (**18**, Scheme 2). The reaction of N'-hydroxybenzimidamide (**19**) with 4-nitrobenzoyl chloride led to the corresponding 1,2,4-oxadiazoles (**20**), which were reduced to amines (**21**) and finally converted into amides (**22a**–**e**).

Synthesis of the series C ligands started from sulfonylation of the appropriate amine (**23a** or **23 b**) with 4-cyanobenzenesulfonyl chloride (**24**) to give the corresponding 4-cyanosulfonamides (**25a** and **25 b**). In the next step, the sulphonamides reacted with hydroxylamine hydrochloride to give benzamide oximes (**26a** and **26 b**), which were coupled with various acyl chlorides, leading to the final 1,2,4-oxadiazoles (**27a**–**c**) (Scheme 3).

The activity of the compounds was assessed *in vitro* in a forskolin-stimulated cAMP assay in T-REx 293 cells expressing human mGlu_4_ receptors in the presence of an EC_20_ concentration of L-Glu; a PAM was identified if it potentiated cAMP inhibition by the G_i/o_-dependent pathway of mGlu_4_ receptor activation; i.e. if it was able to produce a leftward shift in the potency of the endogenous agonist.

Among all tested compounds, only **17d** belonging to chemotype A exhibited PAM activity at mGlu_4_ receptor (EC_50_ = 3700 nM). The introduction of a moiety other than the picolinamide moiety on the left side (R_2_) of the 1,2,4-oxadiazole core in series A was not tolerated (**17c**, **17e**–**g**) as well as all derivatives from series B and C were found to be inactive.

**Figure 3.**
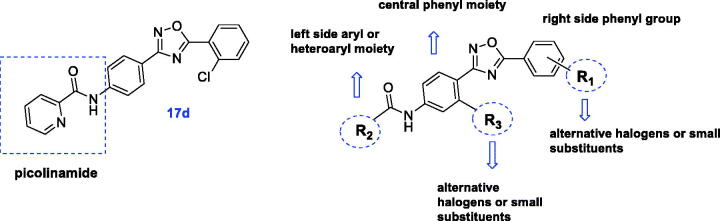
Chemical optimisation strategy for compound **17d**.

Taking into account that substituents R_1_ and R_2_ of compound **17d**, i.e. 2-chloro and 2-pyridyl, respectively, were identical to the parent molecule VU0366037, **17d** became the subject of subsequent modifications (Figure 3), and a new series of analogues (**28**–**62**) was synthesised.

Two synthetic paths were employed to allow modifications of the aromatic ring on the left, centre and right sides of molecule **17d**.

In the first path, the method already described for the synthesis of preliminary series A was applied to obtain compounds **28**–**32**. Additionally, commercially available 4-nitrobenzonitriles substituted at the 2 position were used to obtain derivatives **33**–**51** and **59**–**62**. For the second method, the synthetic approach described for the type C series was used. The starting 4-cyanosulfonyl chloride (**24**) was replaced by pinacolyl chloride, which was reacted with substituted 4-cyanoanilines to provide compounds **52**–**58**.

The results of the *in vitro* cAMP assay of derivatives **28**–**32** (Table 1) showed that the change in the position of the nitrogen atom (or its removal) on the left side of the pyridyl fragment as well as the additional substitution of fluorine or chlorine atoms to the 2-pyridyl ring led to a complete loss of activity. Thus, the picolinamide system was again identified as an important pharmacophore element for the allosteric modulation of mGlu_4_ receptor in the 1,2,4-oxadiazole-based derivatives.

**Table 1.** *In vitro* mGlu_4_ receptor PAM activity of 1,2,4-oxadiazole derivatives.

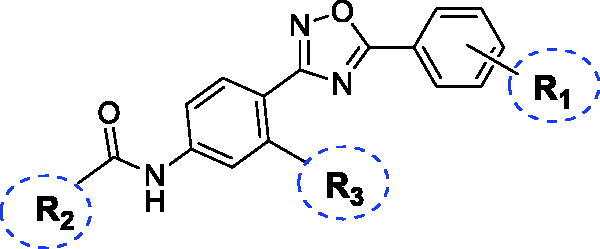						
Cmpd	R_1_	R_2_	R_3_	EC_50_^a^ [nM]	GluMax [%]	Fold shift^b^
**17d**	**2-Cl**	**2-pyridyl**	**H**	**3700**	**116**	1.8
**28**	2-Cl	3-pyridyl	H			NA^c^
**29**	2-Cl	4-pyridyl				NA
**30**	2-Cl	Ph				NA
**31**	2-Cl	6-F-2-pyridyl				NA
**32**	2-Cl	6-Cl-2-pyridyl				NA
**33**	H	2-pyridyl	OMe	1830	130	5.3
**34**	2-Cl	2-pyridyl		656 (138)^d^	114 (3.94)^d^	13.4 (2.00)^d^
**35**	3-Cl	2-pyridyl		5300	145	2.2
**36**	4-Cl	2-pyridyl		1980	157	2.0
**37**	2-F	2-pyridyl		393 (62)^d^	116 (1.77)^d^	3.68 (0.19)^d^
**38**	2-Cl,4-F	2-pyridyl		1980	124	4.6
**39**	2-Cl	6-F-2-pyridyl		920	135	2.0
**40**	2-Cl	5-Cl-3-F-2-pyridyl				NA
**41**	2-Cl	6-Cl-2-pyridyl		1920	115	4.1
**42**	2-Cl	3-Cl-6-MeO-2-pyridyl				NA
**43**	2-Cl	4,6-diF-2-pyridyl		2500	134	5.0
**44**	2-Cl	6-F-2-pyridyl		780	135	6.2
**45**	2-Cl	4-pirymidyl		3100	118	3.1
**46**	2-Cl	2- thiazolyl		2200	126	3.6
**47**	2-F	6-F-2-pyridyl		890	121	6.9
**48**	2-F	3,6-diCl-2-pyridyl				NA
**49**	H	2-pyridyl	Cl	352 (60)^d^	123 (3.24)^d^	6.77 (0.53)^d^
**50**	2-Cl	6-F-2-pyridyl				NA
**51**	2-Cl	6-Cl-2-pyridyl				NA
**52**	2-Cl	2-pyridyl		282 (46)^d^	123 (3.18)^d^	8.03 (0.76)^d^
**53**	2-Cl,4-F	2-pyridyl		1350	119	2.1
**54**	2-MeO	2-pyridyl		750	111	2.2
**55**	H	2-pyridyl	F	2560	127	3.0
**56**	2-Cl	2-pyridyl				NA
**57**	2-Cl,4-F	2-pyridyl				NA
**58**	2-MeO	2-pyridyl				NA
**59**	H	2-pyridyl	CF_3_	740	150	7.6
**60**	2-Cl	2-pyridyl		390 (57)^d^	120 (0.88)^d^	2.94 (0.1)^d^
**61**	H	2-pyridyl	Me	1650	128	5.1
**62**	2-Cl	2-pyridyl		308 (35)^d^	135 (3.65)^d^	3.3 (0.34)^d^

^a^Value determined in a presence of EC_20_ (3 µM) concentration of L-Glu (PAM-mode) in a forskolin-stimulated cAMP assay in T-REx 293 cells expressing human mGlu_4_ receptor; data are mean of two independent experiments; ^b^Fold shift of L-Glu dose-response curve determined in the presence of 10 µM of compound; ^c^NA: non-active; ^d^Mean from at least three independent experiments with SEM value in bracket.

Therefore, using the 2-pyridyl group as the basic fragment on the left side of the molecule, we modified the central ring by introducing various substituents at R_3_, such as OMe, Cl, F, CF_3_ and Me, at position 3 relative to the amide moiety, according to the SAR data of known mGlu_4_ receptor PAMs^9^. Modification to the 2-position (relative to the amide group) was not tolerated as previously indicated^57^. Additionally, modifications on the right side of the molecule (R_1_) were surveyed in parallel by the attachment of fluorine (instead of chlorine) at the 2 position of the phenyl ring or the introduction of an additional fluorine at the 4 position^23^^,^^57^^,^^69^.

Among compounds **33**–**48** containing a methoxy group attached to the central ring, only three compounds did not potentiate cAMP inhibition in response to mGlu_4_ receptor activation (**40**, **42**, and **48**), while the rest of the derivatives presented various levels of PAM activity for mGlu_4_ receptor (EC_50_ = 393–5300 nM). In comparison to compound **17d** without the methoxy substituent (EC_50_ = 3700 nM, Table 1), the most active derivative, **37**, with R_1_ = 2-F significantly enhanced positive modulation of the mGlu_4_ receptor (EC_50_ = 393 nM) and was also more active than VU0366037 (EC_50_ = 517 nM). The change from the fluorine atom to the chlorine atom (R_1_) weakened the potency of **34** (EC_50_ = 656 nM) and halogen removal (**33**), position change (**35**, **36**) or the introduction of an additional 4-fluorine substituent (**38**) caused an even more pronounced decrease in activity. Modifications within the 2-pyridyl fragment by the introduction of an additional fluorine and/or chlorine atoms to the 3, 5 and/or 6 positions generally weakened the activity of compounds **39**–**44**. In this group, only two derivatives, 6-F-2-pyridyl **44** (EC_50_ = 780 nM) and 3-F-2-pyridyl **39** (EC_50_ = 920 nM), showed a similar level of PAM potency as the corresponding 2-pyridyl analogue **34** (EC_50_ = 656 nM).

Subsequent modifications were made by replacing the OMe group at the R_3_ position with the following substituents: Cl, F, CF_3_ or CH_3_ (derivatives: **49**–**62**; Table 1); most of the compounds contained a picolinamide moiety on the left side, and two derivatives (**50**, **51**) had an additional fluorine or chlorine atom at the 3 position of the 2-pyridyl group (these last two compounds were, however, unable to modulate mGlu_4_ receptor activity).

Among the group of chlorinated derivatives, **49** (EC_50_ = 352 nM) and **52** (EC_50_ = 282 nM) were found to be the most active PAMs of mGlu_4_ receptor while analogues with R_1_ substituents other than 2-Cl (**53**, **54**) presented significantly lower levels of PAM potency (EC_50_ = 1350 and 750 nM, respectively).

Introduction of a fluorine atom to the central ring led to inactive compounds (**55**–**58**), and only derivative **55** (EC_50_ = 2560 nM) displayed some degree of activity at mGlu_4_ receptor. On the other hand, it seems that the presence of a trifluoromethyl (**59**, **60**) in this position could restore PAM activity since compound **60** with an EC_50_ = 390 nM was among the most active derivatives. Of the two compounds with a methyl substituent at the R_3_ position, **62** showed a significant level of PAM mGlu_4_ activity, whereas analogue **61** was less active (EC_50_ = 308 vs 1650 nM, respectively).

In summary, as a result of the *in vitro* evaluation of the mGlu_4_ receptor activity of the 1,2,4-oxadiazole derivatives, five compounds, **37**, **49**, **52**, **60** and **62**, showed an increase in mGlu_4_ receptor PAM potency compared with VU0366037, and one derivative (**34**) was found slightly less active. All derivatives contained the preferred picolinamide fragment on the left side of the 1,2,4-oxadiazole moiety, different small R_3_ substituents at the central aryl ring (OMe, Cl, CF_3_, Me), and small R_1_ groups on the right side (Cl, F, OMe).

### mGlu receptor selectivity

**Figure 4.**
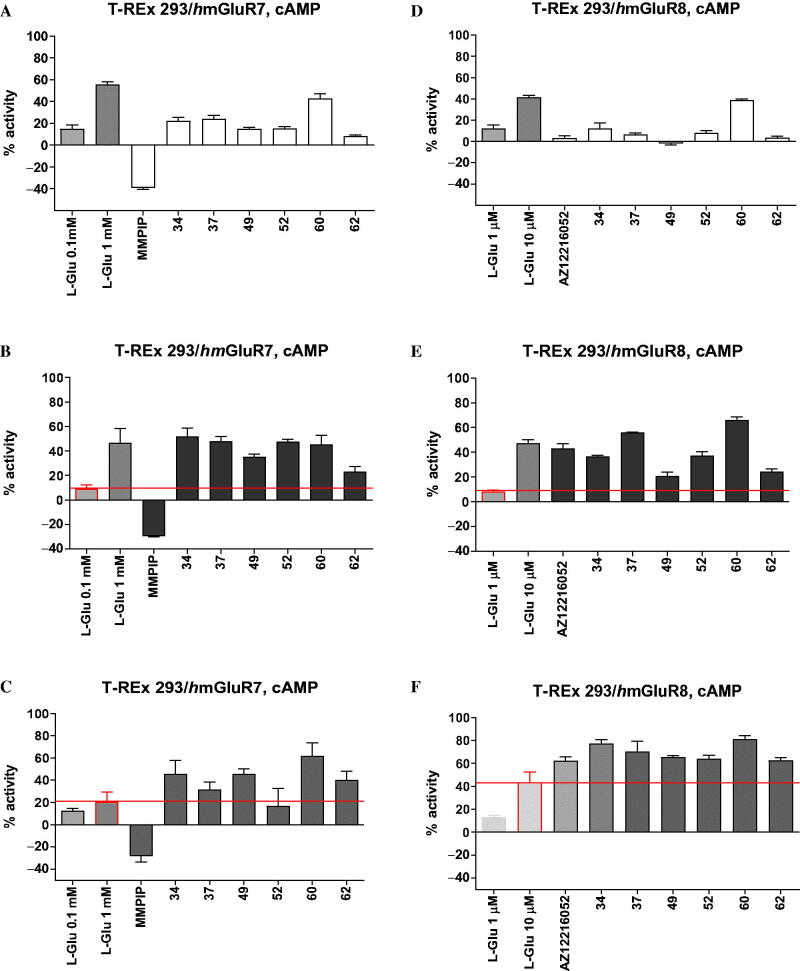
The activity of compounds **34**, **37**, **49**, **52**, **60**, **62** and reference drugs MMPIP (a selective NAM of mGlu_7_ receptor) and AZ12216052 (a PAM of mGlu_8_ receptor) tested at a concentration of 10 μM in the cAMP accumulation assay in cells expressing mGlu_7_ and mGlu_8_ receptors. The percent activity refers to two extreme FRET signals: 0% corresponds to forskolin treatment alone and 100% to maximal FRET signal for the control cells (without any treatment). **A/D** – agonistic activity; **B/E** – PAM activity measured in the presence of L-Glu at the EC_20_ concentration (0.1 mM for mGlu_7_ receptor, and 1 μM for mGlu_8_ receptor); **C/F** – NAM activity measured in the presence of L-Glu at the EC_80_ concentration (1 mM for mGlu_7_ receptor, 10 μM for Glu_8_ receptor).

Further *in vitro* characterisation included an assessment of potential direct agonist activation of mGlu_4_ receptor by the six most potent mGlu_4_ receptor PAMs (**34**, **37**, **49**, **52**, **60**, **62**) and determination of their selectivity for group III mGlu receptor members, i.e. mGlu_7_ and mGlu_8_ (Figure 4), as well as other types of mGlu receptors from group I (mGlu_1_, mGlu_5_) and group II (mGlu_2_) (Supplemental material Figures S3 and S4). The compounds were tested in the agonist, PAM (in the presence of EC_20_ concentration of L-Glu) or NAM (with EC_80_ of L-Glu) modes of forskolin-induced cAMP using T-REx 293 cells expressing the protein of a given receptor.

Only two derivatives, **34** and **37**, showed some agonist activity for mGlu_4_ receptor (EC_50_ = 1.11 µM and 3.94 µM, respectively), although their potencies were weaker than those observed for PAM activity and comparable to the agonist activity of the reference ago-PAM of mGlu_4_ receptor VU0155041 (EC_50_ = 2.5 µM)^54^.

With respect to mGlu_7_ and mGlu_8_ receptors, in addition to the pronounced PAM properties of all compounds, direct agonistic activity of **34**, **37**, **52**, and **60** at both receptors and direct agonistic activity of **49** at mGlu_7_ receptor were detected (Figure 4). At the same time, there was no significant cross-reactivity with mGlu_1,2_ and mGlu_5_ receptors (Supplemental material Figures S3 and S4).

Generally, the evaluated 1,2,4-oxadiazole derivatives positively modulated the group III mGlu receptors in a non-specific way, and their activity profile can be described as group III mGlu receptor selective. Of note is that derivative **62** lacked agonistic action in relation to any of the investigated members of the group III mGlu receptors and presented the properties of pure positive allosteric modulation. Additionally, none of the compounds examined caused a decrease in activity in the presence of the EC_80_ concentration of L-Glu at any of the investigated glutamate receptors, which indicates the lack of antagonistic properties of the discovered group III mGlu receptors allosteric modulators (Supplemental material Figures S2, S3, and S4).

### Preliminary safety and pharmacokinetic screening

The primary assessment of the cardiotoxic risk and mutagenic potential of the six selected 1,2,4-oxadiazole derivatives was performed in the hERG channel assay and the mini-AMES test, respectively. The mutagenicity potential was determined in strains TA98 (frameshift mutation) and TA100 (base-pair substitution) of *Salmonella typhimurium* in the presence and absence of an exogenous metabolic activation system (rat liver S9 fraction) containing mammalian microsomal enzymes.

All tested 1,2,4-oxadiazole derivatives (at 10 µM concentration) were inactive against hERG channels (Supplemental material Figures S8) and did not show mutagenic activity towards *S. typhimurium* TA98 and TA100 with and without S9 (at a concentration of ≤ 30 µM) (Supplemental material Figures S9).

Additionally, the most potent mGlu_4_ receptor PAM *in vitro*, derivative **52**, was tested for its ability to inhibit six key cytochrome P450 family isoenzymes involved in drug metabolism, showing only mild inhibition of the CYP2C9 and CYP2C19 isoenzymes (3.3 < IC_50_<10 µM).

**Figure 5.**
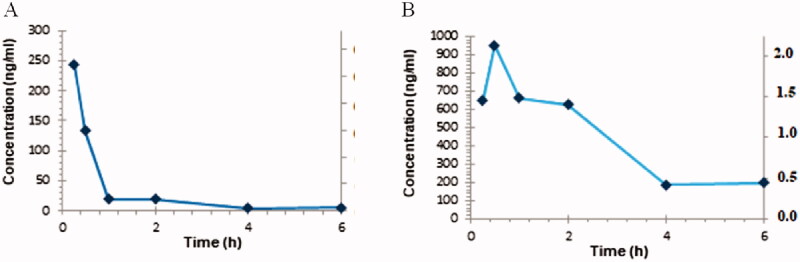
The time course of **52** after i.p. administration in mice: (A) plasma and (B) brain concentration-time profiles.

The pharmacokinetic properties and brain uptake of **52** were further determined in mice after i.p. administration. The compound was administered at a dose of 10 mg/kg in 3% DMSO + 20% Captisol in water. The concentration of **52** was monitored in the plasma and the brain at six time points over 6 h (Figure 5).

The pharmacokinetic analysis (Figure 5) showed that **52** was readily absorbed after i.p. administration (*T*_max_ = 30 min in the brain) (Table 2). The maximum brain concentration (949.76 ng/mL, 2.12 µM/L) was almost four times higher than the maximum plasma concentration (243.22 ng/mL, 0.54 µM/L). The analysed compound penetrated the blood-brain barrier after i.p. administration and peaked in the brain for longer than in plasma (Figure 5).

**Table 2.** Pharmacokinetic parameters in the mouse plasma and brain after a 10 mg/kg i.p. dose of **52**.

Parameters	Plasma	Brain
*T*_max_^a^	0.25	0.5
*T*_1/2_^b^	1.10	2.26
*C*_max_^c^	0.54	2.12
AUC^d^	0.37	5.64

*^a^*Time at maximum observed concentration [h]; ^b^terminal elimination half-life after i.p administration [h]; ^c^maximum concentration after i.p administration [µM/L]; ^d^area under the curve [µM/L × h].

### *In vivo* profiling

Although efforts to determine the therapeutic potential of group III mGlu receptor have focussed mainly on the role of the mGlu_4_ receptor subtype in Parkinson’s disease, it has been suggested that non-selective activation of group III mGlu receptors can be a promising therapeutic approach for the treatment of various neuropsychiatric disorders^8^^,^^70^. For example, the non-selective group III mGlu receptor agonist ACTP-I shows antianxiety-like properties in the mouse elevated plus maze and stress-induced hyperthermia tests^71^, antipsychotic-like activity in amphetamine-induced, MK-801-induced hyperactivity in rats and in DOI-induced head twitches in mice^72^, as well as antidepressant-like potency in the forced swimming test in rats^73^^,^^74^.

Taking into account the rather non-selective group III mGlu receptors character of the discovered PAMs, we investigated the anxiolytic, antipsychotic, and antidepressant-like potential of the selected 1,2,4-oxadiazole derivatives (**34**, **37**, **49**, **52**, **60**, **62**) in the relevant *in vivo* behavioural models, i.e. stress-induced hyperthermia (SIH) test, DOI-induced head twitch response (DOI-induced HTR), and tail suspension test (TST), respectively, according to previously described methods^48^^,^^75^.

#### Modified stress-induced hyperthermia in singly housed mice

**Figure 6.**
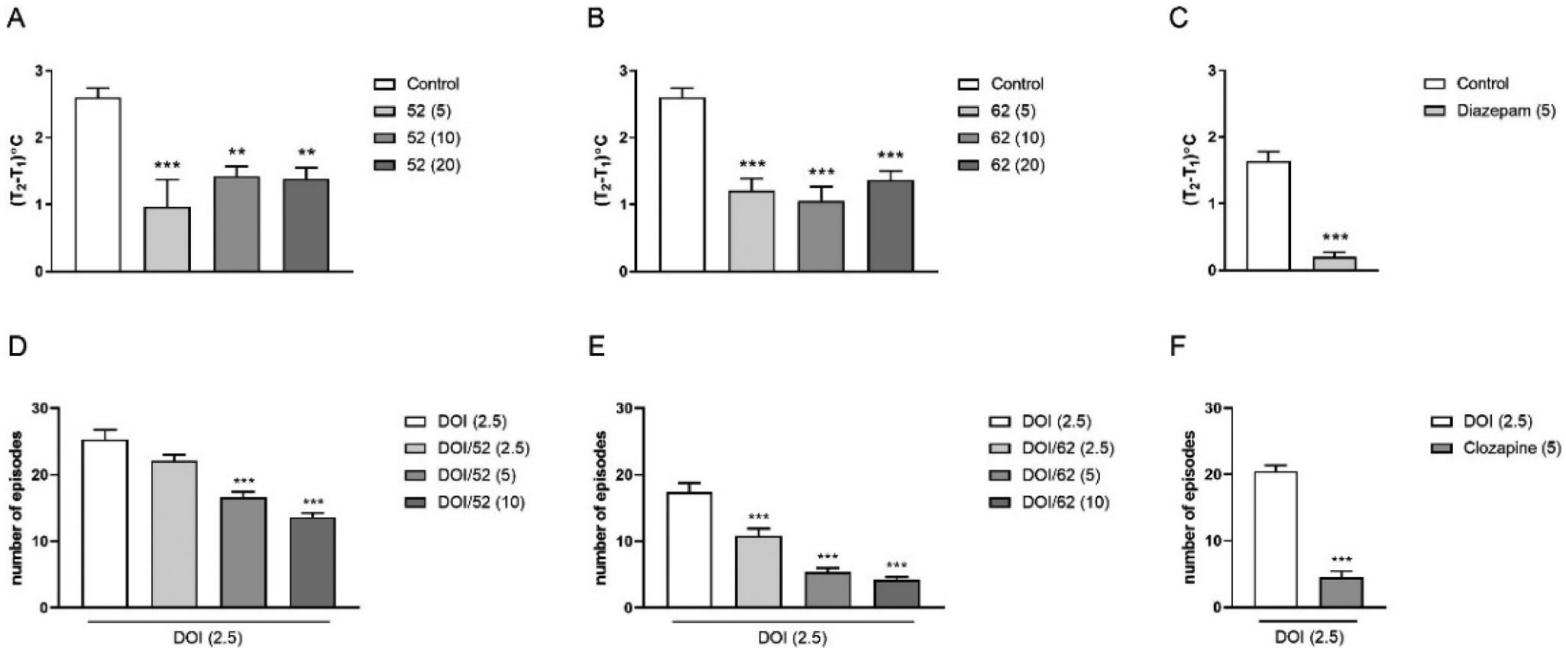
The effects of compounds **52** and **62** on (A, B) stress-induced hyperthermia and (D, E) DOI-induced HTR. Doses are indicated as mg/kg. Bars represent the mean ± SEM ***p < 0.001 vs. vehicle. Compounds **52** and **62** at all doses significantly reduced the SIH response: **52** [F_(3.34)_ = 9.35; *p* < 0.001], **62** [F_(3.36)_=17.12; *p* < 0.001]; and DOI-induced HTR: **52** [F_(3.36)_ = 28.14; *p* < 0.001]), **62** [F (3.16) = 38.24; *p* < 0.001]. The control drugs are presented in panels C (diazepam) and F (clozapine). ***p < 0.001 and **p < 0.01.

Reflecting autonomic aspects involved in anxiety and fear processes, SIH is an extremely useful procedure to measure potential anxiolytic-like effects of new drug candidates^76^. In the performed experiments, an injection of diazepam (5 mg/kg), used as a positive control, significantly reduced SIH (Figure 6). One-way ANOVA followed by Dunnett's *post hoc* analysis revealed different magnitudes of SIH response observed after administration of the selected 1,2,4-oxadiazole derivatives (Figure 6 and Supplemental material Figure S10). None of the administered doses of compound **42** statistically affected the SIH, and weak activity was detected for **37** at a dose of 10 mg/kg. In the case of **34**, dose-dependent efficacy was observed; however, a statistically significant reduction in SIH was observed only at higher doses (10 and 20 mg/kg) (Supplemental material Figure S10). Derivatives **52** and **62** significantly reduced SIH responses at all three doses (Figure 6(A,B)), whereas for 60, statistically significant changes in the SIH test were caused by the administration of two extreme doses, 5 and 20 mg/kg (Supplemental material Figure S10). At the same time, the investigated compounds had no effect on the basal core temperature.

#### DOI-induced HTR

The head-twitch behavioural response in rodents induced by the administration of the selective 5-HT_2A/2C_ agonist 1–(2,5-dimethoxy-4-iodophenyl)-2-aminopropane (DOI) has been proposed as an animal model of symptoms associated with a variety of behavioural and psychiatric conditions, including hallucinations. Due to the readily identifiable behaviour, it can be used to assess potential antipsychotic properties in the pre-clinical evaluation of CNS-active new agents.

All newly synthesised compounds, except **49** for which no influence on DOI-induced HTR was observed (Supplemental material Figure S11), significantly attenuated the number of head shakes in mice compared with the vehicle-treated control group. Clear dose-response effects were observed for derivatives **52** and **62** (Figure 6(D,E)), and the latter was the strongest blocking agent of the head twitch frequency, presenting activity comparable to that of clozapine, which was used in our study as a reference drug at a dose of 5 mg/kg (Figure 6(F)). For **37**, dose-dependent reverse effects were noticed, while for **34** and **60**, significant reductions in the HTR score were seen at two of the administered doses (2.5 and 10 mg/kg, respectively) (Supplemental material Figure S11).

#### Tail suspension test (TST)

The tail suspension is one of the most commonly used behavioural models for assessing antidepressant-like activity in mice. In contrast to imipramine (20 mg/kg) used as a reference drug, none of the tested compounds at doses of 5, 15 or 20 mg/kg significantly affected the immobilisation time of the mice (Supplemental material Figure S12).

In general, it has been observed that the 1,2,4-oxadiazole derivatives exhibit variable efficacy when tested in vivo. This is most likely due to different pharmacokinetic properties of examined compounds, but firm conclusions can only be drawn after more careful profiling of their drug-like parameters. Nevertheless, the obtained results of in vivo studies clearly indicate the anxiolytic and antipsychotic properties of the tested PAMs of mGlu group III receptors. Indeed, as shown in previous literature reports, group III mGlu receptors are thought to be implicated in such pathological conditions^8^^,^^70^. In particular, the anxiolytic effects of the abovementioned non-selective group III agonist ACTP-I^71^, as well as a number of mGlu_4_ receptor PAMs such as Lu AF21934, have been observed in SIH and other acute models used to study anxiety-related behaviour in rodents^48^. Moreover, both agents have been shown to have an antipsychotic-like profile in various rodent models, including DOI-induced HTR in mice^72^^,^^77^. Thus, except for compound **42** which failed to alter SIH and block DOI-induced HTR in mice, the pharmacological in vivo profiles of **34**, **37**, **52**, **60** and **62** resemble that of the non-selective group III agonist ACTP-I and various mGlu_4_ receptor PAMs investigated to date^78^. On the other hand, the lack of antidepressant-like effects of the 1,2,4-oxadiazole derivatives in the TST is similar to our earlier studies, in which we did not find such activity for either the group III agonist ACTP-I^71^ or the selective mGlu_4_ receptor PAM Lu AF21934^48^.

Considering the in vivo properties, the effects of compound **62** deserves special mention in the DOI-induced HTR compared with the efficacy of ADX88178 reported by Addex Therapeutics as a selective and brain-penetrable mGlu_4_ receptor PAM. In contrast to **62**, which presented a clear dose-dependent response and high potency, ADX88178, when tested in doses 3, 10 and 30 mg/kg, lacked dose dependency and showed a flat profile in the reduction of DOI-induced HTR that did not exceed 30% for the medium dose^62^ only.

Despite the strongly postulated role of glutamate hyperactivity in the pathophysiology of schizophrenia^8^^,^^79^, animal data linking mGlu receptor group III function to this disease are limited. The majority of the currently known results come from our studies^72^^,^^77^^,^^80^ and describe the efficacy of group III mGlu receptor allosteric modulators, indicating that mGlu_4_ receptor is the primary target for their antipsychotic-like activity^78^^,^^81^. However, the strong in vivo potency of **62**, revealed from the DOI-induced HTR, shows the antipsychotic potential of less selective group III mGlu receptor agents. This seems to be confirmed by recently published results describing the important role of group III mGlu receptor activation in the clozapine mechanism of action^82^. Therapeutically relevant concentrations of clozapine inhibited thalamocortical hyperglutamatergic transmission by activating presynaptic inhibitory group III mGlu receptors in the medial prefrontal cortex^82^. Thus, the stimulation of group III mGlu receptors on the glutamatergic terminals of thalamocortical neurons counteracting glutamatergic hyperactivity in schizophrenia might contribute to the efficacy of specific agents, which can be observed in behavioural models predicting antipsychotic activity.

## Conclusions

Although the present study was aimed at obtaining selective PAMs of mGlu_4_ receptor active compounds from the series of 1,2,4-oxadiazole derivatives should be classified as group III mGlu receptor-preferring PAMs. To some extent, this may be due to the highest amino acid sequence homology observed between members of the same mGlu receptor group. The group III mGlu receptor subtypes share more than 94% sequence identity in the transmembrane binding pocket, while a comparison of the corresponding amino acid sequence between the group III mGlu receptors and group I and II mGlu receptor subtypes shows similarity in the range of 65–81%^83^. Nevertheless, in the identified group III mGlu receptor PAMs, we discovered potent in vivo compounds evoking a decrease in anxiety-related behaviour assessed using the SIH test, and antipsychotic-like properties from the DOI-induced HTR. Moreover, our finding that the antipsychotic-like effect of **62** was comparable to that of clozapine in the DOI-induced head twitch test and better than that reported for the selective mGlu_4_ receptor PAM ADX88178 might be clinically relevant and requires further pharmacological evaluation.

## Materials and methods

### Chemistry

#### Chemicals

All organic reagents were purchased from commercial suppliers and were used without purification. Solvents and inorganic reagents were acquired from Chempur or POCh (Poland). Reaction progress was monitored by TLC on Merck Silica Gel 60 F_254_ on aluminium plates or Merck Aluminium oxide 60 F_254_, neutral on aluminium plates and visualised with UV light (254 nm). Column chromatography was performed on Merck Silica Gel 60 (0.063–0.200 mm; 70–230 mesh ASTM) or on Merck Aluminium oxide 90 active neutral (0.063–0.200 mm; 70–230 mesh ASTM).

#### Software

MarvinSketch software was used for drawing, displaying and characterising chemical structures, substructures and reactions, Marvin 17.24.0, 2017, ChemAxon. JChem Base was used for structure searching and chemical database access and management, JChem 18.3.0, 2018, ChemAxon (www.chemaxon.com).

#### General procedures

##### General procedure 1 for carboximidamide formation

*Method A*^84^: In a round-bottom flask, the starting benzonitrile (10.0 mmol, 1.0 eq) was dissolved in EtOH (40 ml), and hydroxylamine hydrochloride (20.0 mmol, 2.0 eq) was added followed by a solution of NaOH (20.0 mmol, 2.0 eq) in water (10 ml) (Schemes 1(a) and 2(a)). The reaction mixture was refluxed for 6 h (TLC control), and then evaporated to dryness. Water (50 ml) was added, and the reaction mixture was acidified with 1 N HCl. At this stage, for a few compounds, a yellow solid was precipitated, filtered and identified as a corresponding benzamide byproduct. The filtrate was extracted with AcOEt, and the water layer was alkalised with NH_3_aq. The crude product was extracted with CHCl_3_ or filtered directly as a precipitated solid and further purified by maceration from iPrOH/hexane (1:3).

*Method B*^85^: In a round-bottom flask, the starting benzonitrile (16.0 mmol, 1.0 eq) was dissolved in EtOH (80 ml) and hydroxylamine hydrochloride (80.0 mmol, 5.0 eq) was added followed by TEA (96.0 mmol, 6.0 eq). The reaction mixture was refluxed for 3 h (TLC control), and the EtOH was evaporated *in vacuo*. Water (100 ml) was added, and the reaction mixture was extracted with AcOEt. After evaporation of the solvent, the crude product was purified by column chromatography over silica gel using a gradient of CHCl_3_ to CHCl_3_/MeOH (49:1) followed by trituration with iPrOH/hexane (1:2).

##### General procedure 2 for 1,2,4-oxadiazole formation

To a round-bottom flask containing toluene (15 ml), a carboximidamide (2.0 mmol, 1.0 eq) and the required acid chloride (2.6 mmol, 1.3 eq) was added followed by K_2_CO_3_ (2.6 mmol, 1.3 eq)^86^^,^^87^ (Schemes 1(b) and 2(b)). After 30 min of stirring at rt, the reaction mixture was refluxed until the TLC showed the end of the reaction (5–25 h). The reaction mixture was poured into water (40 ml) and extracted with CHCl_3_. The crude product was purified by column chromatography or by maceration in iPrOH/hexane (1:2). The reaction was also carried out in a MW oven by suspending all reagents in toluene (4 ml) in a reactor flask and heating to 170 °C for 10 min to complete the reaction.

##### General procedure 3 for reduction of the NO_2_ group

*Method A*: Reduction was carried out with Fe powder in the presence of 90% CH_3_COOH according to the method described in the literature^88^(Schemes 1(c) and 2(c)).

*Method B*: To a solution of nitrooxadiazole (1.0 mmol, 1eq) in EtOH (15 ml), SnCl_2_ (4.0 mmol, 4.0 eq) dissolved in 5 N HCl (1.5 ml) was added dropwise. The reaction mixture was refluxed for 5 h (TLC control), and the solvent was removed *in vacuo*. Water (30 ml) was added, and the reaction mixture was alkalised with 2 M NaOH to pH = 9. The resulting suspension was extracted with AcOEt. Evaporation of the solvent afforded the crude amino derivative, which was further purified by column chromatography followed by maceration from iPrOH/hexane (1:2).

*Method C*: Reduction was performed in MeOH in the presence of Raney Ni and hydrazine hydrate according to known procedures^89^.

##### General procedure 4 for amide formation from acyl chlorides

The starting substituted aniline (0.75 mmol, 1.0 eq) was dissolved in pyridine (3 ml). The corresponding acyl chloride (0.98 mmol, 1.3 eq) was added in one portion. After stirring overnight at room temperature, the reaction mixture was poured into water (50 ml). The precipitated solid was filtered and dried. The crude product was purified by column chromatography or maceration.

##### General procedure 5 for amide formation from carboxylic acids

BOP (0.88 mmol, 1.6 eq) and the corresponding carboxylic acid (0.83 mmol, 1.5 eq) were dissolved under argon in anhydrous MeCN (10 ml). TEA (1.10 mmol, 2.0 eq) was added dropwise, and after 30 min of stirring at rt, a solution of the required aniline (0.55 mmol, 1.0 eq) in MeCN (5 ml) was added to the reaction mixture. Stirring continued until TLC showed disappearance of the starting aniline. The precipitated solid was filtered, washed with MeCN and dried. In cases where the reaction mixture was homogenous, water (50 ml) was added, and the product was extracted with CHCl_3_. The organic layer was dried, and the solvent was evaporated *in vacuo*. The crude product was purified by column chromatography or maceration.

### *In vitro* pharmacology

#### Drugs

Reference compounds: AZ 12216052, CBiPES, VU0155041, VU0469650, VU1545, MPIPP, and Ro 67–4853 were purchased from Tocris Bioscience, and the mGlu receptor agonist L-Glu was purchased from Sigma Aldrich. 3–(2-Pyridyl)-5–(2-chlorophenyl)-1,2,4-oxadiazole was synthesised in-house according to the method previously described^90^.

#### Generation of cDNA constructs and cell lines

Generation the of cell lines with tetracycline-inducible expression of human metabotropic receptors 4, 7 and 8 was described in detail by Chruścicka et al.^91^ (mGlu_4_ receptor NM_000841, mGlu_7_ receptor NM_000844.2, and mGlu_8_ receptor NM_000845) (Figure S5 and Figure S6, Table S1). Additionally, a new cell line with the human mGlu_2_ receptor was obtained. The sequence of GRM2 (NM_000839) was subcloned from pcDNA3.1+ (supplied from the University of Missouri–Rolla U.S.) into the multicloning side of the pcDNA5/FTR/TO vector (Invitrogen, Carlsbad, CA) using BamHI and XhoI restriction enzymes. After restriction analysis, T-REx 293 cells were transfected with the received plasmid and expression of mGlu_2_ receptor in the cells was analysed by RT-PCR and Western blot (Figure S5 and Figure S6). In a similar way, cells with inducible expression of GRM1 (NM-000838.2) and GRM5 (NM_000842.1) were prepared. GRM1 was subcloned into the pcDNA5/FTR/TO vector by the XhoI enzyme (blunt ends) and GRM5 by XhoI and XbaI (blunt ends). Both were inserted initially in the pCMV6-XL vector (Origene). Cells were cotransfected with pcDNA5/FRT/TO-GRM and pOG44 plasmid coding Flp recombinase. Next, stably transfected clones were established by antibiotic selection (hygromycin). The presence of the *h*mGlu_1_ receptor or *h*mGlu_5_ receptor protein was detected in cell lysates by Western blotting with mouse anti-human mGlu_1_ or mGlu_5_ receptors monoclonal antibodies (both from R&D Systems) (Figure S7). Cells were grown under standard cell culture conditions (37 °C, 5% CO_2_) in DMEM supplemented with 10% tetracycline-free FBS. The expression was induced with tetracycline added to culture medium at 0.75 μg/mL.

#### Forskolin-induced cAMP production assay

Determination of the intracellular cAMP through a homogeneous time-resolved fluorescence (HTRF) cAMP dynamic 2 kit from Cisbio (Codolet, France) was described previously by Chruścicka et al.^91^. Briefly, cells were grown in DMEM supplemented with 10% foetal bovine serum (FBS) (tetracycline free). Forty-eight hours before the experiments, mGlu receptor expression was induced by the addition of 0.75 µg/mL tetracycline. Twenty hours before the experiment, FBS and L-Glu were removed from the medium. Thereafter, the cells were scraped and centrifuged. A cell pellet was suspended in Hanks-HEPES (130 mM NaCl, 5.4 mM KCl, 1.8 mM CaCl_2_, 0.8 mM MgSO_4_, 0.9 mM NaH_2_PO_4_, 20 mM HEPES, and 3.25 mM glucose; pH 7.4). Then, the cell suspension was incubated in the presence of 10 µM forskolin (or 3 µM forskolin for mGlu_7_ receptor), the agonist L-glutamate and a compound for 5 min. Next, 10 µl of the cell suspension was mixed with 5 µl of cAMP-d2 conjugate and 5 µL of anti-cAMP cryptate conjugate. After 1 h of incubation at rt, the fluorescence at 620 nm and 665 nm was read (Tecan Infinite M1000). The results were calculated as the 665 nm/620 nm ratio multiplied by 10^4^. Each sample was prepared in triplicate.

#### Calcium flux assay

Cells, 7000 per well, were plated two days before the assay in 384-well clear bottom black wall plate (BD PureCoat Amine, 354719) in FluoroBrite DMEM with 2 mM L-Glu, sodium pyruvate, 10% dialysed FBS, blasticidin and hygromycin. Tetracycline (0.75 mg/mL) was added to cells for 3 h, deprived from FBS and induced for 1 h in the presence of tetracycline (0.75 mg/mL), 2 mM Fluo-8 AM, 0.1% Pluronic F-127, 2 mM probenecid, and 0.04% trypan blue in Hanks-HEPES buffer. Cells were incubated with Fluo-8 loading for 20 min at 37 °C, followed by 15 min at RT. The fluorescence signal was measured on a uCell (Hammamatsu; Japan). Modulators were added after 40 s of the measurement procedure, and L-glutamate was added at 4 min 30 s for a V_final_ = 60 μL. The total time of measurement was 7 min 30 s (0.5 s intervals). For analysis, the data used were from 4 min 30 s to 7 min 30 s.

Statistical calculations were performed using GraphPad Prism 5.04 software (GraphPad Software, La Jolla, CA, USA). The curves were fitted to a 3-parametric logistic equation, allowing for the determination of EC_50_ values. Each data point was analysed in triplicate.

### Safety screening

#### *In vitro* binding to hERG assay

The propensity of the tested compounds to block human hERG potassium channels was investigated in a whole cell patch clamp assay in CHO-K1 cells expressing hERG channels by BLIRT, Gdańsk, Poland. Experiments were performed using CHO-K1 cells stably transfected with the hERG potassium channel. hERG potassium current was recorded with whole-cell patch-clamp technique at room temperature using an Axopatch 200B amplifier (Axon Instruments, CA, USA) and a CV203BU head‐stage. Data were acquired through a DigiData 1200 Series (Axon Instruments, CA, USA). Solutions were perfused using a rapid solution changer RSC-200 (Bio-Logic - Science Instruments, Claix, France) connected to an EVH-9 system. Patch‐pipettes were pulled using a PP-830 pipette puller (Narishige, Tokyo, Japan) and polished in a microforge MF-830 (Narishige, Tokyo, Japan). The extracellular recording solution was: 140 mM NaCl, 2,8 mM KCl, 2 mM, CaCl_2_, 2 mM MgCl_2_, 10 mM glucose, 10 mM HEPES; titrated to pH 7.3 with NaOH. During the experiments, the tested cell was washed with a control solution (extracellular solution with the addition of 0.1% DMSO) or with a tested compound solution (extracellular solution with the addition of tested compound in concentration: 10 µM dissolved in DMSO, the total amount of DMSO was 0.1% and did not influence on hERG channels).^92^ In the protocol of the experiments, all recordings were performed in duplicate. In the first recording, the cell was washed with a control solution, in the second recording with a solution containing a test compound (the cell was washed with the test compound at least 1 min before starting recording). During the analysis, the intensity of the current flowing through the membrane at a given potential was measured and then normalised to the maximum intensity observed for a given control recording. Then the change of the normalised current was calculated. The experiments and controls were repeated several times using different cells each time. In the end, the average value of normalised current at the given potential was calculated. In all the experiments, the characteristics of potassium currents flowing through the cell membrane changed with time. Therefore, a single experiment consisted of two records comparing the change in the intensity of the current.

#### Inhibition of the cytochrome P450 isoform activity (screening)

The assays for cytochrome P450 inhibition facilitate the identification of drug candidates with a lower potential for drug-drug interactions. In vitro experiments conducted to determine whether a drug inhibits a specific CYP enzyme involve an incubation of the drug with probe substrates for the CYP enzymes.

Recombinant cytochrome P450 isoforms available for the assay: CYP1A2, CYP2B6, CYP2C9, CYP2C19, CYP2D6 and CYP3A4 isoform with various probe substrates enabling fluorescence detection.

The examined compound was prepared as a 10 mM stock solution in DMSO. The following three concentrations of the test compound were used for screening determination: 1.1, 3.3 and 10 μM. Reference compounds were prepared as a stock solution in DMSO at the following concentration: KTZ 0.025 mM (3A4), SFZ 0.5 mM (2C9), TCP 5 mM (2C19), TCP 25 mM (2B6), Furafylline 2.5 mM (1A2), Quinidine 25 μM (2D6). The assay was performed on 96-well microtiter plates (Microplate 96 well, PS, F-botton, black, non-binding, Greiner BIO-ONE). Plates filled with solution of cofactors (NADP, G6P, MgCl_2_, G6PDH) as well as examined compound were scanned with a fluorescence plate reader in order to eliminate false results originating from autofluorescence of the test compounds. Isoform-specific substrates: 0.2 mM BFC (3A4), 0.15 mM MFC (2C9), 0.05 mM CEC (2C19), 0.01 mM CEC (1A2), 25 mM EFC (2B6), 7.7 × 10^−5 ^mM AMMC (2D6) were prepared in 0.5 M PBS buffer pH = 7.4 and incubated at 37 °C individually with CYP enzymes (Supersomes Human, Corning). After 10 min of preincubation, isoform/substrate solutions were transferred to black plates which were incubated 30 min for 3A4, 2C19, 2B6, 1A2, 2D6 and 45 min for 2C9. At the end of the incubation, the reaction was stopped and the fluorescence of the product was determined continuously with the specific for every product excitation as well as emission values with the use of multiwell plate scanner equipped with fluorescence measurement (Fluorescence EnSpire Multimode Plate Reader).

#### Mini AMES test

The Ames microplate fluctuation protocol (MPF) assay was performed with *Salmonella typhimurium* strains TA98 and TA100, enabling the detection of base-pair substitution. The bacterial strain as well as the exposure and indicator medium were obtained from Xenometrix AG (Allschwil, Switzerland). The mutagenic potential of the tested structures was evaluated by incubation of the bacteria with the test compound at concentrations of 1 mM and 10 mM for 90 min (37 °C) in exposure medium containing a limited amount of histidine. After the addition of indicator medium, each well of the 24-well plate was aliquoted into 48 wells of a 384-well plate.

The occurrence of reversion events to histidine prototrophy was observed as the growth of bacteria in the indicator medium without histidine after 72 h of incubation at 37 °C. Bacterial growth in 384-well plates was visualised by a colour change of medium from violet to yellow due to the addition of pH indicator dye. The absorbance was measured with a microplate reader (EnSpire) at 420 nm. The reference mutagen NQNO (0.5 mM) was used as a positive control in the experiments. The medium control baseline (MCB) was calculated, as derived from the mean number of revertants in the medium control plus one standard deviation.

### Pharmacokinetic studies

The method described below was successfully applied to a pharmacokinetic study of **52** in mice (male Albino Swiss) after i.p. injection. Compound **52** was administered to mice at a dose of 10 mg/kg i.p. At 0.25, 0.50, 1.0, 2.0, 4.0, and 6.0 h (three animals were used for one time point) the mice were anaesthetised by the use of Morbital (Biowet, Puławy, Poland), and the blood was collected from the portal vein to tubes containing 5% EDTA. The mice were then perfused with 0.1 M PBS to remove remaining blood from the body, and the brains were taken out for the analysis. Blood was centrifuged at 2000 rpm for 10 min at 4 °C, and the plasma was collected and frozen at −80 °C for further analysis. Plasma and tissue samples from all drug-treated animals were thawed at room temperature prior to use. Standard protocol of sample preparation: 200 µL of acetonitrile was added to the Eppendorf tubes with 50 µL of studied plasma samples or tissue homogenate. Samples were mixed for 5 min on a mixer at 25 °C and 1400 rpm. Tubes were then centrifuged at 2000 × g for 15 min at 4 °C. A total of 180 µL of each supernatant was transferred to a plate well. Finally, each sample was injected onto the LC-MS column. Calibration curve serial dilution method: Plasma was spiked with a standard at different concentrations. Acetonitrile was added. After mixing and centrifugation, the supernatant was collected.

#### LC-MS analysis

##### Chromatographic conditions

Plasma and tissue samples from all drug-treated animals at selected time points were analysed using a previously developed non-validated LC-MS/MS method. A sensitive and highly selective liquid chromatography-tandem mass spectrometry (LC-MS) method was used to determine the drug concentration in mouse plasma samples or tissue homogenates. LC-MS analysis was carried out on a Bruker amaZon SL mass spectrometer using positive/negative ion ESI mode. Chromatographic separation was achieved on an Ascentis Express C18 column (5 cm × 2.1 mm, 2.7 µM, Supelco Technologies) at room temperature with a thermostatted column oven. A gradient elution of eluents A (acetonitrile (LiChrosolv, Reag. Ph Eur) + 0.1% formic acid (Sigma Aldrich, 98–100%)) and B (water +0.1% formic acid) was used for separation. The flow rate was set at 1 ml/min. The injection volume was 20 µL, and the time of injection was 4 min.

##### Mass spectrometric conditions

An ion trap mass spectrometer (Bruker amaZon SL) was equipped with an electrospray source operating in positive/negative ion mode. Data were collected and processed using Bruker Quant Analysis software. Quantification of the analytes was performed in SIM mode.

### In vivo pharmacology

#### Animals and housing

Male Albino Swiss mice (20–25 g) and male C57BL/6J mice (20–22 g) were used in DOI-induced HTR and SIH tests and in the TST, respectively. All mice were 5–6 weeks old and were purchased from Charles-River Company (Germany). The animals were housed under standard laboratory conditions of lighting (light phase 6:00–18:00 h), temperature (19–21 °C) and humidity of 50% with food and water freely available. The experimental groups consisted of five to ten animals, depending on the experimental protocol. The experiments were carried out between 10:00–14:00 h by an observer blind to the treatment. All procedures were conducted according to the guidelines of the National Institutes of Health Animal Care and Use Committee and were approved by the Ethics Committee of the Institute of Pharmacology, Polish Academy of Sciences in Krakow.

#### Drugs and treatment

The compounds were dissolved in a small amount of 100% EtOH and then adjusted with 20% cyclodextrin, and the pH was adjusted to 6.0. The investigated compounds were administered intraperitoneally (i.p.) 60 min before the behavioural test. Control drugs i.e. diazepam, clozapine and imipramine (Sigma–Aldrich, St. Louis, USA), were administered i.p. 60 min before the behavioural test. Cyclodextrin (20%) was used as a vehicle. All solutions were prepared immediately prior to the experiments and were administered at a constant volume of 10 ml/kg.

#### Modified stress-induced hyperthermia in singly housed mice

The procedure for modified stress-induced hyperthermia was adapted from Van der Heyden^93^ and based on the procedure introduced by Borsini^94^. Each experimental group consisted of eight to ten animals. The animals were housed individually in a 26 × 21 × 14 cm Macolon cage 24 h before testing. For this assay, the body temperature was measured for each mouse at *t* = 0 min (*T*_1_) and *t* = +15 min (*T*_2_). Albino Swiss mice were placed into a new cage immediately following *T*_1_, with the difference in temperature (*T*_2_−*T*_1_) used as the measure of stress-induced hyperthermia. Pilot studies by Spooren demonstrated that a *T*_2_−*T*_1_ interval of 15 min is optimal for SIH assays^95^. A comparison between *T*_1_ in vehicle-treated mice and those administered the test compound was used to determine whether the agent affects body temperature alone. Diazepam (5 mg/kg) was used as the positive control. The rectal temperature was measured to the nearest 0.1 °C with a Physitemp Theralert thermometer, TH-5, Clifton NJ, USA, with the temperature sensor for mice, Type T, Copper-Constantan Thermocouple, Braintree Scientific Inc. The lubricated thermistor probe (2 mm diameter) was inserted 20 mm into the rectum. The mouse was held at the base of the tail during this determination, and the thermistor probe was left in place until a constant reading was obtained for 15 s. The effects of the investigated compounds on the SIH response were investigated after administration of the compounds at doses of 5, 10 and 20 mg/kg. The mean basal temperature (*T*_1_) of the mice did not differ between the groups. The vehicle used (20% (2-hydroxypropyl)-β-cyclodextrin) did not have any influence on the basal body temperature, which was typically between 36 and 37 °C for the Albino Swiss mice used in our laboratory.

#### DOI-induced head twitch test

The experiments were performed according to the procedure described in our previous studies^75^^,^^80^. Briefly, to habituate mice to the experimental environment, each animal was transferred to a 12 cm (diameter) × 20 cm (height) glass cage lined with sawdust 30 min before treatment. Test compounds were administered intraperitoneally (i.p.) at doses of 2.5, 5, and 10 mg/kg body weight 60 min before the test was performed. The head twitch response (HTR) in mice was induced by DOI (2.5 mg/kg, i.p.). Immediately after treatment, the number of head twitches was counted during a 20 min session. Haloperidol and clozapine were used as the reference compounds at active doses of 0.2 and 5 mg/kg, respectively.

#### Tail suspension test

The tail suspension test was performed according to the procedure of Steru^96^. Imipramine (20 mg/kg, i.p.) was used as a reference drug. C57BL/6J mice were individually suspended by their tails by a plastic string that was positioned horizontally 75 cm above the tabletop using adhesive tape placed approximately 1 cm from the tip of the tail. The immobility duration was recorded for 6 min. The mice were considered immobile only when they hung down passively and were completely motionless.

## Supplementary Material

Supplemental Material
