# Supplementary material for: New 1,2,4-oxadiazole derivatives with positive mGlu4 receptor modulation activity and antipsychotic-like properties
Source: J Enzyme Inhib Med Chem. 2021 Dec 11;37(1):211–25. doi: 10.1080/14756366.2021.1998022 (PMC8667925; doi:10.1080/14756366.2021.1998022)
Supplement: Supplemental Material [file IENZ_A_1998022_SM2515.pdf]

Supplemental material

New 1,2,4-oxadiazole derivatives with positive $mGlu_4$ receptor modulation activity and antipsychotic-like properties

Anna Stankiewicz[a], Katarzyna Kaczorowska[a,*], Ryszard Bugno[a], Aneta Kozioł[a], Maria H. Paluchowska[a], Grzegorz Burnat[b], Barbara Chruścicka[b], Paulina Chorobik[b], Piotr Brański[b], Joanna M. Wierońska[b], Beata Duszyńska[a], Andrzej Pilc[b], Andrzej J. Bojarski[a,*]

[a] *Department of Medicinal Chemistry,* [b] *Department of Neurobiology, Maj Institute of Pharmacology, Polish Academy of Sciences, 12 Smętna Street, 31-343 Kraków, Poland*

Corresponding Author:

E-mail: k.kaczor@if-pan.krakow.pl

Postal address: 12 Smętna Street, 31-343 Kraków

Phone: +48 792287874

Fax: +48 12 637 45 00

Table of contents:

1. **Chemistry** **(page 3)**

2. **In vitro mGluR selectivity profile** **(page 129)**

Figure S1. Activity of: **34**, **37**, **49**, **52**, **60** and **62** at untransfected T-REx 293 cells (control experiments).

Figure S2. Activity of: **34**, **37**, **49**, **52**, **60** and **62** at $mGlu_4$ receptor measured in NAM mode.

Figure S3. Activity of: **34**, **37**, **49**, **52**, **60** and **62** at $mGlu_1$ receptor and $mGlu_5$ receptor measured in agonistic, PAM and NAM modes.

Figure S4. Activity of: **34**, **37**, **49**, **52**, **60** and **62** at $mGlu_2$ receptor measured in agonistic and PAM modes.

Figure S5. mRNA expression of mGluR 2, 4, 7 and 8 analysed by RT-PCR in transfected or not T-REx 293 cell line after 24h Tet induction.

Table S1. RT-PCR primer sequences.

Figure S6. Analysis of the expression of $mGlu_2$, $mGlu_4$, $mGlu_7$, and $mGlu_8$ by the Western blot method, in the T-REx 293 cell lines.

Figure S7. Analysis of the expression of $mGlu_1$ and $mGlu_5$ by the Western blot method, in the T-REx 293 cell lines.

3. **Preliminary safety** **(page 135)**

3.1. ***In vitro binding to hERG assay***

Figure S8. Voltage-activation curves of compounds **34**, **37**, **49**, **52**, **60**, **62**

3.2. ***Mutagenicity potential-mini-AMES***

Figure S9. The AMES test revealed no mutagenic potential for **34**, **37**, **49**, **52**, **60** and **62**

4. **In vivo tests** **(page 141)**

Figure S10. Effects of compounds: **34**, **37**, **49**, **60** in SIH.

Figure S11. Effects of compounds: **34**, **37**, **49**, **60** on DOI induced HTR.

Figure S12. Effects of compounds: **34**, **37**, **49**, **52**, **60**, **62** in TST.

1. Chemistry:

Analytical Methods. ^{1}H NMR spectra were measured at 300 MHz and ^{13}C NMR spectra at 75 MHz on a Varian Mercury-VX (300 MHz) spectrometer in $CDCl_3$ or DMSO-d_6 solutions with TMS as an internal standard. The spectral data of new compounds refer to their free bases. Chemical shifts are expressed in δ (ppm). Splitting patterns describe apparent multiplicities and are designated as s (singlet), d (doublet), t (triplet), q (quartet), m (multiplet), and br s (broad singlet). Coupling constants are given in units of hertz (Hz). For compounds containing fluorine atoms, couplings from C-F were observed on ^{13}C carbon spectra. In cases where it was possible, multiplets in ^{13}C NMR were identified and denoted in the experimental part. All final compounds were purified to > 95% purity (*Note: some nitro intermediates were not fully characterized due to its poor solubility and difficulties with purification*) as assessed by UPLC/MS analysis performed on Waters TQD spectrometer combined with UPLC Acquity H-Class with PDA eLambda detector. Waters Aquity UPLC BEH C18 1.7 μm, 2.1×100 mm chromatographic column was used with temperature of 40°C, flow rate of 0.300 mL/min, and injection volume was 1.0 μL. All mass spectra were recorded under electrospray ionization in positive mode (ESI+) and chromatograms were recorded with UV detection in range 190-300 nm. *Method A*: The gradient conditions used are: 100% phase A (80% water + 20% MeCN + 0.1% FA) to 100% phase B (MeCN + 0.1% FA) at 3.5 minutes, equilibrated to initial conditions until 4.0 minutes and kept for additional 2.0 minutes. Total time of analysis – 6.0 minutes. *Method B:* The gradient conditions used are: 80% phase A (water + 0.1% FA) and 20% phase B (MeCN + 0.1% FA) to 100% phase B (MeCN + 0.1% FA) at 3.0 minutes, kept till 3.5 minutes, then equilibrated to initial conditions until 4.0 minutes and kept for additional 2.0 minutes. Total time of analysis – 6.0 minutes.

HRMS spectra of representative compounds (presented in Table 1 in manuscript) were measured on a Bruker Daltonics ultrafleXtreme (MALDI-TOF/TOF) apparatus and calibrated to an internal standard.

The synthesis of each intermediate and final compound was performed according to the procedure described in our previous Patent Application **EP 2853532A1** [1].

Summary of experimental section of chemistry:

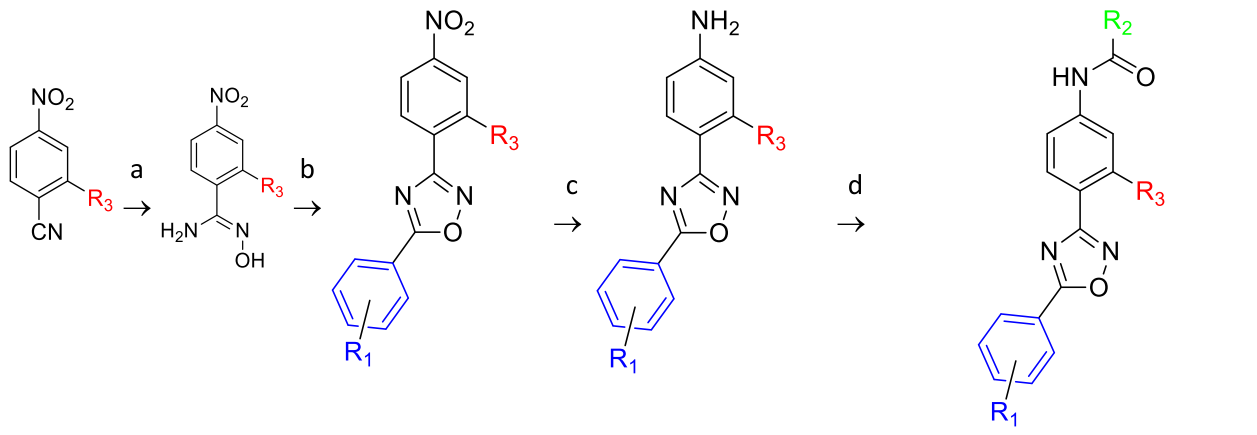

	R3
**14**	H
**14a**	OMe
**14b**	Cl
**14c**	CF_3
**14d**	Me

	R1	R3
**15a**	4-OMe	H
**15b**	2-Cl	H
**15c**	4-F	H
**15d**	H	OMe
**15e**	2-Cl	OMe
**15f**	3-Cl	OMe
**15g**	4-Cl	OMe
**15h**	2-F	OMe
**15i**	2-Cl,4-F	OMe
**15j**	H	Cl
**15k**	2-Cl	Cl
**15l**	H	CF_3
**15m**	2-Cl	CF_3
**15n**	H	Me
**15o**	2-Cl	Me

	R1	R3
**16a**	4-OMe	H
**16b**	2-Cl	H
**16c**	4-F	H
**16d**	H	OMe
**16e**	2-Cl	OMe
**16f**	3-Cl	OMe
**16g**	4-Cl	OMe
**16h**	2-F	OMe
**16i**	2-Cl,4-F	OMe
**16j**	H	Cl
**16k**	2-Cl	Cl
**16l**	H	CF_3
**16m**	2-Cl	CF_3
**16n**	H	Me
**16o**	2-Cl	Me

	R1	R2	R3
**17a**	4-OMe	4-pyridyl	H
**17b**	4-OMe	3-pyridyl	H
**17c**	4-OMe	2-thienyl	H
**17d**	2-Cl	2-pyridyl	H
**17e**	2-Cl	5-Me-1,2-oxazol-3-yl	H
**17f**	4-F	1,2-oxazol-5-yl	H
**17g**	4-F	2-Cl-3-pyridyl	H
**28**	2-Cl	3-pyridyl	H
**29**	2-Cl	4-pyridyl	H
**30**	2-Cl	Ph	H
**31**	2-Cl	6-F-2-pyridyl	H
**32**	2-Cl	6-Cl-2-pyridyl	H
**33**	H	2-pyridyl	OMe
**34**	2-Cl	2-pyridyl	OMe
**35**	3-Cl	2-pyridyl	OMe
**36**	4-Cl	2-pyridyl	OMe
**37**	2-F	2-pyridyl	OMe
**38**	2-Cl,4-F	2-pyridyl	OMe
**39**	2-Cl	6-F-2-pyridyl	OMe
**40**	2-Cl	5-Cl-3-F-2-pyridyl	OMe
**41**	2-Cl	6-Cl-2-pyridyl	OMe
**42**	2-Cl	3-Cl-6-MeO-2-pyridyl	OMe
**43**	2-Cl	4,6-diF-2-pyridyl	OMe
**44**	2-Cl	3-F-2-pyridyl	OMe
**45**	2-Cl	4-pirymidyl	OMe
**46**	2-Cl	thiazoyl	OMe
**47**	2-F	6-F-2-pyridyl	OMe
**48**	2-F	3,6-diCl-2-pyridyl	OMe
**49**	H	2-pyridyl	Cl
**50**	2-Cl	6-F-2-pyridyl	Cl
**51**	2-Cl	6-Cl-2-pyridyl	Cl
**59**	H	2-pyridyl	CF_3
**60**	2-Cl	2-pyridyl	CF_3

**61**	H	2-pyridyl	Me
**62**	2-Cl	2-pyridyl	Me

Scheme 1. Reagents and conditions: (a) $NH_2OH{\cdot}HCl$, $NaOH_{aq}$, EtOH, reflux, 1-5 h; (b) R_1COCl, toluene, K_2CO_3, MW 170°C, 10 min; (c) Fe, CH_3COOH, EtOH, water, 60°C, 1-2 h or $SnCl_2$, 5N HCl, EtOH, reflux, 2 h; (d) R_2COCl, py, rt, overnight.

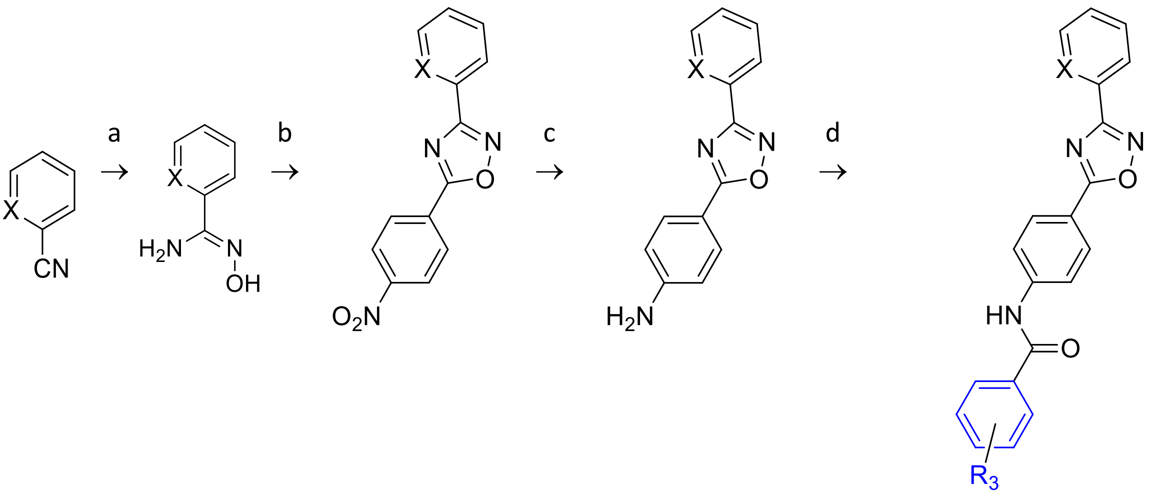

	X
**19a**	C
**19b**	N

	X
**20a**	C
**20b**	N

	X
**21a**	C
**21b**	N

	R3	X
**22a**	H	C
**22b**	4-OMe	C
**22c**	H	N
**22d**	2-Cl	N
**22e**	2-F	N

Scheme 2. Reagents and conditions: (a) $NH_2OH{\cdot}HCl$, $NaOH_{aq}$, EtOH, reflux, 1-5 h; (b) 4-nitrobenzoyl chloride, toluene, K_2CO_3, MW 170°C, 10 min; (c) Fe, CH_3COOH, EtOH, water, 60°C, 1-2 h or Raney Ni, NH_2-NH_2 aq, MeOH/THF, 60°C, 30 min; (d) R_2COCl, py, rt, overnight.

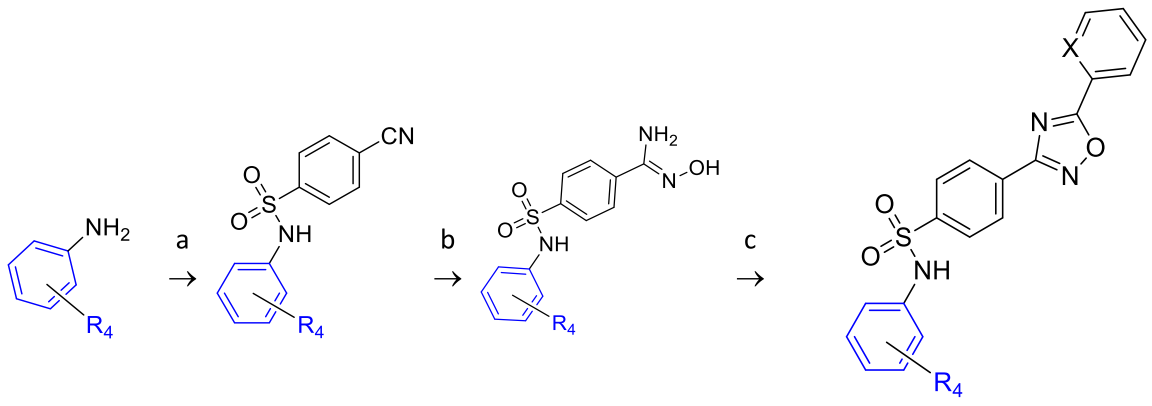

	R_4
23a	2-Cl
23b	2,4-diF

	R_4
25a	2-Cl
25b	2,4-diF

	R_4
26a	2-Cl
26b	2,4-diF

	R_4	X
27a	2-Cl	C
27b	2-Cl	N
27c	2,4-diF	N

Scheme 3. Reagents and conditions: (a) py, rt, overnight; (b) $NH_2OH{\cdot}HCl$, $NaOH_{aq}$, EtOH, reflux, 1–5 h; (c) R_2COCl, toluene, K_2CO_3, MW 170°C, 10 min or rt, 30 min than reflux, 6 h.

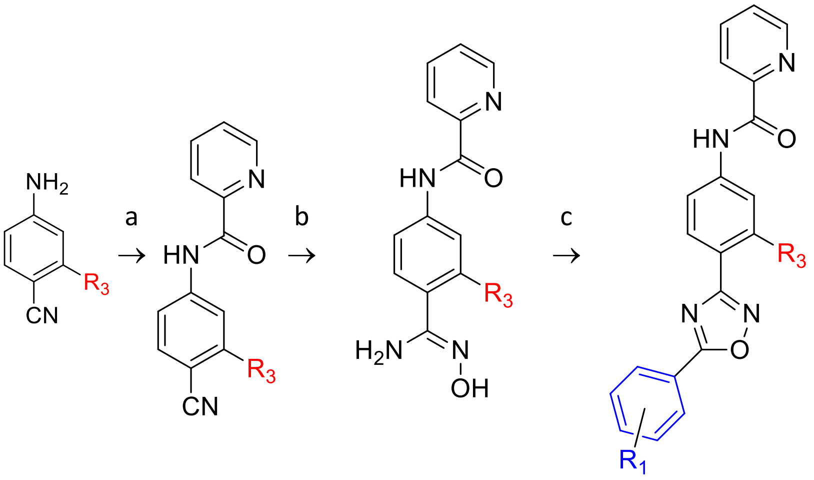

	R_3
25c	Cl
25d	F

	R_3
26c	H
26d	H

	R_1	R_3
52	2-Cl	Cl
53	2-Cl,4-F	Cl
54	2-MeO	Cl
55	H	F
56	2-Cl	F
57	2-Cl,4-F	F
58	2-MeO	F

Scheme 4. Reagents and conditions: (a) (a) py, rt, overnight; (b) $NH_2OH{\cdot}HCl$, $NaOH_{aq}$, EtOH, reflux, 1–5 h; (c) R_1COCl, toluene, K_2CO_3, MW 170°C, 10 min;

Synthetic procedures

***N'*-Hydroxy-4-nitrobenzene-1-carboximidamide (14)**

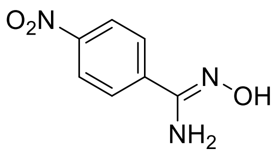

Prepared according to general procedure 1 (for carboximidamide formation) from 4-nitrobenzonitrile (**13**) and method described in the literature [2]. After alkalization, the precipitated solid was triturated and purified by maceration (2-PrOH/hexane 1:3), giving a

white solid product (9.23 g, 75.5%). LC-MS (method A) R_T = 0.65 min (purity: 100%), *m/z* [M+H$^+$] found: 182.2 (calc.181.0). The compound is fully characterized in *New J. Chem.*, **2017**, 41, 9908-9917 [2].

***N'*-Hydroxy-2-methoxy-4-nitrobenzimidamide (14a)**

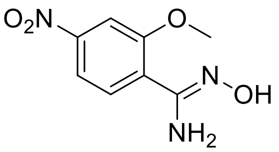

Prepared according to general procedure 1 using 2-methoxy-4-nitrobenzonitrile. The precipitated product was purified by maceration (2-PrOH/hexane 1:2); pale yellow solid, 3.71 g, 62.6%. ^{1}H NMR (300 MHz, $CDCl_3$ + CD_3OD), δ ppm: 7.74-7.26 (m, 3H), 3.88 (s, 3H, CH_3), 3.82 (s br. 3H, NH_2, OH); ^{13}C NMR (75 MHz, $CDCl_3$ + CD_3OD), δ ppm: 157.4, 150.8, 149.1, 130.2, 127.1, 115.6, 106.3, 56.1; LC-MS (method A) R_T = 0.57 min (purity: 100%), *m/z* found: 212.0, calc.: 212.1 [M+H$^+$].

2-Chloro-*N'*-hydroxy-4-nitrobenzimidamide (14b)

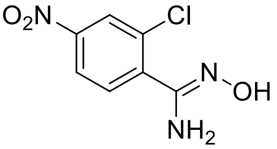

Prepared from 2-chloro-4-nitrobenzonitrile according to general procedure 1. The reaction mixture was diluted with water and acidified with 1N HCl. Precipitated solid was filtered and identified as byproduct 2-chloro-4-nitrobenzamide (1.80 g, 47.0%). The filtrate was extracted with AcOEt, and the water layer was alkalized with $NH_{3(aq)}$. Precipitated product was filtered and purified by maceration (2-PrOH/hexane 1:3); pale yellow solid (0.65 g, 16.0%). ^{1}H NMR (300 MHz, $CDCl_3$), δ ppm: 9.75 (s, 1H, OH), 8.29 (d, *J* = 2.3 Hz, 1H), 8.17 (dd, *J* = 8.5 and 2.3 Hz, 1H), 7.67 (d, *J* = 8.5 Hz, 1H), 6.02 (s br., 2H, NH_2); ^{13}C NMR (75 MHz, $CDCl_3$), δ ppm:

149.8, 148.4, 140.2, 133.8, 132.7, 124.9, 122.4. LC-MS (method A) R_T = 0.81 min (purity: 100%), *m/z* found: 216.1, calc.: 216.0 [M+H$^+$].

***N*'-Hydroxy-4-nitro-2-(trifluoromethyl)benzimidamide** (**14c**)

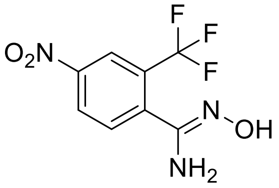

2-trifluoromethylo-4-nitrobenzonitrile (3.50 g, 16.19 mmol, 1 eq) was dissolved in EtOH (80 mL), and hydroxylamine hydrochloride (5.63 g, 80.95 mmol, 5 eq) was added, followed by TEA (4.17 g, 5.74 mL, 97.14 mmol, 6 eq). The reaction mixture was refluxed for 3 h (TLC control), and EtOH was evaporated in vacuo. Water (100 mL) was added to the reaction mixture, and the resulting suspension was extracted with AcOEt. After evaporation of the solvent, the crude product was purified by column chromatography (SiO_2, gradient from $CHCl_3$ to $CHCl_3$/MeOH 49:1) followed by maceration 2-PrOH/hexane (1:2); white solid, 1.72 g, 42.7%. LC-MS (method A) R_T = 1.42 min (purity: 80%), *m/z* found: 250.6, calc.: 250.0 [M+H$^+$]. *The product was used directly to the next step without additional purification.*

***N*'-Hydroxy-2-methyl-4-nitrobenzimidamide** (**14d**)

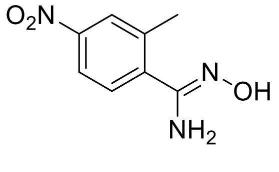

Prepared according to general procedure 1 from 2-methyl-4-nitrobenzonitrile. Reaction gave mixture of two products: 2-metylo-4-nitrobenzamide (byproduct formed after hydrolysis of starting 2-methyl-4-nitrobenzonitrile – 2.10 g), isolated after extraction of acidified water layer and desired **14d** isolated after AcOEt extraction of alkalized water layer. Product was purified by maceration (2-PrOH/hexane 1:2), pale yellow solid (0.68 g, 16.2%). ^{1}H NMR (300 MHz,

DMSO-d_6), δ ppm: 9.64 (s, 1H, OH), 8.10 (d, *J* = 2.4 Hz, 1H), 8.05 (dd, *J* = 8.2 and 2.2 Hz, 1H), 7.53 (d, *J* = 8.4 Hz, 1H), 5.93 (s br. 2H, NH_2), 2.45 (s, 3H, CH_3); ^{13}C NMR (75 MHz, DMSO-d_6), δ ppm: 150.8, 147.1, 140.6, 138.7, 130.2, 124.6, 120.4, 19.8; LC-MS (method B) R_T = 0.59 min (purity: 97.46%), *m/z* found: 196.5, calc.:196.1 [$M+H^+$].

5-(4-Methoxyphenyl)-3-(4-nitrophenyl)-1,2,4-oxadiazole (15a)

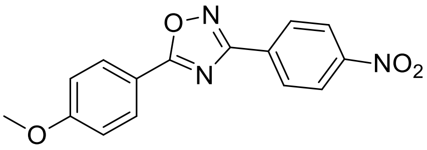

Prepared according to general procedure 2 from carboximidamide (**14**) and 4-methoxybenzoyl chloride. Product was purified by maceration (EtOH/water 1:1); pale yellow solid, 3.74 g, 57.0%. ^1H NMR (300 MHz, DMSO-d_6), δ ppm: 8.38 (d, *J* = 9.0 Hz, 2H), 8.30 (d, *J* = 9.0 Hz, 2H), 8.12 (d, *J* = 9.0 Hz, 2H), 7.18 (d, *J* = 9.0 Hz, 2H), 3.89 (s, 3H, CH_3); ^{13}C NMR (75 MHz, DMSO-d_6), δ ppm: 176.5, 167.4, 164.0, 149.8, 132.7, 130.5 (2C), 128.9 (2C), 124.7 (2C), 116.0, 115.6 (2C), 56.2. *Due to poor solubility LC-MS analysis was not performed.*

5-(2-Chlorophenyl)-3-(4-nitrophenyl)-1,2,4-oxadiazole (**15b**)

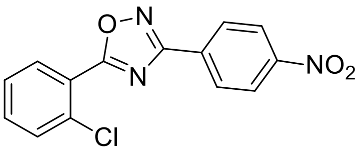

Prepared according to general procedure 2 from carboximidamide (**14**) and 2-chlorobenzoyl chloride. Crude product was purified by maceration (2-PrOH/hexane 1:3); pale yellow solid, 2.48 g, 74.5%. LC-MS (method B) R_T = 3.84 min (purity 88.6%), m/z found: 302.0, calc.:301.0 [M+H+]. *The product was used directly to the next step without additional purification.*

5-(4-Fluorophenyl)-3-(4-nitrophenyl)-1,2,4-oxadiazole (**15c**)

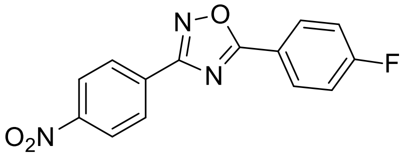

Prepared according to general procedure 2 from **14** and 4-fluorobenzoyl chloride. Product was purified by maceration (2-PrOH); pale yellow solid, 4.32 g, 83.1%. ^{1}H NMR (300 MHz, DMSO-d$_6$), δ ppm: 8.44-8.22 (m, 6H), 7.51-7.45 (m, 2H); ^{13}C NMR (75 MHz, DMSO-d$_6$), δ ppm: 175.7, 167.5, 165.6 (d, J = 252.9 Hz), 149.8, 132.4, 131.4 (d, J = 9.6 Hz, 2C), 129.0 (2C), 124.8 (2C), 120.3 (d, J = 3.2 Hz), 117.3 (d, J = 22.6 Hz, 2C). *Due to poor solubility, LC-MS analysis was not performed.*

3-(2-Methoxy-4-nitrophenyl)-5-phenyl-1,2,4-oxadiazole (**15d)**

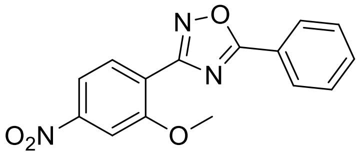

Prepared from **14a** and benzoyl chloride according to general procedure 2. Product was purified by maceration (2-PrOH/hexane 1:2); yellow solid, 1.71 g, 86.8%. ^{1}H NMR (300 MHz, CDCl$_3$), δ ppm: 8.25-8.14 (m, 3H), 8.03-7.97 (m, 2H), 7.78-7.71 (m, 1H), 7.70-7.62 (m, 2H), 4.05 (s, 3H);^{13}C NMR (75 MHz, CDCl$_3$), δ ppm: 175.0, 166.0, 158.7, 150.5, 133.9, 132.4, 130.0 (2C), 128.4 (2C), 123.6, 121.6, 115.9, 107.6, 57.2; LC-MS (method A) R_T = 3.49 min (purity: 84.90%), *m/z* found: 298.88, calc.: 298.1 [M+H$^+$]. *The product was used directly to next step without additional purification.*

5-(2-Chlorophenyl)-3-(2-methoxy-4-nitrophenyl)-1,2,4-oxadiazole (**15e**)

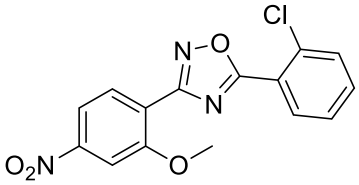

Prepared from **14a** and 2-chlorobenzoyl chloride according to general procedure 2. Product was purified by maceration (2-PrOH/hexane 1:2); yellow solid, 1.90 g, 88.0%. ^{1}H NMR (300 MHz, DMSO-d_6), δ ppm: 8.23-8.18 (m, 1H), 8.16 (dd, *J* = 7.8, 1.5 Hz, 1H), 8.00-7.97 (m, 2H), 7.77-7.68 (m, 2H), 7.60 (dt, *J* = 7.7, 1.7 Hz, 1H), 4.04 (s, 3H); ^{13}C NMR (75 MHz, DMSO-d_6) δ ppm: 173.4, 165.8, 158.7, 150.6, 134.8, 132.9, 132.7, 132.5, 131.9, 128.5, 122.9, 121.3, 116.0, 107.8, 57.3; LC-MS (method A) R_T = 3.57 min (purity: 96.93%), *m/z* found: 331.8, calc.: 332.0 [M+H$^+$].

5-(3-Chlorophenyl)-3-(2-methoxy-4-nitrophenyl)-1,2,4-oxadiazole (15f)

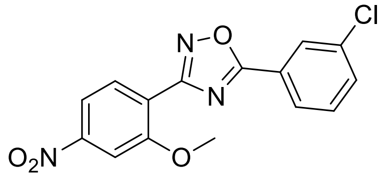

Prepared from **14a** and 3-chlorobenzoyl chloride according to general procedure 2. Product was purified by maceration (2-PrOH/hexane 1:2); yellow solid, 0.79 g, 84.0%. ^{1}H NMR (300 MHz, DMSO-d_6), δ ppm: 8.24 (dd, *J* = 8.2, 0.4 Hz, 1H), 8.21-8.12 (m, 1H), 8.16-8.13 (m 1H), 8.02-7.99 (m, 2H), 7.82 (ddd, *J* = 8.1, 2.2, 1.0 Hz,1H), 7.71 (t, *J* = 7.9 Hz, 1H), 4.06 (s, 3H); 13C NMR (75 MHz, DMSO-d6), δ ppm: 173.5, 165.7, 158.3, 150.2, 134.2, 133.2, 132.1, 131.7, 127.5, 126.7, 125.1, 120.9, 115.5, 107.3, 56.9; LC-MS (method A) R_T = 3.84 min (purity: 100%), *m/z* found: 332.1, calc.: 332.0 [M+H$^+$].

5-(4-Chlorophenyl)-3-(2-methoxy-4-nitrophenyl)-1,2,4-oxadiazole (15g)

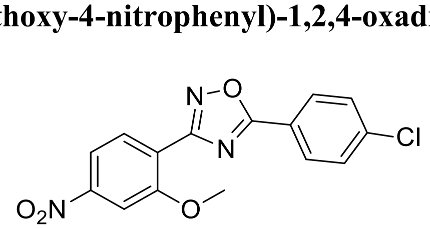

Prepared from **14a** and 4-chlorobenzoyl chloride according to general procedure 2. Product was purified by maceration (2-PrOH/hexane 1:2); yellow solid, 0.55 g, 58.5%. ^{1}H NMR (300 MHz,

DMSO-d_6), δ ppm: 8.24-8.19 (m, 3H), 8.03-7.99 (m, 2H), 7.77-7.73 (m, 2H), 4.06 (s, 3H); ^{13}C NMR (75 MHz, DMSO-d_6), δ ppm: 173.8, 165.7, 158.3, 150.2, 138.4, 132.1, 129.9 (4C), 122.1, 121.0, 115.5, 107.3, 56.9; LC-MS (method A) R_T = 3.83 min (purity: 98.89%), *m/z* found: 332.4, calc.: 332.0 [M+H$^+$].

5-(2-Fluorophenyl)-3-(2-methoxy-4-nitrophenyl)-1,2,4-oxadiazole (15h)

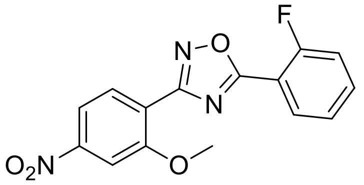

Prepared from **14a** and 2-fluorobenzoyl chloride according to general procedure 2. The product was purified by maceration (2-PrOH/hexane 1:2); pale yellow solid, 1.81 g, 87.9%. ^{1}H NMR (300 MHz, DMSO-d_6), δ ppm: 8.22-8.15 (m, 2H), 7.99-7.95 (m, 2H), 7.82-7.73 (m, 1H), 7.55-7.45 (m, 2H), 4.04 (s, 3H); ^{13}C NMR (75 MHz, DMSO-d_6), δ ppm: 172.2 (d, *J* = 4.3 Hz), 165.8, 160.4 (d, *J* = 257.9 Hz), 158.8, 150.7, 136.2 (d, *J* = 8.8 Hz), 132.5, 131.3, 125.9 (d, *J* = 3.6 Hz), 121.6, 117.7 (d, *J* = 20.5 Hz), 115.9, 112.1 (d, *J* = 11.3 Hz), 107.8, 57.3; LC-MS (method A) R_T = 3.43 min (purity: 98.70%), *m/z* found: 316.4, calc. 316.1 [M+H$^+$].

5-(2-Chloro-4-fluorophenyl)-3-(2-methoxy-4-nitrophenyl)-1,2,4-oxadiazole(15i)

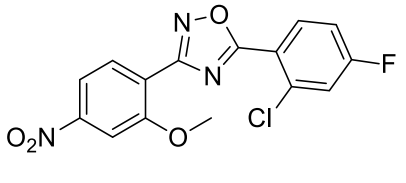

Prepared from **14a** and 2-chloro-4-fluorobenzoyl chloride according to general procedure 2. Product was purified by maceration (2-PrOH/hexane 1:2); pale yellow solid, 2.10 g, 90.9%. ^{1}H NMR (300 MHz, DMSO-d_6), δ ppm: 8.27-8.18 (m, 2H), 7.98 (dd, *J* = 7.0, 2.1 Hz 2H), 7.80 (dd, *J* = 8.9, 2.5 Hz, 1H), 7.51 (ddd, *J* = 8.8, 8.1, 2.6 Hz, 1H), 4.05 (s, 3H); ^{13}C NMR (75 MHz, DMSO-d_6), δ ppm: 173.0, 165.7, 164.6 (d, *J* = 255.5 Hz), 158.7, 150.6, 134.8 (d, *J* =10.2 Hz),

134.6 (d, J = 11.5 Hz), 132.5, 121.2, 119.8 (d, J = 3.5 Hz), 119.4 (d, J = 25.8 Hz), 116.2 (d, J = 22.1 Hz), 116.0, 107.8, 57.3; LC-MS (method A) R_T = 3.66 min (purity: 100%), *m/z* found: 350.4, calc.: 350.0 [M+H$^+$].

3-(2-Chloro-4-nitrophenyl)-5-phenyl-1,2,4-oxadiazole (15j)

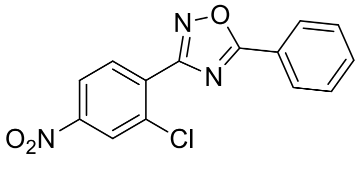

Prepared from **14b** and benzoyl chloride according to general procedure 2. Product was purified by maceration (2-PrOH/hexane 1:2); pale yellow solid, 0.60 g, 85.7%. ^{1}H NMR (300 MHz, DMSO-d$_6$), δ ppm: 8.44 (d, J = 2.0 Hz, 1H), 8.31-8.21 (m, 4H), 7.68-7.55 (m, 3H); ^{13}C NMR (75 MHz, DMSO-d$_6$), δ ppm: 175.9, 166.4, 149.1, 134.9, 133.3, 132.7, 132.1, 129.3 (2C), 128.3 (2C), 126.1, 123.6, 121.7; LC-MS (method B) R_T = 3.77 min (purity: 96.40%), *m/z* found: 302.4, calc.: 302.0 [M+H$^+$].

3-(2-Chloro-4-nitrophenyl)-5-(2-chlorophenyl)-1,2,4-oxadiazole (15k)

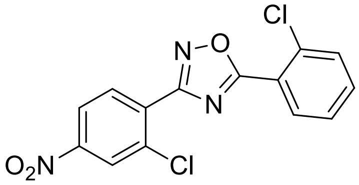

Prepared according to general procedure from **14b** and 2-chlorobenzoyl chloride. Product was purified by maceration (2-PrOH/hexane 1:2), 0.60 g, 85.7%. ^{1}H NMR (300 MHz, CDCl$_3$), δ ppm: 8.45 (d, J = 2.1 Hz, 1H), 8.33-8.24 (m, 2H), 8.18 (dd, J = 7.8, 1.7 Hz, 1H), 7.64-7.57 (m, 2H), 7.52-7.43 (m, 1H); ^{13}C NMR (75 MHz, CDCl$_3$), δ ppm: 174.4, 166.2, 149.2, 134.9, 134.1, 133.6, 132.8, 132.0, 131.8, 131.7, 127.2, 126.1, 122.9, 121.7; LC-MS (method A) R_T = 3.87 min (purity: 91.57%), *m/z* found: 336.7, calc.: 336.0 [M+H$^+$]. *The product was used directly to next step without additional purification.*

3-(4-Nitro-2-(trifluoromethyl)phenyl)-5-phenyl-1,2,4-oxadiazole (15l)

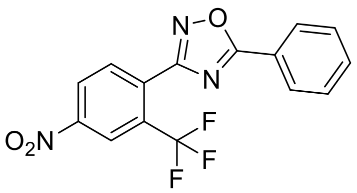

Prepared from **14c** and benzoyl chloride according to general procedure 2. Product was purified by maceration (2-PrOH/hexane 1:2); pale yellow solid, 0.73 g, 67.6%. LC-MS (method B) R_T = 3.76 min (purity: 92.07%), *m/z* found: 336.6, calc.: 336.0 [M+H$^+$]. *The product was used directly to the next step without additional purification.*

5-(2-Chlorophenyl)-3-(4-nitro-2-(trifluoromethyl)phenyl)-1,2,4-oxadiazole (15m)

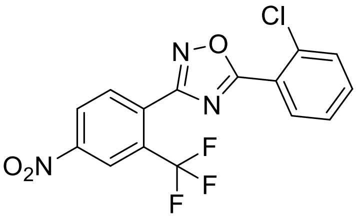

Prepared from **14c** and 20chlorobenzoyl chloride according to general procedure 2. Product was purified by column chromatography (SiO_2, $CHCl_3$/hexane 2:1) followed by maceration (2-PrOH/hexane 1:2); pale yellow solid, 0.98 g, 40.2%. ^{1}H NMR (300 MHz, DMSO-d_6), δ ppm: 8.71 (dd, *J*= 8.5, 2.3 Hz, 1H), 8.66 (d, *J*= 2.3 Hz, 1H), 8.30 (d, *J*= 8.5 Hz, 1H), 8.21-8.16 (m, 1H), 7.80-7.71 (m, 2H), 7.66-7.59 (m, 1H); ^{13}C NMR (75 MHz, DMSO-d_6), δ ppm: 174.6, 166.5, 149.5, 135.2, 134.6, 132.9, 137,7, 132.0, 130.6 (q, *J* = 1.8 Hz), 129.5 (q, *J* = 33.2),, 128.6, 128.4, 122.9 (q, *J* = 5.7 Hz), 122.7 (q, *J* = 273.9), 122.4; LC-MS (method A) R_T = 3.81 min (purity: 100%), *m/z* found: 370.4, calc.: 370.0 [M+H$^+$].

3-(2-Methyl-4-nitrophenyl)-5-phenyl-1,2,4-oxadiazole (15n)

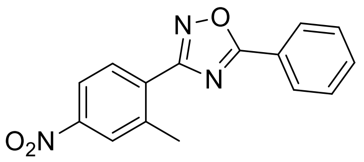

Prepared from **14d** and benzoyl chloride according to general procedure 2. The product was purified by column chromatography (SiO_2, $CHCl_3$) followed by maceration (2-PrOH/hexane 1:2); white solid, 0.60 g, 52.2%. LC-MS (method B) R_T = 3.95 min (purity: 58.30%), *m/z* found: 282.7, calc.: 282.1 [M+H$^+$]. *The product was used directly to the next step without additional purification due to poor solubility and difficulty with purification.*

5-(2-Chlorophenyl)-3-(2-methyl-4-nitrophenyl)-1,2,4-oxadiazole (**15o**)

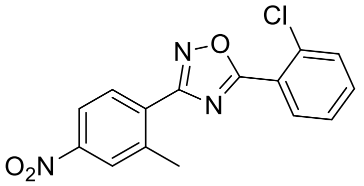

Prepared from **14d** and 2-chlorobenzoyl chloride according to general procedure 2. Product was purified by maceration (2-PrOH/hexane 1:2); white solid, 0.83 g, 79.8%. ^{1}H NMR (300 MHz, DMSO-d$_6$), δ ppm: 8.29-8.22 (m, 3H), 8.18 (dd, *J* = 7.6, 1.6 Hz, 1H), 7.77-7.68 (m, 2H), 7.63-7.58 (m, 1H), 2.72 (s, 3H); ^{13}C NMR (75 MHz, DMSO-d$_6$), δ ppm: 173.9, 167.9, 149.0, 140.6, 134.9, 132.9, 132.7, 131.9, 131.8, 131.7, 128.5, 126.4, 122.8, 121.7, 22.0; LC-MS (method A) R_T = 3.28 min (purity: 60.8%), *m/z* found: 315.8, calc.: 316.0 [M+H$^+$].

4-(5-(4-Methoxyphenyl)-1,2,4-oxadiazol-3-yl)aniline (**16a**)

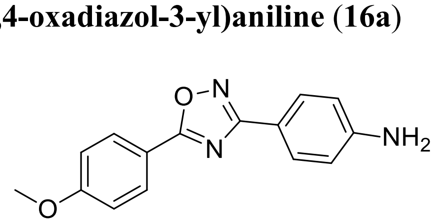

Prepared according to general procedure 3, method A from **15a**; white solid, 1.88 g, 65.3%. ^{1}H NMR (300 MHz, $CDCl_3$), δ ppm: 8.14 (d, *J* = 9.0, 2H), 7.96 (d, *J* = 8.7, 2H), 7.02 (d, *J* = 9.0,

2H), 6.75 (d, J = 8.7, 2H), 3.95 (s br. 2H, NH_2), 3.89 (s, 3H, CH_3); ^{13}C NMR (75 MHz, $CDCl_3$), δ ppm: 175.0, 168.7, 163.0, 149.0, 130.0 (2C), 129.0 (2C), 117.1, 117.0, 114.7 (2C), 114.4 (2C), 55.5; LC-MS (method B) R_T= 3.07 min (purity: 100%), *m/z* found: 268.4, calc. 268.1 [$M+H^+$].

4-(5-(2-Chlorophenyl)-1,2,4-oxadiazol-3-yl)aniline (16b)

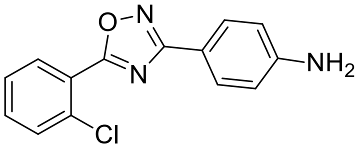

Prepared according to general procedure 3, method B from **15b**. Crude product was purified by column chromatography (SiO_2, $CHCl_3$/MeOH 99:1) followed by maceration (2-PrOH/hexane 1:3); white solid, 0.98 g, 54.1%. ^1H NMR (300 MHz, $CDCl_3$), δ ppm: 8.12 (dd, J = 7.6 and 1.9 Hz, 1H), 8.00-7.95 (m, 2H), 7.57 (dd, J = 7.8 and 1.4, 1H), 7.49 (td, J = 7.3 and 1.8 Hz, 1H), 7.41 (td, J = 7.7 and 1.6 Hz, 1H), 6.78-6.73 (m, 2H), 3.98 (s br. 2H, NH_2); ^{13}C NMR (75 MHz, $CDCl_3$), δ ppm: 173.7, 168.6, 149.3, 133.8, 132.9, 131.9, 131.4, 129.0 (2C), 127.1, 123.9, 116.5, 114.7 (2C); LC-MS (method B) R_T = 3.21 min (purity: 96.77%), *m/z* found: 272.3, calc.: 272.0 [$M+H^+$].

4-(5-(4-Fluorophenyl)-1,2,4-oxadiazol-3-yl)aniline (16c)

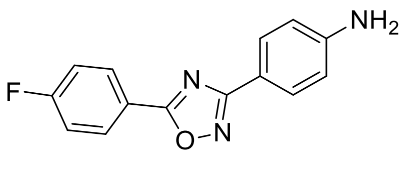

Prepared according to general procedure 3, method A from **15c**; precipitated solid was purified by maceration (2-PrOH); white solid, 2.22 g, 62.0%. ^1H NMR (300 MHz, $CDCl_3$), δ ppm: 8.24-8.18 (m, 2H, ArH), 7.98-7.93 (m, 2H, ArH), 7.26-7.19 (m, 2H, ArH), 6.78-6.73 (m, 2H, ArH), 3.98 (s br. 2H, NH_2); ^{13}C NMR (75 MHz, $CDCl_3$), δ ppm: 174.2, 168.9, 165.3 (d, J = 254.1 Hz), 149.2, 130.5 (d, J = 9.1 Hz, 2C), 129.0 (2C), 120.9 (d, J = 3.1 Hz), 116.6, 116.4 (d, J =

22.3 Hz, 2C), 114.7 (2C); LC-MS (method B) R_T = 3.13 min (purity: 99.35%), *m/z* found: 256.95, calc.: 256.1 [$M+H^+$].

3-Methoxy-4-(5-phenyl-1,2,4-oxadiazol-3-yl)aniline (16d)

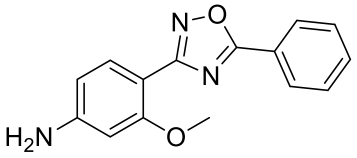

Prepared from **15d** according to general procedure 3, method B. Purification of final product was performed by maceration (2-PrOH/hexane 1:2); white solid, 1.22g, 79.7%. ^{1}H NMR (300 MHz, $CDCl_3$), δ ppm: 8.27-8.13 (m, 2H), 7.97 (d, *J* = 8.3 Hz, 1H), 7.65-7.44 (m, 3H), 6.38 (dd, *J* = 8.3, 2.1 Hz, 1H), 6.32 (d, *J* = 2.1 Hz, 1H), 4.02 (s br. 2H), 3.94 (s, 3H); ^{13}C NMR (75 MHz, $CDCl_3$), δ ppm: 173.9, 167.4, 159.7, 150.5, 132.7, 132.3, 128.9 (2C), 128.1 (2C), 124.6, 107.1, 106.0, 98.0, 55.8; LC-MS (method A) R_T = 2.77 min (purity: 97.85%), *m/z* found: 268.05, calc.: 268.1 [$M+H^+$].

4-(5-(2-Chlorophenyl)-1,2,4-oxadiazol-3-yl)-3-methoxyaniline (16e)

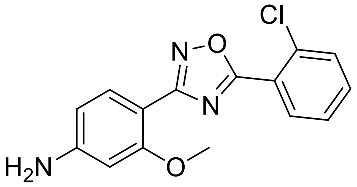

Prepared from **15e** according to general procedure 3, method B. Purification of crude product was performed by maceration (2-PrOH/hexane 1:2); white solid, 1.33 g, 81.6%. ^{1}H NMR (300 MHz, DMSO-d_6), δ ppm: 8.10 (dd, *J* = 7.7, 1.7 Hz, 1H), 7.78-7.64 (m, 3H), 7.64-7.52 (m, 1H), 6.36 (d, *J* = 1.9 Hz, 1H), 6.30 (dd, *J* = 8.4, 2.0 Hz, 1H), 5.84 (s br. 2H), 3.81 (s, 3H); ^{13}C NMR (75 MHz, DMSO-d_6), δ ppm: 171.5, 166.6, 159.3, 153.3, 133.7, 132.1, 131.9, 131.8, 131.1, 127.8, 123.2, 106.0, 101.7, 96.6, 55.2; LC-MS (method A) R_T = 2.91 min (purity: 100%), *m/z* found: 301.6, calc.: 302.1 [$M+H^+$].

4-(5-(3-Chlorophenyl)-1,2,4-oxadiazol-3-yl)-3-methoxyaniline (**16f**)

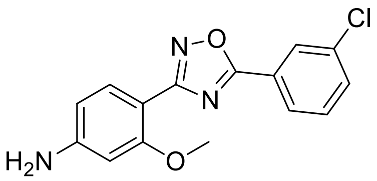

Prepared from **15f** according to general procedure 3, method B. Purification of crude product was performed by maceration (2-PrOH/hexane 1:3); white solid, 0.56 g, 82.3%. ^{1}H NMR (300 MHz, $CDCl_3$), δ ppm: 8.20 (t, J = 1.7 Hz, 1H), 8.11-8.04 (m, 1H), 7.96 (d, J = 8.4 Hz, 1H), 7.55 (ddd, J = 8.1, 2.1, 1.1 Hz, 1H), 7.52-7.44 (m, 1H), 6.40 (dd, J = 8.4, 2.1 Hz, 1H), 6.33 (d, J = 2.1 Hz, 1H), 4.03 (s br, 2H), 3.96 (s, 3H); ^{13}C NMR (75 MHz, $CDCl_3$), δ ppm: 172.7, 167.6, 159.7, 150.7, 135.2, 132.7, 132.4, 130.4, 128.2, 126.3, 126.2, 107.2, 105.8, 98.0, 55.9; LC-MS (method B) R_T = 3.23 min (purity: 98.19%), *m/z* found: 302.2, calc. 302.1 [M+H$^+$].

4-(5-(4-Chlorophenyl)-1,2,4-oxadiazol-3-yl)-3-methoxyaniline (**16g**)

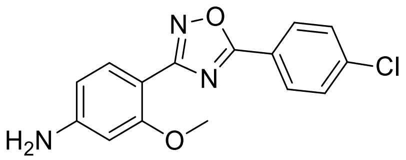

Prepared from **15g** according to general procedure 3, method B. Purification of crude product was performed by column chromatography (SiO_2, $CHCl_3$) and maceration (2-PrOH/hexane 1:3); white solid, 0.36 g, 76.6%. ^{1}H NMR (300 MHz, $CDCl_3$), δ ppm: 8.20-8.09 (m, 2H), 7.95 (d, J = 8.4 Hz, 1H), 7.60-7.46 (m, 2H), 6.39 (dd, J = 8.4, 2.1 Hz, 1H), 6.33 (d, J = 2.1 Hz, 1H), 4.03 (s br, 2H), 3.94 (s, 3H); 13C NMR (75 MHz, $CDCl_3$), δ ppm: 173.0, 167.5, 159.7, 150.7, 138.8, 132.7, 129.5 (2C), 129.4 (2C), 123.1, 107.1, 105.8, 98.0, 55.8; LC-MS (method B) R_T = 3.24 min (purity: 97.20%), *m/z* found: 302.1, calc.: 302.1 [M+H$^+$].

4-(5-(2-Fluorophenyl)-1,2,4-oxadiazol-3-yl)-3-methoxyaniline (**16h**)

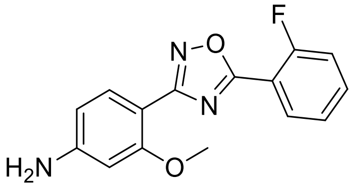

Prepared according to general procedure 3, method B from **15h**. Purification of product was performed by maceration (2-PrOH/hexane 1:2); white solid, 1.36 g, 88.3%. ^{1}H NMR (300 MHz, DMSO-d$_6$), δ ppm: 8.16 (dt, *J* = 7.6, 1.8 Hz, 1H), 7.81-7.64 (m, 2H), 7.57-7.40 (m, 2H), 6.36 (d, *J* = 1.9 Hz, 1H), 6.30 (dd, *J* = 8.4, 2.0 Hz, 1H), 5.83 (s br. 2H), 3.81 (s, 3H); ^{13}C NMR (75 MHz, DMSO-d$_6$), δ ppm: 169.9 (d, *J* = 4.2 Hz), 166.6, 159.3, 159.8 (d, *J* = 257.1 Hz), 153.3, 135.0 (d, *J* = 8.7 Hz), 131.8, 130.7, 125.3 (d, *J* = 3.6 Hz), 117.1 (d, *J* = 20.8 Hz), 112.1 (d, *J* = 11.5 Hz), 106.0, 101.7, 96.6, 55.2; LC-MS (method A) R_T = 2.71 min (purity: 100%), *m/z* found: 286.04, calc.: 286.1 [M+H$^+$].

4-(5-(2-Chloro-4-fluorophenyl)-1,2,4-oxadiazol-3-yl)-3-methoxyaniline (16i)

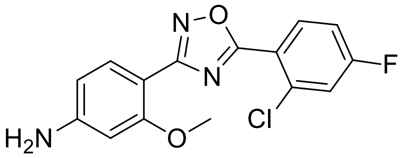

Prepared from **15i** according to general procedure 3, method B. Crude product was purified by maceration (2-PrOH/hexane 1:2); white solid, 1.88 g, 97.9%. ^{1}H NMR (300 MHz, DMSO-d$_6$), δ ppm: 8.15 (dd, *J* = 8.8, 6.0 Hz, 1H), 7.96 (d, *J* = 8.3 Hz, 1H), 7.31 (dd, *J* = 8.5, 2.5 Hz, 1H), 7.14 (ddd, *J* = 8.8, 7.6, 2.5 Hz, 1H), 6.38 (dd, *J* = 8.4, 2.1 Hz, 1H), 6.33 (d, *J* = 2.1 Hz, 1H), 4.01 (s br. 2H), 3.96 (s, 3H) ^{13}C NMR (75 MHz, DMSO-d$_6$), δ ppm: 171.3, 167.1, 164.2 (d, *J* = 254.6 Hz), 159.8, 153.8, 134.4 (d, *J* = 10.1Hz), 134.3 (d, *J* = 11.3 Hz), 132.3, 120.5 (d, *J* = 3.5 Hz), 119.2 (d, *J* = 25.7 Hz), 116.0 (d, *J* = 22.0 Hz), 106.5, 102.2, 97.1, 55.7; LC-MS (method A) R_T = 3.05 min (purity: 99.60%), *m/z* found: 320.4, calc.: 320.0 [M+H$^+$].

3-Chloro-4-(5-phenyl-1,2,4-oxadiazol-3-yl)aniline (16j)

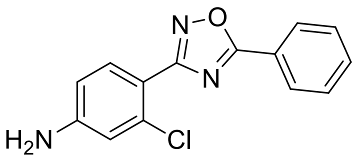

Prepared from **15j** according to general procedure 3, method B. Purification of product was performed by column chromatography (SiO_2, $CHCl_3$/MeOH 99:1) followed by maceration (2-PrOH/hexane 1:2); white solid, 0.28g, 56.0%. ^{1}H NMR (300 MHz, $CDCl_3$), δ ppm: 8.23-8.17 (m, 2H), 7.87 (d, *J* = 8.5 Hz, 1H), 7.68-7.45 (m, 3H), 6.82 (d, *J* = 2.3 Hz, 1H), 6.65 (dd, *J* = 8.5, 2.3 Hz,1H), 4.03 (s br., 2H); ^{13}C NMR (75 MHz, $CDCl_3$), δ ppm: 174.6, 167.8, 149.4, 134.4, 132.9, 132.6, 129.0 (2C), 128.1 (2C), 124.3, 116.3, 115.5, 113.1; LC-MS (method B) R_T = 3.26 min (purity 91.1%), *m/z* found: 271.8, calc.: 271.7 [M+H$^+$].

3-Chloro-4-(5-(2-chlorophenyl)-1,2,4-oxadiazol-3-yl)aniline (16k)

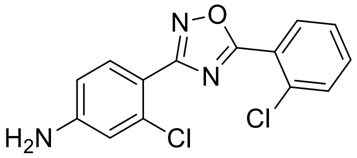

Prepared from **15k** according to general procedure 3, method B. Product was purified by maceration (2-PrOH/hexane 1:2); white solid, 0.38 g, 69.6%. ^{1}H NMR (300 MHz, $CDCl_3$), δ ppm: δ 8.13 (dd, *J* = 7.7, 1.8 Hz, 1H), 7.89 (d, *J* = 8.5 Hz, 1H), 7.58 (dd, *J* = 8.0, 1.5 Hz, 1H), 7.50 (dt, *J* = 7.7, 1.8 Hz, 1H), 7.42 (dt, *J* = 7.5, 1.5 Hz, 1H), 6.82 (d, *J* = 2.3 Hz, 1H), 6.65 (dd, *J* = 8.5, 2.3 Hz 1H), 4.03 (s br. 2H); ^{13}C NMR (75 MHz, $CDCl_3$), δ ppm: 173.1, 167.5, 149.5, 134.5, 133.9, 133.0, 132.9, 131.9, 131.4, 127.0, 123.7, 116.3, 115.3, 113.1; LC-MS (method A) R_T = 3.32 min (purity: 93.49%), *m/z* found: 306.3, calc.: 306.0 [M+H$^+$].

4-(5-Phenyl-1,2,4-oxadiazol-3-yl)-3-(trifluoromethyl)aniline (16l)

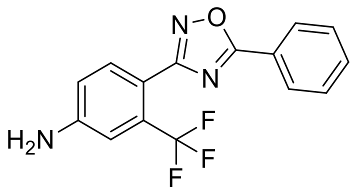

Prepared according to general procedure 3, method B from **15l**. Purification of product was performed by maceration (2-PrOH/hexane 1:2); white solid, 0.45 g, 67.4%. ^{1}H NMR (300 MHz, $CDCl_3$), δ ppm: 8.24-8.14(m, 2H), 7.70 (d, *J* = 8.4 Hz, 1H), 7.64-7.48 (m, 3H), 7.09 (d, *J* = 2.4 Hz, 1H), 6.92-6.83 (m, 1H), 4.13 (s br. 2H); ^{13}C NMR (75 MHz, $CDCl_3$), δ ppm: 175.1, 168.3, 148.5, 133.3, 132.7, 130.5 (q, *J* = 31.7 Hz), 129.1 (2C), 128.1 (2C), 124.2, 123.4 (q, *J* = 273.9 Hz), 116.8, 114.4 (q, *J* = 1.8 Hz), 112.8 (*J* = 5.6 Hz); LC-MS (method B) R_T = 3.31 min (purity: 94.9%), *m/z* found: 306.6, calc. 306.1 [$M+H^+$].

4-(5-(2-Chlorophenyl)-1,2,4-oxadiazol-3-yl)-3-(trifluoromethyl)aniline (16m)

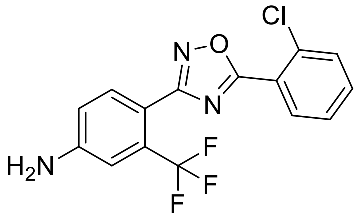

Prepared according to general procedure 3, method B from **15m**. Purification of the final product was performed by maceration (2-PrOH/hexane 1:2); white solid, 0.61 g, 75.3%. ^{1}H NMR (300 MHz, $CDCl_3$), δ ppm: 8.14 (dd, *J* = 7.8, 1.7 Hz, 1H), 7.73 (d, *J* = 8.4 Hz, 1H), 7.57 (dd, *J* = 7.9, 1.4 Hz, 1H), 7.50 (td, *J* = 7.2, 1.8 Hz, 1H), 7.43 (td, *J* = 7.6, 1.5 Hz, 1H), 7.08 (d, *J* = 2.4 Hz, 1H), 6.87 (dd, *J* = 8.5, 2.4 Hz, 1H), 4.14 (s br. 2H); ^{13}C NMR (75 MHz, $CDCl_3$), δ ppm: 173.7, 167.9, 148.6, 133.9, 133.4, 133.1, 131.9, 131.4, 130.5 (q, *J* = 31.9 Hz), 127.1, 123.5 123.4 (q, *J* = 273.8 Hz), 116.7, 114.1 (q, *J* = 1.9 Hz), 112.9 (q, *J* = 5.7 Hz); LC-MS (method A) R_T = 3.42 min (purity 96.9%), *m/z* found: 340.5, calc.: 340.0 [$M+H^+$].

3-Methyl-4-(5-phenyl-1,2,4-oxadiazol-3-yl)aniline (16n)

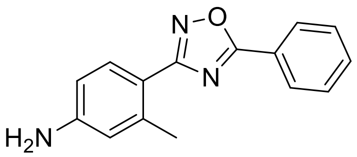

Prepared according to general procedure 3, method B from **15n**. Purification was performed by maceration (2-PrOH/hexane 1:2); white solid, 0.35 g, 66.0%. ^{1}H NMR (300 MHz, $CDCl_3$), δ ppm: 8.25-8.17 (m, 2H), 8.00-7.93 (m, 1H), 7.63-7.49 (m, 3H), 6.62 (dd, *J* = 8.2, 1.3 Hz, 2H), 3.89 (s br. 2H), 2.62 (s, 3H); ^{13}C NMR (75 MHz, $CDCl_3$), δ ppm: 174.0, 169.5, 148.5, 140.0, 132.4, 131.8, 129.0 (2C), 128.1 (2C), 124.6, 117.2, 116.2, 112.2, 22.6; LC-MS (method B) R_T = 3.25 min (purity: 97.9%), *m/z* found: 252.6, calc.: 252.1 [M+H$^+$].

4-(5-(2-Chlorophenyl)-1,2,4-oxadiazol-3-yl)-3-methylaniline (16o)

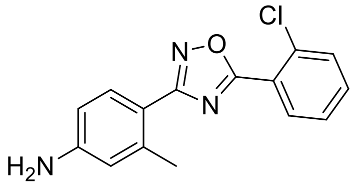

Prepared according to general procedure 3, method B from **15o**. Crude product was purified by maceration (2-PrOH/hexane 1:2); white solid, 0.55 g, 76.4%. ^{1}H NMR (300 MHz, $CDCl_3$), δ ppm: 8.14 (dd, *J* = 7.7, 1.7 Hz, 1H), 8.02-7.90 (m, 1H), 7.61-7.54(m, 1H), 7.49 (dt, *J* = 7.7, 1.9 Hz, 1H), 7.42 (dt, *J* = 7.5, 1.5 Hz, 1H), 6.66-6.57 (m, 2H), 3.89 (s br. 2H), 2.63 (s, 3H); ^{13}C NMR (75 MHz, $CDCl_3$), δ ppm: 172.6, 169.2, 148.6, 140.1, 133.8, 132.8, 131.9, 131.8, 131.4, 127.0, 124.0, 117.2, 116.0, 112.3, 22.6; LC-MS (method A) R_T = 3.37 min (purity: 100%), *m/z* found: 286.04, calc.: 286.1 [M+H$^+$].

***N*-(4-(5-(4-Methoxyphenyl)-1,2,4-oxadiazol-3-yl)phenyl)isonicotinamide** (**17a**)

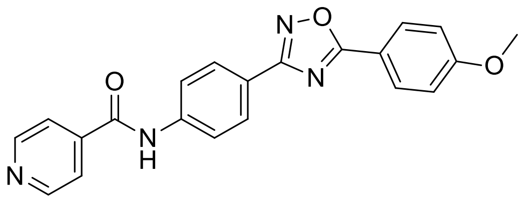

Prepared from **16a** and isonicotinoyl chloride, hydrochloride according to general procedure 4. Crude product was purified by maceration (MeOH); white solid, 0.26 g, 92.9%.

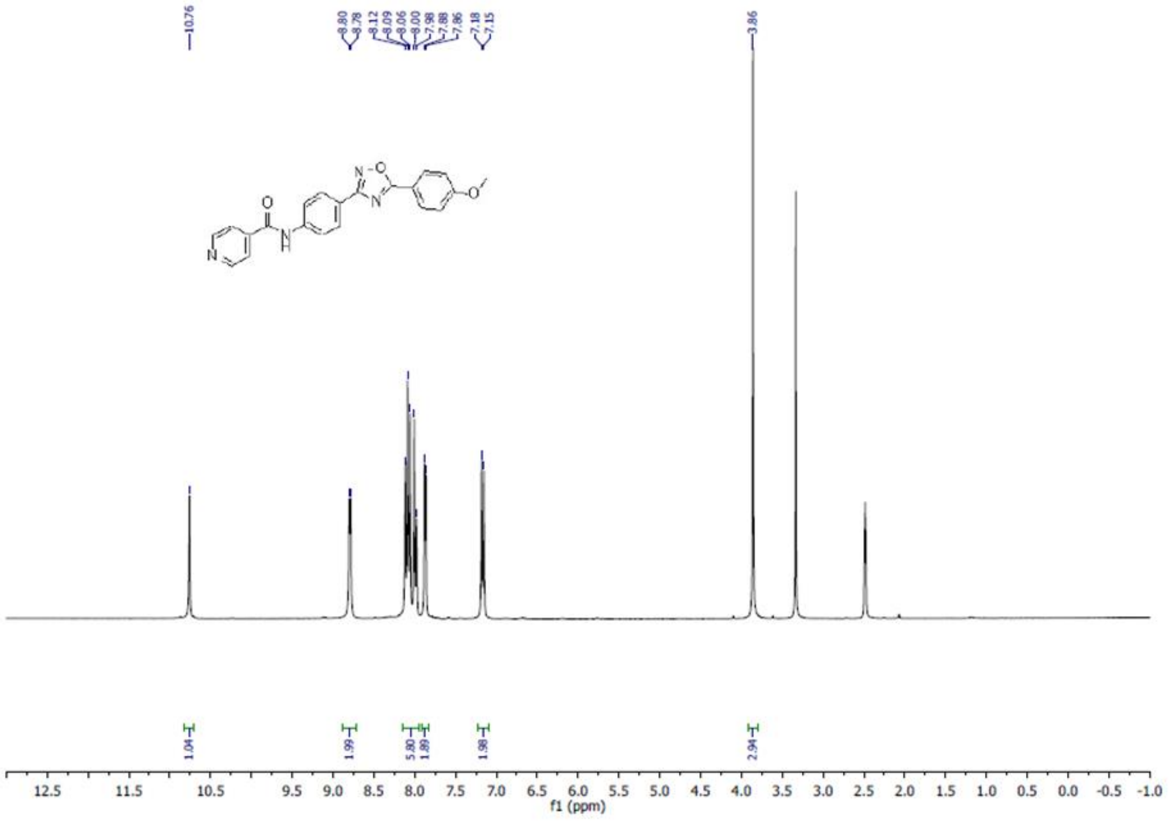

^{1}H NMR (300 MHz, DMSO-d$_6$), δ ppm: 10.76 (s br. 1H, NH), 8.79 (d br., *J* = 5.5 Hz, 2H), 8.12-7.98 (m, 6H), 7,87 (d br., *J* = 5.6 Hz, 2H), 7.17 (d br., *J* = 8.8 Hz, 2H), 3,86 (s, 3H, CH_3);

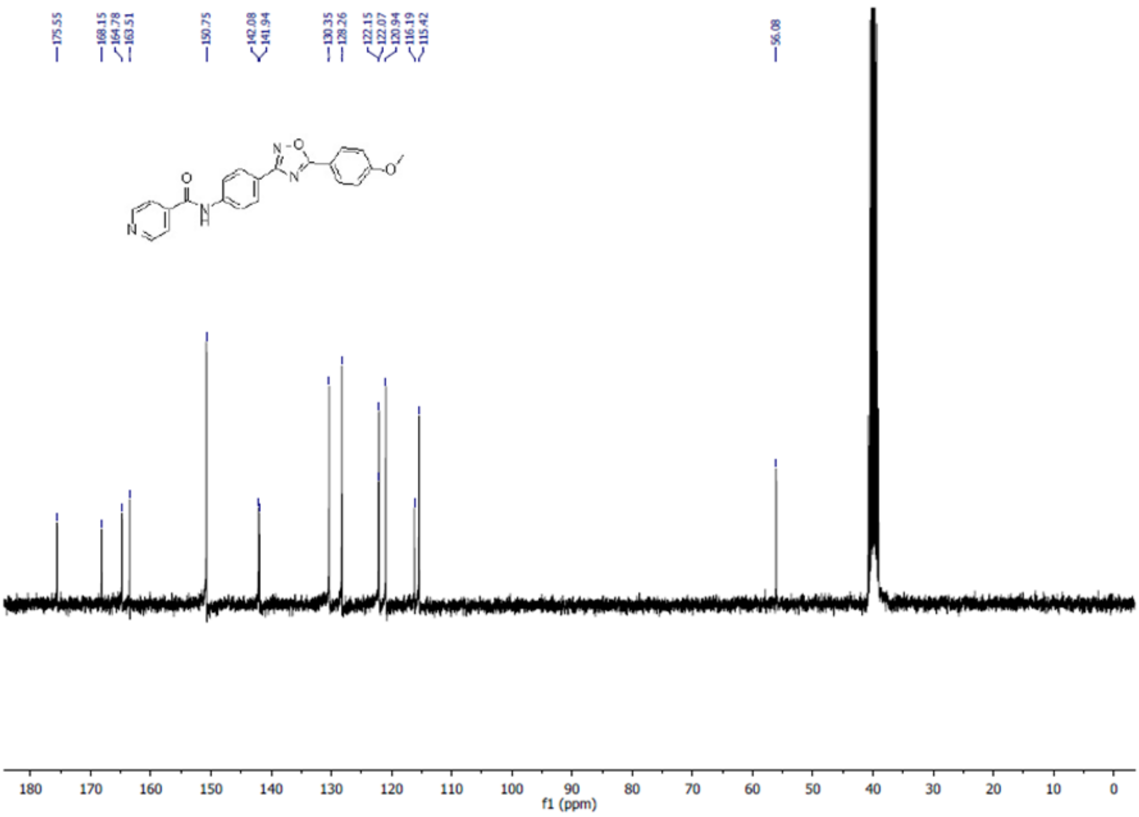

^{13}C NMR (75 MHz, DMSO-d_6), δ ppm: 175.6, 168.2, 164.8, 163.5, 150.8 (2C), 142.1, 141.9, 130.4 (2C), 128.3 (2C), 122.1, 122.1 (2C), 120.9 (2C), 116.2, 115.4 (2C), 56.1; LC-MS (method B) R_T = 3.03 min (purity: 94.18%), *m/z* found: 373.4, calc.: 373.1 [M+H$^+$].

***N*-(4-(5-(4-Methoxyphenyl)-1,2,4-oxadiazol-3-yl)phenyl)nicotinamide (17b)**

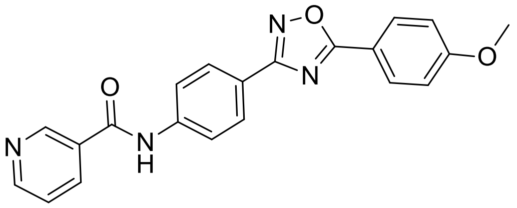

Prepared from **16a** and nicotinoyl chloride, hydrochloride according to general procedure 4. Product was purified by maceration (MeOH); white solid, 0.25 g, 89.3%.

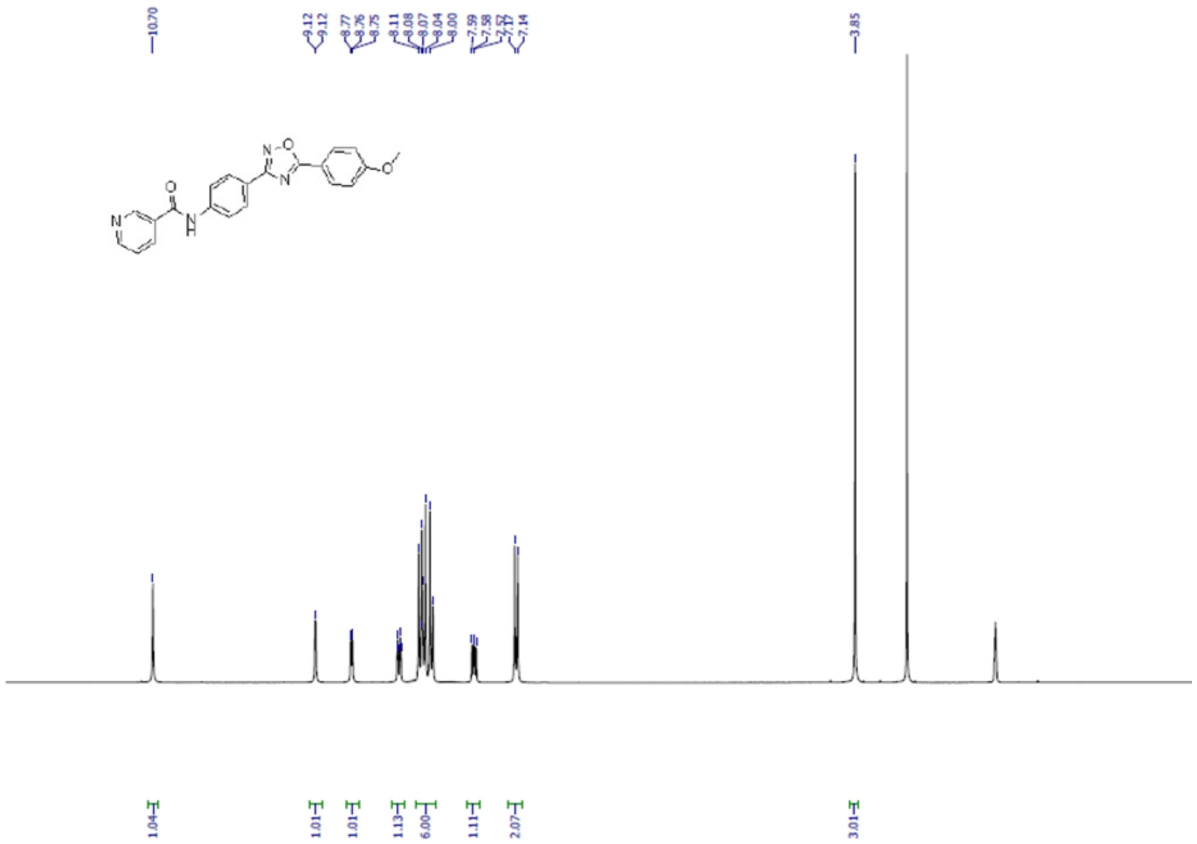

^{1}H NMR (300 MHz, DMSO-d_6), δ ppm: 10.70 (s br. 1H, NH), 9.12 (d br., *J* = 1.9 Hz, 1H), 8.76 (dd, *J* = 4.8 and 1.5 Hz, 1H), 8.31 (dt, *J* = 7.9 and 1.8 Hz, 1H), 8.11-7.97 (m, 6H), 7,57 (dd, *J* = 7.9 and 4.8 Hz, 1H), 7.16 (d, *J* = 8.9 Hz, 2H), 3,85 (s, 3H, CH_3);

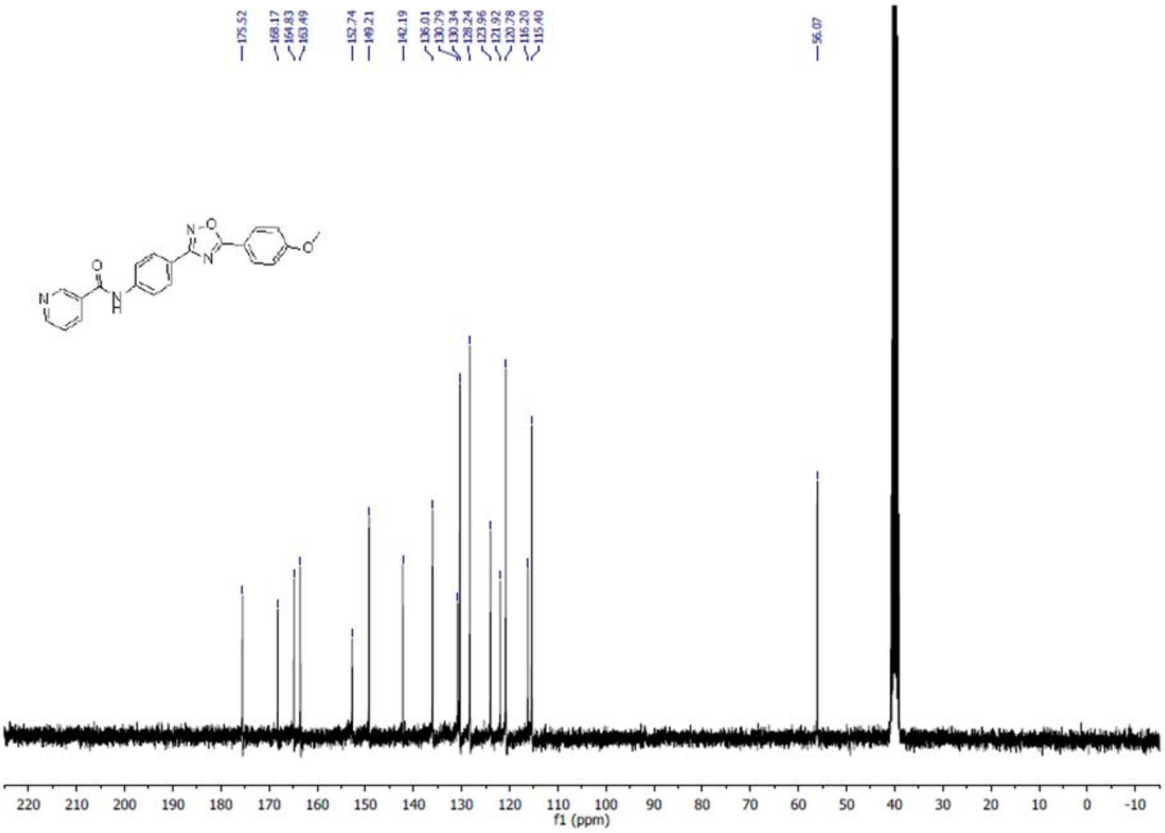

^{13}C NMR (75 MHz, DMSO-d_6), δ ppm: 175.5, 168.2, 164.8, 163.5, 152.7, 149.2, 142.2, 136.0, 130.8 (2C), 130.3, 128.2 (2C), 124.0, 121.9, 120.8 (2C), 116.2, 115.4 (2C), 56.1; LC-MS (method B) R_T = 3.06 min (purity: 95.20%), *m/z* found: 372.8, calc.: 373.1 [M+H$^+$].

***N*-(4-(5-(4-Methoxyphenyl)-1,2,4-oxadiazol-3-yl)phenyl)thiophene-2-carboxamide** (**17c**)

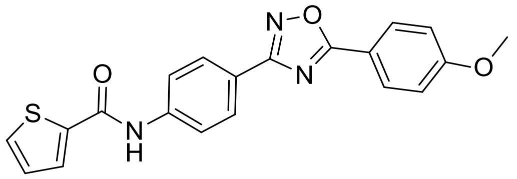

Prepared according to general procedure 4 from **16a** and 2-thiophenecarbonyl chloride. Crude product was purified by column chromatography (SiO_2, $CHCl_3$/MeOH 49:1) followed by maceration (MeOH); white solid, 0.17 g, 81.0%.

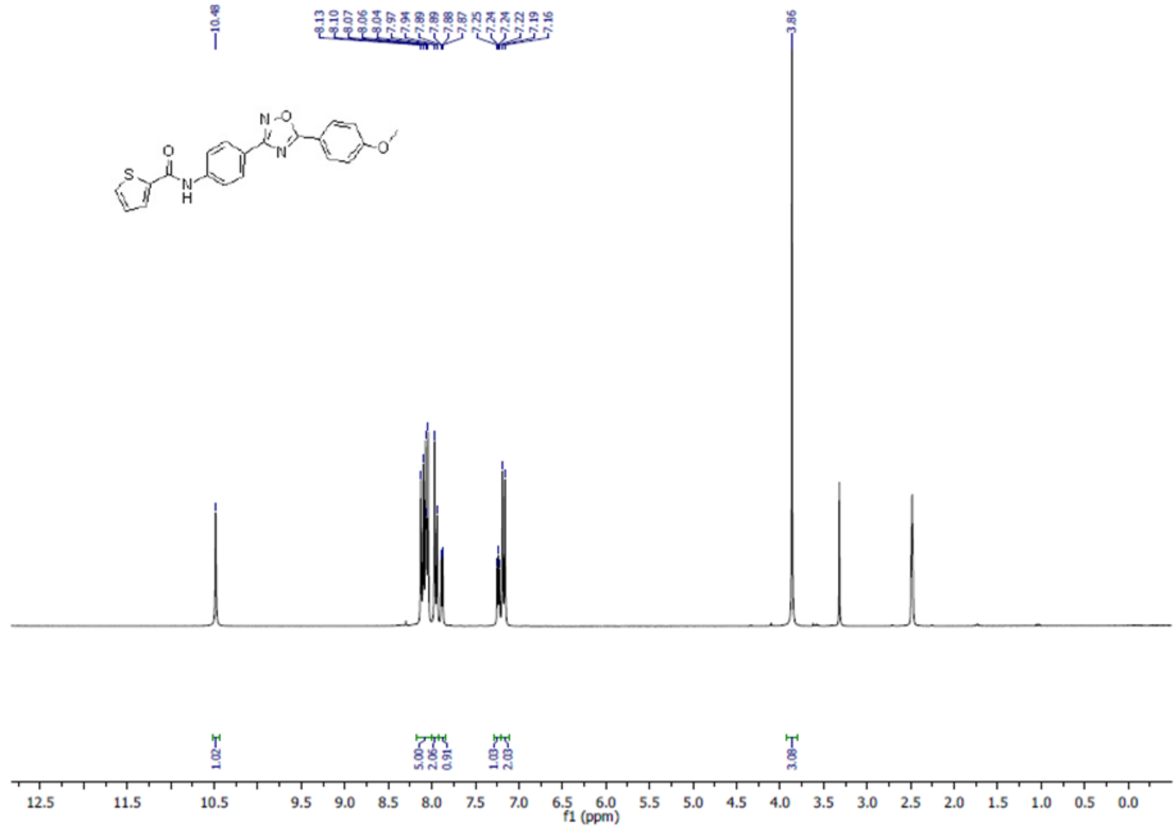

^{1}H NMR (300 MHz, DMSO-d$_6$), δ ppm: 10.48 (s br. 1H, NH), 8.13-8.04 (m, 5H), 7.97-7.95 (m, 2H), 7.88 (d br., *J* = 4.9 Hz, 1H), 7.24 (dd, *J* = 4.8 and 4.0 Hz, 1H), 7.17(d, *J* = 8.9 Hz, 2H), 3.86 (s, 3H, CH_3);

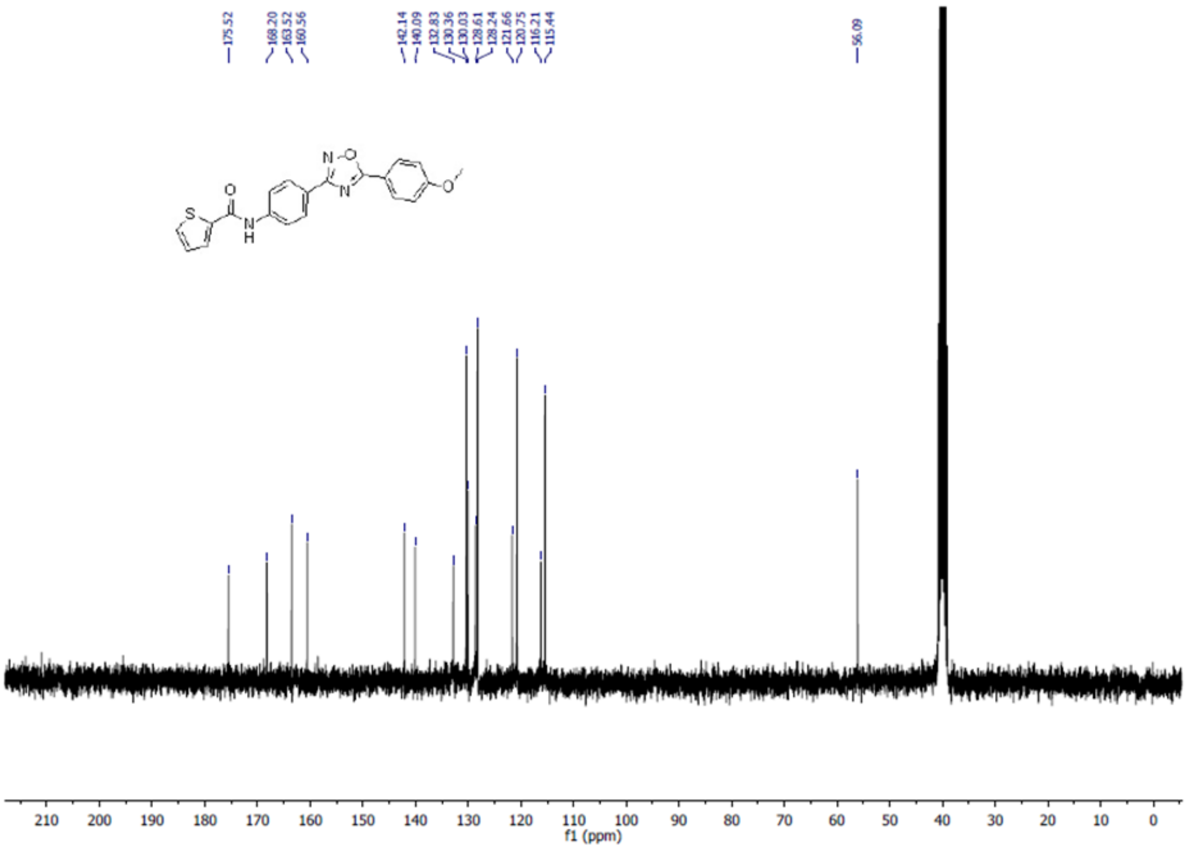

^{13}C NMR (75 MHz, DMSO-d$_6$), δ ppm: 175.5, 168.2, 163.5, 160.6, 142.1, 140.1, 132.8, 130.4 (2C), 130.0, 128.6, 128.2 (2C), 121.7, 120.8 (2C), 116.2, 115.4 (2C), 56.1; LC-MS (method B) R_T = 3.55 min (purity: 99.50%), *m/z* found: 378.2, calc.: 378.1 [M+H$^+$].

***N*-(4-(5-(2-Chlorophenyl)-1,2,4-oxadiazol-3-yl)phenyl)picolinamide** (**17d**)

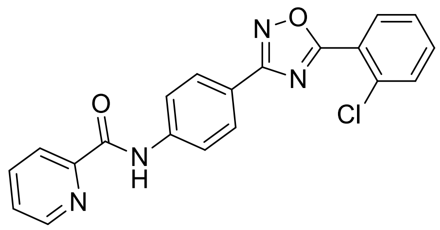

Prepared from **16b** and 2-pyridinecarbonyl chloride, hydrochloride according to general procedure 4. Crude product was purified by column chromatography (SiO_2, $CHCl_3$/MeOH 99:1) followed by maceration (2-PrOH/hexane 1:2); white solid, 0.19 g, 91.8%.

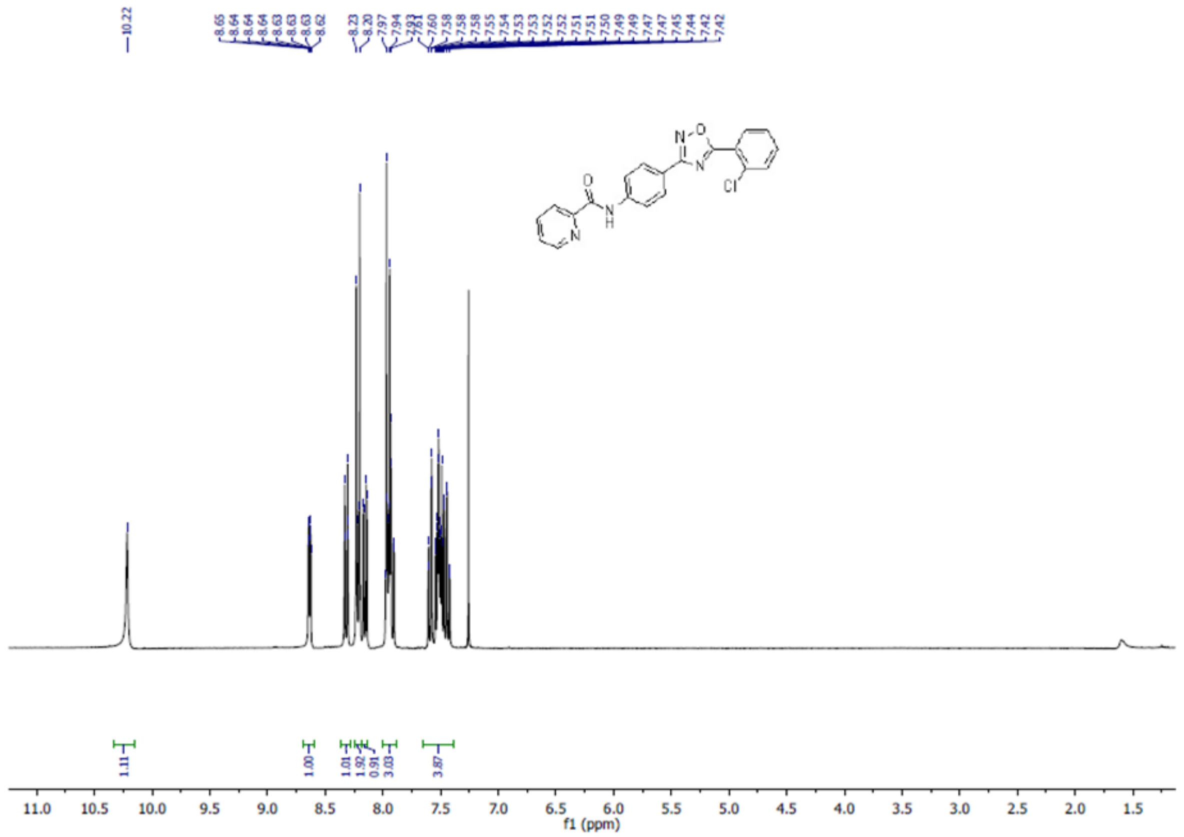

^{1}H NMR (300 MHz, $CDCl_3$), δ ppm: 10.22 (s br. 1H, NH), 8.64 (ddd, *J* = 4.8, 1.6 and 0.9 Hz, 1H), 8.32 (dt, *J* = 7.8 and 1.0 Hz, 1H), 8.24-8.19 (m, 2H), 8.15 (dd, *J* = 7.6 and 1.8 Hz, 1H), 7.98-7.90 (m, 3H), 7.61-7.42 (m, 4H).

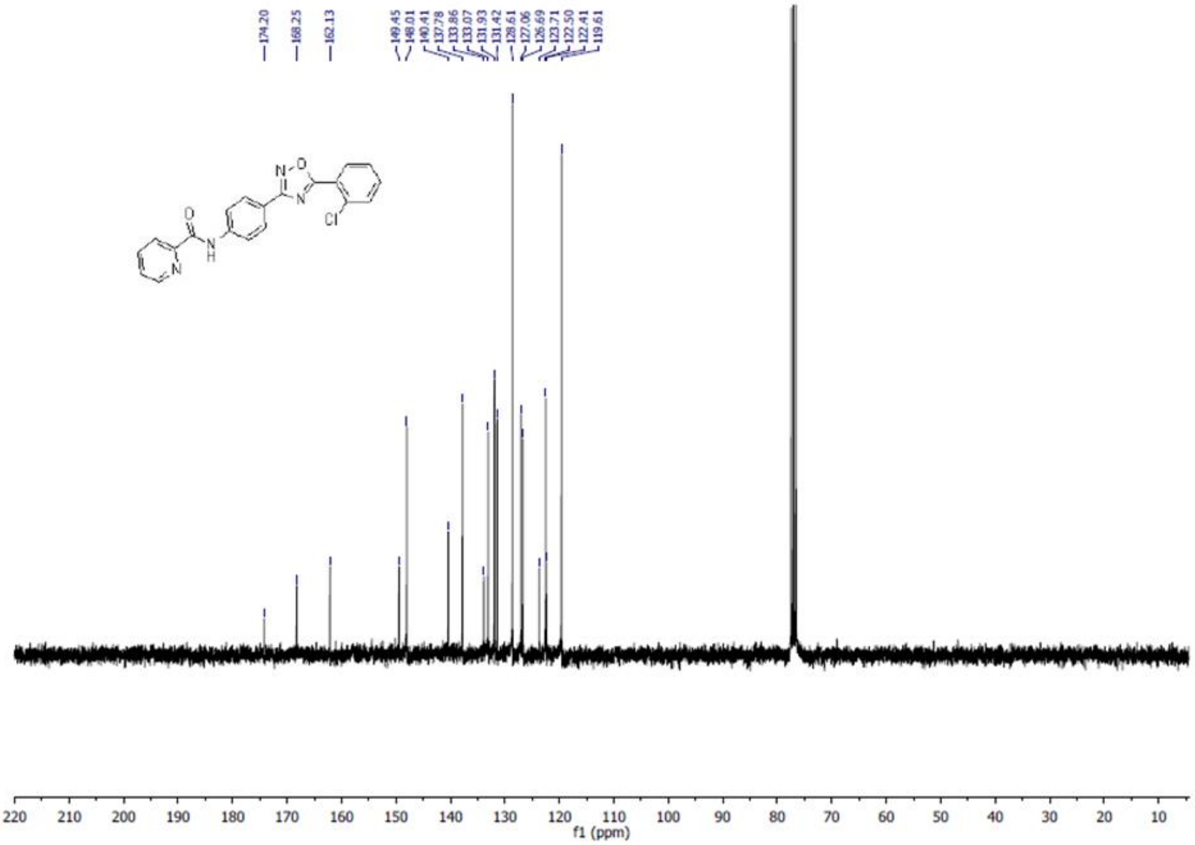

^{13}C NMR ($CDCl_3$), δ ppm: 174.2, 168.2, 162.1, 149.4, 148.0, 140.4, 137.8, 133.9, 133.1, 131.9, 131.4, 128.6 (2C), 127.1, 126.7, 123.7, 122.5, 122.4, 119.6 (2C); LC-MS (method A) R_T = 3.82 min (purity: 100%), *m/z* found: 377.1, calc.: 377.1 [$M+H^+$].

***N*-(4-(5-(2-Chlorophenyl)-1,2,4-oxadiazol-3-yl)phenyl)-5-methylisoxazole-3-carboxamide (17e)**

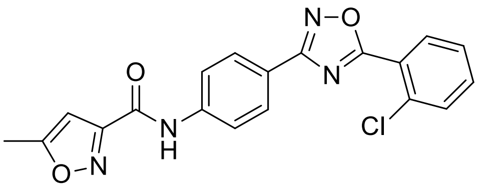

Prepared according to general procedure 4 from **16b** and 5-methyloxazole-3-carbonyl chloride. Crude product was purified by column chromatography (SiO_2 $CHCl_3$/hexane 3:1) followed by maceration (2-PrOH/hexane 1:2); white solid, 0.10 g, 79.4%.

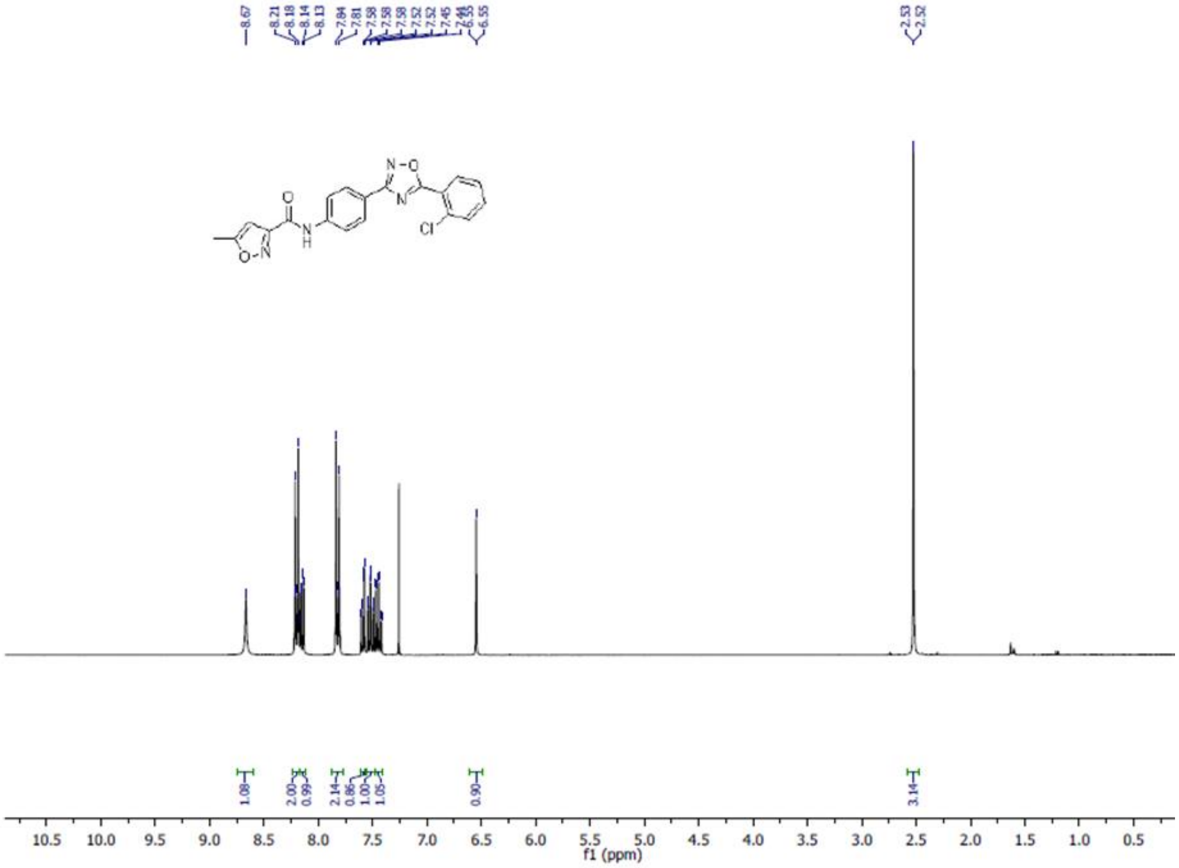

^{1}H NMR ($CDCl_3$), δ ppm: 8.67 (s br. 1H, NH), 8.21-8.18 (m, 2H), 8.15 (dd, *J* = 7.6 and 1.7 Hz, 1H), 7.84-7.80 (m, 2H), 7.59 (dd, *J* = 7.8 and 1.3 Hz, 1H), 7.52 (td, *J* = 7.3 and 1.8 Hz, 1H), 7.43 (td, *J* = 7.7 and 1.6 Hz, 1H), 6.55 (d, *J* = 0.9 Hz, 1H), 2.53 (d, *J* = 0.8 Hz, 3H, CH_3).

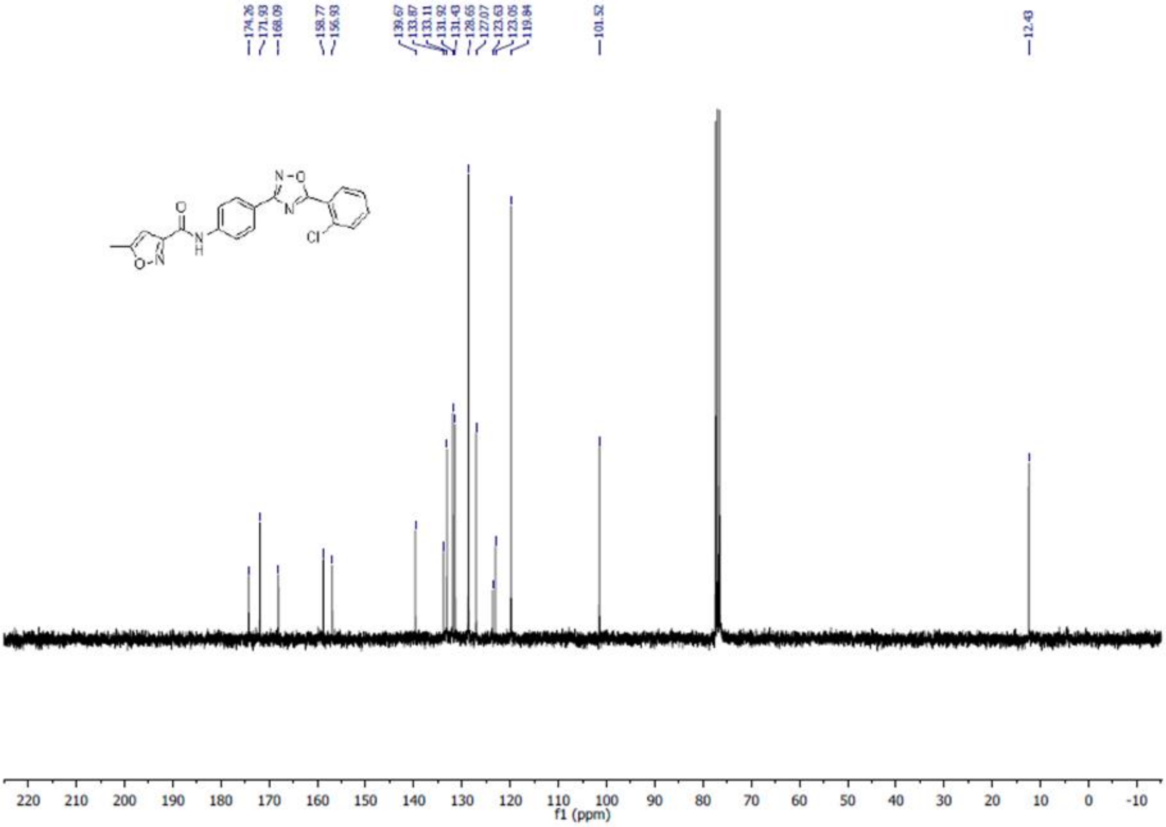

^{13}C NMR ($CDCl_3$), δ ppm: 174.3, 171.9, 168.1, 158.8, 156.9, 139.7, 133.9, 133.1, 131.9, 131.4, 128.6 (2C), 127.1, 123.6, 123.0, 119.8 (2C), 101.5, 12.4; LC-MS (method A) R_T = 3.61 min (purity:100%), *m/z* found: 381.1, calc.: 381.1 [M+H$^+$].

***N*-(4-(5-(4-Fluorophenyl)-1,2,4-oxadiazol-3-yl)phenyl)isoxazole-5-carboxamide(17f)**

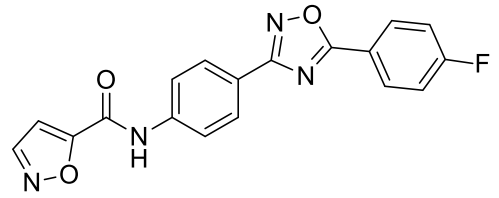

Prepared from **16c** and isoxazole-5-carbonyl chloride according to general procedure 4. Crude product was purified by maceration (2-PrOH); white solid, 0.19 g, 70.4%.

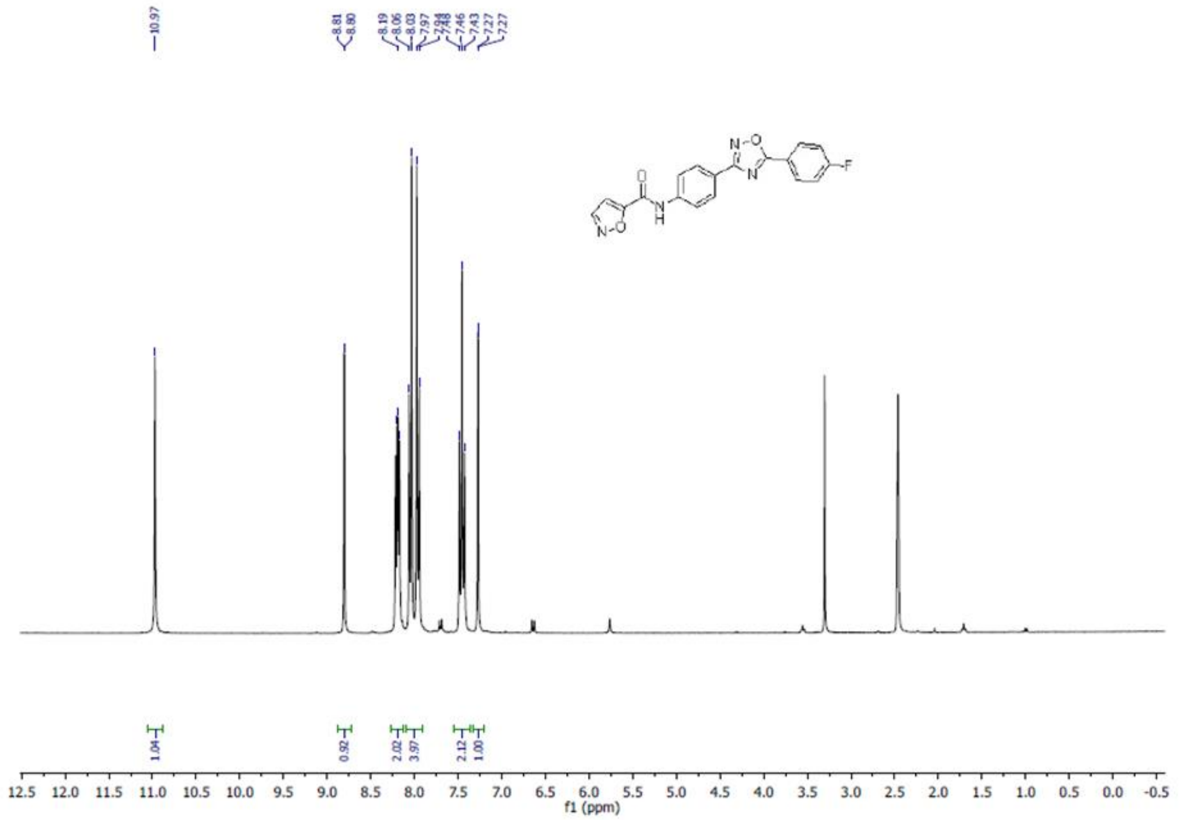

^{1}H NMR (300 MHz, DMSO-d$_6$), δ ppm: 10.99 (s br. 1H, NH), 8.83 (d br., *J* = 1.8, 1H), 8.22-8.17 (m, 2H), 8.06-7.94 (m, 4H), 7.48-7.43 (m, 2H), 7.29 (d, *J* = 1.8 Hz, 1H).

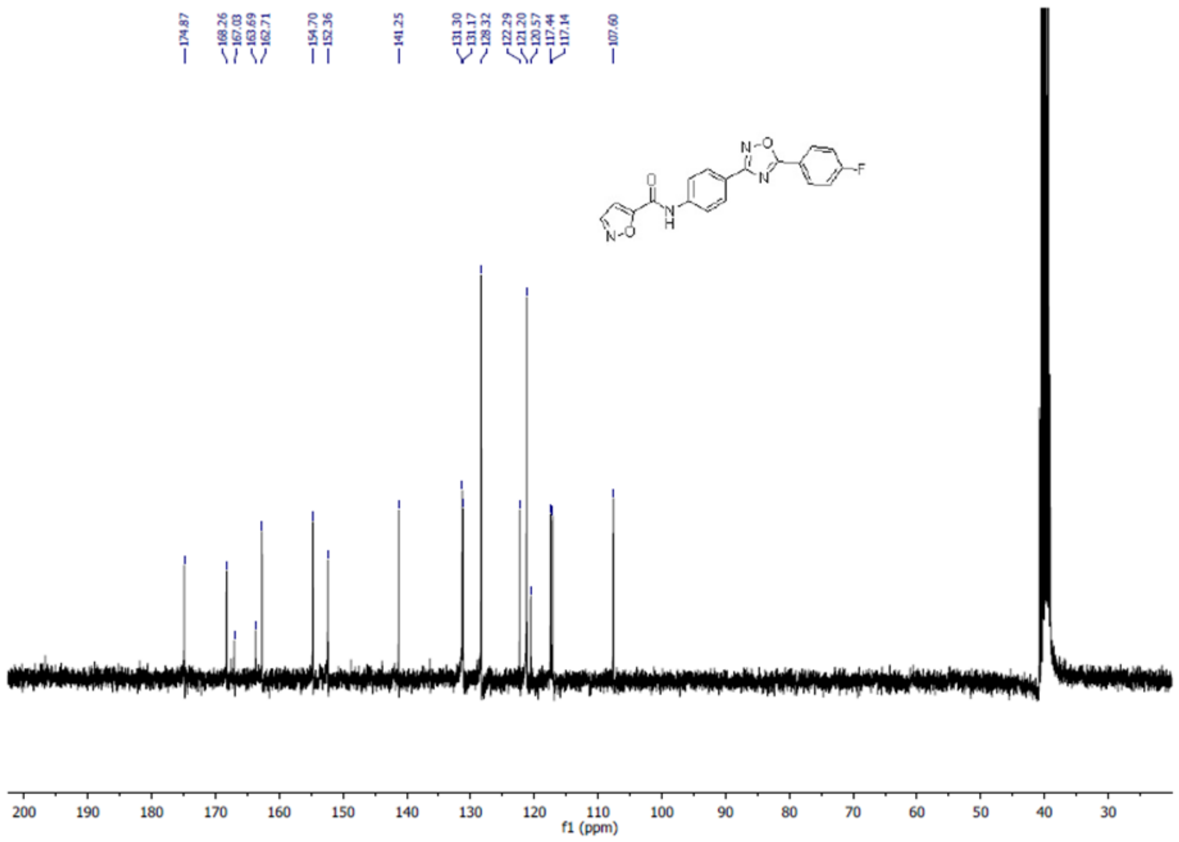

^{13}C NMR (75 MHz, DMSO-d$_6$), δ ppm: 174.9, 168.3, 165.3 (d, *J* = 252.1 Hz), 162.7, 154.7, 152.4, 141.3, 131.2 (d, *J* = 9.5 Hz, 2C), 128.3 (2C), 122.3, 121.2 (2C), 120.6, 117.3 (d, *J* = 22.5 Hz, 2C), 107.6; LC-MS (method B) R_T = 3.34 min (purity: 95.36%), *m/z* found: 351.1, calc.: 351.1 [M+H$^+$].

2-Chloro-*N*-(4-(5-(4-fluorophenyl)-1,2,4-oxadiazol-3-yl)phenyl)nicotinamide(**17g**)

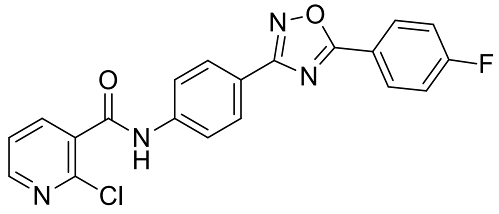

Prepared from **16c** and 2-chloropyridine-3-carbonyl chloride according to general procedure 4. Crude product was purified by column chromatography (SiO_2, $CHCl_3$/MeOH 99:1) followed by maceration (2-PrOH); white solid, 0.22 g, 71.0%.

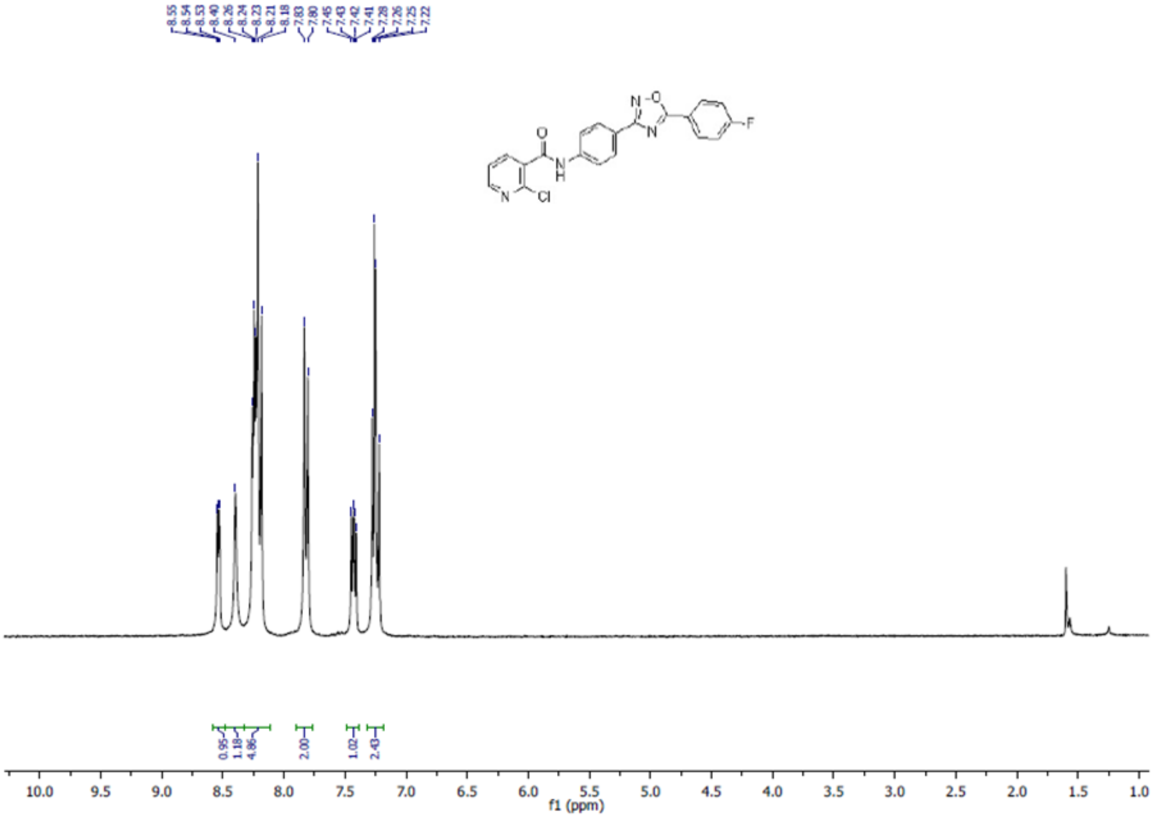

^{1}H NMR ($CDCl_3$), δ ppm: 8.54 (dd, *J* = 4.7 and 1.9 Hz, 1H), 8.40 (s br. 1H, NH), 8.26-8.18 (m, 5H), 7.81 (d, *J* = 8.6 Hz, 2H), 7.42 (dd, *J* = 7.6 and 4.8 Hz, 1H), 7.28-7.21 (m, 2H);

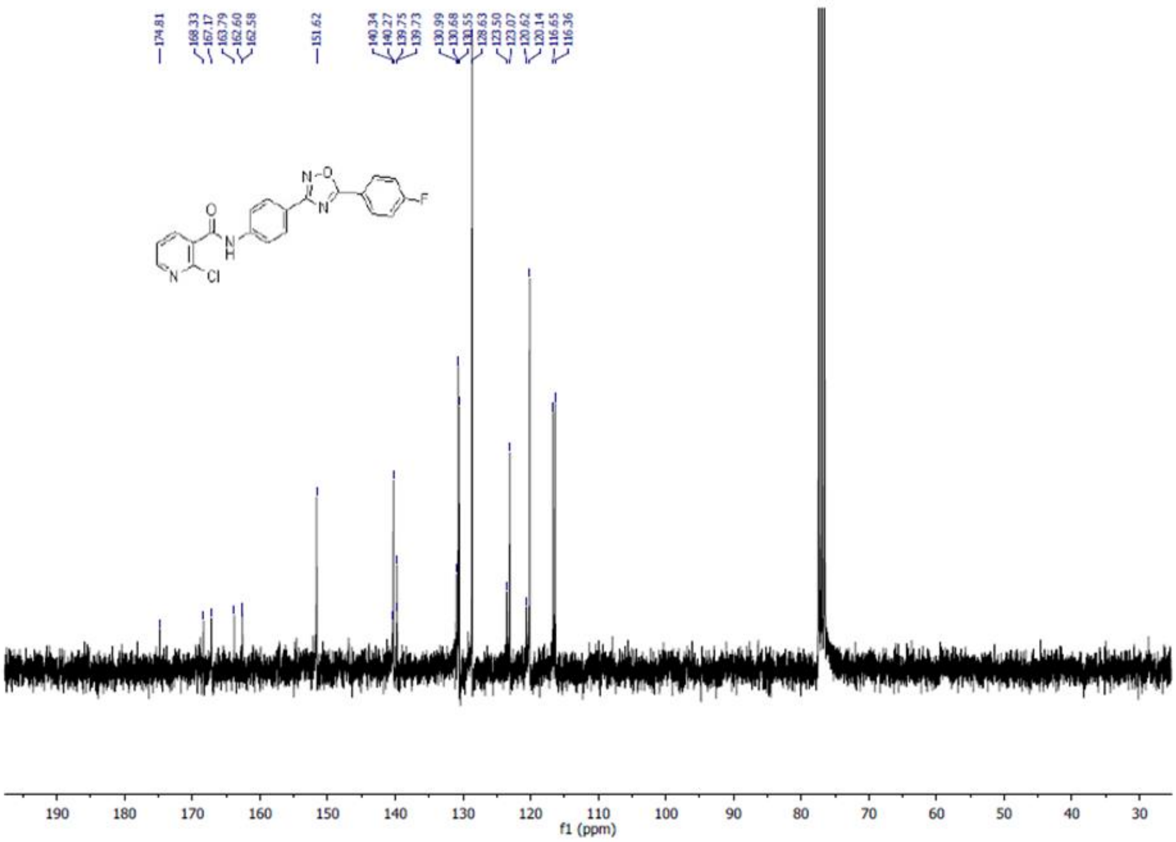

^{13}C NMR ($CDCl_3$), δ ppm: 174.8, 168.3, 165.5 (d, *J* = 254.9 Hz), 162.6, 151.6, 140.3, 139.8, 131.0, 130.7 (d, *J* = 9.2 Hz, 2C), 128.6 (3C), 123.5, 123.1, 120.6, 120.1 (2C), 116.5 (d, *J* = 22.3 Hz, 2C); LC-MS (method B) R_T = 3.39 min (purity: 98.30%), *m/z* found: 395.0, calc.: 395.1 [$M+H^+$].

***N*'-Hydroxybenzimidamide (19a)**

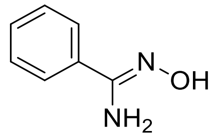

Prepared from benzonitrile according to general procedure 1 and the method described in the literature. N'-hydroxybenzimidamide is fully described in the literature [3]. ^{1}H NMR ($CDCl_3$), δ ppm: 5.76 (s, 2H, NH), 7.16-7.18 (m, 3H), 7.71 (d, *J* = 8 Hz, 2H), 9.58 (s, 1H, OH); ^{13}C NMR ($CDCl_3$), δ ppm: 128.09 (2C), 128.19 (2C), 128.38, 131.24, 168.01

***N*'-Hydroxypicolinimidamide (19b)**

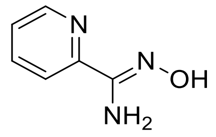

Prepared from pyridine-2-carbonitrile according to general procedure 1. white crystals, 4.09 g, 62.2%. LC-MS (method B) R_T = 0.47 min (purity: 100%), *m/z* found: 138.2, calc.: 138.1 [M+H$^+$]. *The product was used directly to the next step.*

5-(4-Nitrophenyl)-3-phenyl-1,2,4-oxadiazole (20a)[4]

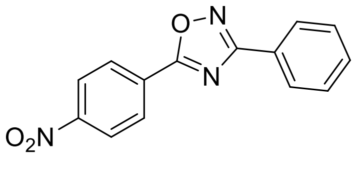

Prepared from **19a** and 4-nitrobenzoyl chloride according to general procedure 2 (MW); white crystals, 7.88 g, 80.3%. LC-MS (method A) R_T = 3.76 min (purity: 98.88%), *m/z* found: 268.4, calc. 268.1 [M+H$^+$]. *The product was used directly to the next step.*

5-(4-Nitrophenyl)-3-(pyridin-2-yl)-1,2,4-oxadiazole (20b)

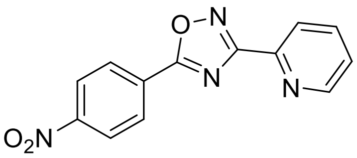

Prepared according to general procedure 2 from **19b** and 4-nitrobenzoyl chloride (MW). Crude product was purified by maceration (2-PrOH/hexane 1:2); yellow crystals, 2.94 g, 75.4%. LC-MS (method A) R_T = 2.86 min (purity: 83.68%), *m/z* found: 269.0, calc. 269.1 [M+H$^+$]. *The product was used in the next steps without further purification*.

4-(3-Phenyl-1,2,4-oxadiazol-5-yl)aniline (21a)

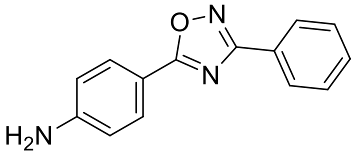

Prepared from **20a** according to general procedure 3, method A. Precipitated solid was purified by flash column chromatography (SiO_2, $CHCl_3$) followed by maceration (2-PrOH); white crystals, 1.72 g, 64.7%. LC-MS (method B) R_T = 3.15 min (purity: 100%), *m/z* found: 237.9, calc.: 238.1 [M+H$^+$]. *The product was used directly to the next step.*

4-(3-(Pyridin-2-yl)-1,2,4-oxadiazol-5-yl)aniline (21b)

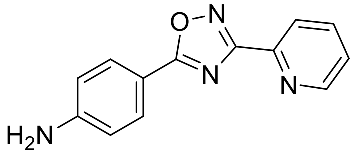

Prepared according to general procedure 3, method C from **20b**. Crude product was purified by column chromatography (SiO_2, $CHCl_3$/MeOH 49:1); pale yellow solid, 0.98 g, 38.8%. ^{1}H NMR (300 MHz, DMSO-d$_6$), δ ppm: 8.77 (ddd, *J* = 4.7, 1.6 and 0.9 Hz, 1H), 8.12 (dt, *J* = 7.8 and 1.0 Hz, 1H), 8.04 (td, *J* = 7.6 and 1.7 Hz, 1H), 7.87-7.83 (m, 2H), 7.60 (ddd, *J* = 7.6, 4.8 and 1.3 Hz, 1H), 6.74-6.69 (m, 2H), 6.23 (s br. 2H, NH_2); ^{13}C NMR (75 MHz, DMSO-d$_6$), δ ppm:

176.7, 168.3, 154.2, 150.6, 146.7, 138.0, 130.2 (2C), 126.3, 123.7, 113.9 (2C), 109.8; LC-MS (method A) R_T = 2.30 min (purity: 92.07%), *m/z* found: 238.8, calc.: 239.1 [$M+H^+$].

***N*-(4-(3-Phenyl-1,2,4-oxadiazol-5-yl)phenyl)benzamide (22a)**

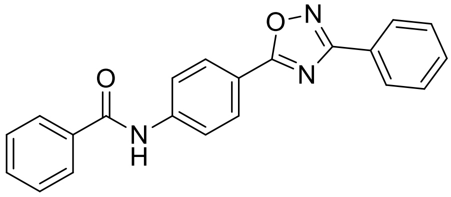

Prepared according to general procedure 4 from **21a** and benzoyl chloride. Crude product was purified by maceration (MeOH/water 2:1); white solid, 0.27 g, 93.8%.

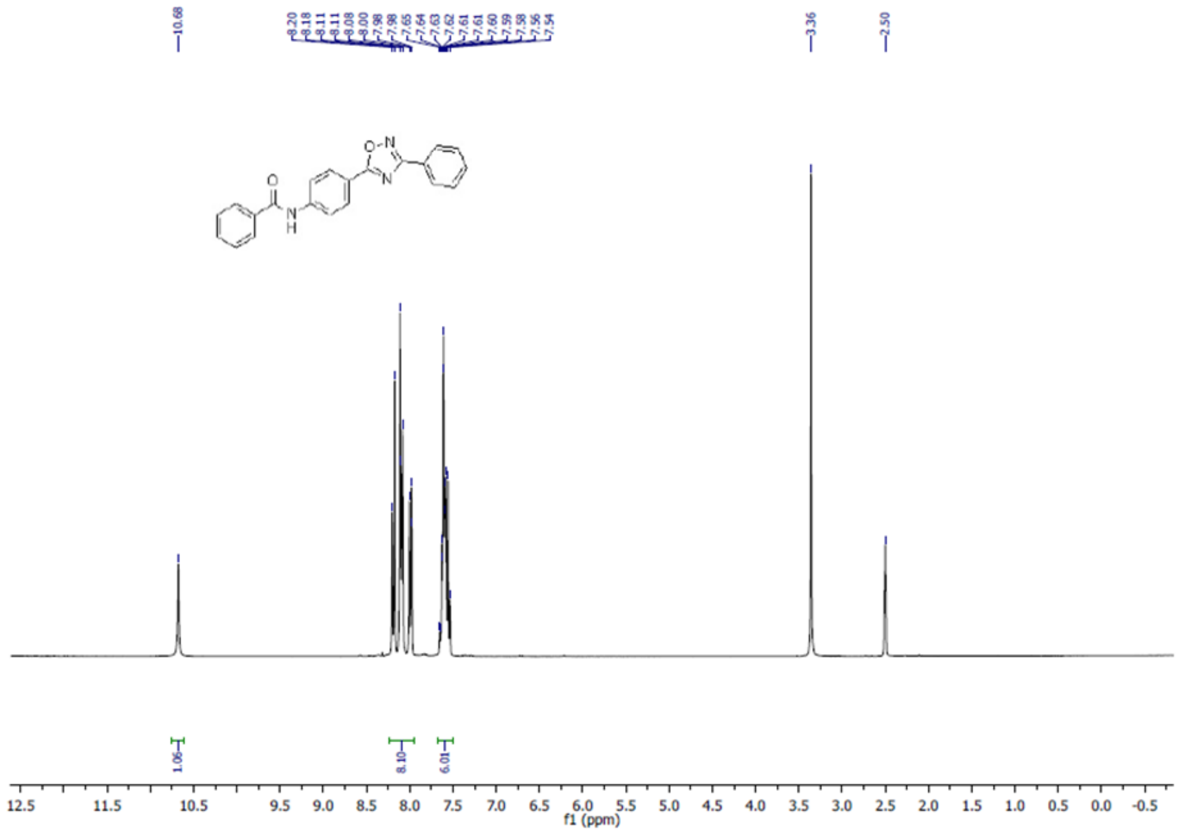

^{1}H NMR (300 MHz, DMSO-d_6), δ ppm: 10.68 (s br. 1H, NH), 8.20-7.98 (m, 8H), 7.63-7.54 (m, 6H);

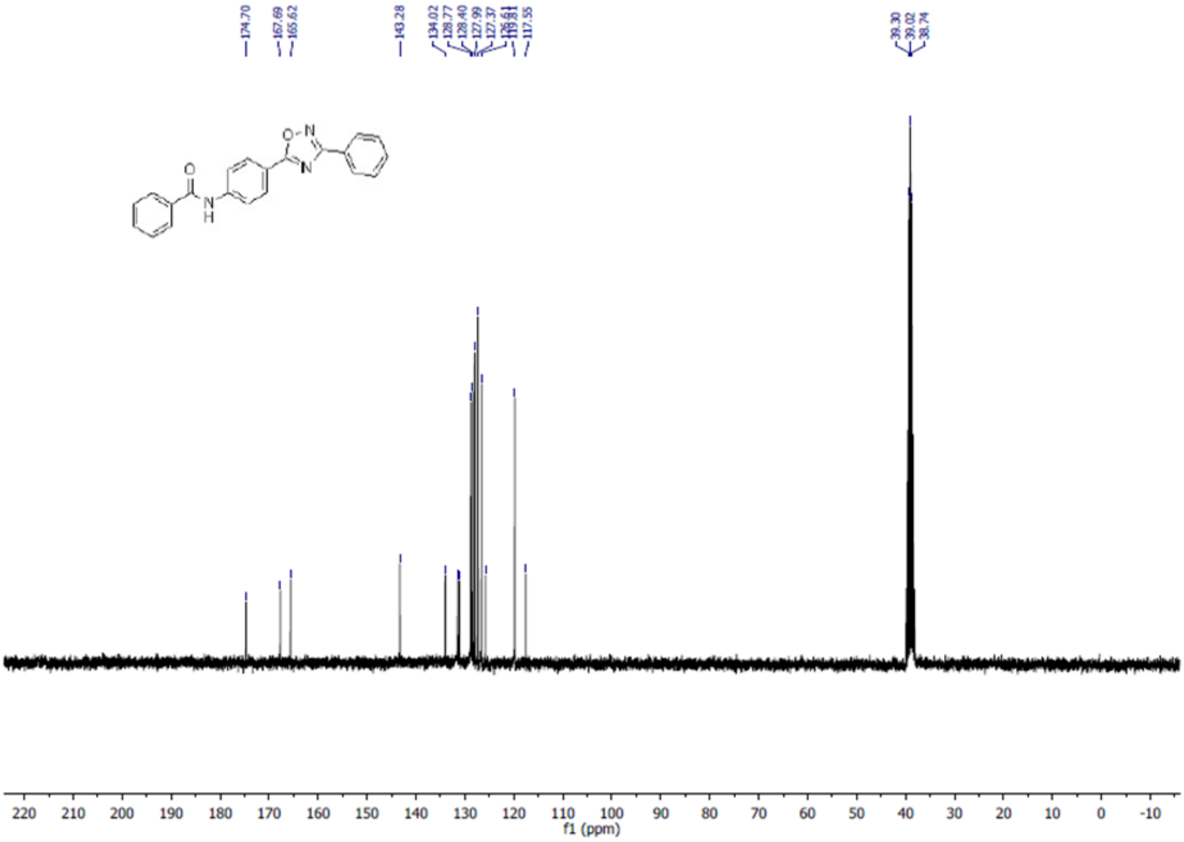

^{13}C NMR (75 MHz, DMSO-d_6), δ ppm: 174.7, 167.7, 165.6, 143.3, 134.0, 131.5, 131.1, 128.8 (2C), 128.4 (2C), 128.0 (2C), 127.4 (2C), 126.6 (2C), 125.8, 119.8 (2C), 117.5; LC-MS (method B) R_T = 3.71 min (purity: 96.43%), *m/z* found: 342.1, calc.: 342.1 [M+H$^+$].

4-Methoxy-*N*-(4-(3-phenyl-1,2,4-oxadiazol-5-yl)phenyl)benzamide (**22b**)

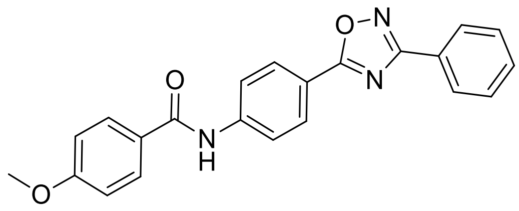

Prepared according to general procedure 4 from **21a** and 4-methoxybenzoyl chloride. Crude product was purified by maceration (MeOH); white solid, 0.30 g, 96.7%.

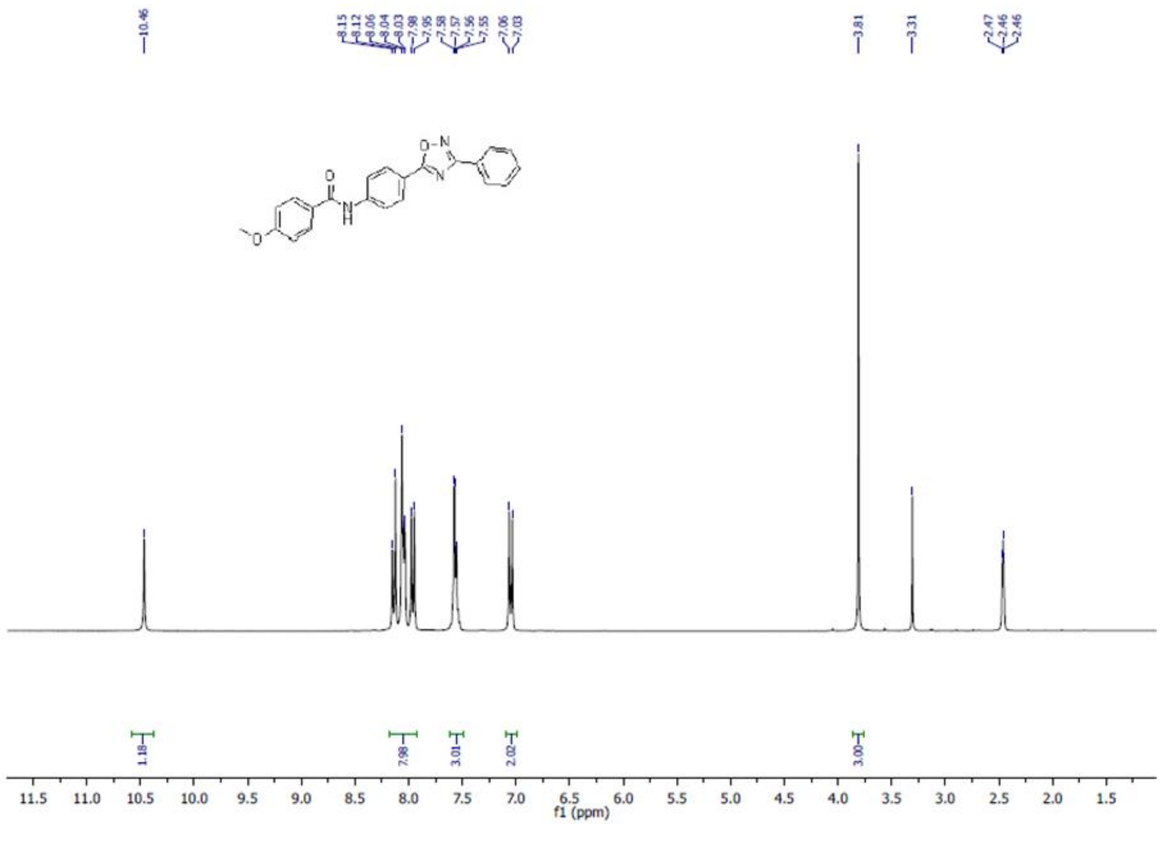

^{1}H NMR (300 MHz, DMSO-d$_6$), δ ppm: 10.46 (s br. 1H, NH), 8.15-7.98 (m, 8H), 7.58-7.54 (m, 3H), 7.04 (d br., *J* = 8.8 Hz, 2H); 3.81 (s, 3H).

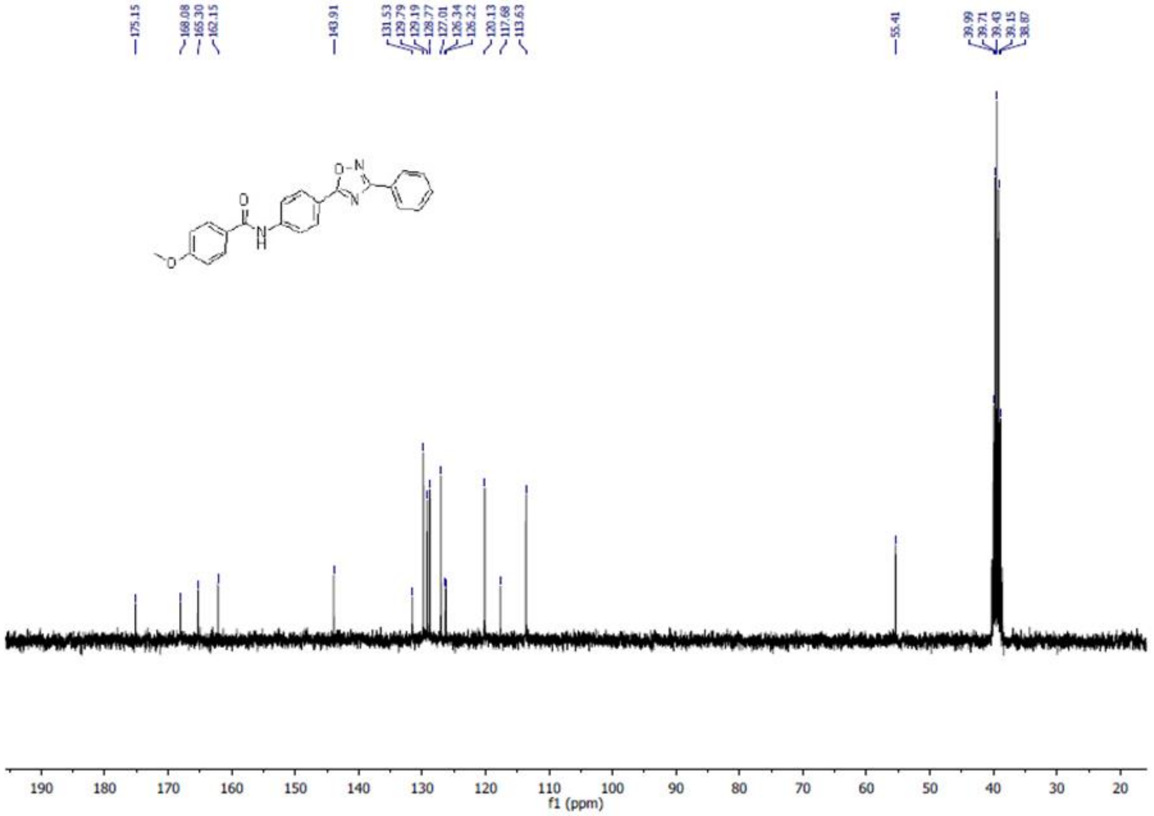

^{13}C NMR (75 MHz, DMSO-d_6), δ ppm: 175.1, 168.1, 165.3, 162.1, 143.9, 131.5, 129.8 (2C), 129.2 (2C), 128.8 (2C), 127.0 (2C), 126.3, 126.2, 120.1 (2C), 117.7, 113.6 (2C), 55.4; LC-MS (method B) R_T = 3.71 min (purity: 97.46%), *m/z* found: 372.7, calc.: 372.1 [M+H$^+$].

***N*-(4-(3-(Pyridin-2-yl)-1,2,4-oxadiazol-5-yl)phenyl)benzamide** (**22c**)

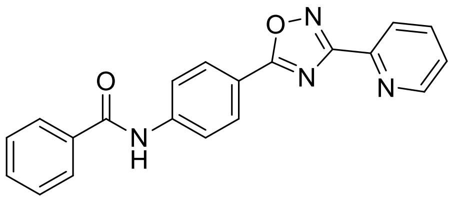

Prepared according to general procedure 4 from **21b** and benzoyl chloride. Crude product was purified by maceration (2-PrOH/hexane 1:2); white solid, 0.19 g, 88.4%.

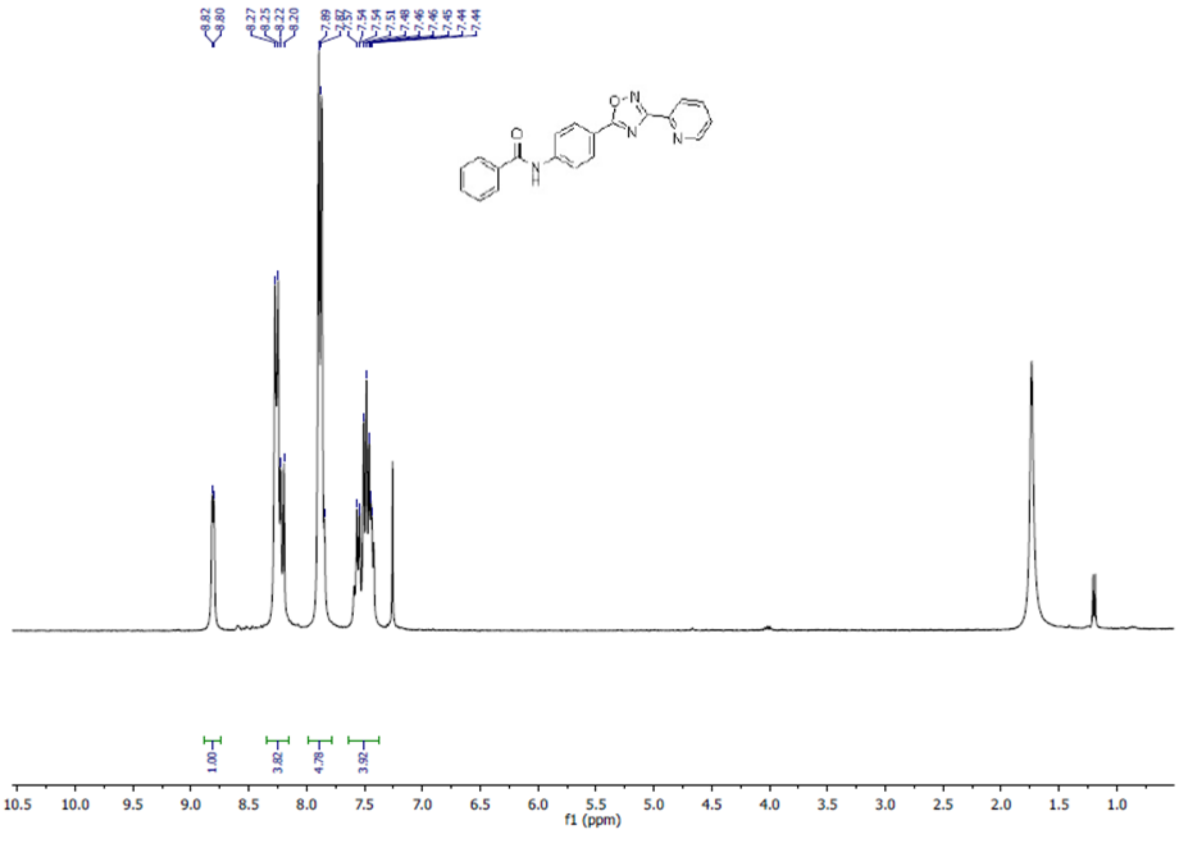

^{1}H NMR (300 MHz, $CDCl_3$), δ ppm: 8.81 (d br., *J* = 3.9 Hz, 1H), 8.27-8.20 (m, 4H), 7.89-7.85 (m, 5H), 7.59-7.42 (m, 4H);

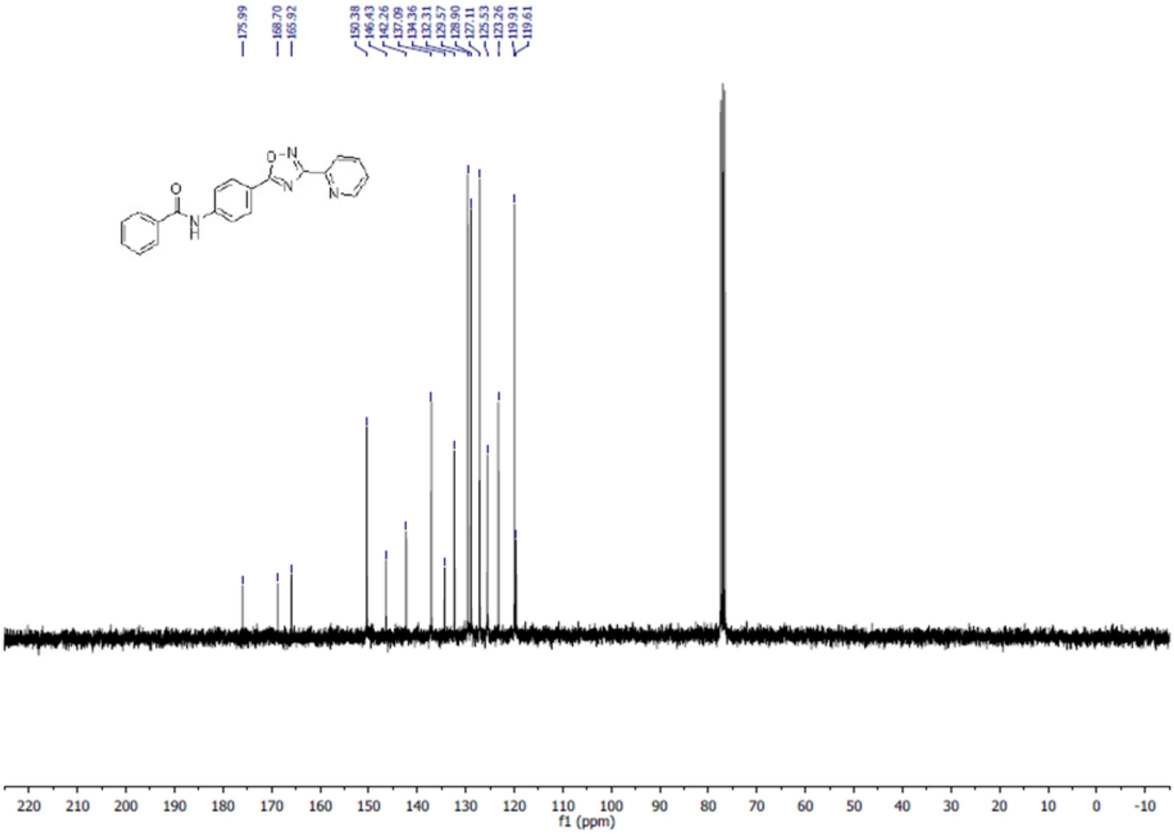

^{13}C NMR (75 MHz, $CDCl_3$), δ ppm: 176.0, 168.7, 165.9, 150.4, 146.4, 142.3, 137.1, 134.4, 132.3, 129.6 (2C), 128.9 (2C), 127.1 (2C), 125.5, 123.3, 119.9 (2C), 119.6; LC-MS (method B) R_T = 2.96 min (purity: 99.25%), *m/z* found: 343.2, calc.: 342.1 [$M+H^+$].

2-Chloro-*N*-(4-(3-(pyridin-2-yl)-1,2,4-oxadiazol-5-yl)phenyl)benzamide (22d)

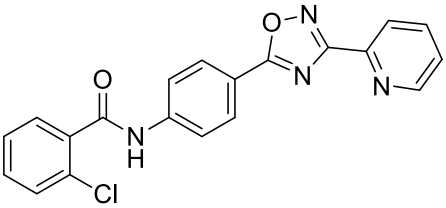

Prepared according to general procedure 4 from **21b** and 2-chlorobenzoyl chloride. Crude product was purified by column chromatography (SiO_2, $CHCl_3$/MeOH 49:1) followed by maceration (2-PrOH/hexane 1:2); white solid, 0.20 g, 87.0%.

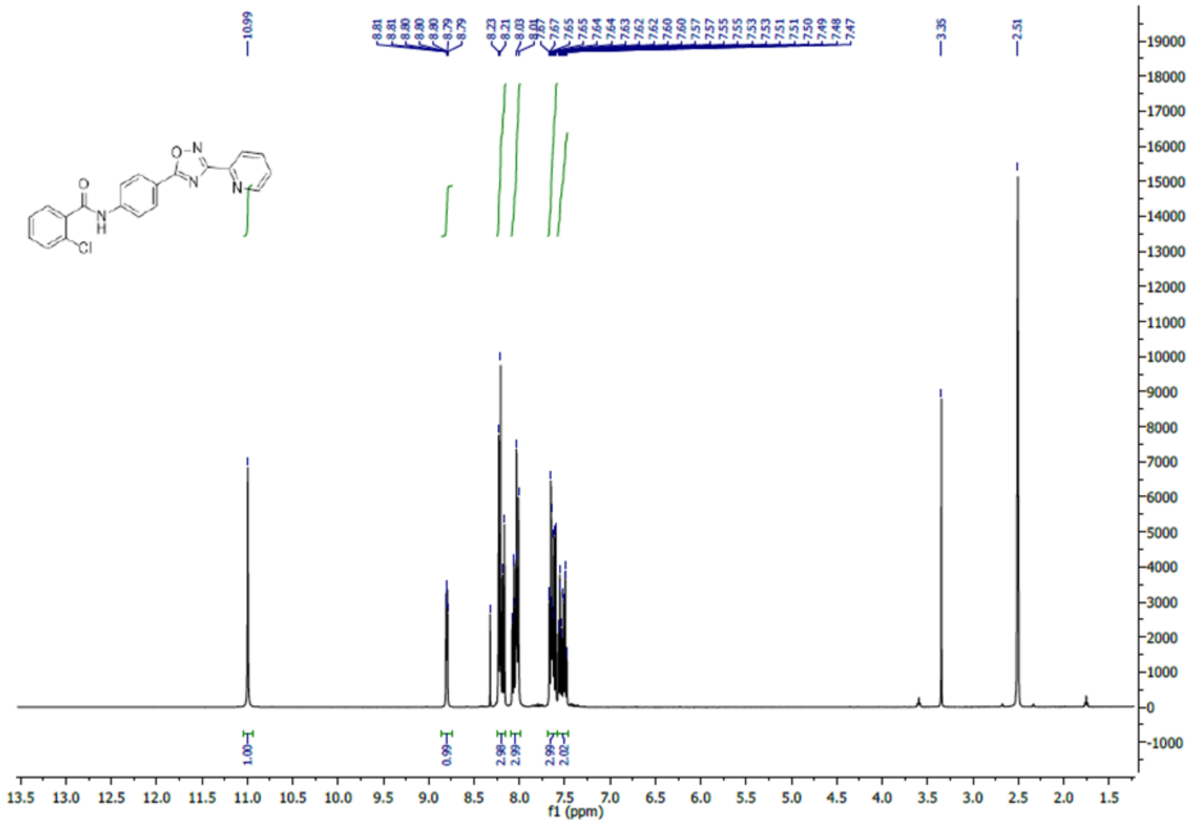

^{1}H NMR (400 MHz, DMSO-d6) δ 10.99 (s, 1H), 8.84 – 8.77 (m, 1H), 8.20 (dd, *J* = 17.6, 8.3 Hz, 3H), 8.05 (ddd, *J* = 12.4, 9.2, 5.2 Hz, 3H), 7.69 – 7.59 (m, 3H), 7.52 (dtd, *J* = 22.2, 7.4, 1.5 Hz, 2H).

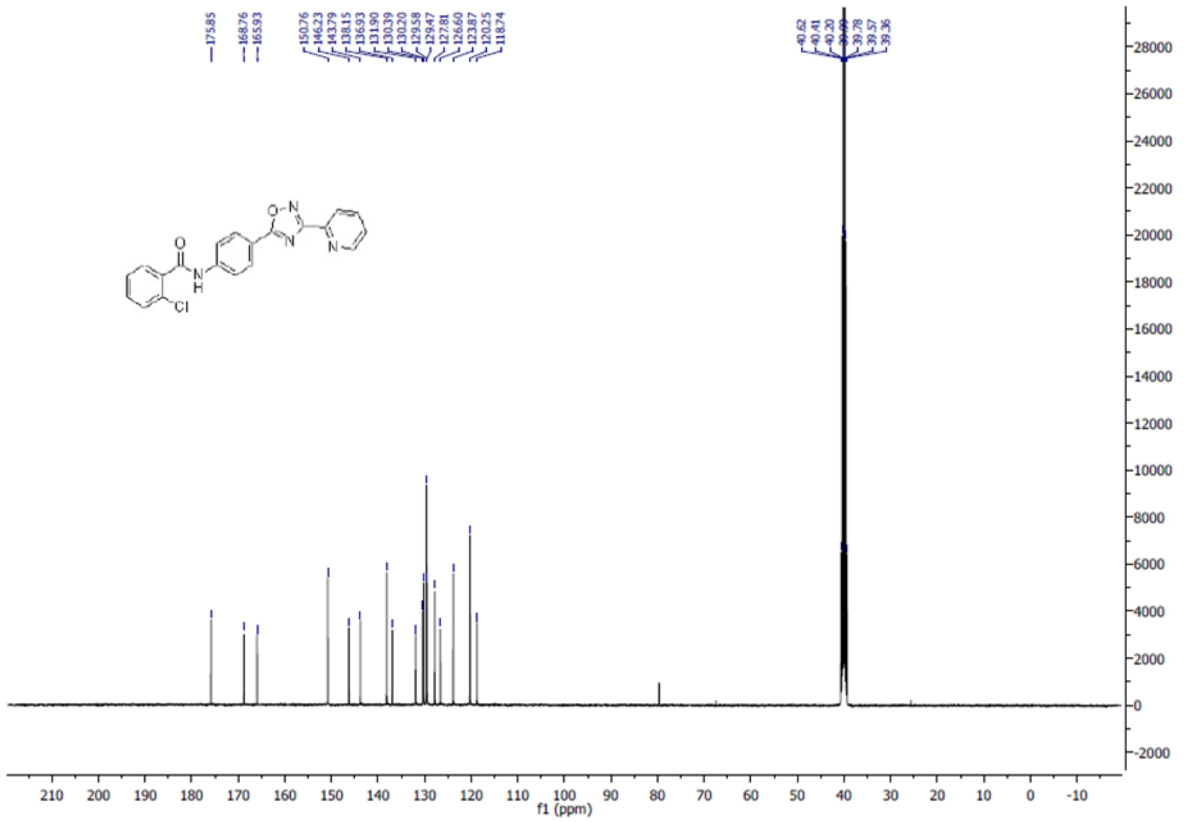

^{13}C NMR (101 MHz, DMSO-d6), δ ppm: 175.85, 168.76, 165.93, 150.76, 146.23, 143.79, 138.15, 136.93, 131.90, 130.39, 130.20, 129.58 (2C), 129.47, 127.81, 126.60, 123.87, 120.25(2C), 118.74, LC-MS (method B) R_T = 3.03 min (purity: 100%), *m/z* found: 376.8, calc.: 377.1 [M+H$^+$].

2-Fluoro-*N*-(4-(3-(pyridin-2-yl)-1,2,4-oxadiazol-5-yl)phenyl)benzamide (22e)

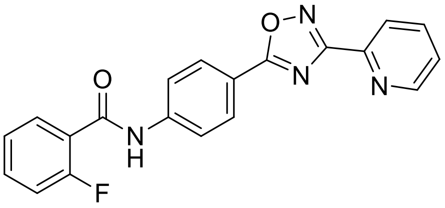

Prepared according to general procedure 4 from **21b** and 2-fluorobenzoyl chloride. Crude product was purified by column chromatography (SiO_2, $CHCl_3$/MeOH 49:1) followed by maceration (2-PrOH/hexane 1:2); white solid, 0.22 g, 96.9%.

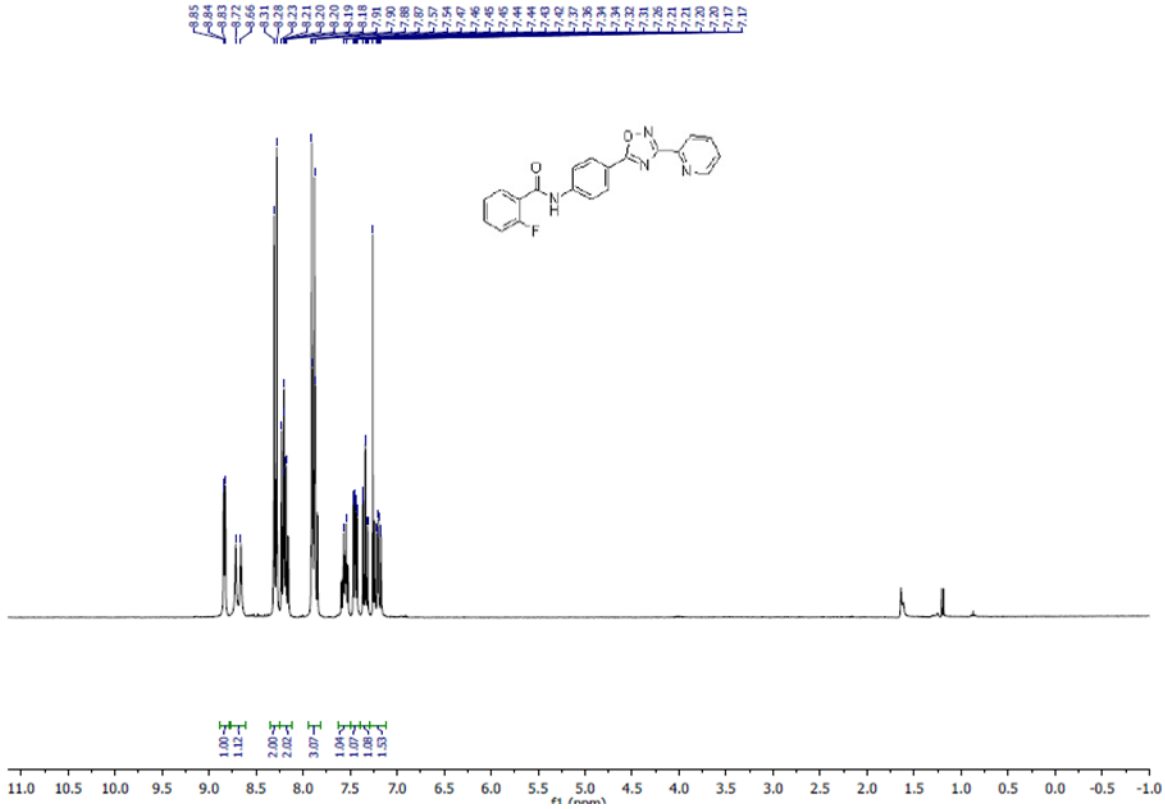

^{1}H NMR (300 MHz, $CDCl_3$), δ ppm: 8.84 (ddd, *J* = 4.8, 1.7 Hz and 0.9, 1H), 8.70 (d br., *J* = 16.4 Hz, 1H), 8.32-8.27 (m, 2H), 8.23-8.15 (m, 2H), 7.92-7.84 (m, 3H), 7.60-7.52 (m, 1H), 7.44 (ddd, *J* = 7.6, 4.8 and 1.2 Hz, 1H), 7.34 (td, *J* = 7.8 and 1.0 Hz, 1H), 7.20 (ddd, *J* = 12.5, 8.3 and 1.0 Hz, 1H);

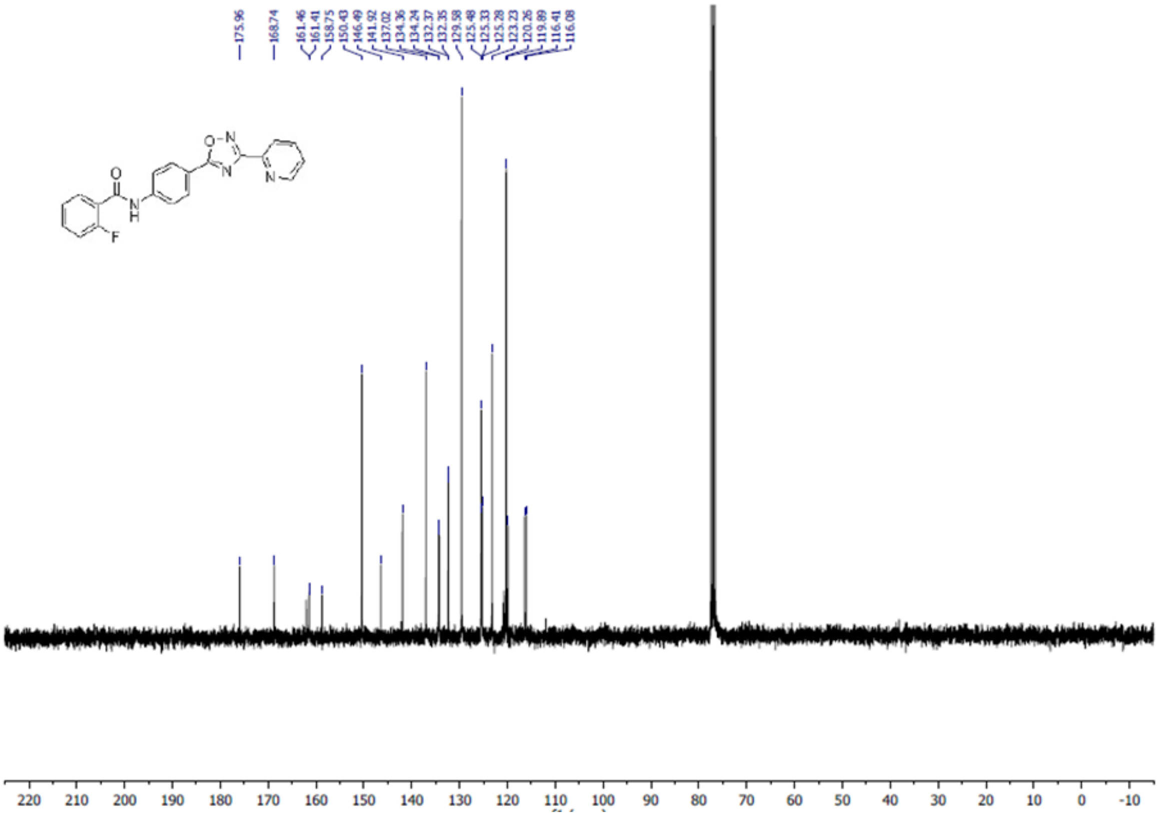

^{13}C NMR (75 MHz, $CDCl_3$), δ ppm: 176.0, 168.7, 161.4 (d, *J* = 3.5 Hz), 160.2 (d, *J* = 246.4 Hz), 150.4, 146.5, 141.9, 137.0, 134.3 (d, *J* = 9.5 Hz), 132.3 (d, *J* = 1.6 Hz), 129.6 (2C), 125.5, 125.3 (d, *J* = 3.2 Hz), 123.2, 120.7 (d, *J* = 11.0 Hz), 120.3 (2C), 119.9, 116.3 (d, *J* = 25.1 Hz); LC-MS (method B) R_T = 2.99 min (purity: 100%), *m/z* found: 360.8, calc.: 361.1 [M+H$^+$].

_N_-(2-Chlorophenyl)-4-cyanobenzene-1-sulfonamide (**25a**)

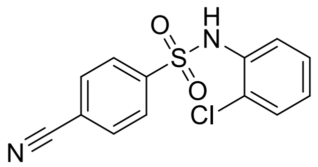

To a solution of 2-chloroaniline (**23a**) (1.90 g, 14.88 mmol, 1.2 eq) in pyridine (30 mL) 4-cyanobenzene-1-sulfonyl chloride (**24**) (2.50 g, 12.40 mmol, 1.0 eq) was added in one portion. After stirring in rt overnight reaction mixture was poured into water (100 mL). Precipitated solid was filtered off, dried, and purified by maceration (2-PrOH/hexane 1:2); white solid, 2.43 g, 66.9%. LC-MS (method A) R_T = 2.91 min (purity: 86.10%), *m/z* found: 292.0, calc.: 293.0 [M+H$^+$]. *The product was used in the next steps without further purification.*

4-Cyano-_N_-(2,4-difluorophenyl)benzene-1-sulfonamide (**25b**)

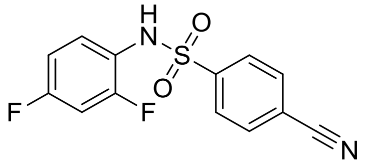

Prepared according to the procedure described for compound **25a** from 4-cyanobenzene-1-sulfonyl chloride and 2,4-difluoroaniline. Precipitated solid was filtered off and dried, resulting in pale yellow solid, 1.46 g, 80.2%. LC-MS (method A) R_T = 2.97 min (purity: 95.78%), *m/z* found: 294.7, calc.: 295.0 [M+H$^+$]. *The product was used directly to the next step.*

_N_-(3-Chloro-4-cyanophenyl)picolinamide (**25c**)

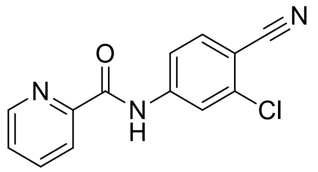

4-amino-2-chlorobenzonitrile (5.00 g, 32.77 mmol, 1.00 eq) was dissolved in 100 mL of THF. TEA (9.92 g, 13.68 mL, 98.31 mmol, 3.0 eq) was added dropwise followed by addition of 2-

pyridinecarbonyl chloride, hydrochloride (7.00 g, 39.32 mmol, 1.2 eq). Reaction mixture was stirred in rt overnight. Solvent was evaporated, 60 mL of water was added and resulted mixture was extracted with $CHCl_3$. Crude product was purified by column chromatography (SiO_2, $CHCl_3$/MeOH 49:1); pale yellow solid, 3.27 g, 38.7%. LC-MS (method A) R_T = 3.04 min (purity: 100%), *m/z* found: 257.9, calc.: 258.1 [$M+H^+$]. *The product was used directly to the next step.*

***N*-(4-Cyano-3-fluorophenyl)picolinamide** (**25d**)

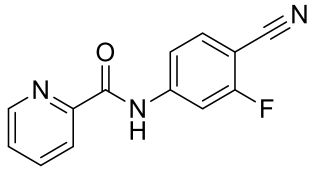

Prepared according to procedure described for **25c** from 4-amino-2-fluorobenzonitrile. Crude product was purified by column chromatography (SiO_2, $CHCl_3$/MeOH 49:1); pale yellow solid, 3.91 g, 88.3%. ^{1}H NMR (300 MHz, $CDCl_3$), δ ppm: 10.33 (s br. 1H, NH), 8.63 (ddd, *J* = 4.8 Hz, 1.6 and 0.8 Hz, 1H), 8.28 (d br., *J* = 7.8 Hz, 1H), 8.01-7.92 (m, 2H), 7.62-7.52 (m, 2H), 7.48 (dd, *J* = 8.6 and 1.9 Hz, 1H); ^{13}C NMR (75 MHz, $CDCl_3$), δ ppm: 164.0 (d, *J* = 257.1 Hz), 162.4, 148.5, 148.2, 143.6 (d, *J* = 11.3 Hz), 138.0, 133.9 (d, *J* = 1.7 Hz), 127.3, 122.7, 115.2 (d, *J* = 3.1 Hz), 114.3, 107.0 (d, *J* = 24.8 Hz), 96.0 (d, *J* = 15.6 Hz); LC-MS (method A) R_T = 2.84 min (purity: 98.79%), *m/z* found: 241.7, calc.: 242.1 [$M+H^+$].

4-(*N*-(2-Chlorophenyl)sulfamoyl)-N'-hydroxybenzimidamide (**26a**)

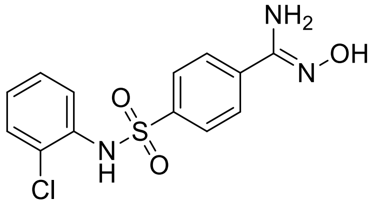

Prepared from **25a** according to general procedure 1. After extraction and evaporation of solvent $CHCl_3$ (20 mL) was added and precipitated white crystals were filtered off and used directly in next steps, 2.33 g, 86.3%. ^1H NMR (300 MHz, DMSO-d_6), δ ppm: 10.04 (s, 1H, NH), 9.94 (s, 1H, OH), 7.81 (d br., *J* = 8.5 Hz, 2H), 7.68 (d, *J* = 8.5 Hz, 2H), 7.39 (dd, *J* = 7.7 and 1.1 Hz, 1H), 7.30-7.16 (m, 3H), 5.95 (s br. 2H, NH_2); ^{13}C NMR (75 MHz, DMSO-d_6), δ ppm: 150.1, 140.7, 137.8, 133.9, 130.4, 129.6, 128.2, 128.1, 127.9, 127.0 (2C), 126.3 (2C); LC-MS (method A) R_T = 1.76 min (purity: 97.80%), *m/z* found: 326.03, calc.: 326.0 [M+H$^+$].

4-(*N*-(2,4-Difluorophenyl)sulfamoyl)-N'-hydroxybenzimidamide (26b)

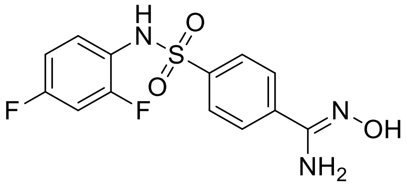

Prepared from **25b** according to general procedure 1. Acidification of water solution of reaction mixture resulted precipitation of yellow solid of benzamide by-product (0.65 g). Crude product was purified by maceration (2-PrOH); white crystals, 0.40 g, 25%. ^1H NMR (300 MHz, DMSO-d_6), δ ppm: 10.13 (s, 1H, NH), 9.93 (s, 1H, OH), 7.81 (d br., *J* = 8.6, 2H), 7.64 (d, *J* = 8.6 Hz, 2H), 7.25-7.17 (m, 2H), 7.06-6.99 (m, 1H), 5.92 (d br., *J* = 3.3 Hz, 2H, NH_2); ^{13}C NMR (75 MHz, DMSO-d_6), δ ppm: 160.6 (dd, *J* = 246.8 and 11.2 Hz), 156.9 (dd, *J* = 250.8 and 12.9 Hz), 150.0 (q, *J* = 2.7 Hz), 140.1, 137.9 (d, *J* = 2.8 Hz), 129.3 (dd, *J* = 10.1 and 1.9 Hz), 126.9 (2C), 126.3 (2C), 121.0 (dd, *J* = 13.0 and 3.6 Hz), 112.2 (dd, *J* = 22.4 and 3.9 Hz), 105.1 (dd, *J* = 26.8 and 24.3 Hz); LC-MS (method A) R_T = 1.61 min (purity: 100%), *m/z* found: 327.9, calc.: 328.0 [M+H$^+$].

***N*-(3-Chloro-4-(N'-hydroxycarbamimidoyl)phenyl)picolinamide (26c)**

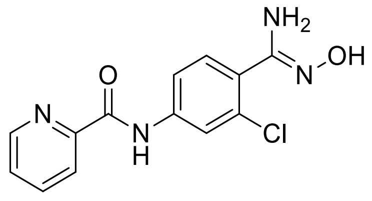

Prepared from **25c** according to general procedure 1, method A. Reaction gave mixture of two products: N-(4-carbamoyl-3-chlorophenyl)picolinamide (1.90 g, 52.0%) isolated after extraction of acidic water layer with AcOEt and N-(3-chloro-4-(N'-hydroxycarbamimidoyl) phenyl)picolinamide isolated after extraction of alkalized water layer with AcOEt. Crude final product was purified by maceration (2-PrOH/hexane 1:2); white solid, 0.51 g, 13.9%. ^{1}H NMR (300 MHz, $CDCl_3$+ CD_3OD), δ ppm: 8.54 (ddd, *J* = 3.1 Hz, 1H), 8.15 (dt, *J* =, 7.8 and 1.0 Hz, 1H), 7.95 (d, *J* = 2.1 Hz, 1H), 7.85 (td, *J* = 7.7 and 1.7 Hz, 1H), 7.53 (dd, *J* = 8.4 and 2.1 Hz, 1H), 7.45 (ddd, *J* = 7.7, 4.8 and 1.2 Hz, 1H), 7.37 (d, *J* = 8.4 Hz, 1H), 3.70 (s br. 4H, NH_2, OH, NH); ^{13}C NMR (75 MHz, $CDCl_3$+ CD_3OD), δ ppm: 162.5, 151.8, 148.9, 148.1, 139.5, 137.9, 133.3, 131.4, 127.5, 126.9, 122.5, 120.6, 117.7; LC-MS (method A) R_T = 0.48-0.83 min (purity: 100%), *m/z* found: 291.96, calc.: 291.1 [M+H$^+$].

***N*-(3-Fluoro-4-(N'-hydroxycarbamimidoyl)phenyl)picolinamide (26d)**

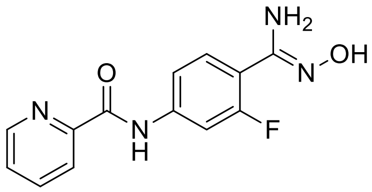

Prepared according to general procedure 1 from **25d**. Isolated product was purified by maceration (2-PrOH/hexane 1:2); white solid, 1.12 g, 52.6%. ^{1}H NMR (300 MHz, $CDCl_3$ + DMSO-d_6), δ ppm: 10.37 (s br. 1H, NH), 9.57 (s br. 1H, OH), 8.59 (ddd, *J* = 4.8, 1.6 and 0.9 Hz, 1H), 8.15 (dt, *J* = 7.8 and 1.0 Hz, 1H), 7.90 (td, *J* = 7.8 and 1.7 Hz, 1H), 7.85 (dd, *J* = 13.7 and 2.1 Hz, 1H), 7.57 (t, *J* = 8.4 Hz, 1H), 7.49 (ddd, *J* = 7.6, 4.8 and 1.2 Hz, 1H), 7.41 (dd, *J* = 8.5 and 2.0 Hz, 1H), 5.29 (s br. 2H, NH_2); ^{13}C NMR (75 MHz, $CDCl_3$ + DMSO-d_6), δ ppm:

161.9, 159.5 (d, J = 247.6 Hz), 148.7, 148.1 (d, J = 2.4 Hz), 147.6, 139.6 (d, J = 11.7 Hz), 137.3, 129.1 (d, J = 4.7 Hz), 126.3, 121.9, 115.8 (d, J = 11.9 Hz), 114.7 (d, J = 2.9 Hz), 106.8 (d, J = 28.3 Hz); LC-MS (method A) R_T = 0.71 min (purity: 95.0%), *m/z* found: 275.0, calc.: 275.1 [M+H$^+$].

***N*-(2-Chlorophenyl)-4-(5-phenyl-1,2,4-oxadiazol-3-yl)benzenesulfonamide (27a)**

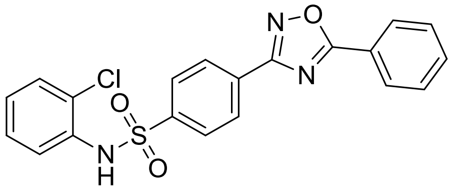

Prepared according to general procedure 2 from **26a** and benzoyl chloride. Crude product was purified by column chromatography (Al_2O_3, $CHCl_3$/hexane 3:1) followed by maceration (2-PrOH/hexane 1:3); white solid, 0.16 g, 50.6%.

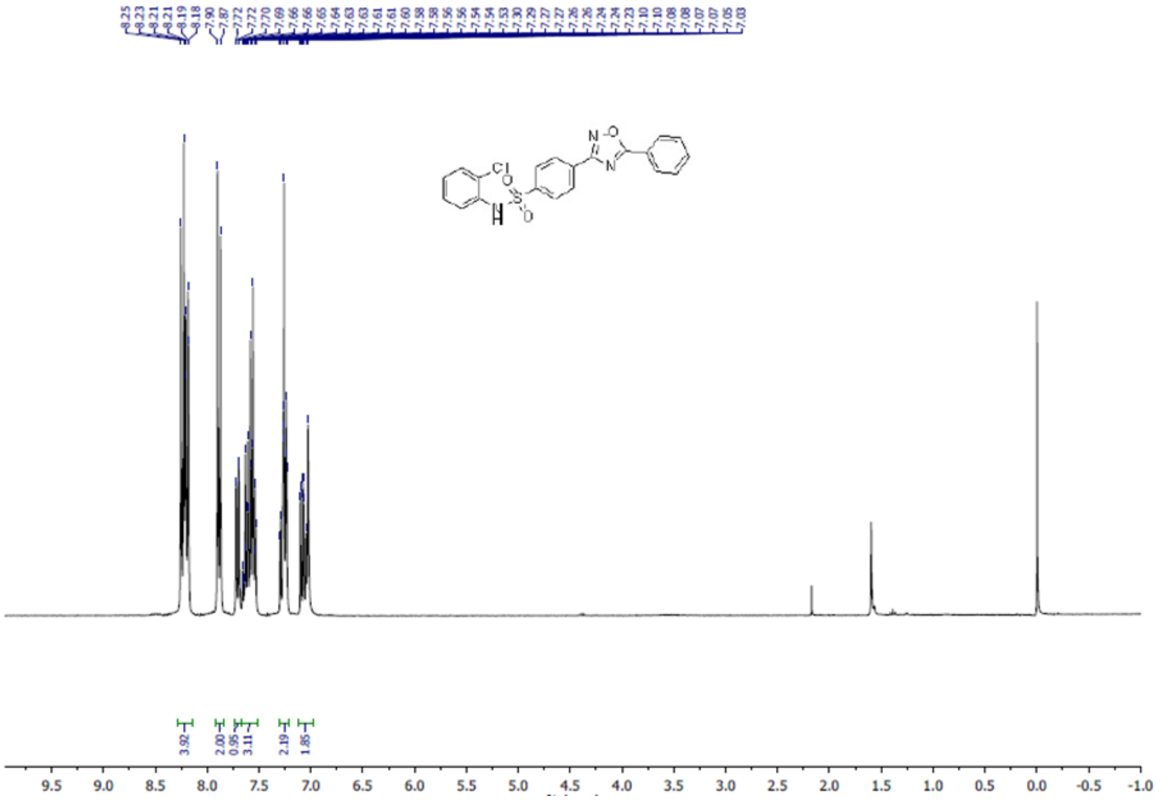

^{1}H NMR (300 MHz, $CDCl_3$), δ ppm: 8.25-8.18 (m, 4H), 7.91-7.87 (m, 2H), 7.71 (dd, J = 8.1 and 1.4 Hz, 1H), 7.66-7.53 (m, 3H), 7.30-7.23 (m, 2H), 7.10-7.03 (m, 2H);

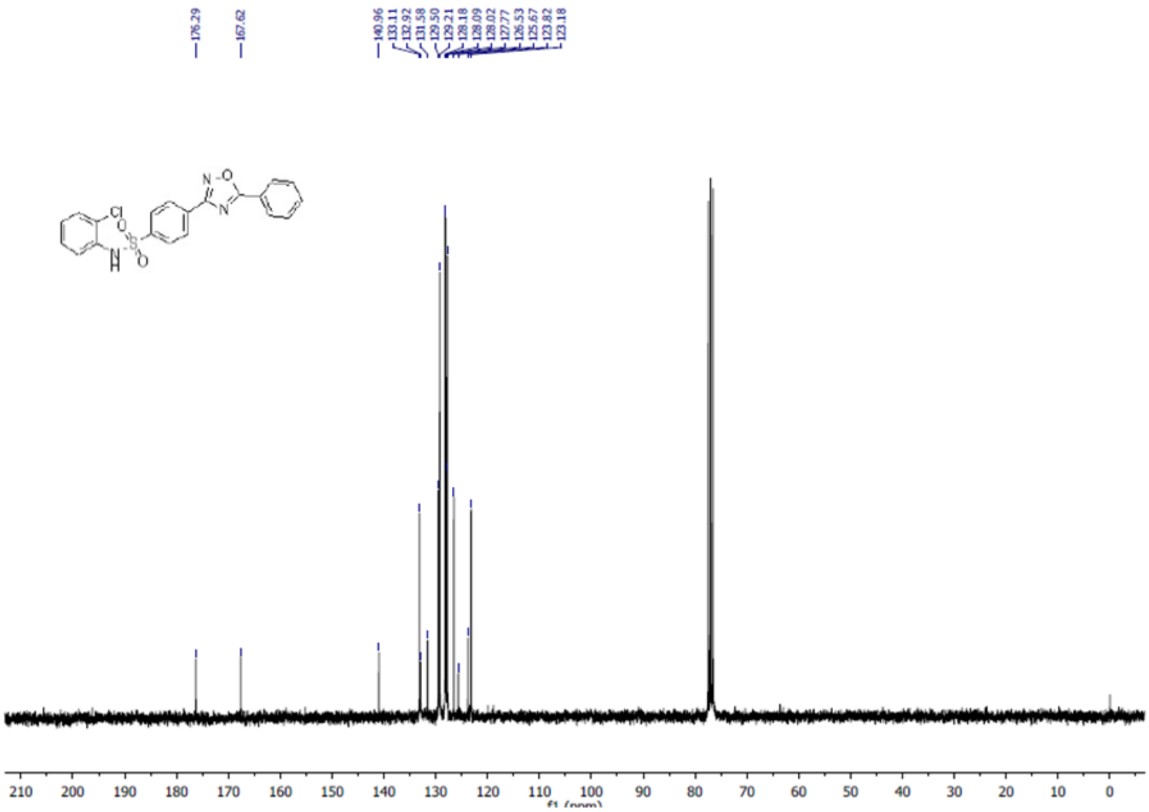

^{13}C NMR (75 MHz, $CDCl_3$), δ ppm: 176.3, 167.6, 141.0, 133.1, 132.9, 131.6, 129.5, 129.2 (2C), 128.2 (2C), 128.1 (2C), 128.0, 127.8 (2C), 126.5, 125.7, 123.8, 123.2; LC-MS (method A) R_T = 3.71 min (purity: 100%), *m/z* found: 412.08, calc.: 412.0 [$M+H^+$].

N-(2-Chlorophenyl)-4-(5-(pyridin-2-yl)-1,2,4-oxadiazol-3-yl)benzenesulfonamide (**27b**)

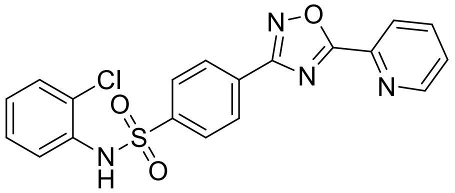

Prepared according to general procedure 2 from **26a** and 2-pyridinecarbonyl chloride, hydrochloride. Crude product was purified by column chromatography (SiO_2, $CHCl_3$/MeOH 99:1 than Al_2O_3, $CHCl_3$/hexane 3:1) followed by maceration (2-PrOH/hexane 1:3); white solid, 0.15 g, 47.4%.

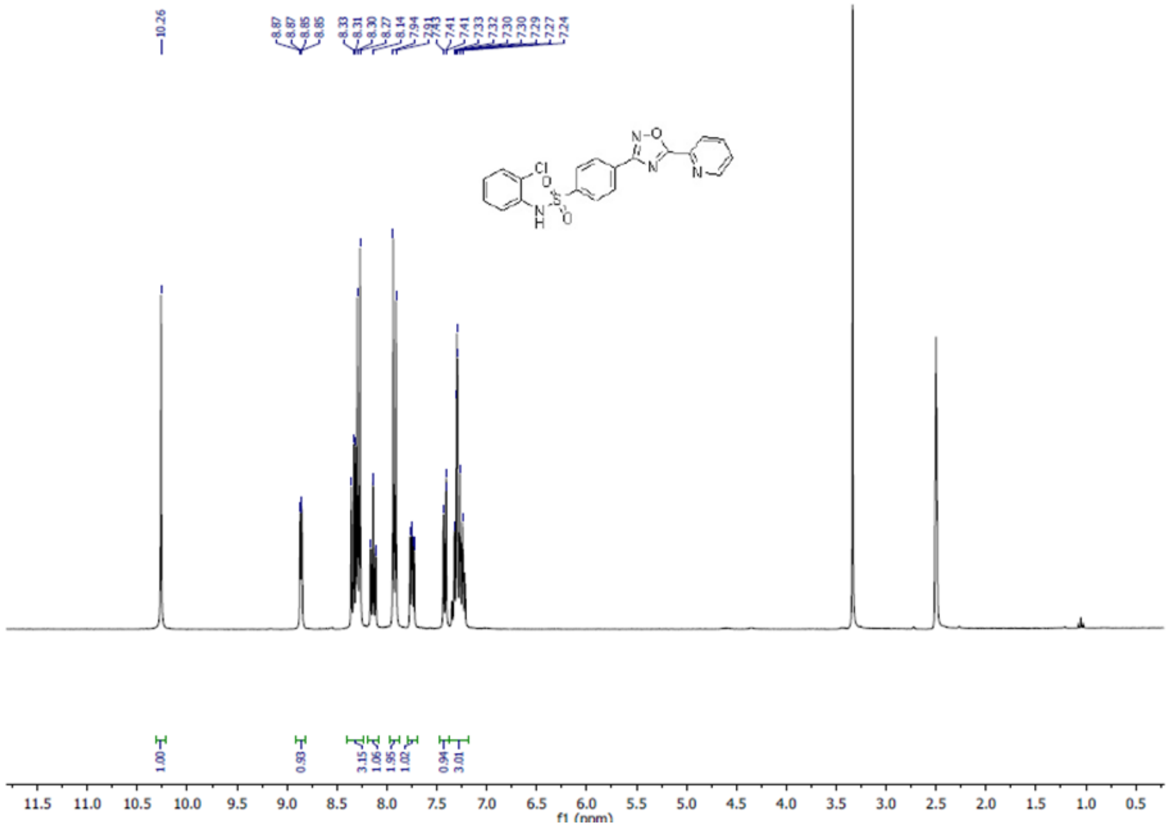

^{1}H NMR (300 MHz, DMSO-d_6), δ ppm: 10.26 (s br., 1H, NH), 8.86 (ddd, J = 4.7, 1.4 and 0.6 Hz, 1H), 8.36-8.27 (m, 3H), 8.14 (td, J = 7.8 and 1.8 Hz, 1H), 7.93 (d br., J = 8.5 Hz, 2H), 7.75 (ddd, J = 7.7, 4.8 and 1.1 Hz, 1H), 7.41 (dd, J = 7.8 and 1.2 Hz, 1H), 7.35-7.21 (m, 3H);

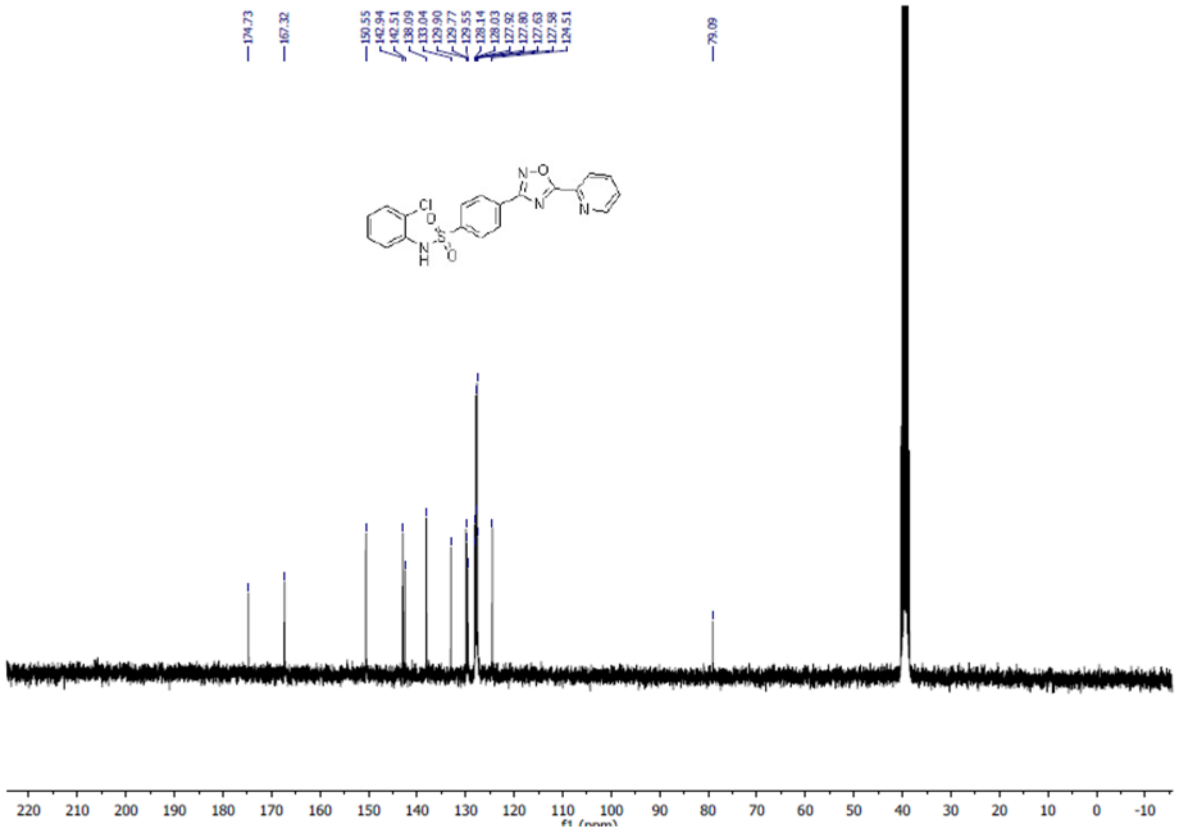

^{13}C NMR (75 MHz, DMSO-d$_6$), δ ppm: 174.7, 167.3, 150.6, 142.9, 142.5, 138.1, 133.0, 129.9, 129.8, 129.6, 128.1, 128.0, 127.9 (2C), 127.8, 127.6 (2C), 127.5, 124.5; LC-MS (method A) R_T=3.15 min (purity: 100%), *m/z* found: 413.11, calc.: 413.1 [M+H$^+$].

_N_-(2,4-Difluorophenyl)-4-(5-(pyridin-2-yl)-1,2,4-oxadiazol-3-yl)benzenesulfonamide (27c)

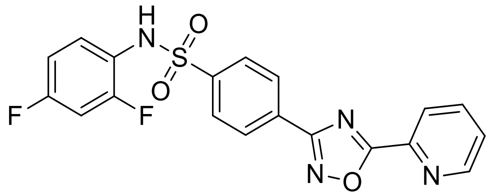

Prepared according to general procedure 2 from **26b** and 2-pyridinecarbonyl chloride, hydrochloride. Crude product was purified by column chromatography (SiO_2, $CHCl_3$/MeOH 99:1) followed by maceration (2-PrOH/hexane 1:2); white solid, 0.13 g, 52.0%.

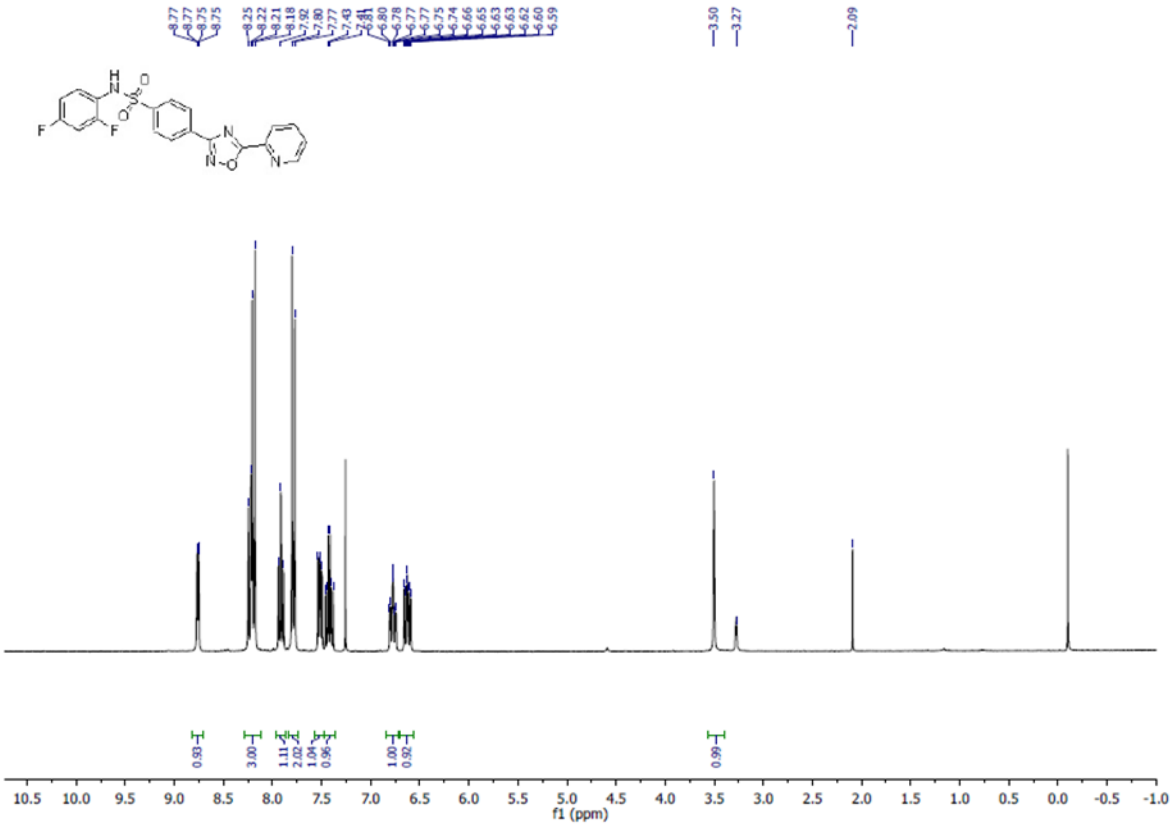

^{1}H NMR (300 MHz, $CDCl_3$ + CD_3OD), δ ppm: 8.76 (ddd, *J* = 4.8, 1.6 and 0.9 Hz, 1H), 8.24 (dt, *J* = 7.9 and 0.9 Hz, 1H), 8.21-8.17 (m, 2H), 7.93 (td, *J* = 7.8 and 1.7 Hz, 1H), 7.81-7.77 (m,

2H), 7.52 (ddd, *J* = 7.7, 4.8 and 1.1 Hz, 1H), 7.42 (td, *J* = 8.9 and 5.9 Hz, 1H), 6.81-6.74 (m, 1H), 6.63 (ddd, *J* = 11.0, 8.3 and 2.8 Hz, 1H), 3.50 (s br., 1H, NH);

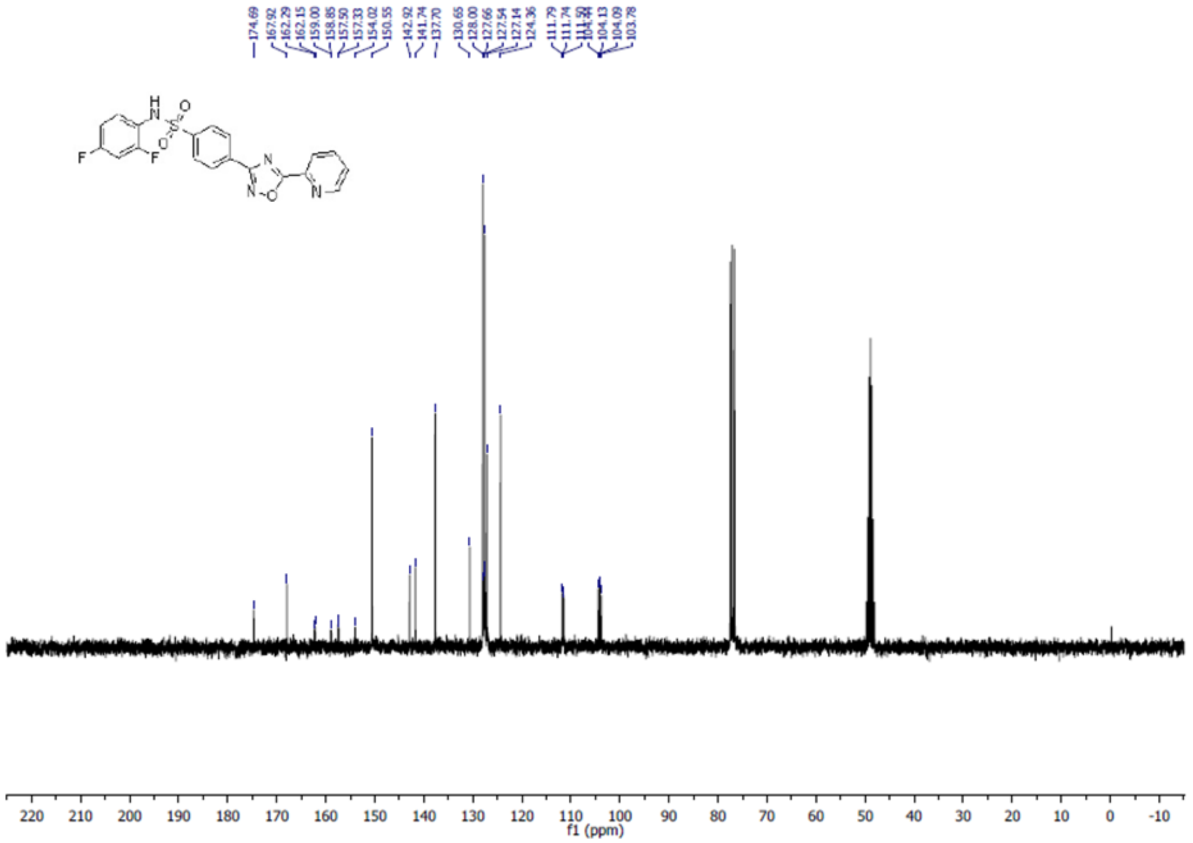

^{13}C NMR (75 MHz, $CDCl_3$ + CD_3OD), δ ppm: 174.7, 167.9, 160.6 (dd, *J* = 248.8 and 11.0 Hz), 155.8 (dd, *J* = 249.8 and 12.2 Hz), 150.6, 142.9, 141.7, 137.7, 130.7, 128.1 (3C), 127.7 (dd, *J* = 9.8 and 1.9 Hz), 127.5 (2C), 127.1, 124.4, 111.6 (dd, *J* = 22.3 and 3.8 Hz), 104.1 (dd, *J* = 26.6 and 23.8 Hz); LC-MS (method A) R_T = 3.05 min (purity: 100%), *m/z* found: 415.2, calc.: 415.1 [M+H$^+$].

***N*-(4-(5-(2-Chlorophenyl)-1,2,4-oxadiazol-3-yl)phenyl)nicotinamide** (**28**)

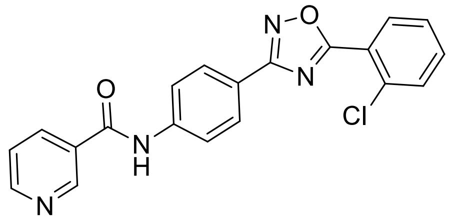

Prepared from **16a** and nicotinoyl chloride, hydrochloride according to general procedure 4. Crude product was purified by maceration (2-PrOH/hexane 1:2); white solid, 0.24 g, 85.7%.

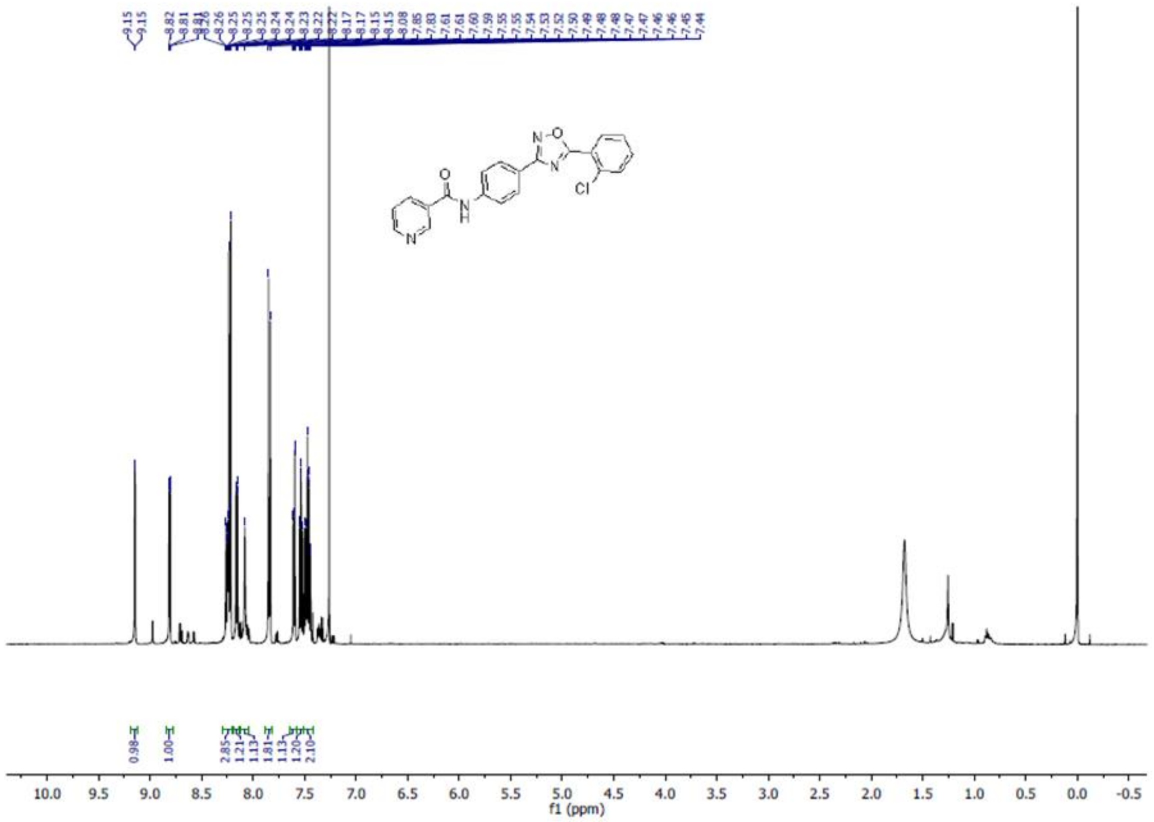

^{1}H NMR (300 MHz, $CDCl_3$), δ ppm: 9.15 (d, *J* = 1.7 Hz, 1H), 8.81 (dd, *J* = 4.8, 1.7 Hz, 1H), 8.29-8.20 (m, 3H), 8.16 (dd, *J* = 7.8, 1.5 Hz, 1H), 8.08 (s br. 1H), 7.84 (d, *J* = 8.8 Hz, 2H), 7.61 (dd, *J* = 8.1, 1.0 Hz, 1H), 7.56-7.51 (m, 1H), 7.50-7.44 (m, 2H);

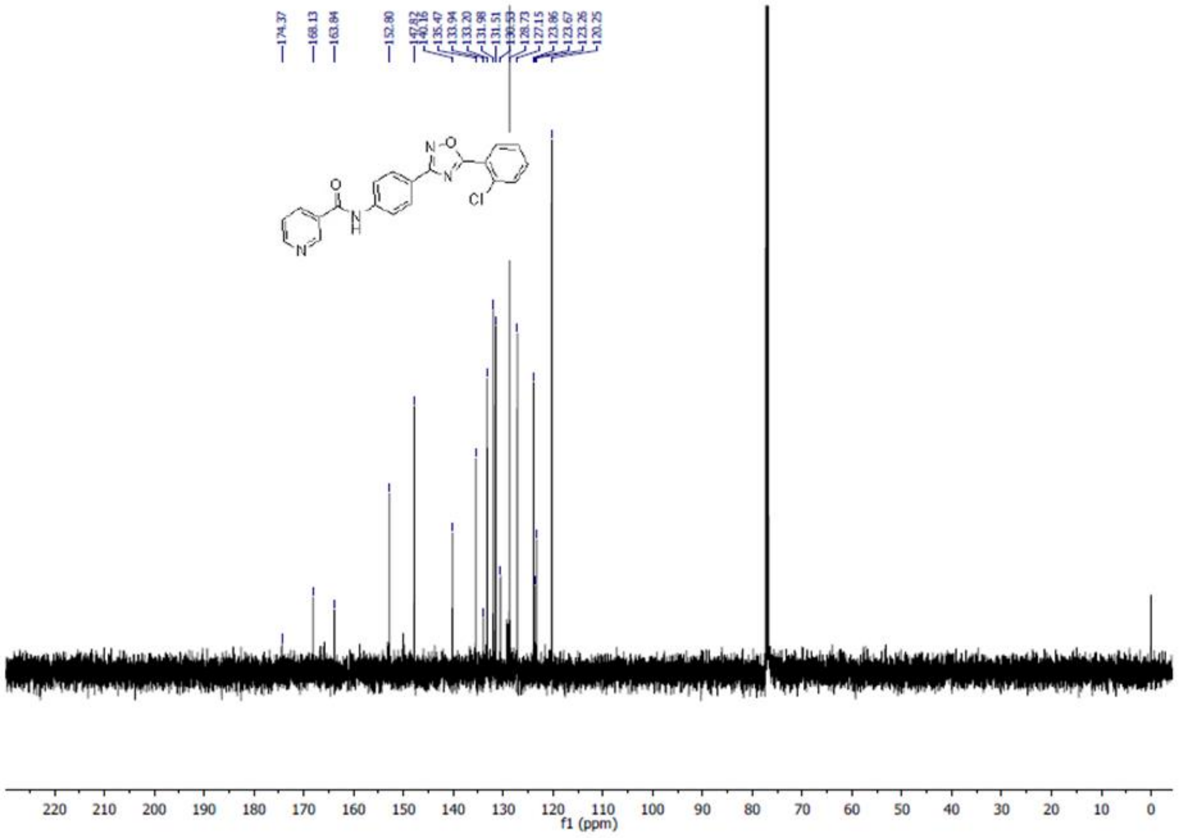

^{13}C NMR (75 MHz, $CDCl_3$), δ ppm: 174.4, 168.1, 163.8, 152.8, 147.8, 140.2, 135.5, 133.9, 133.2, 132.0, 131.5, 130.5, 128.7 (2C), 127.2, 123.9, 123.7, 123.3, 120.3 (2C); LC-MS (method B) R_T = 3.20 min (purity ≥ 95%), *m/z* found: 377.4, calc.: 377.073 [M+H$^+$], HRMS: 377.091, mp=161-163°C

***N*-(4-(5-(2-Chlorophenyl)-1,2,4-oxadiazol-3-yl)phenyl)isonicotinamide (29)**

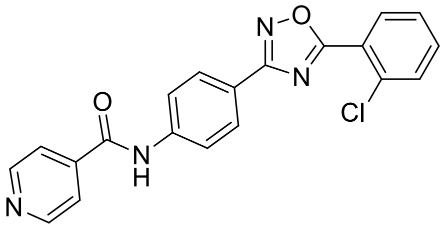

Prepared from **16a** and isonicotinoyl chloride, hydrochloride according to general procedure 4. Crude product was purified by maceration (2-PrOH/hexane 1:2); white solid, 0.26 g, 92.9%.

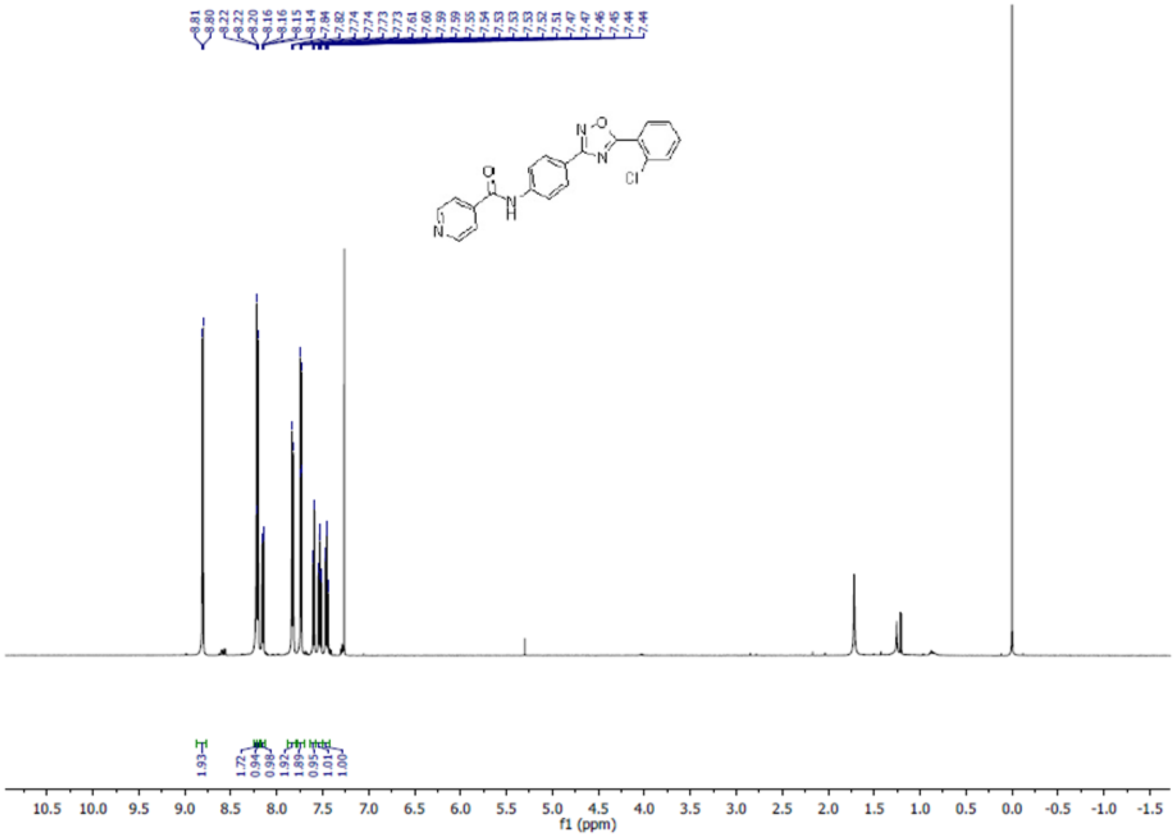

^{1}H NMR (300 MHz, $CDCl_3$), δ ppm: 8.82-8.79 (m, 2H), 8.23-8.19 (m, 3H), 8.16 (dd, *J* = 7.7, 1.6 Hz, 1H), 7.84-7.81 (m, 2H), 7.75-7.72 (m, 2H), 7.60 (dd, *J* = 8.1, 1.0 Hz, 1H), 7.55-7.51 (m, 1H), 7.47-7.43 (m, 1H);

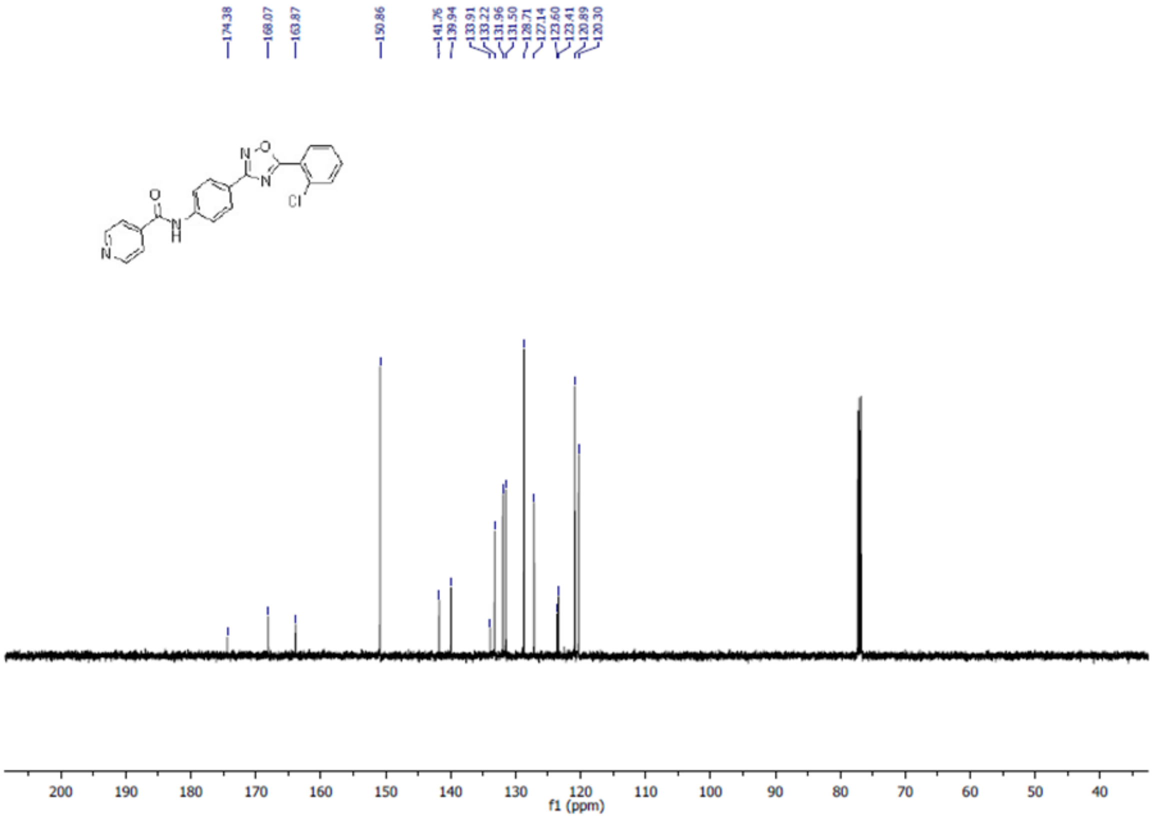

^{13}C NMR (75 MHz, $CDCl_3$), δ ppm: 174.4, 168.1, 163.4, 150.9 (2C), 141.8, 140.0, 133.9, 133.2, 132.0, 131.5, 128.7 (2C), 127.1, 123.6, 123.4, 120.9 (2C), 120.3 (2C); LC-MS (method B) R_T = 3.18 min (purity: 100%), *m/z* found: 377.03, calc. 377.073 [$M+H^+$], HRMS: 377.089, mp=184-186°C.

***N*-(4-(5-(2-Chlorophenyl)-1,2,4-oxadiazol-3-yl)phenyl)benzamide** (**30**)

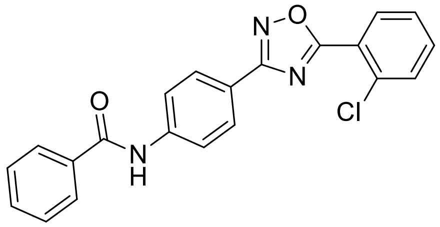

Prepared from **16a** and benzoyl chloride, according to general procedure 4. Crude product was purified by column chromatography (SiO_2, $CHCl_3$/MeOH) followed by maceration (2-PrOH/hexane 1:2); white solid, 0.25 g, 90.6%.

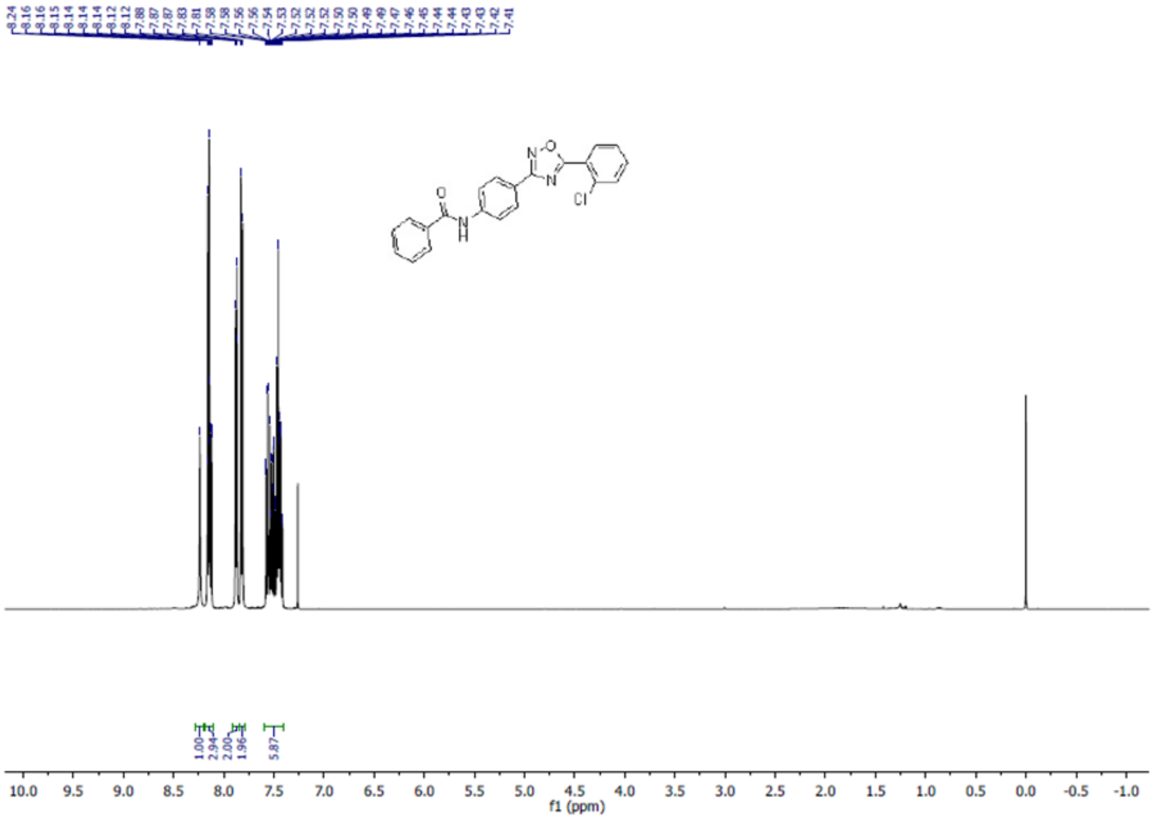

^{1}H NMR (300 MHz, $CDCl_3$), δ ppm: 8.24 (s br, 1H), 8.19-8.10 (m, 3H), 7.91-7.85 (m, 2H), 7.82 (d, J = 8.7 Hz, 2H), 7.60-7.40 (m, 6H);

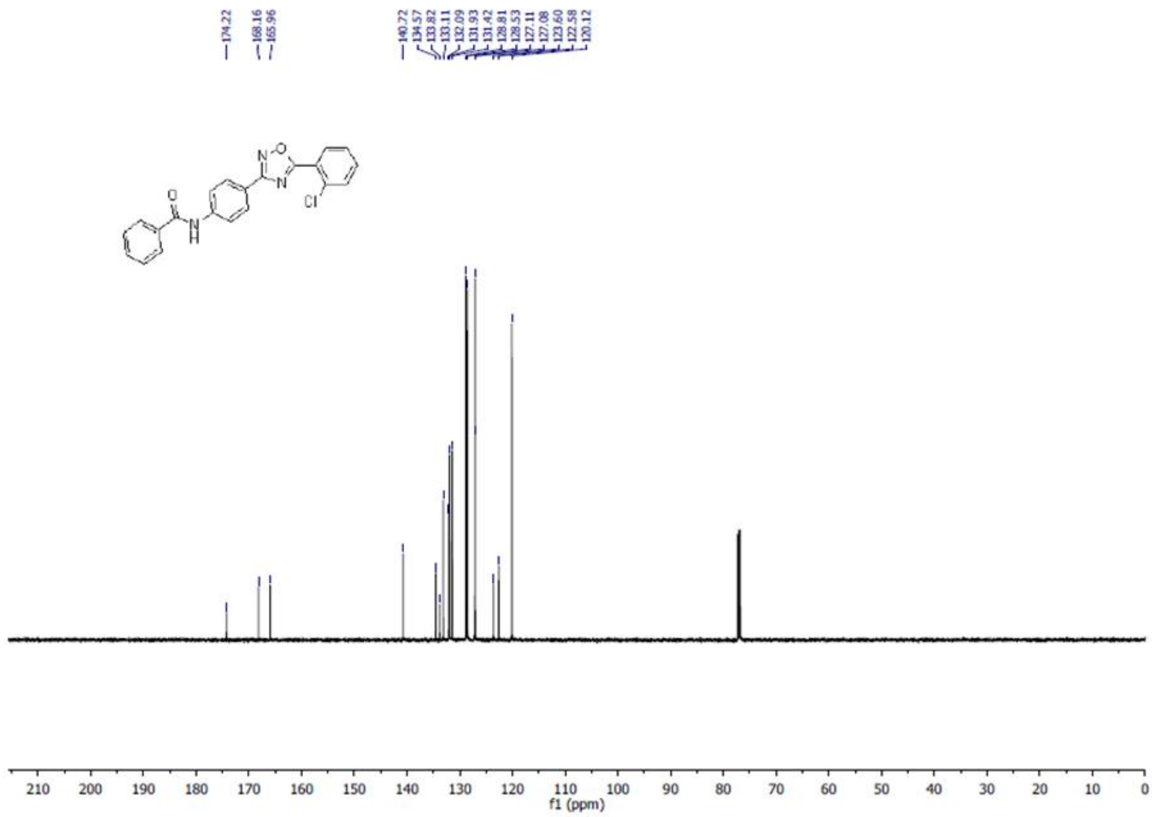

^{13}C NMR (75 MHz, $CDCl_3$), δ ppm: 174.2, 168.2, 166.0, 140.7, 134.6, 133.8, 133.1, 132.1, 131.9, 131.4, 128.8 (2C), 128.5 (2C), 127.10 (2C), 127.08, 123.6, 122.6, 120.1 (2C); LC-MS (method B) R_T = 3.68 min (purity 100%), *m/z* found: 376.1, calc.: 376.077 [M+H^+], HRMS: 376.084, mp=164-165°C.

***N*-(4-(5-(2-Chlorophenyl)-1,2,4-oxadiazol-3-yl)phenyl)-6-fluoropicolinamide** (**31**)

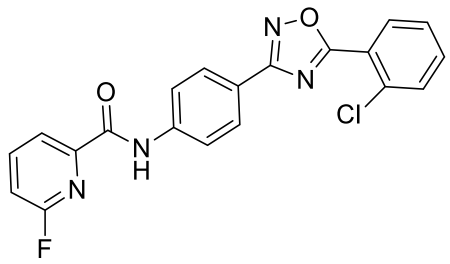

Prepared according to general procedure 5 from **16a** and 6-fluoropyridine-2-carboxylic acid. Precipitated solid was filtered directly from the reaction mixture and washed with MeCN to yield pure product; white solid, 0.19 g, 87.2%.

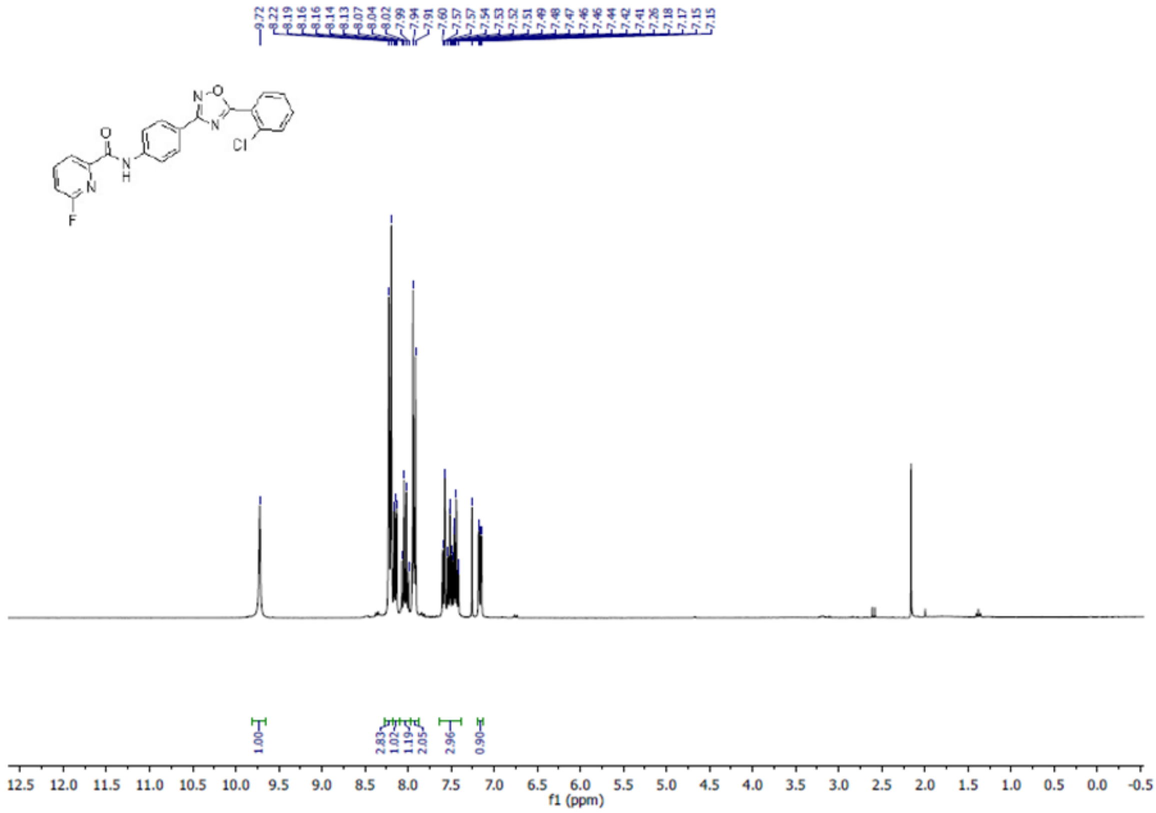

^{1}H NMR (300 MHz, $CDCl_3$), δ ppm: 9.72 (s, 1H), 8.25-8.18 (m, 3H), 8.15 (dd, *J* = 7.8, 1.8 Hz, 1H), 8.03 (q, *J* = 7.6 Hz, 1H), 7.96-7.93 (m, 2H), 7.61-7.41 (m, 3H), 7.16 (dd, J = 8.2, 2.4 Hz, 1H) (*singnal derived from residual MeCN presented on ^{1}H NMR spectra*)

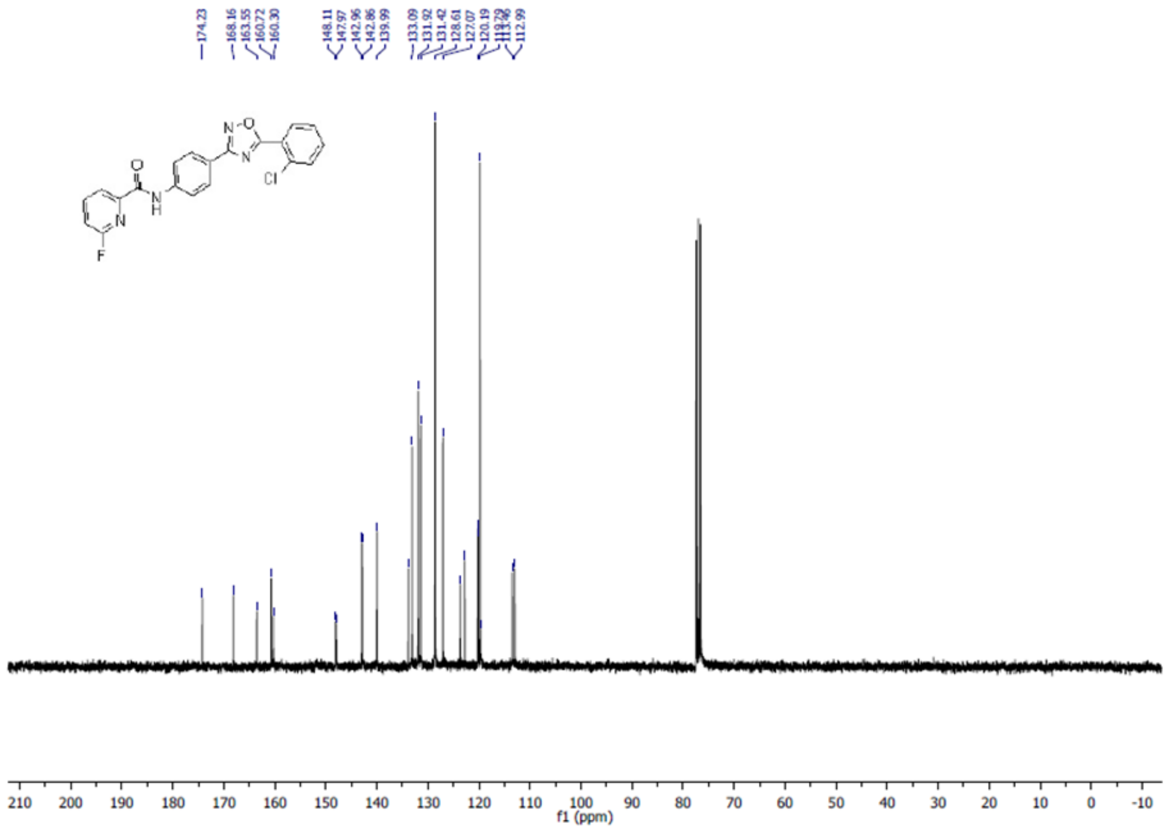

^{13}C NMR (75 MHz, $CDCl_3$), δ ppm: 174.2, 168.2, 161.9 (d, *J* = 244.2 Hz), 160.7, 148.1 (d, *J* = 10.7 Hz), 142.9 (d, *J* = 7.5 Hz), 140.0, 133.9, 133.1, 131.9, 131.4, 128.6 (2C), 127.1, 123.7, 122.8, 120.2 (d, *J* =3.8 Hz), 119.8 (2C), 113.2 (d, *J* = 35.5 Hz); LC-MS (method A) R_T = 3.86 min (purity: 98.20%), *m/z* found: 395.2, calc.: 395.064 [M+H$^+$], HRMS: 395.070, mp=185-187°C.

6-Chloro-*N*-(4-(5-(2-chlorophenyl)-1,2,4-oxadiazol-3-yl)phenyl)picolinamide (**32**)

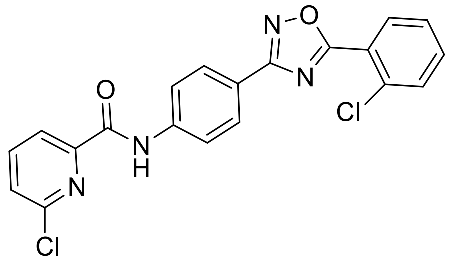

Prepared from **16a** and 6-chloropyridine-2-carboxylic acid according to general procedure 5. Precipitated solid was filtered directly from the reaction mixture and washed with MeCN to yield pure product; white solid, 0.16 g, 70.5%.

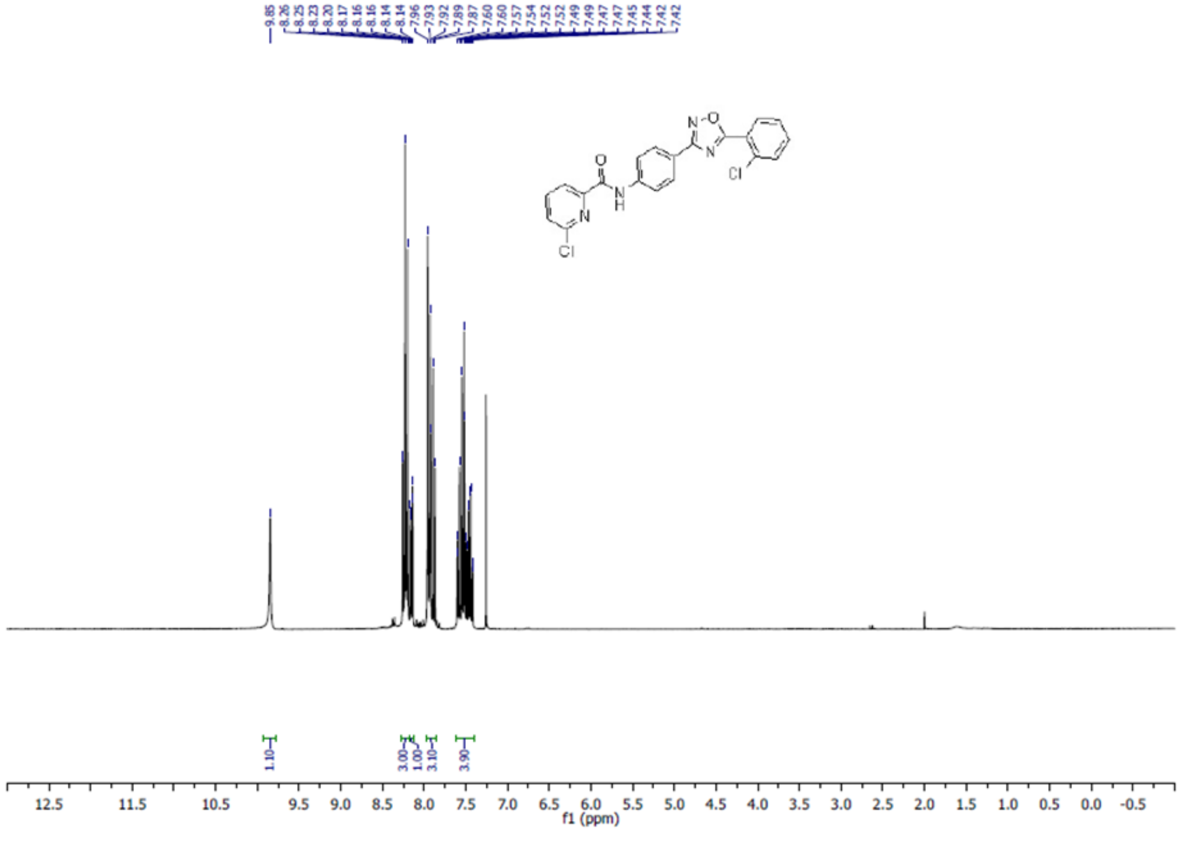

^{1}H NMR (300 MHz, $CDCl_3$), δ ppm: 9.85 (s, 1H), 8.26-8.19 (m, 3H), 8.15 (dd, *J* = 7.4, 1.8 Hz, 1H), 7.96-7.87 (m, 3H), 7.61-7.41 (m, 4H);

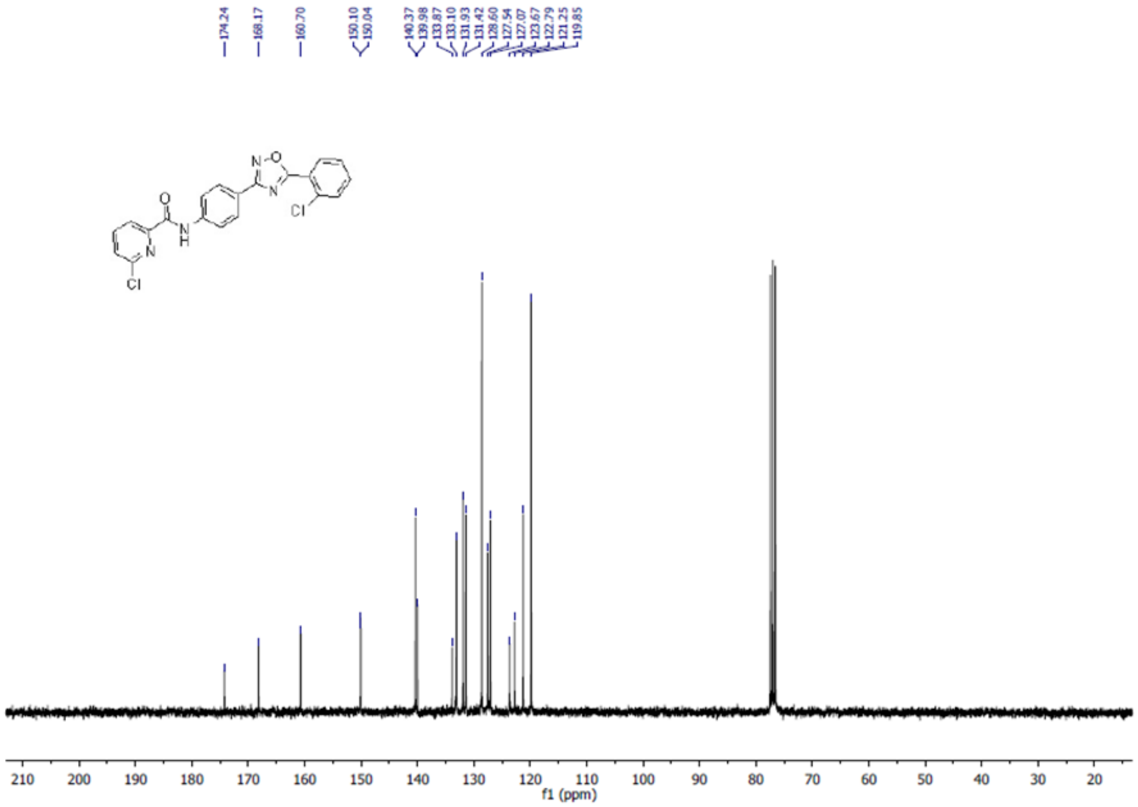

^{13}C NMR (75 MHz, $CDCl_3$), δ ppm: 174.2, 168.2, 160.7, 150.1, 150.0, 140.4, 140.0, 133.9, 133.1, 131.9, 131.4, 128.6 (2C), 127.5, 127.1, 123.7, 122.8, 121.3, 119.9 (2C); LC-MS (method A) R_T = 3.99 min (purity: 100%), *m/z* found: 411.69, calc. 411.034 [$M+H^+$], HRMS: 411.054, mp=180-182°C

***N*-(3-Methoxy-4-(5-phenyl-1,2,4-oxadiazol-3-yl)phenyl)picolinamide** (**33**)

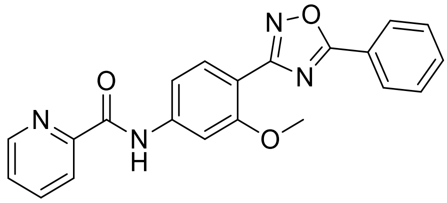

Prepared from **16d** and 2-pyridinecarbonyl chloride, hydrochloride according to general procedure 4. Crude product was purified by column chromatography (SiO_2, $CHCl_3$/hexane 2:1) followed by maceration (2-PrOH/hexane 1:2); white solid, 0.15 g, 71.4%.

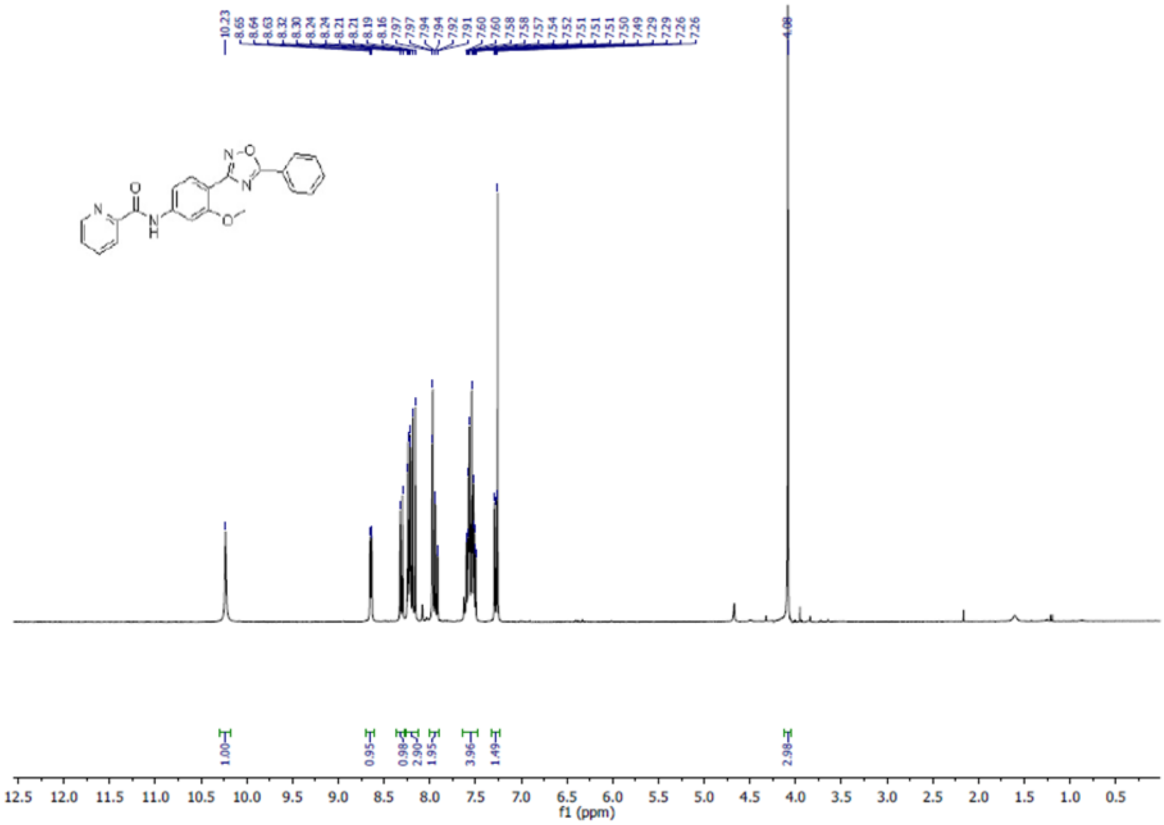

^{1}H NMR (300 MHz, $CDCl_3$), δ ppm: 10.23 (s br. 1H), 8.66-8.62 (m, 1H), 8.31 (dt, J = 7.9, 1.0 Hz, 1H), 8.24-8.16 (m, 3H), 7.97-7.91 (m, 2H), 7.60-7.49 (m, 4H), 7.32-7.24 (m, 1H), 4.09 (s, 3H);

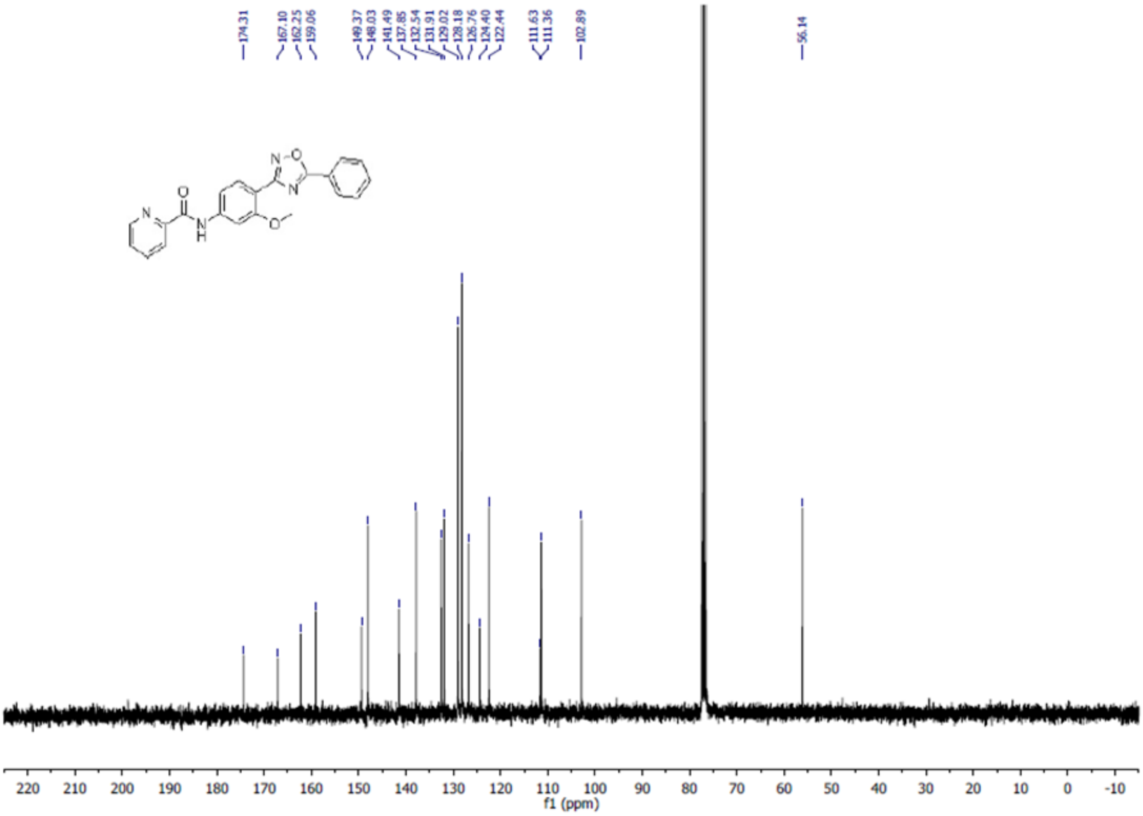

^{13}C NMR (75 MHz, $CDCl_3$), δ ppm: 174.3, 167.1, 162.3, 159.1, 149.4, 148.0, 141.5, 137.9, 132.5, 131.9, 129.0 (2C), 128.2 (2C), 126.8, 124.4, 122.4, 111.6, 111.4, 102.9, 56.1; LC-MS (method A) R_T = 3.43 min (purity: 98.27%), *m/z* found: 373.09, calc.: 373.122 [M+H$^+$], HRMS: 373.123, mp=150-151˚C

***N*-(4-(5-(2-Chlorophenyl)-1,2,4-oxadiazol-3-yl)-3-methoxyphenyl)picolinamide** (**34**)

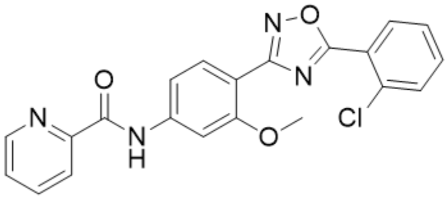

Prepared from **16e** and 2-pyridinecarbonyl chloride, hydrochloride according to general procedure 4. Crude product was purified by column chromatography (SiO_2, gradient from $CHCl_3$/hexane 2:1 to $CHCl_3$/MeOH 99:1) followed by maceration (2-PrOH/hexane 1:2); white solid, 0.113 g, 56.5%.

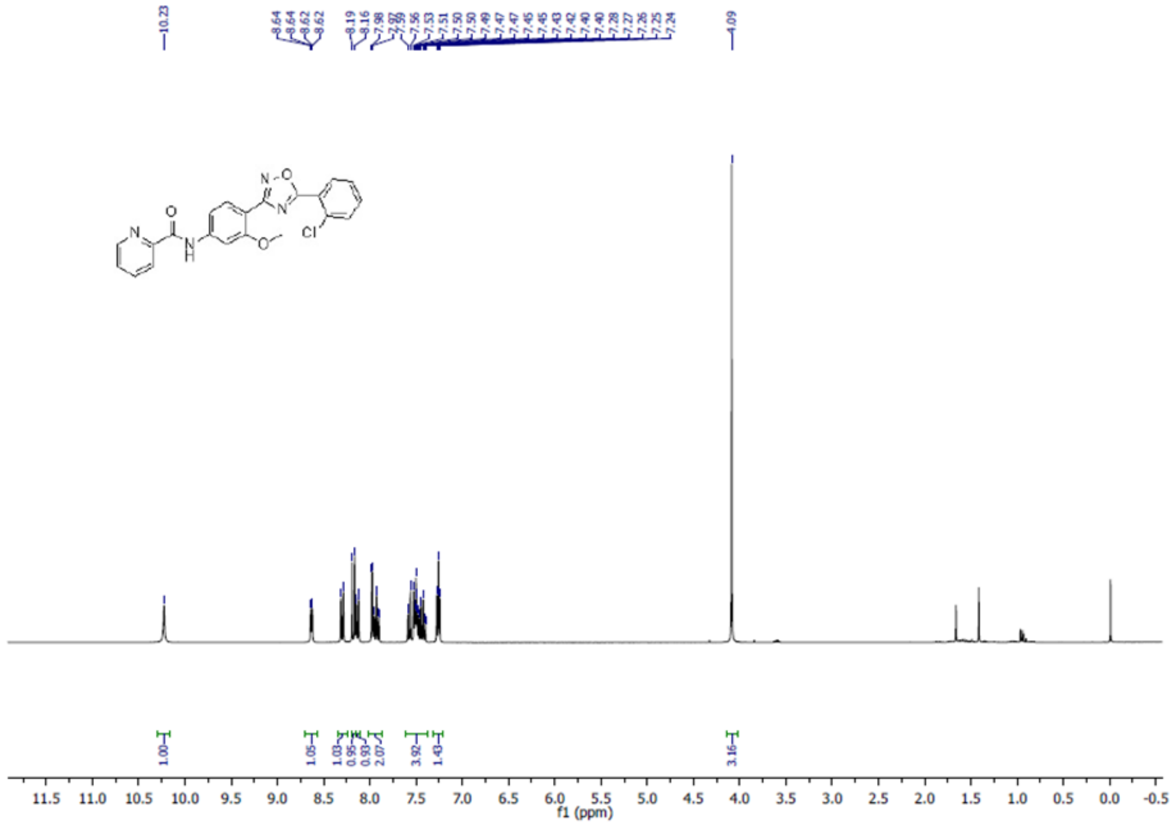

^{1}H NMR (300 MHz, $CDCl_3$), δ ppm: 10.23 (s br. 1H), 8.65-8.61 (m, 1H), 8.30 (d, *J* = 7.8 Hz, 1H), 8.18 (d, *J* = 8.4 Hz, 1H), 8.13 (dd, *J* = 7.7, 1.8 Hz, 1H), 7.99-7.89 (m, 2H), 7.60-7.39 (m, 4H), 7.26 (dd, *J* = 8.4, 2.0 Hz, 1H), 3.16 (s. 3H);

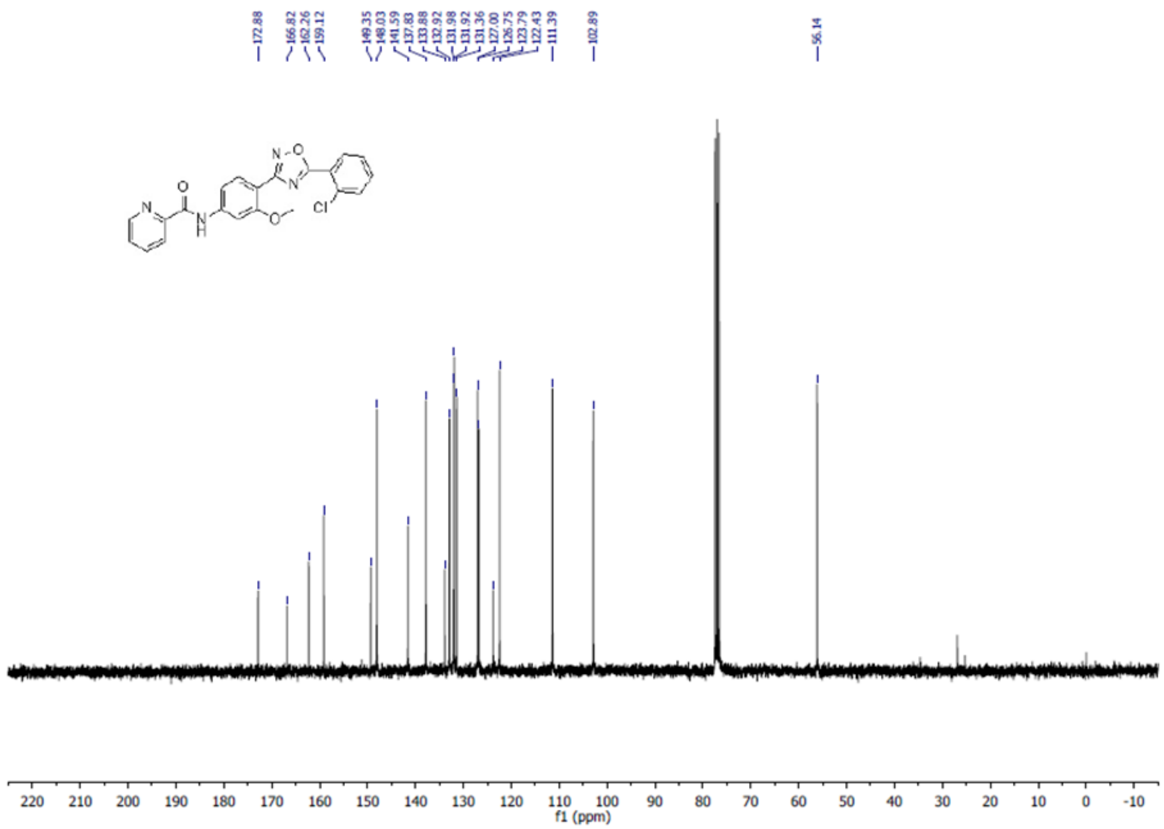

^{13}C NMR (75 MHz, $CDCl_3$), δ ppm: 172.9, 166.8, 162.3, 159.1, 149.4, 148.0, 141.6, 137.8, 133.9, 132.9, 132.0, 131.9, 131.4, 127.0, 126.8, 123.8, 122.4, 111.4, 111.3, 102.9, 56.2; LC-MS (method A) R_T = 3.52 min (purity: 96.20%), *m/z* found: 407.2, calc.: 407.083 [M+H$^+$], HRMS: 407.091, mp=159-161°C.

_N_-(4-(5-(3-Chlorophenyl)-1,2,4-oxadiazol-3-yl)-3-methoxyphenyl)picolinamide (**35**)

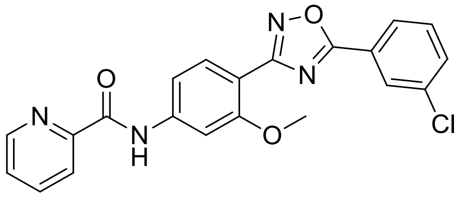

Prepared from **16f** and 2-pyridinecarbonyl chloride, hydrochloride according to general procedure 4. Crude product was purified by column chromatography (SiO_2, $CHCl_3$/MeOH 99:1) followed by maceration (2-PrOH/hexane 1:2); white solid, 0.19 g, 70.4%.

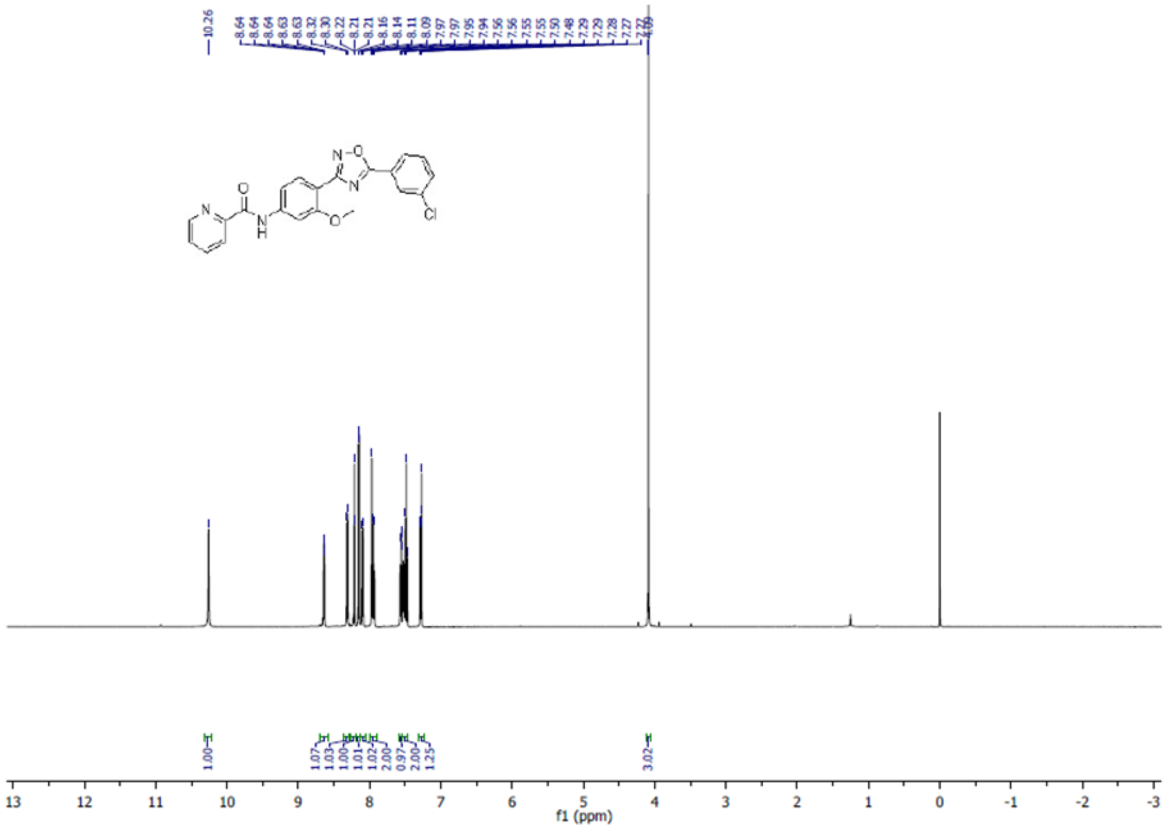

^{1}H NMR (300 MHz, $CDCl_3$), δ ppm: 10.26 (s br. 1H, NH), 8.70-8.58 (m, 1H), 8.31 (d br, J = 7.8 Hz, 1H), 8.21 (t, J = 1.7 Hz, 1H), 8.15 (d, J = 8.4 Hz, 1H), 8.10 (dt, J = 7.8 and 1.3 Hz, 1H), 8.00-7.90 (m, 2H), 7.56 (ddd, J = 8.0, 2.1 and 1.1 Hz, 1H), 7.48 (t, J = 7.9 Hz 2H), 7.28-7.26 (m, 1H), 4.09 (s, 3H, CH_3);

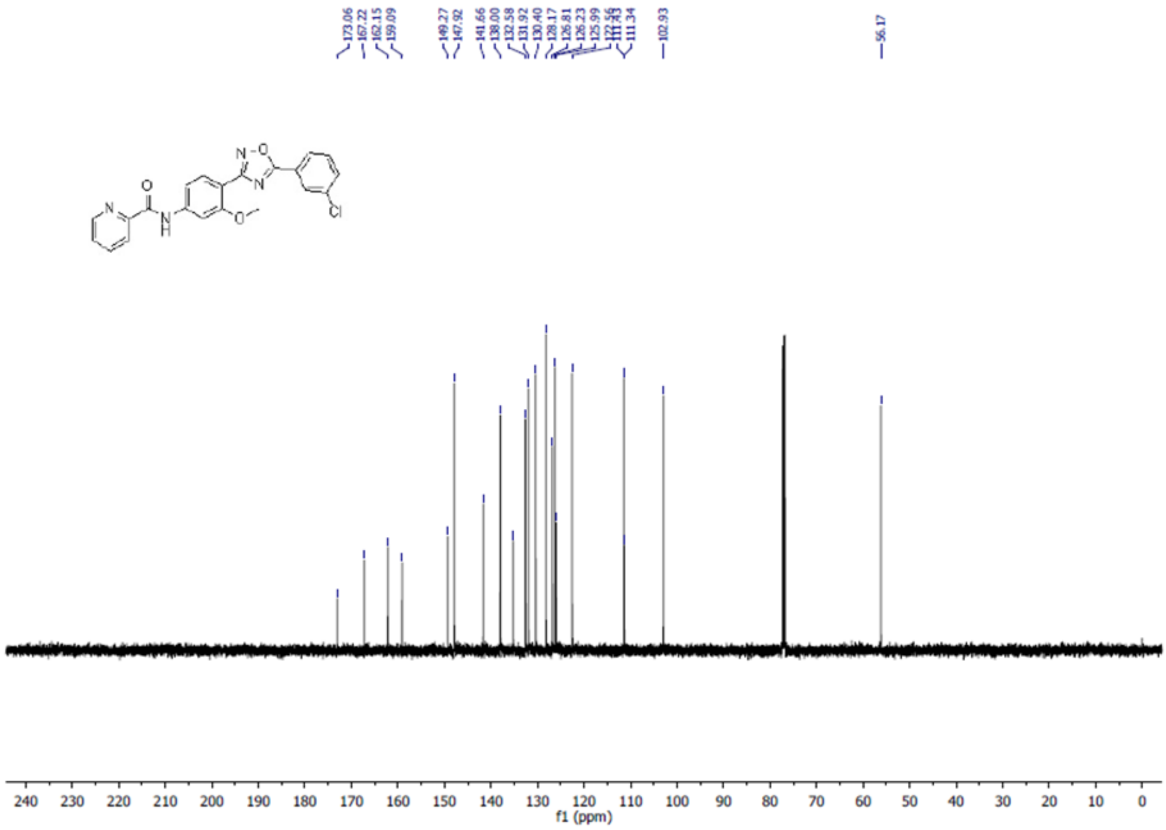

^{13}C NMR (75 MHz, $CDCl_3$), δ ppm: 173.1, 167.2, 162.2, 159.1, 149.3, 147.9, 141.7, 130.0, 135.2, 132.6, 131.9, 130.4, 128.2, 126.8, 126.2, 126.0, 122.5, 111.4, 111.3, 102.9, 56.2; LC-MS (method B) R_T = 3.77 min (purity: 100%), *m/z* found: 407.02, calc.: 407.083 [M+H$^+$], HRMS: 407.085, mp=202-204°C.

N-(4-(5-(4-Chlorophenyl)-1,2,4-oxadiazol-3-yl)-3-methoxyphenyl)picolinamide (**36**)

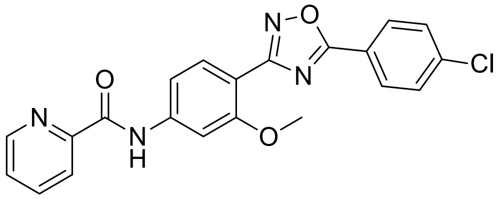

Prepared from **16g** and 2-pyridinecarbonyl chloride, hydrochloride according to general procedure 4. Crude product was purified by column chromatography (SiO_2, $CHCl_3$/MeOH 99:1) followed by maceration (2-PrOH/hexane 1:2); white solid, 0.18 g, 66.7%.

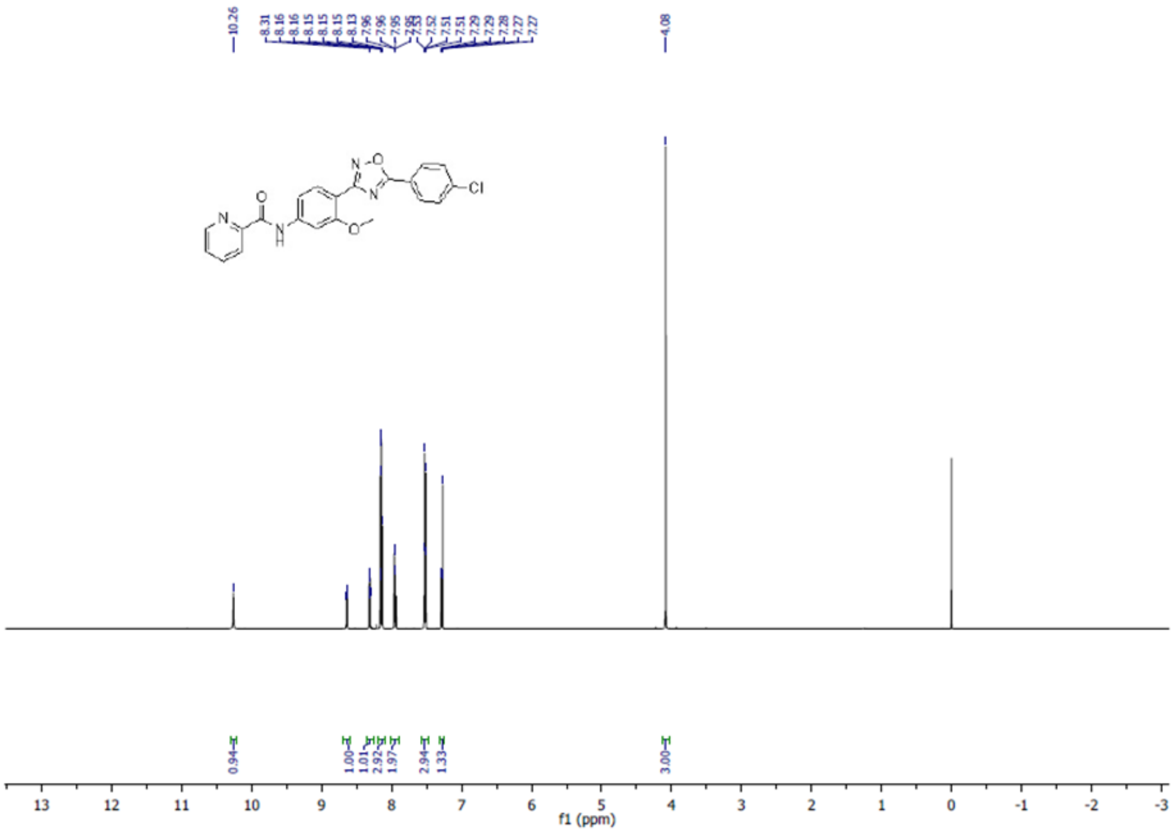

^{1}H NMR (300 MHz, $CDCl_3$), δ ppm: 10.26 (s br. 1H, NH), 8.64 (ddd, J = 4.8, 1.7 and 0.9 Hz, 1H), 8.31 (dt, J = 7.8 and 1.2 Hz, 1H), 8.18-8.08 (m, 3H), 7.95 (dd, J = 5.3 and 1.8 Hz, 2H), 7.54-7.51 (m, 3H), 7.29 (dd, J = 8.5 and 2.0 Hz, 1H), 4.08 (s, 3H, CH_3);

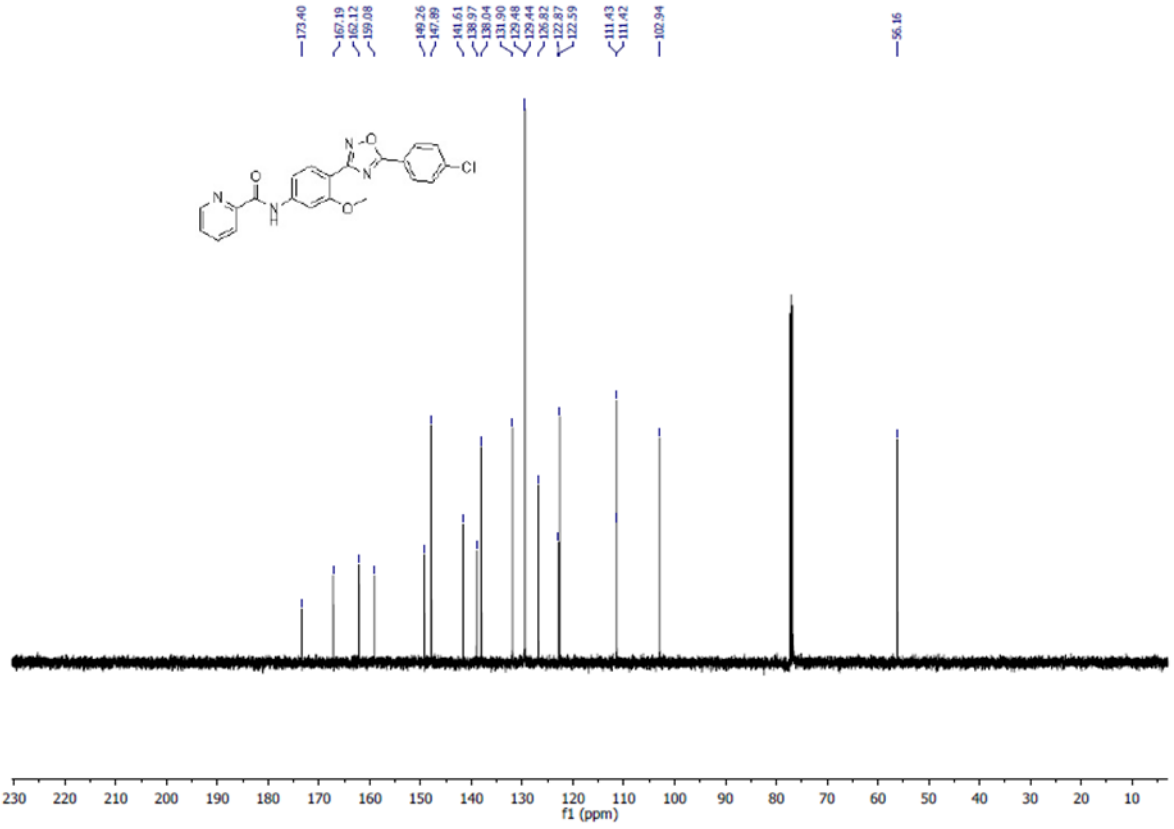

^{13}C NMR (75 MHz, $CDCl_3$), δ ppm: 173.4, 167.2, 162.1, 159.1, 149.3, 147.9, 141.6, 139.1, 138.0, 131.9, 129.5 (2C), 129.4 (2C), 126.8, 122.9, 122.6, 111.4, 111.4, 102.9, 56.2; LC-MS (method B) R_T = 3.82 min (purity: 100%), *m/z* found: 407.2, calc.: 407.083 [M+H^+], HRMS: 407.085, mp=179-181˚C.

***N*-(4-(5-(2-Fluorophenyl)-1,2,4-oxadiazol-3-yl)-3-methoxyphenyl)picolinamide** (**37**)

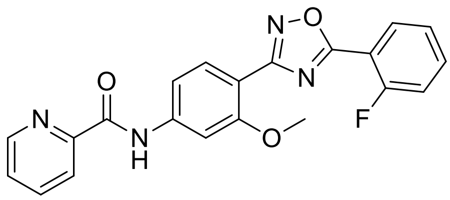

Prepared from **16h** and 2-pyridinecarbonyl chloride, hydrochloride according to general procedure 4. Crude product was purified by column chromatography (Al_2O_3, AcOEt/hexane 2:1) followed by maceration (MeOH); white solid 0.128 g, 62.4%.

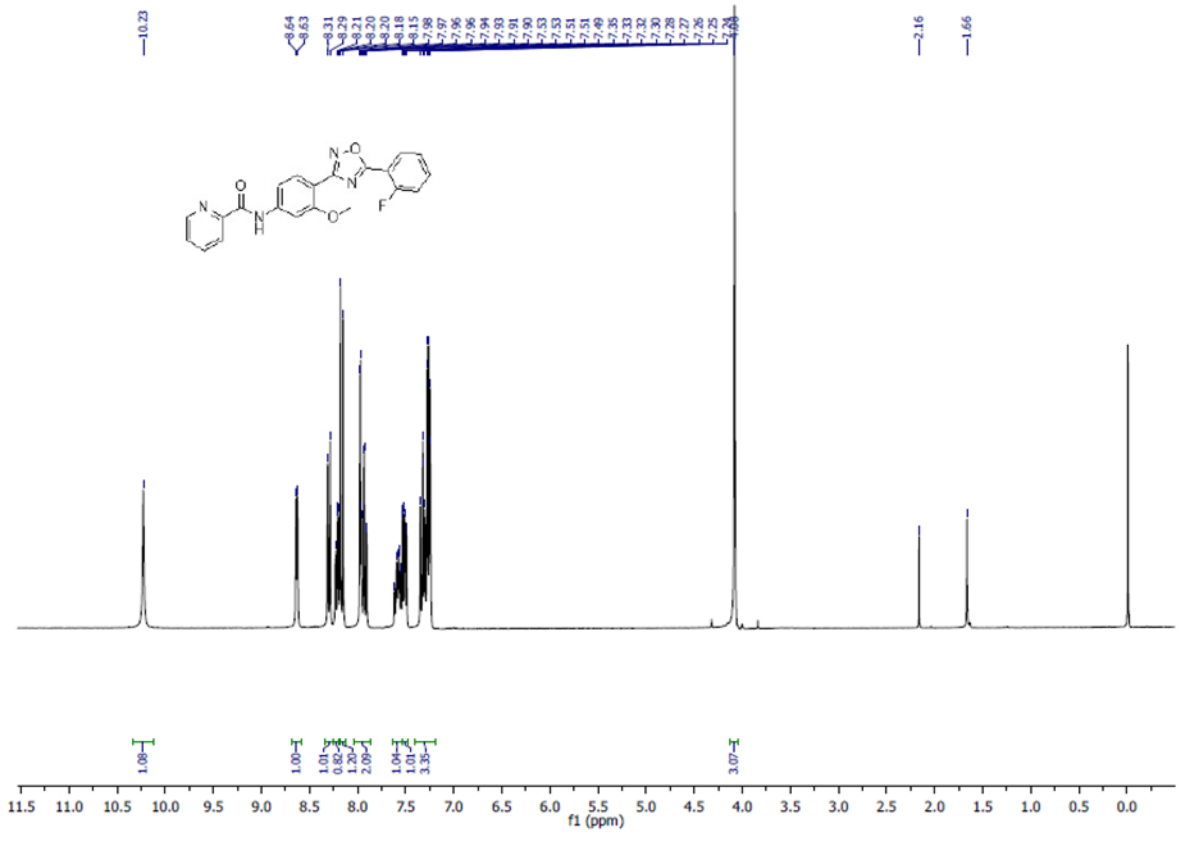

^{1}H NMR (300 MHz, $CDCl_3$), δ ppm: 10.23 (s br. 1H, NH), 8.64 (ddd, *J* = 4.8, 1.6 and 0.9 Hz, 1H), 8.30 (dt, *J* = 7.8 and 1.0 Hz, 1H), 8.21 (td, *J* = 7.6 and 1.8 Hz, 1H), 8.16 (d, *J* =8.4 Hz, 1H), 7.98 (d, *J* = 1.9 Hz, 1H), 7.93 (td, *J* = 7.8, 1.7 Hz, 1H), 7.62-7.55 (m, 1H), 7.54 (ddd, *J* = 7.6, 4.8 and 1.2 Hz, 1H), 7.35-7.24 (m, 3H), 4.08 (s, 3H, CH_3);

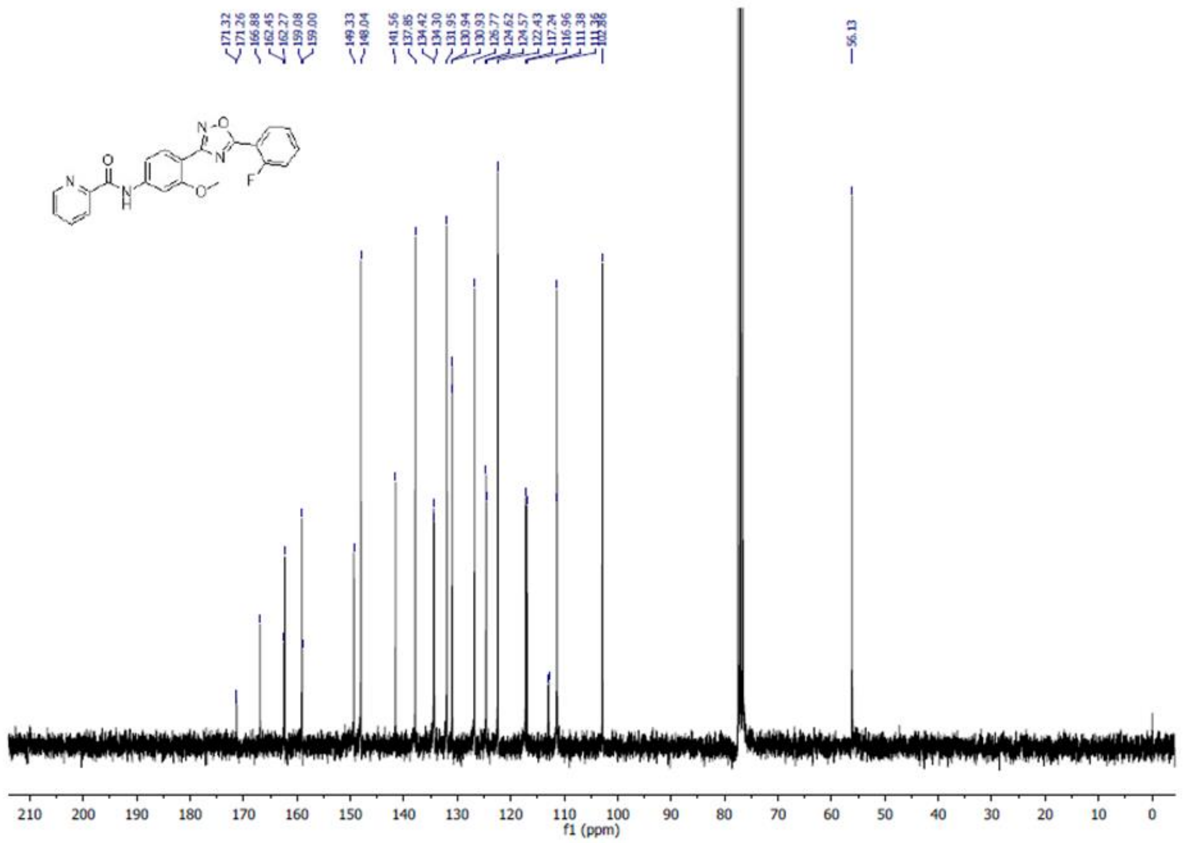

^{13}C NMR (75 MHz, $CDCl_3$), δ ppm: 171.3 (d, J = 4.2 Hz), 166.9, 162.3, 160.8 (d, J = 260.4 Hz), 159.1, 149.3, 148.0, 141.6, 137.9, 134.4 (d, J = 8.6 Hz), 131.9, 130.9 (d, J = 0.9 Hz), 126.8, 124.6 (d, J = 3.8 Hz), 122.4, 117.1 (d, J = 20.9 Hz), 113.0 (d, J = 11.4 Hz), 111.4, 111.4, 102.9, 56.1; LC-MS (method A) R_T = 3.33 min (purity: 96.10%), *m/z* found: 391.4, calc.: 391.113 [M+H$^+$], HRMS: 391.124, mp=164-166°C.

_N_-(4-(5-(2-Chloro-4-fluorophenyl)-1,2,4-oxadiazol-3-yl)-3-methoxyphenyl)picolinamide (38)

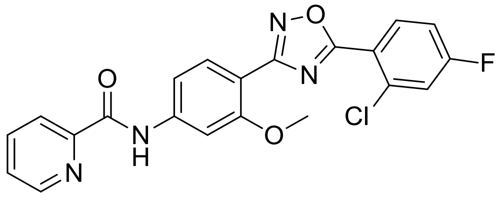

Prepared according to general procedure 4 from **16i** and 2-pyridinecarbonyl chloride, hydrochloride. Crude product was purified by column chromatography (SiO_2, $CHCl_3$/hexane 2:1) followed by maceration (2-PrOH/hexane 1:2); white solid, 0.18 g, 90.0%.

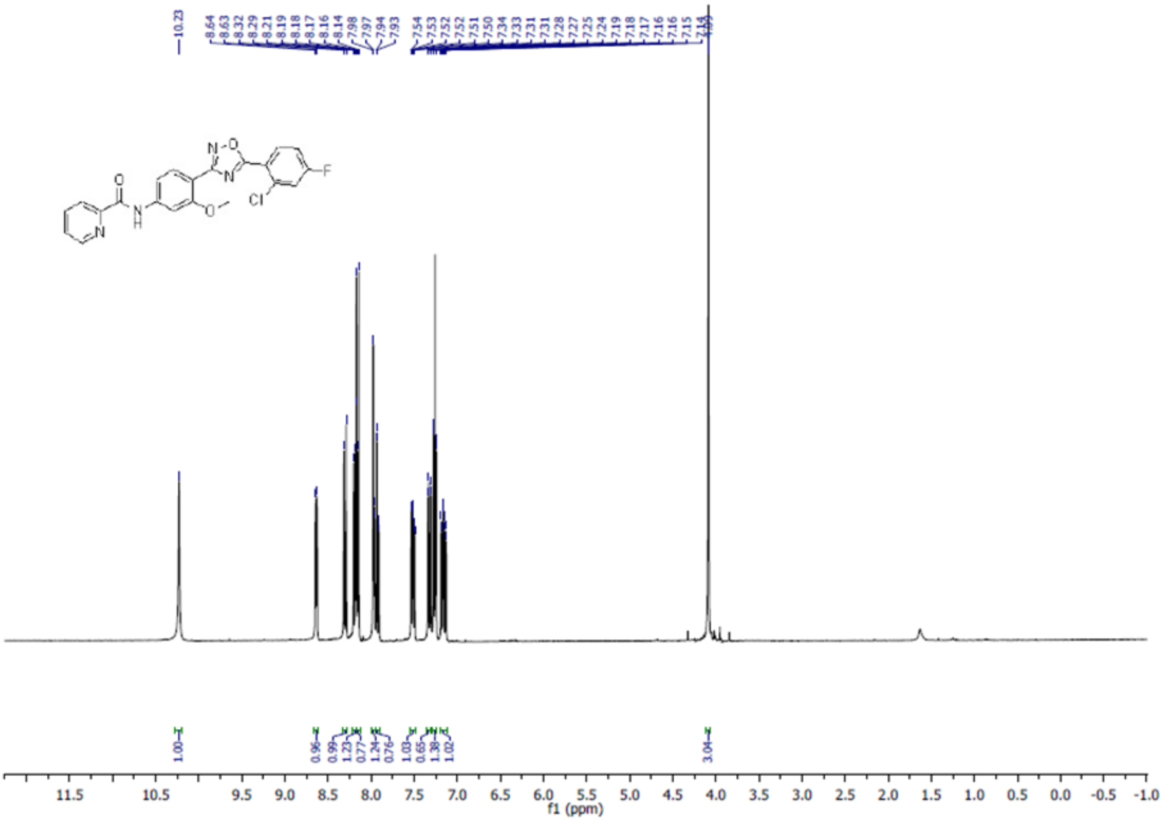

^{1}H NMR (300 MHz, $CDCl_3$), δ ppm: 10.23 (s br. 1H, NH), 8.64 (ddd, *J* = 4.8, 1.6 and 0.9 Hz, 1H), 8.30 (dt, *J* = 7.8 and 1.0 Hz, 1H), 8.19 (dd, *J* = 9.0 and 6.0 Hz, 1H), 8.15 (d, *J* =8.4 Hz, 1H), 7.98 (d, *J* = 1.9 Hz, 1H), 7.94 (td, *J* = 7.8, 1.7 Hz, 1H), 7.51 (ddd, *J* = 7.6, 4.8 and 1.2 Hz, 1H), 7.32 (dd, *J* = 8.4 and 2.5 Hz, 1H), 7. 26 (dd, *J* = 8.5 and 2.0 Hz, 1H), 7.16 (ddd, *J* = 8.8, 7.6 and 2.5 Hz, 1H), 4.08 (s, 3H, CH_3);

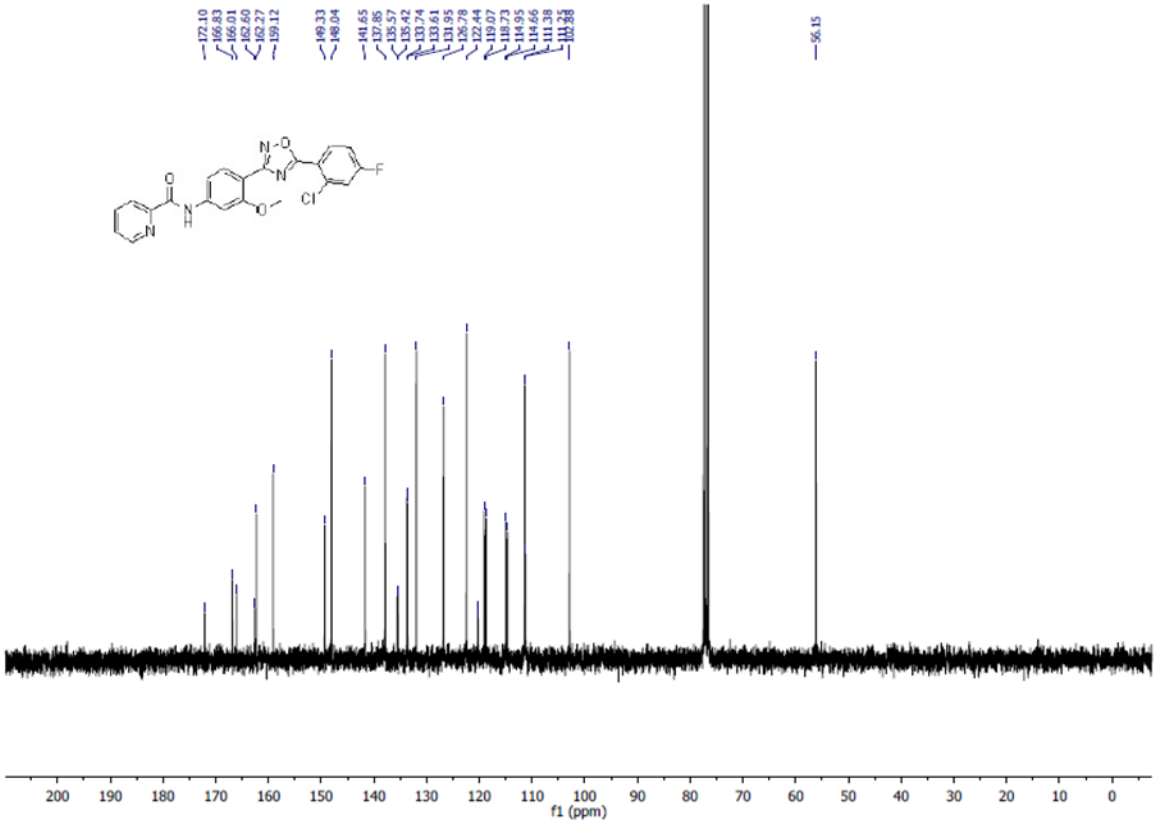

^{13}C NMR (75 MHz, $CDCl_3$), δ ppm: 172.1, 166.8, 164.3 (d, J = 257.2 Hz), 162.3, 159.1, 149.3, 148.0, 141.7, 137.9, 135.5 (d, J = 10.8 Hz), 133.7 (d, J = 9.7 Hz), 132.0, 126.8, 122.4, 120.2 (d, J = 3.7 Hz), 118.9 (d, J = 25.0 Hz), 114.8 (d, J = 21.7 Hz), 111.4, 111.3, 102.9, 56.2; LC-MS (method A) R_T = 3.65 min (purity: 99.60%), *m/z* found: 425.02, calc.: 425.074 [M+H$^+$], HRMS: 425.096, mp=209-210°C.

***N*-(4-(5-(2-Chlorophenyl)-1,2,4-oxadiazol-3-yl)-3-methoxyphenyl)-6-fluoropicolinamide (39)**

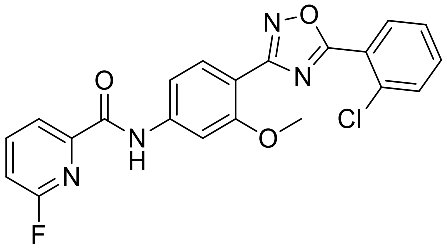

Prepared from **16e** and 6-fluoropyridine-2-carboxylic acid according to general procedure 5. After 24 h of stirring in rt additional portion of BOP (1.0 eq) and acid (1.1 eq) was added to the reaction mixture. Stirring was continued for 12h (LC-MS control). Crude product was purified by column chromatography (Al_2O_3, gradient from AcOEt/hexane 1:2 to AcOEt/hexane 2:1) followed by maceration (2-PrOH/hexane 1:2); white solid, 0.10 g, 47.6%.

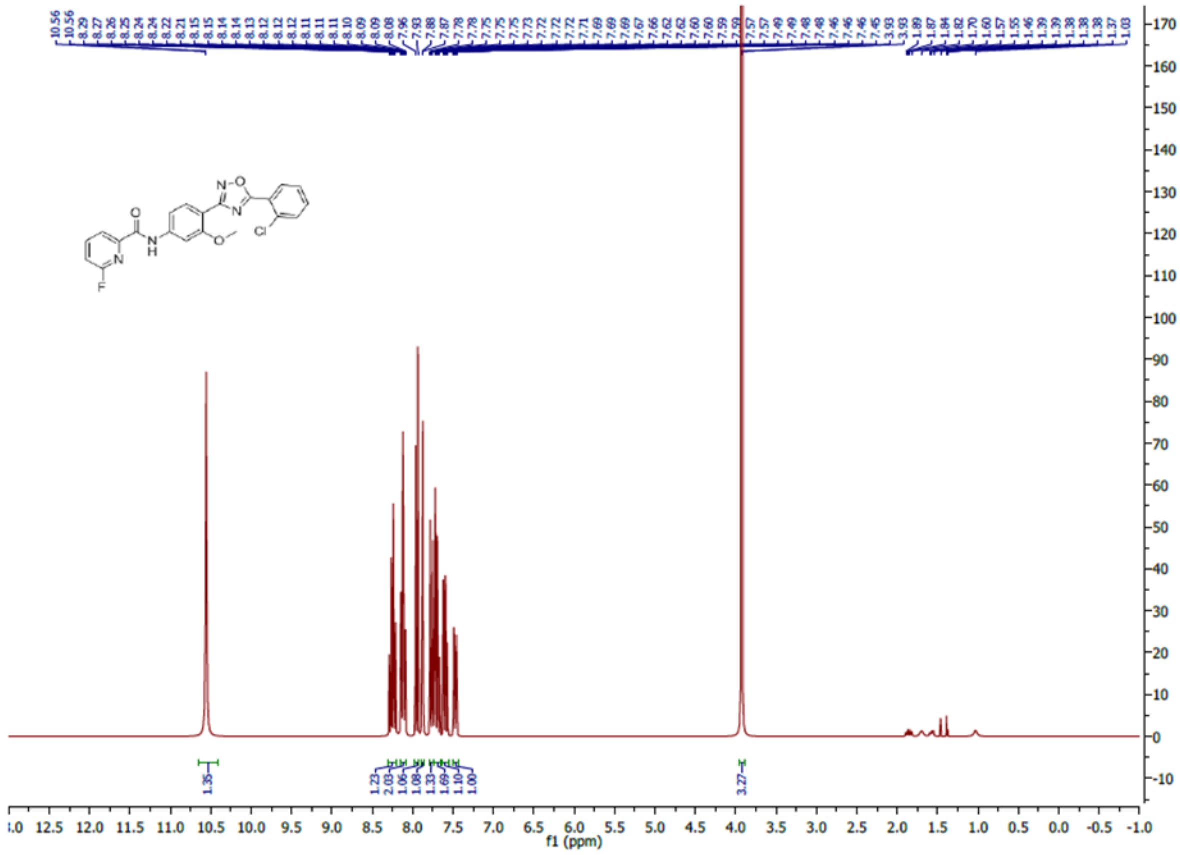

^1H NMR (300 MHz, DMSO-d_6), δ ppm: 10.56 (s br. 1H, NH), 8.29-8.21 (m, 1H), 8.15-8.09 (m, 2H), 7.94 (d, J = 8.5 Hz, 1H), 7.87 (d, J =1.9 Hz, 1H,), 7.76 (dd, J = 8.6 and 1.9 Hz, 1H),

7.73-7.66 (m, 2H), 7.61 (ddd, J = 7.7, 6.9 and 1.9 Hz, 1H), 7.49-7.46 (m, 1H), 3.93 (s, 3H, CH_3);

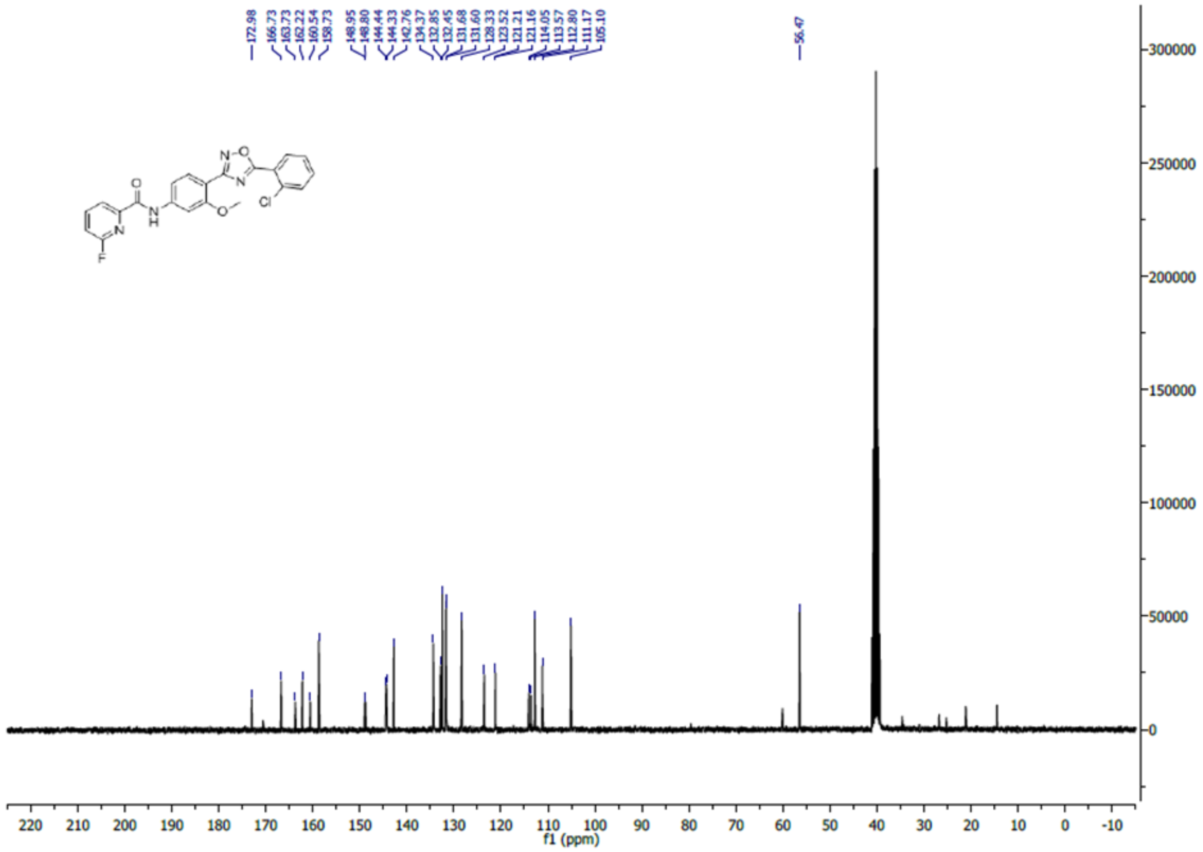

^{13}C NMR (75 MHz, DMSO-d6), δ ppm: 173.0, 166.7, 162.2, 162.1 (d, J = 241.1 Hz), 158.7, 148.9 (d, J = 11.4 Hz), 144.4 (d, J = 8.0 Hz), 142.8, 134.4, 132.8, 132.5, 131.7, 131.6, 128.3, 123.5, 121.2 (d, J = 3.6 Hz), 113.8 (d, J = 36.3 Hz), 112.8, 111.2, 105.1, 56.5; LC-MS (method A) R_T = 3.60 min (purity: 100%), m/z found: 425.02, calc.: 425.074 [M+H+], HRMS: 425.079, mp=177-179°C.

5-Chloro-*N*-(4-(5-(2-chlorophenyl)-1,2,4-oxadiazol-3-yl)-3-methoxyphenyl)-3-fluoro-picolinamide (**40**)

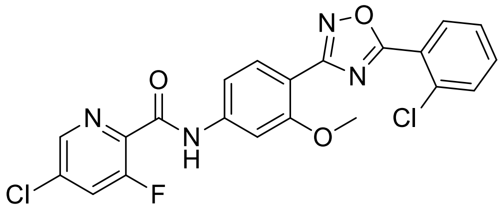

Prepared according to general procedure 5 from **16e** and 5-chloro-3-fluoropyridine-2-carboxylic acid. Crude product was purified by column chromatography (SiO_2, gradient from AcOEt/hexane 1:2 to 2:1) followed by maceration (2-PrOH/hexane 1:2); white solid, 0.16 g, 70.2%.

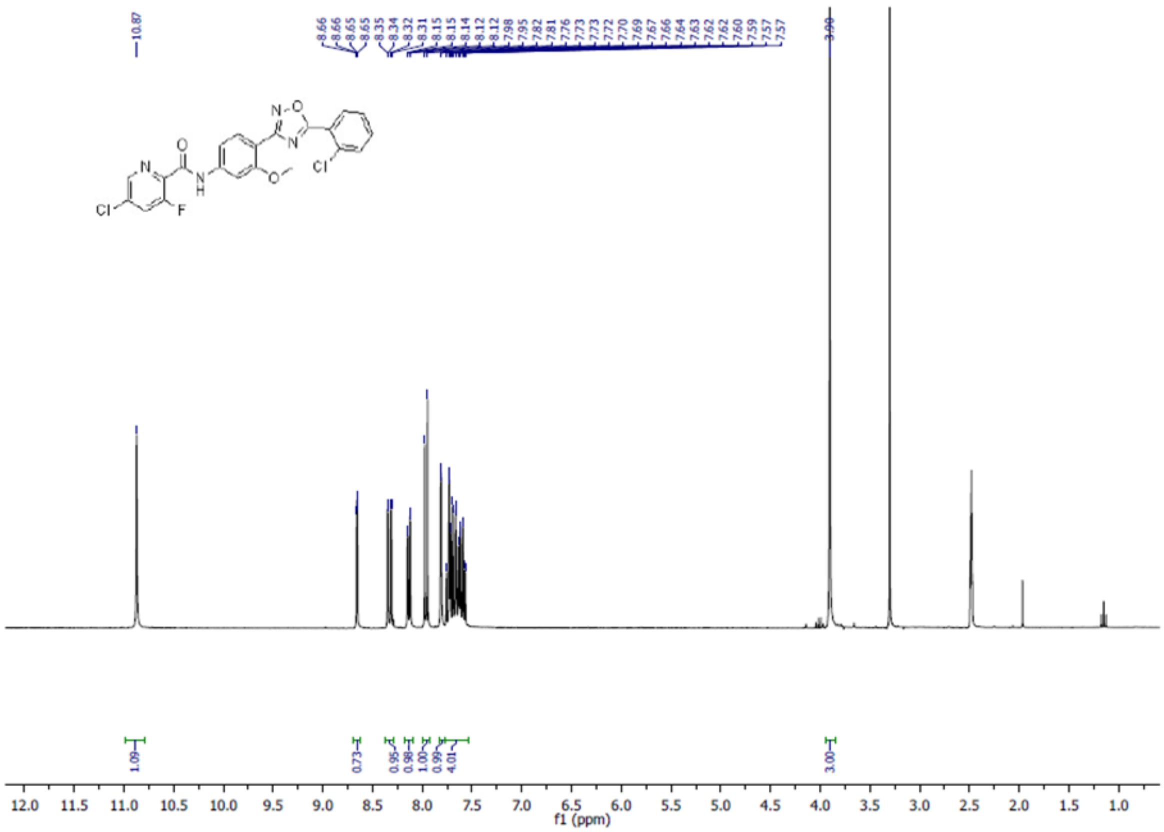

^{1}H NMR (300 MHz, DMSO-d_6), δ ppm: 10.87 (s br. 1H, NH), 8.6 (dd, *J* = 1.9 and 0.9 Hz, 1H), 8.33 (dd, *J* = 10.4 and 1.9 Hz, 1H), 8.13 (ddd, *J* = 7.6, 1.6 and 0.4 Hz, 1H), 7.96 (d, *J* = 8.5 Hz, 1H), 7.81 (d, *J* = 1.8 Hz, 1H), 7.76-7.57 (m, 4H), 3.90 (s, 3H, CH_3);

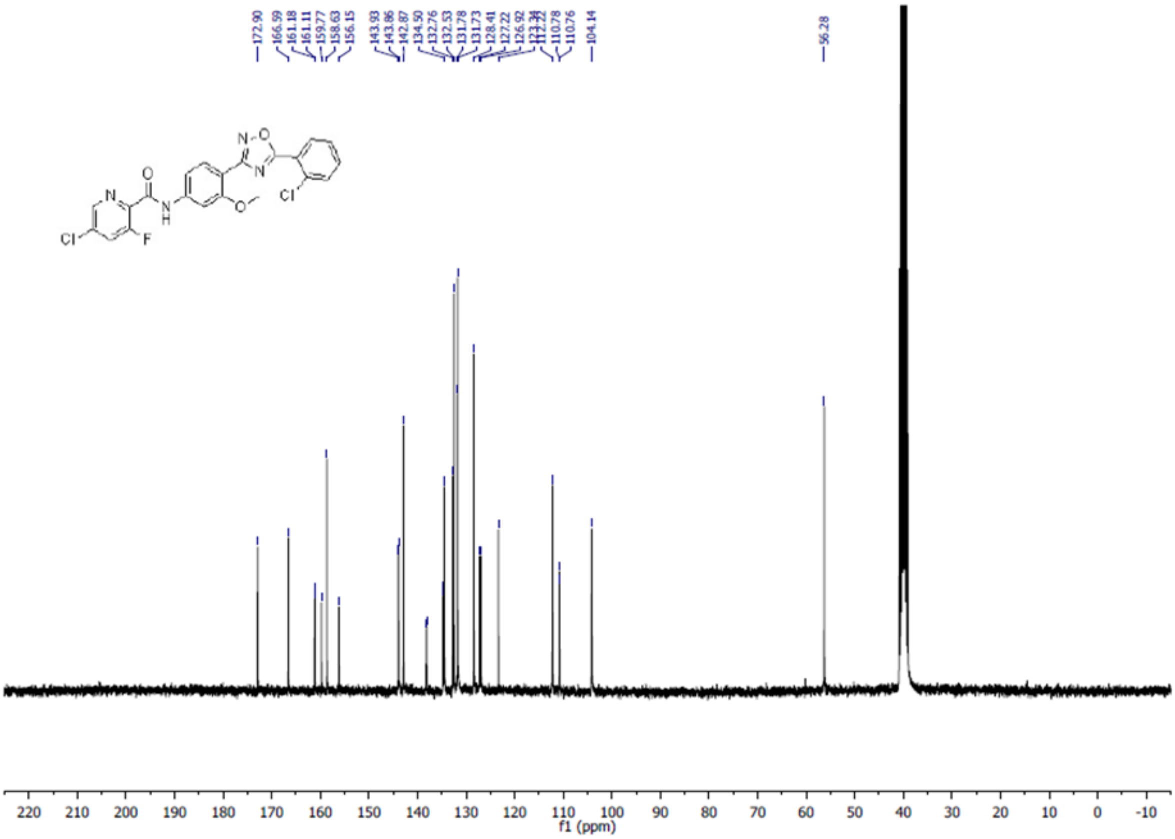

^{13}C NMR (75 MHz, DMSO-d_6), δ ppm: 172.9, 166.6, 161.1 (d, *J* = 5.6 Hz), 158.6, 158.0 (d, *J* = 272.8 Hz), 143.9 (d, *J* = 5.0 Hz), 142.9, 138.1 (d, *J* = 7.7 Hz), 134.7 (d, *J* = 4.7 Hz), 134.5, 132.8, 132.5, 131.8, 131.7, 128.4, 127.0 (d, *J* = 22.4 Hz), 123.3, 112.2, 110.8 (d, *J* = 1.8 Hz), 104.1, 56.3; LC-MS (method A) R_T = 3.65 min (purity: 100%), *m/z* found: 459.46, calc.: 459.035 [M+H$^+$], HRMS:459.035, mp=188-189°C.

6-Chloro-*N*-(4-(5-(2-chlorophenyl)-1,2,4-oxadiazol-3-yl)-3-methoxyphenyl)picolinamide (41)

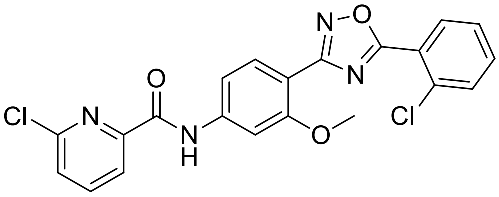

Prepared according to general procedure 5 from **16e** and 6-chloropyridine-2-carboxylic acid. Solid that was precipitated from the reaction mixture was filtered and washed with MeCN. The filtrate was concentrated, and the rest of the product was isolated by column chromatography (SiO_2, $CHCl_3$/MeOH 1:1). Combined products were further purified by maceration (2-PrOH/hexane/$CHCl_3$ 1:2:0.5); white solid, 0.094 g, 42.9%.

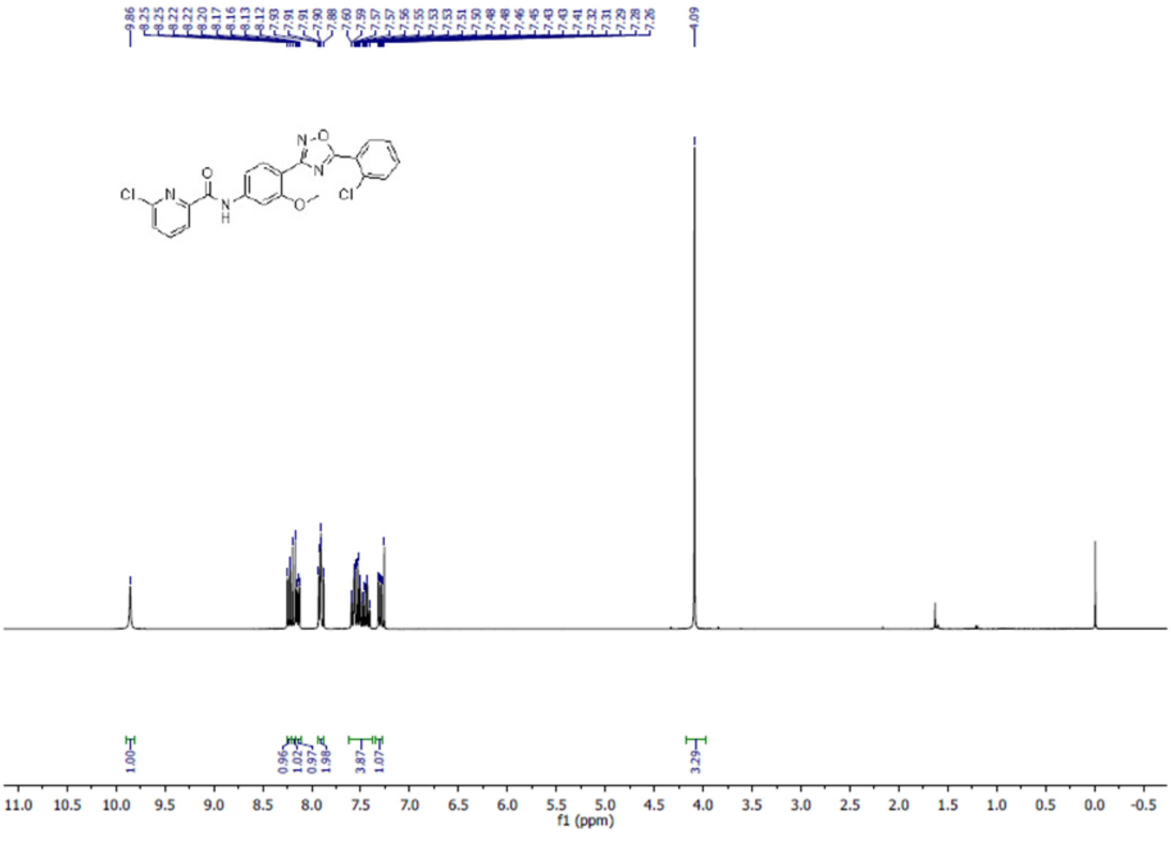

^{1}H NMR (300 MHz, $CDCl_3$), δ ppm: 9.86 (s br. 1H, NH), 8.23 (dd, J = 7.6 and 0.8 Hz, 1H), 8.18 (d, J = 8.4 Hz, 1H), 8.15 (dd, J = 7.6 and 1.7 Hz, 1H), 7.93-7.89 (m, 2H), 7.60-7.40 (m, 4H), 7.30 (dd, J = 8.5, 1.9 Hz, 1H), 4.09 (s, 3H, CH_3);

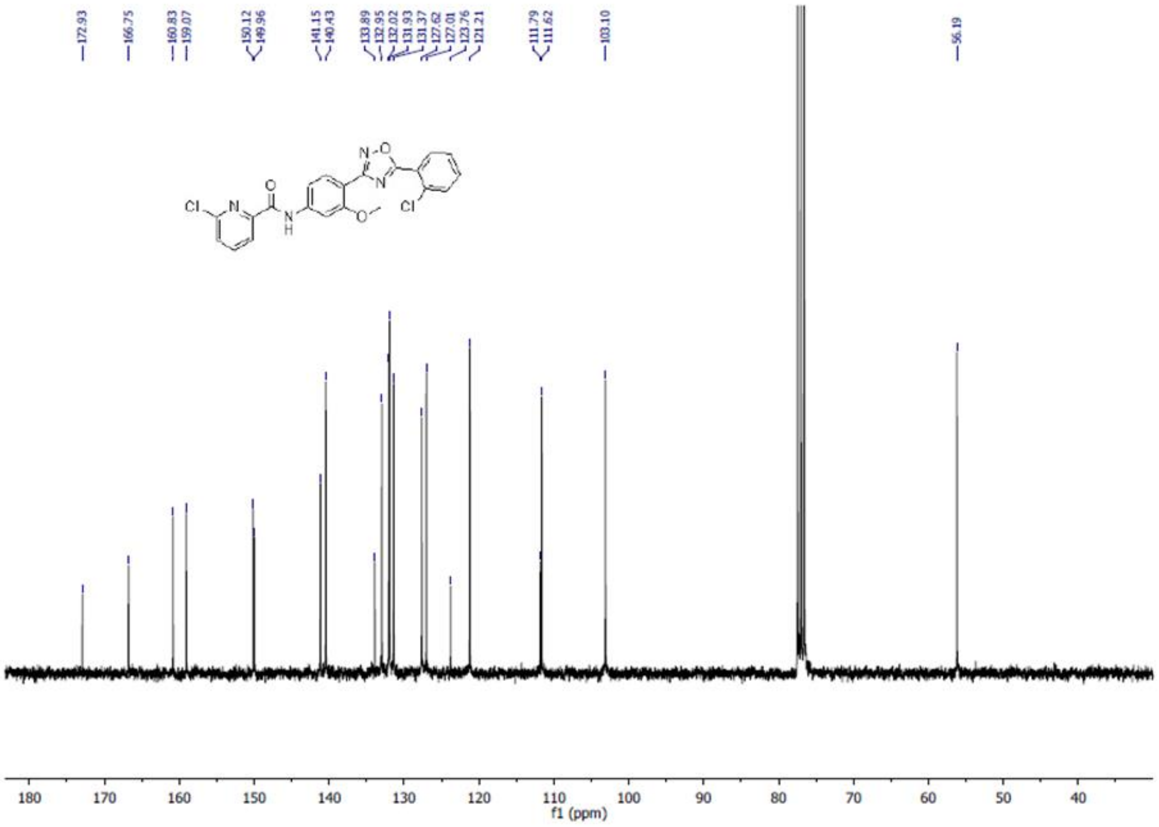

^{13}C NMR (75 MHz, $CDCl_3$), δ ppm: 172.9, 166.8, 160.8, 159.1, 150.1, 150.0, 141.2, 140.4, 133.9, 133.0, 132.0, 131.9, 131.4, 127.6, 127.0, 123.8, 121.2, 111.8, 111.6, 103.1, 56.2; LC-MS (method A) R_T = 3.76 min (purity: 100%), *m/z* found: 441.56, calc.:441.044 [$M+H^+$], HRMS: 441.063, mp=175-177°C.

3-Chloro-*N*-(4-(5-(2-chlorophenyl)-1,2,4-oxadiazol-3-yl)-3-methoxyphenyl)-6-methoxy-picolinamide (**42**)

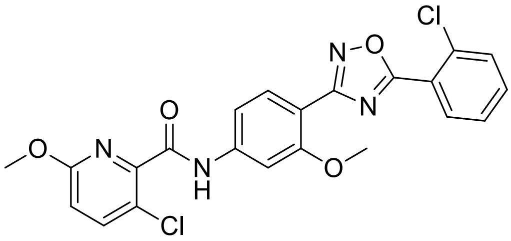

Prepared according to general procedure 4 from **16e** and 3-chloro-6-methoxypyridine-2-carbonyl chloride. Crude product was purified by column chromatography (SiO_2, $CHCl_3$) followed by maceration (2-PrOH/hexane 1:2); white solid, 0.24 g,100.0%.

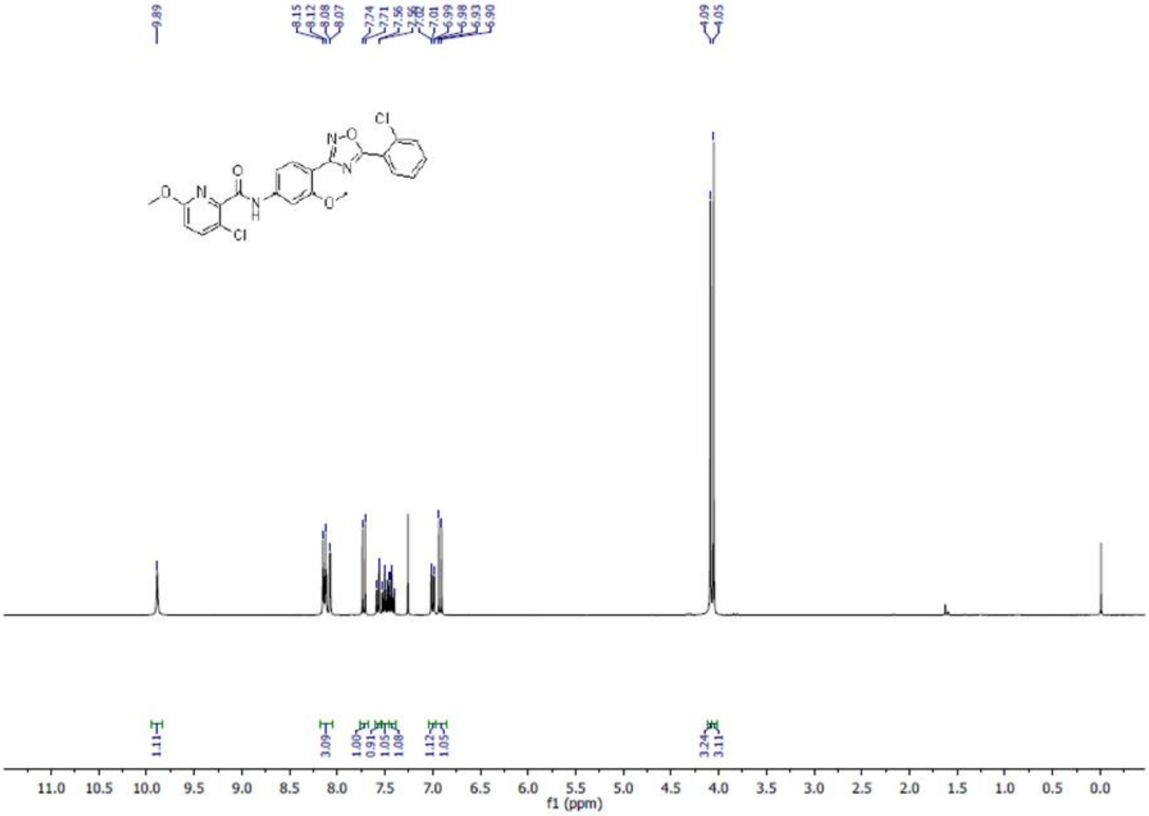

^1H NMR (300 MHz, $CDCl_3$), δ ppm: 9.89 (s br. 1H, NH), 8.15-8.07 (m, 3H), 7.73 (d, J = 8.8 Hz, 1H), 7.56 (dd, J = 8.0 and 1.4 Hz, 1H), 7.50 (td, J = 7.8 and 1.8 Hz, 1H), 7.43 (td, J = 7.6

and 1.4 Hz, 1H), 7.00 (dd, *J* = 8.4 and 2.0 Hz, 1H), 6.92 (d, *J* = 8.8, 1H), 4.09 (s, 3H, CH_3), 4.05 (s, 3H, CH_3);

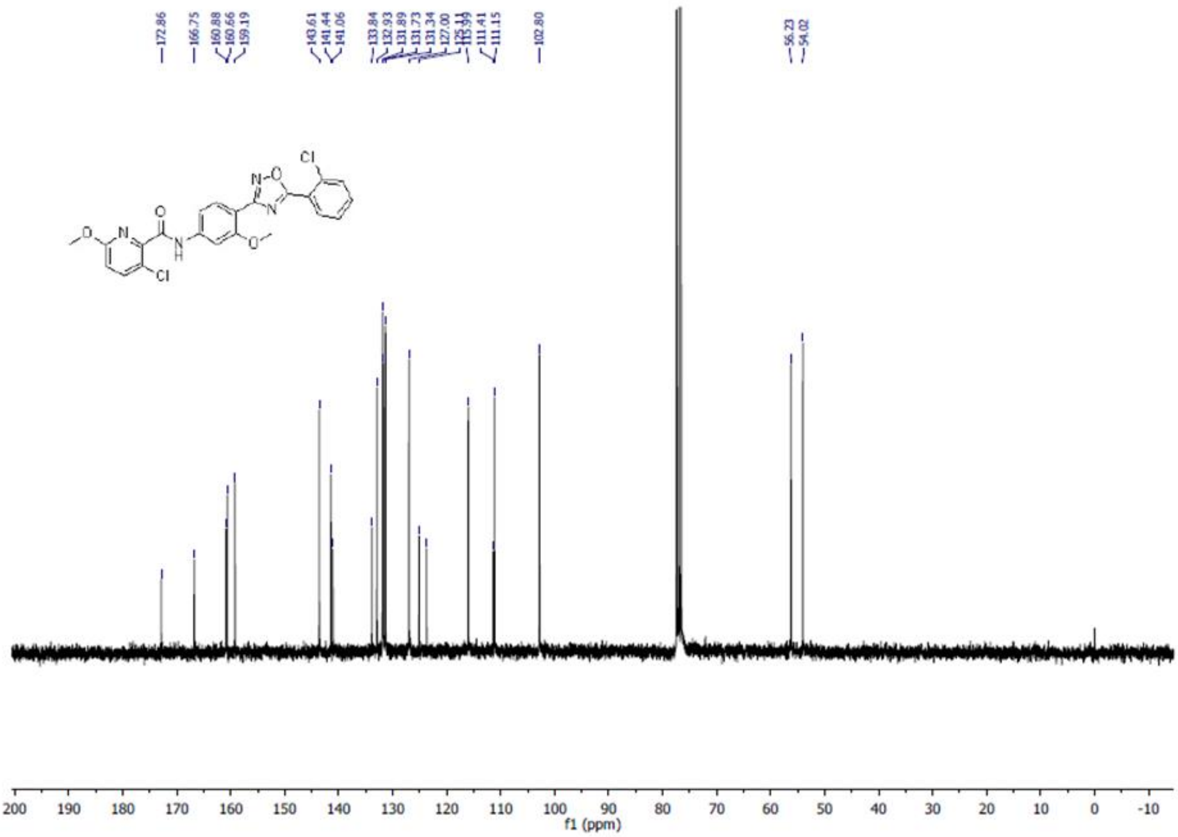

^{13}C NMR (75 MHz, $CDCl_3$), δ ppm: 172.9, 166.8, 160.9, 160.7, 159.2, 143.6, 141.5, 141.1, 133.8, 132.9, 131.9, 131.7, 131.3, 127.0, 125.1, 123.7, 116.0, 111.4, 111.2, 102.8, 56.2, 54.0; LC-MS (method A) R_T = 3.74 min (purity: 100%), *m/z* found: 471.4, calc. 471.055 [M+H$^+$], HRMS: 471.078, mp=174-175˚C.

_N_-(4-(5-(2-Chlorophenyl)-1,2,4-oxadiazol-3-yl)-3-methoxyphenyl)-4,6-difluoro-picolinamide (**43**)

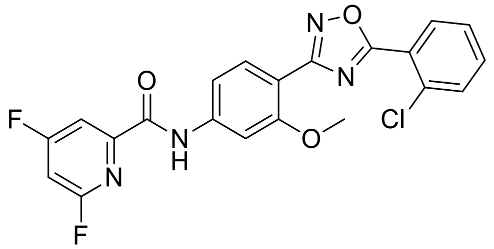

Prepared according to general procedure 5 from **16e** and 3,5-difluoropyridine-2-carboxylic acid. The product was isolated from the reaction mixture by column chromatography (SiO_2, $CHCl_3$) and further purified by maceration (2-PrOH/hexane 1:2); white solid, 0.22 g, 100.0%.

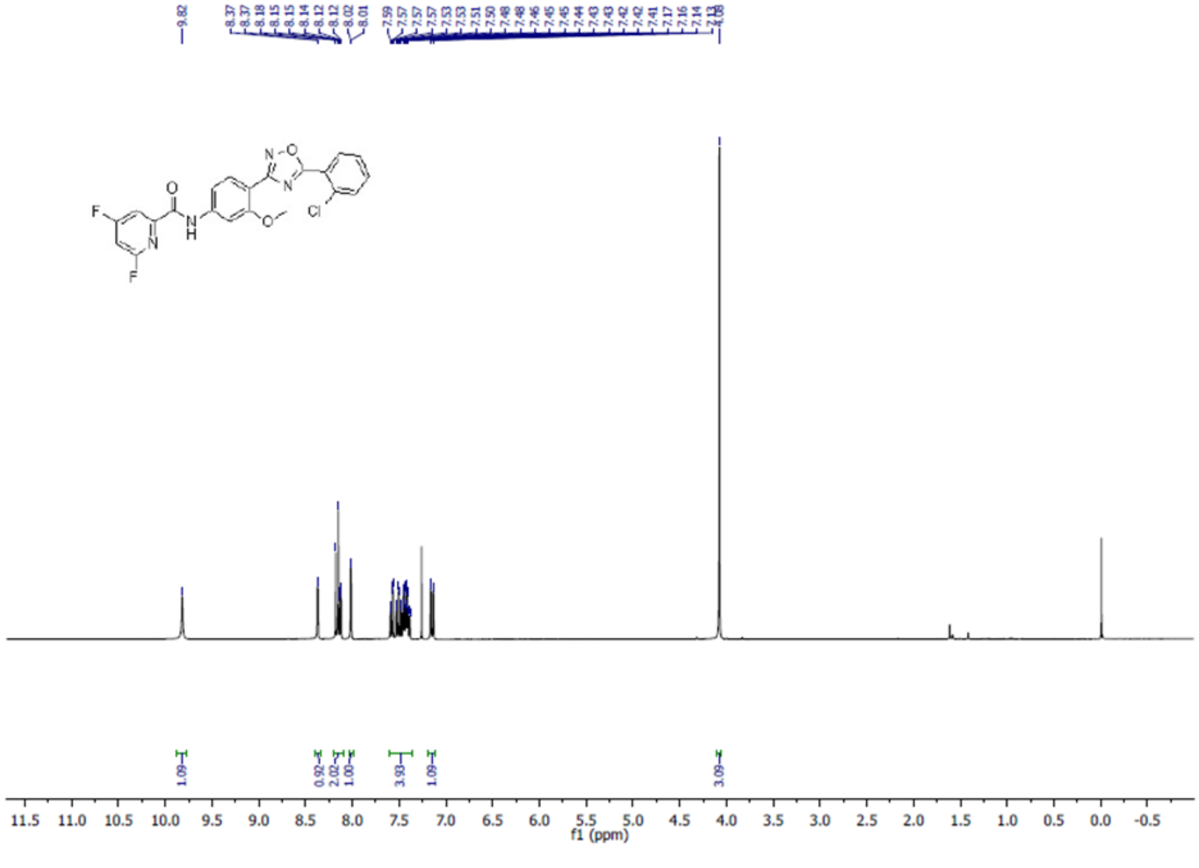

^{1}H NMR (300 MHz, $CDCl_3$), δ ppm: 9.82 (s br. 1H, NH), 8.37 (d br., J = 2.0 Hz, 1H), 8.18-8.12 (m, 2H), 8.02 (d, J = 1.9 Hz, 1H), 7.60-7.38 (m, 4H), 7.15 (dd, J = 8.4 and 2.0 Hz, 1H), 4.08 (s, 3H, CH_3);

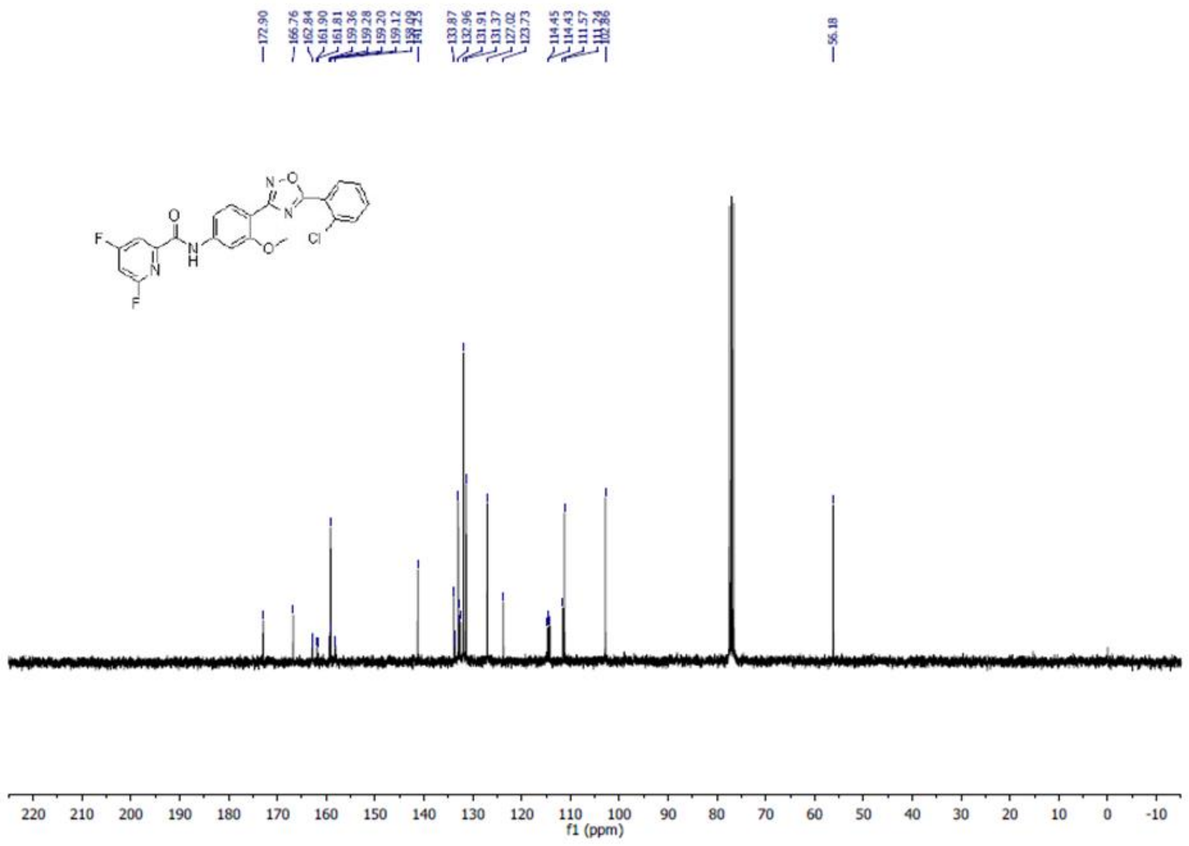

^{13}C NMR (75 MHz, $CDCl_3$), δ ppm: 172.9, 166.8, 162.4 (dd, *J* = 76.7 and 6.0 Hz), 159.2, 159.1, 158.7 (dd, *J* = 88.4 and 6.0 Hz), 141.3, 133.9, 133.7 (t, *J* = 3.9 Hz), 133.0, 132.7 (dd, *J* = 24.2 and 5.0 Hz), 131.9 (2C), 131.4, 127.0, 123.7, 114.4 (dd, *J* = 22.2 and 20.8 Hz), 111.6, 111.2, 102.9, 56.2; LC-MS (method A) R_T = 3.53 min (purity: 99.05%), *m/z* found: 443.9, calc. 443.064 $[M+H^+]$, HRMS: 443.076, mp=158-159°C.

_N_-(4-(5-(2-Chlorophenyl)-1,2,4-oxadiazol-3-yl)-3-methoxyphenyl)-3-fluoropicolinamide (44)

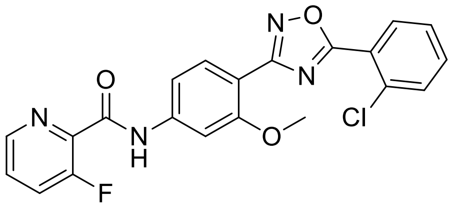

Prepared according to general procedure 5 from **16e** and 3-fluoropyridine-2-carboxylic acid. Precipitated solid was filtered from the reaction mixture and washed with MeCN followed by maceration (2-PrOH/hexane 1:2); white solid, 0.21 g, 100.0%.

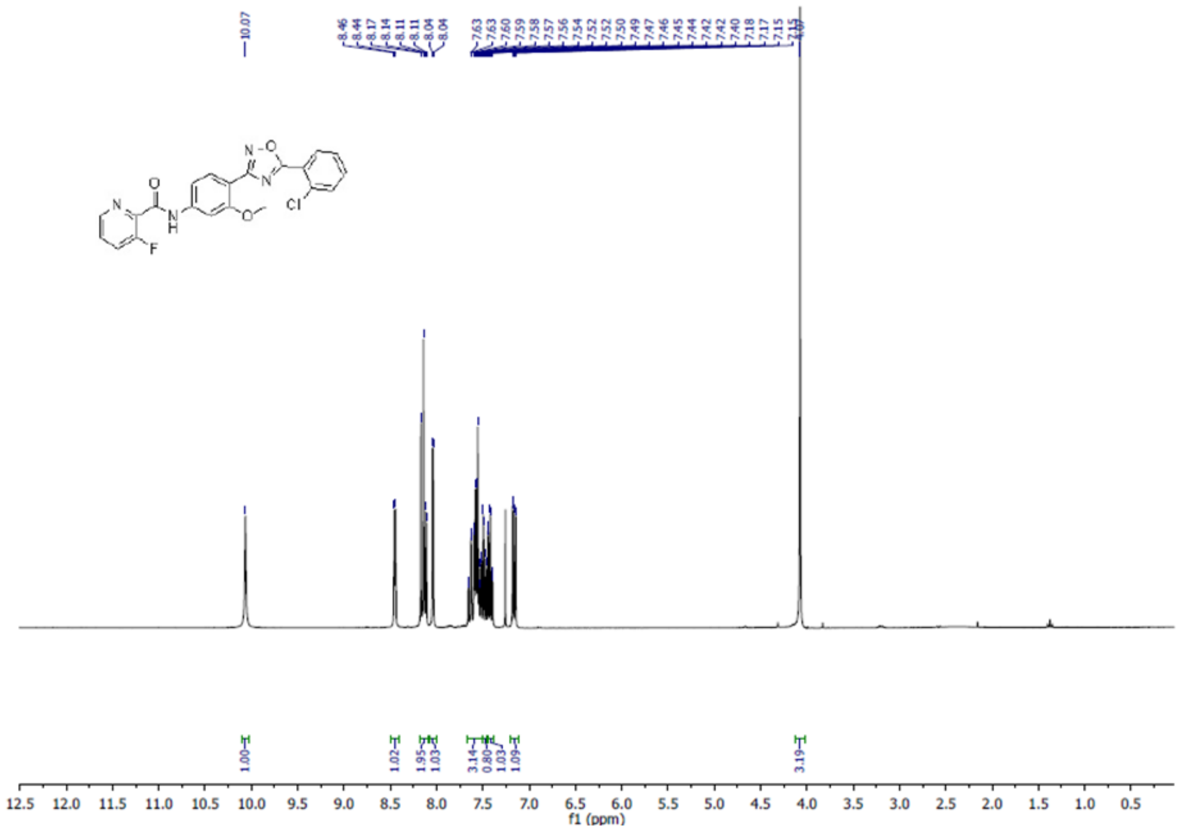

^{1}H NMR (300 MHz, $CDCl_3$), δ ppm: 10.07 (s br. 1H, NH), 8.45 (dt, J = 4.4 and 1.4 Hz, 1H), 8.17-8.11 (m, 2H), 8.04 (d, J = 1.9 Hz, 1H), 7.66-7.53 (m, 3H), 7.50 (dd, J = 7.9, 1.9, 1H), 7.42 (td, J = 7.6 and 1.5 Hz, 1H), 7.16 (dd, J = 8.5 and 2.0 Hz, 1H), 4.07 (s, 3H, CH_3);

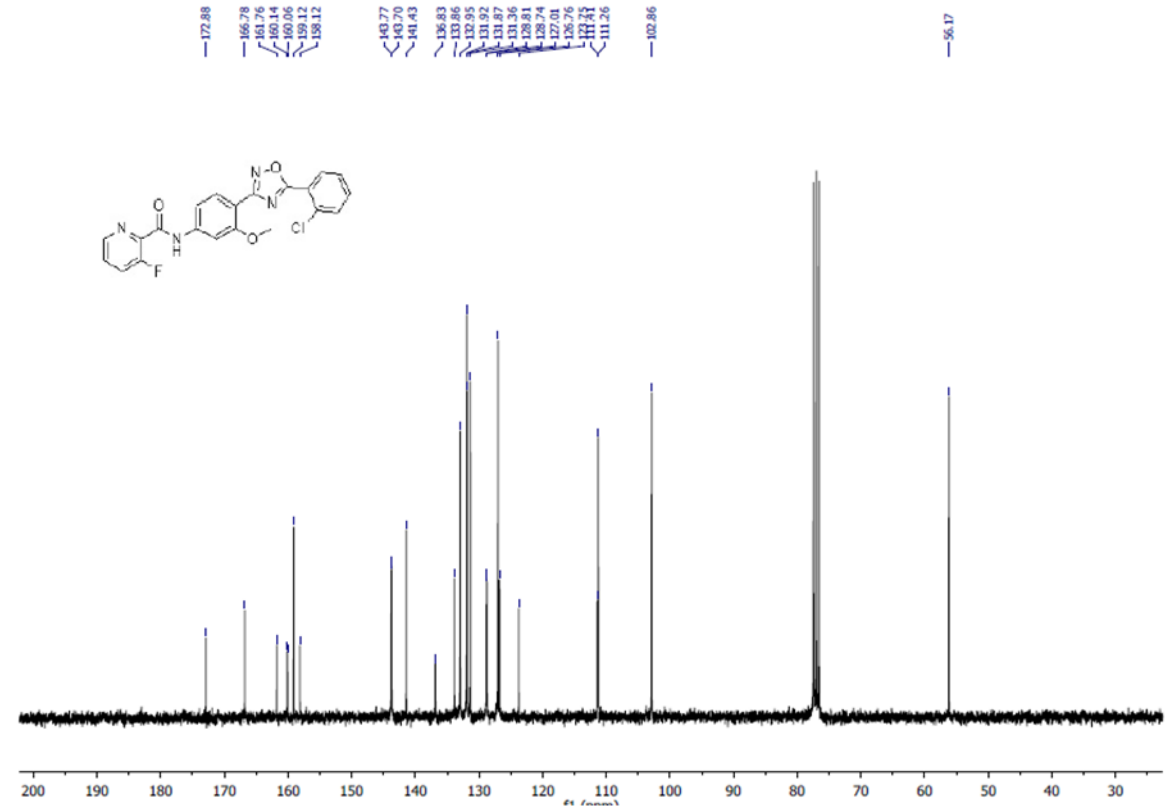

^{13}C NMR (75 MHz, $CDCl_3$), δ ppm: 172.9, 166.8, 160.0 (d, *J* = 274.7 Hz), 160.1 (d, *J* = 6.0 Hz), 159.1, 143.7 (d, *J* = 5.6 Hz), 141.4, 136.8 (d, *J* = 3.4 Hz), 133.9, 133.0, 131.9, 131.8, 131.4, 128.8 (d, *J* = 5.3 Hz), 127.0, 126.8, 123.8, 111.4, 111.3, 102.9, 56.2; LC-MS (method A) R_T = 3.40 min (purity: 99.55%), *m/z* found: 425.02, calc.: 425.074 [M+H$^+$], HRMS: 425.089, mp=146-148°C.

***N*-(4-(5-(2-Chlorophenyl)-1,2,4-oxadiazol-3-yl)-3-methoxyphenyl)pyrimidine-4--carboxamide (45)**

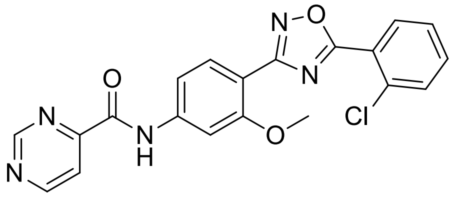

Prepared according to general procedure 5 from **16e** and pyrimidine-4-carboxylic acid. Precipitated solid was filtered directly from the reaction mixture and purified by column chromatography (SiO_2, $CHCl_3$/hexane 4:1) followed by maceration (2-PrOH/hexane 1:2); white solid, 0.14 g, 70.0%.

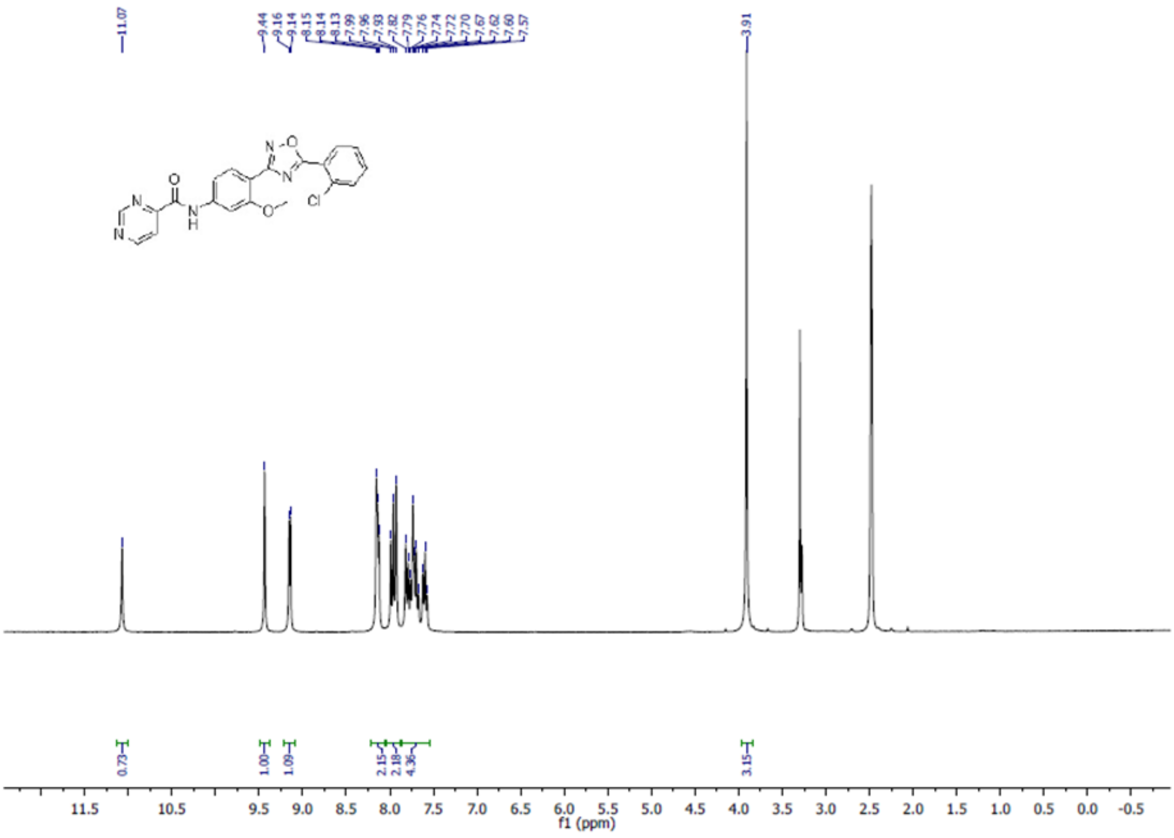

^{1}H NMR (300 MHz, DMSO-d_6), δ ppm: 11.07 (s, 1H, NH), 9.44 (s br. 1H), 9.15 (d, J = 5.1 Hz, 1H), 8.15-8.12 (m, 2H), 7.99-7.93 (m, 2H), 7.82-7.57 (m, 4H), 3.91 (s, 3H, CH_3);

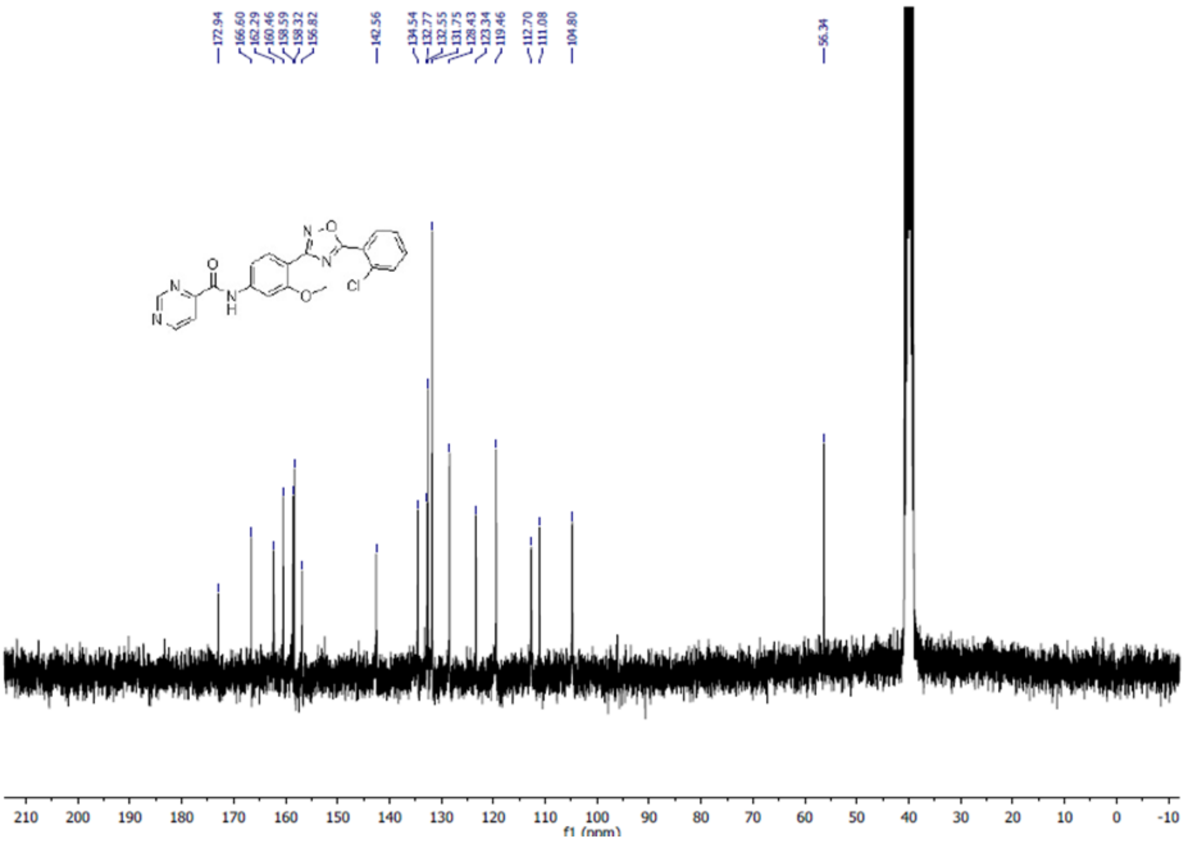

^{13}C NMR (75 MHz, DMSO-d_6), δ ppm: 172.9, 166.6, 162.3, 160.5, 158.6, 158.3, 156.8, 142.6, 134.5, 132.8, 132.6, 131.8 (2C), 128.4, 123.3, 119.5, 112.7, 111.1, 104.8, 56.3; LC-MS (method B) R_T = 3.23 min (purity: 97.79%), *m/z* found: 408.1, calc.: 408.078 [M+H$^+$], HRMS: 408.090, mp=217-218°C.

***N*-(4-(5-(2-Chlorophenyl)-1,2,4-oxadiazol-3-yl)-3-methoxyphenyl)thiazole-2-carboxamide (46)**

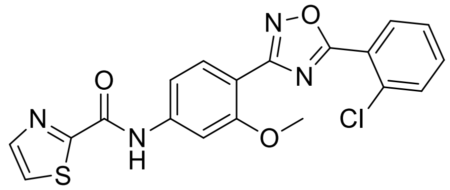

Prepared according to general procedure 5 from **16e** and 1,3-thiazole-2-carboxylic acid. Crude product isolated from the reaction mixture was purified by column chromatography (SiO_2, $CHCl_3$/hexane 4:1) and further purified by maceration (2-PrOH/hexane 1:2); white solid, 0.190 g, 92.7%.

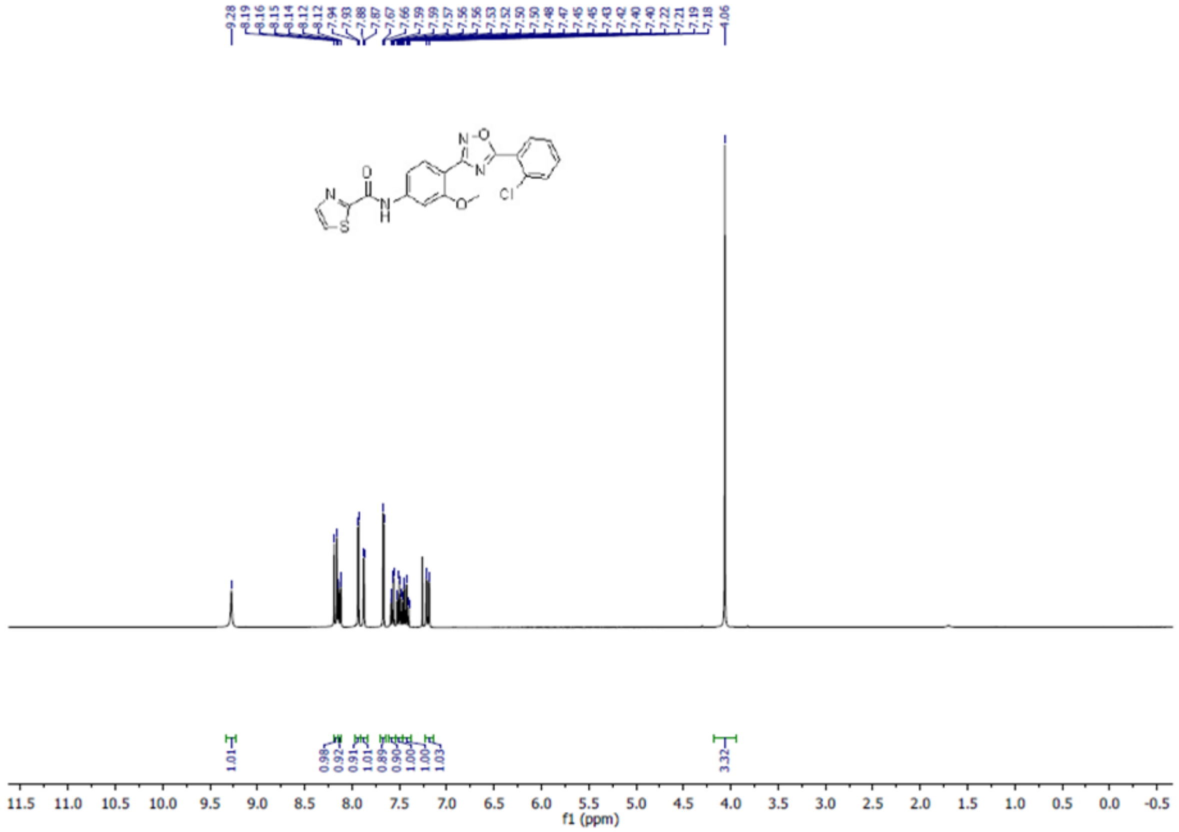

^{1}H NMR (300 MHz, $CDCl_3$), δ ppm: 9.28 (s br. 1H, NH), 8.17 (d, *J* = 8.4 Hz, 1H), 8.13 (dd, *J* = 7.6 and 1.8 Hz, 1H), 7.93 (d, *J* = 3.1 Hz, 1H), 7.87 (d, *J* = 1.9 Hz, 1H), 7.67 (d, *J* = 1.9 Hz, 1H), 7.56 (dd, *J* = 7.8 and 1.4 Hz, 1H), 7.50 (td, *J* = 7.6 and 1.8 Hz, 1H), 7.43 (td, *J* = 7.5 and 1.6 Hz, 1H), 7.20 (dd, *J* = 8.5 and 2.0 Hz, 1H), 4.06 (s, 3H, CH_3);

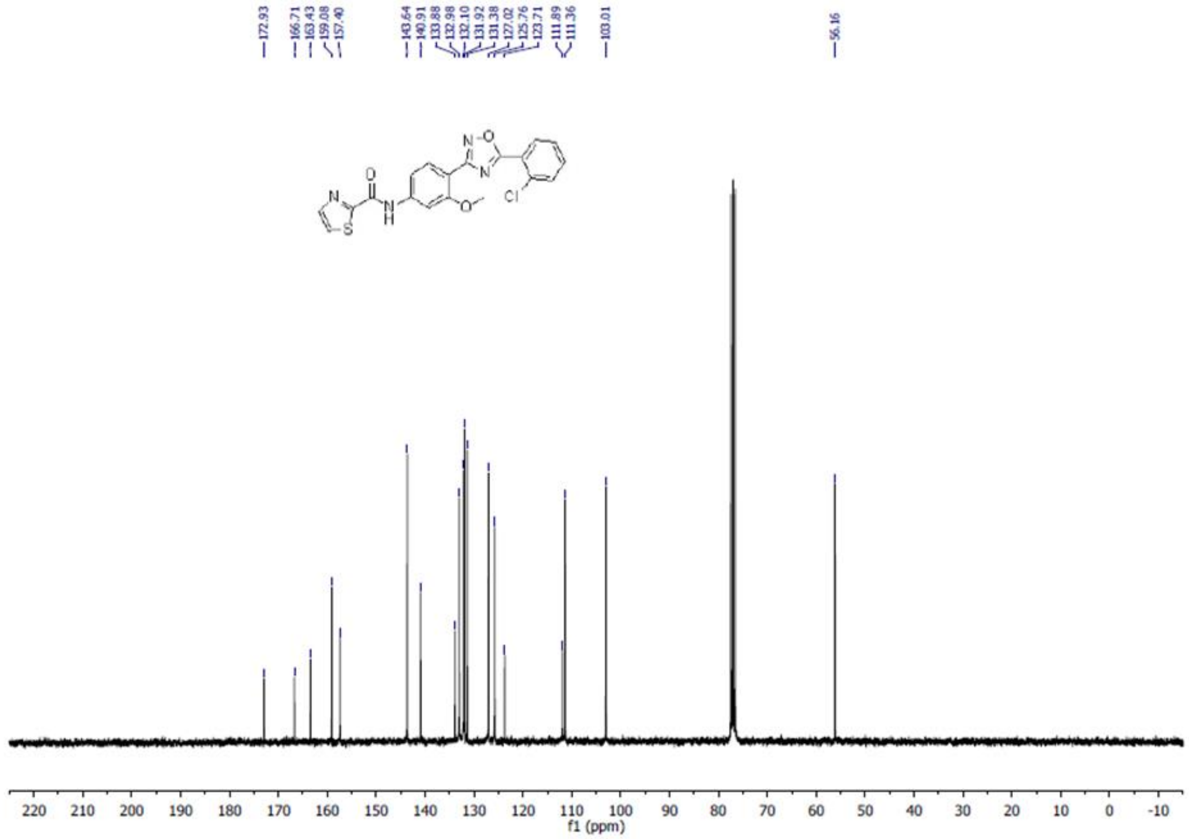

^{13}C NMR (75 MHz, $CDCl_3$), δ ppm: 172.9, 166.7, 163.4, 159.1, 157.4, 143.6, 140.9, 133.9, 133.0, 132.1, 131.9, 131.4, 127.0, 125.8, 123.7, 111.9, 111.4, 103.0, 56.2; LC-MS (method A) R_T = 3.49 min (purity: 99.16%), *m/z* found: 413.0, calc.: 413.040 [$M+H^+$], HRMS: 413.045, mp=153-155°C.

6-Fluoro-*N*-(4-(5-(2-fluorophenyl)-1,2,4-oxadiazol-3-yl)-3-methoxyphenyl)picolinamide (47)

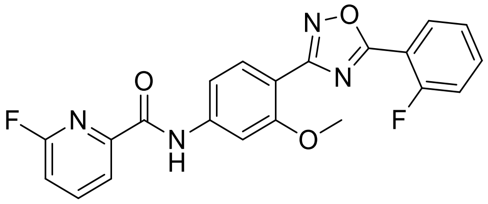

Prepared according to general procedure 5 from **16h** and 6-fluoro-pyridine-2-carboxylic acid. Precipitated solid was filtered directly from the reaction mixture and washed with MeCN. The filtrate was concentrated, and the rest of the product was isolated by column chromatography (SiO_2, AcOEt/hexane 1:1). The product was further purified by maceration (2-PrOH/hexane 1:2); white solid, 0.168 g, 78.1%.

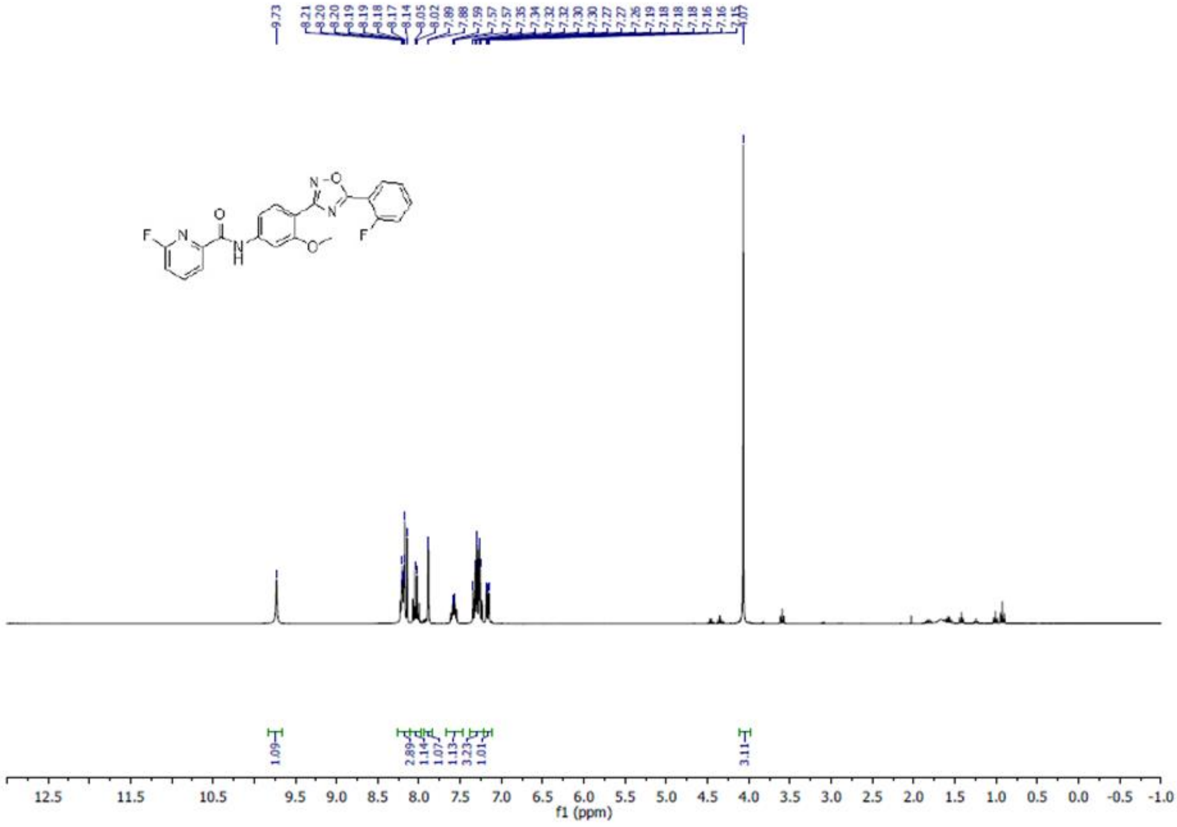

^{1}H NMR (300 MHz, $CDCl_3$), δ ppm: 9.73 (s br. 1H, NH), 8.21-8.14 (m, 3H), 8.03 (q, J = 7.6 Hz, 1H), 7.89 (d, J = 1.9 Hz, 1H), 7.62-7.54 (m, 1H), 7.35-7.24 (m, 3H), 7.17 (ddd, J = 8.1, 2.4 and 0.6 Hz, 1H), 4.07 (s, 3H, CH_3);

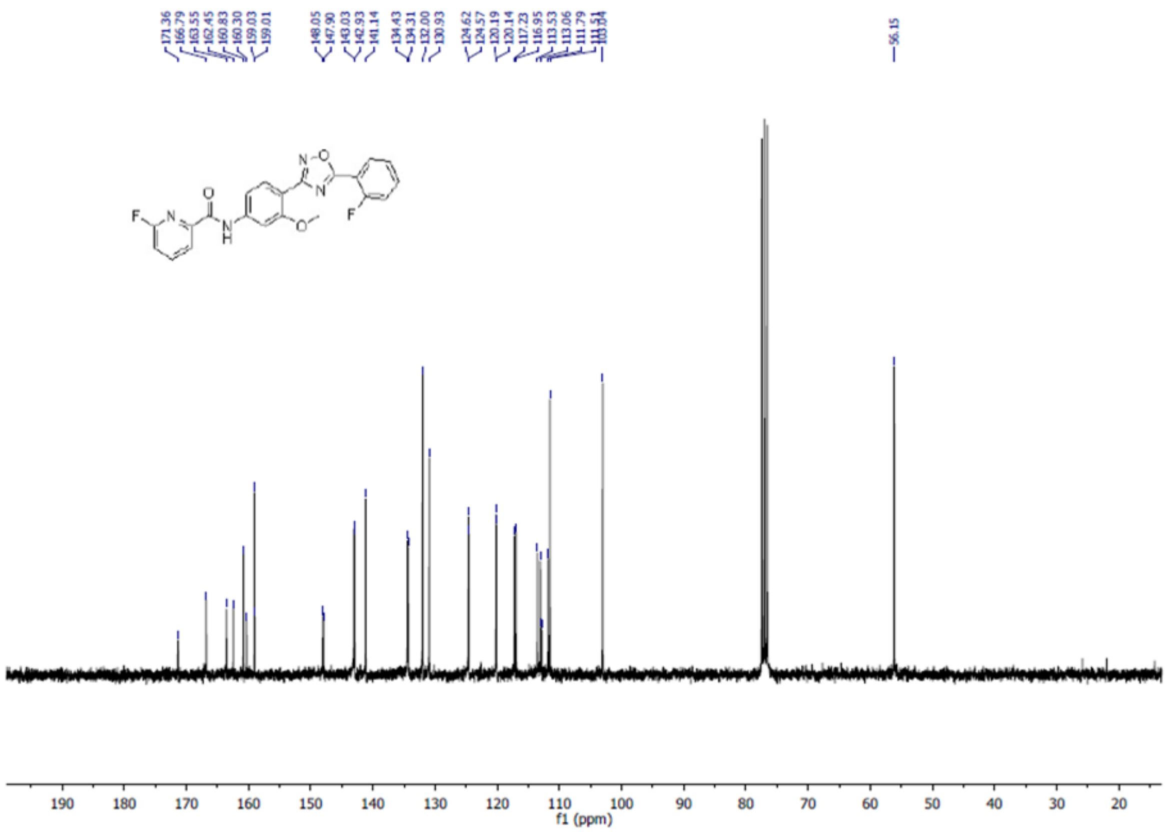

^{13}C NMR (75 MHz, $CDCl_3$), ppm: δ 171.4 (d, *J* = 4.5 Hz), 165.2 (d, *J* = 244.5 Hz), 160.8, 160.7 (d, *J* = 260.3 Hz), 160.3, 159.0, 148.0 (d, *J* = 10.7 Hz), 143.0 (d, *J* = 7.7 Hz), 141.1, 134.4 (d, *J* = 8.6 Hz), 132.0, 130.9, 124.6 (d, *J* = 3.8 Hz), 120.1 (d, *J* = 3.8 Hz), 117.1 (d, *J* = 20.9 Hz), 113.4 (d, *J* = 35.8 Hz), 112.9 (d, *J* = 11.4 Hz), 111.8, 111.5, 103.4, 56.2; LC-MS (method A) R_T = 3.43 min (purity: 100%), *m/z* found: 409.0, calc. 409.103 [M+H$^+$], HRMS: 409.116, mp=163-165°C.

3,6-Dichloro-*N*-(4-(5-(2-fluorophenyl)-1,2,4-oxadiazol-3-yl)-3-methoxyphenyl) picolinamide (48)

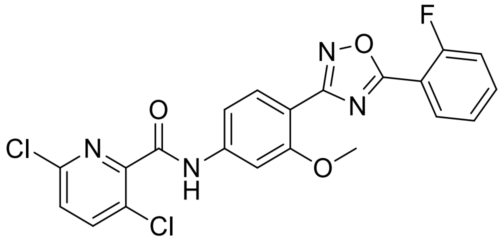

Prepared according to general procedure 4 from **16h** and 3,6-dichloropyridine-2-carbonyl chloride. Crude product was purified by column chromatography (SiO_2, $CHCl_3$) followed by maceration (2-PrOH/hexane 1:2); white solid, 0.18 g, 75.0%.

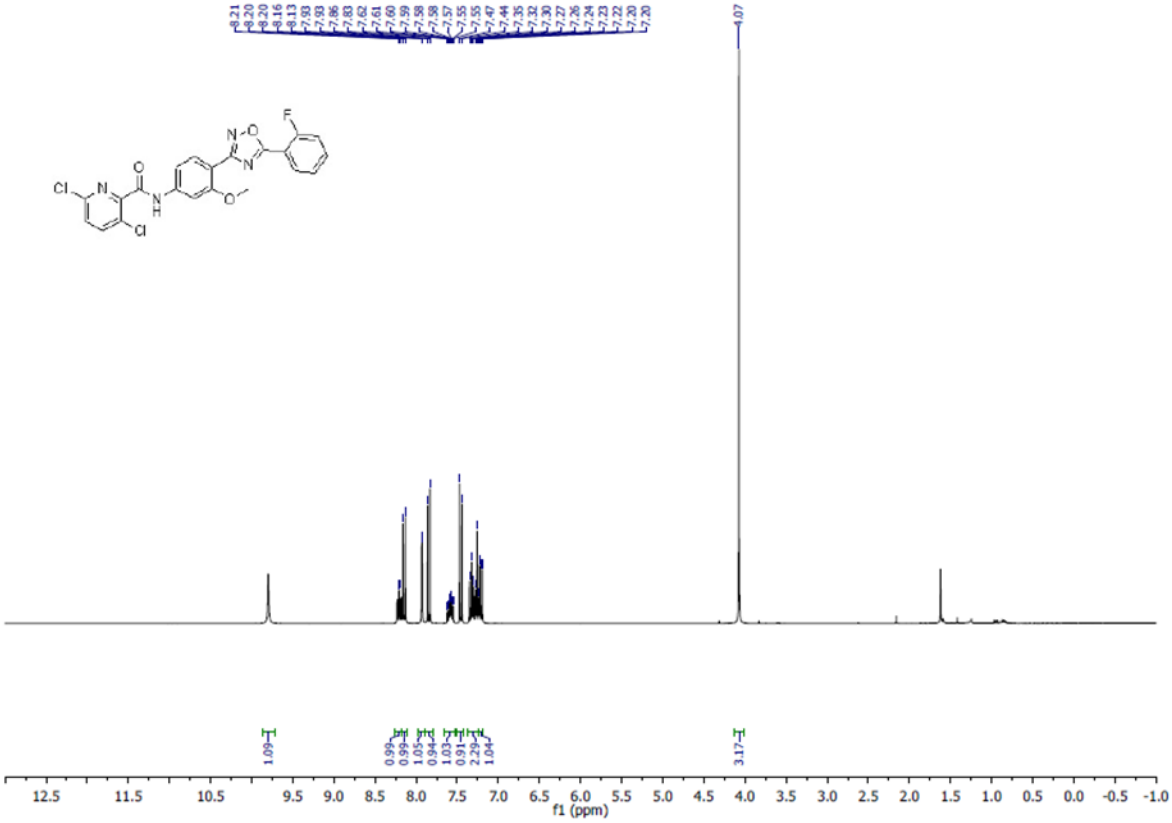

^{1}H NMR (300 MHz, $CDCl_3$), δ ppm: 9.80 (s br. 1H, NH), 8.20 (td, *J* = 7.6 and 1.8 Hz, 1H), 8.14 (d, *J* = 8.4 Hz, 1H), 7.93 (d, *J* = 1.9 Hz, 1H), 7.84 (d, *J* = 8.4 Hz, 1H), 7.62-7.55 (m, 1H), 7.45 (d, *J* = 8.4 Hz, 1H), 7.35-7.24 (m, 2H), 7.21 (dd, *J* = 8.5 and 2.0 Hz, 1H), 4.07 (s, 3H, CH_3);

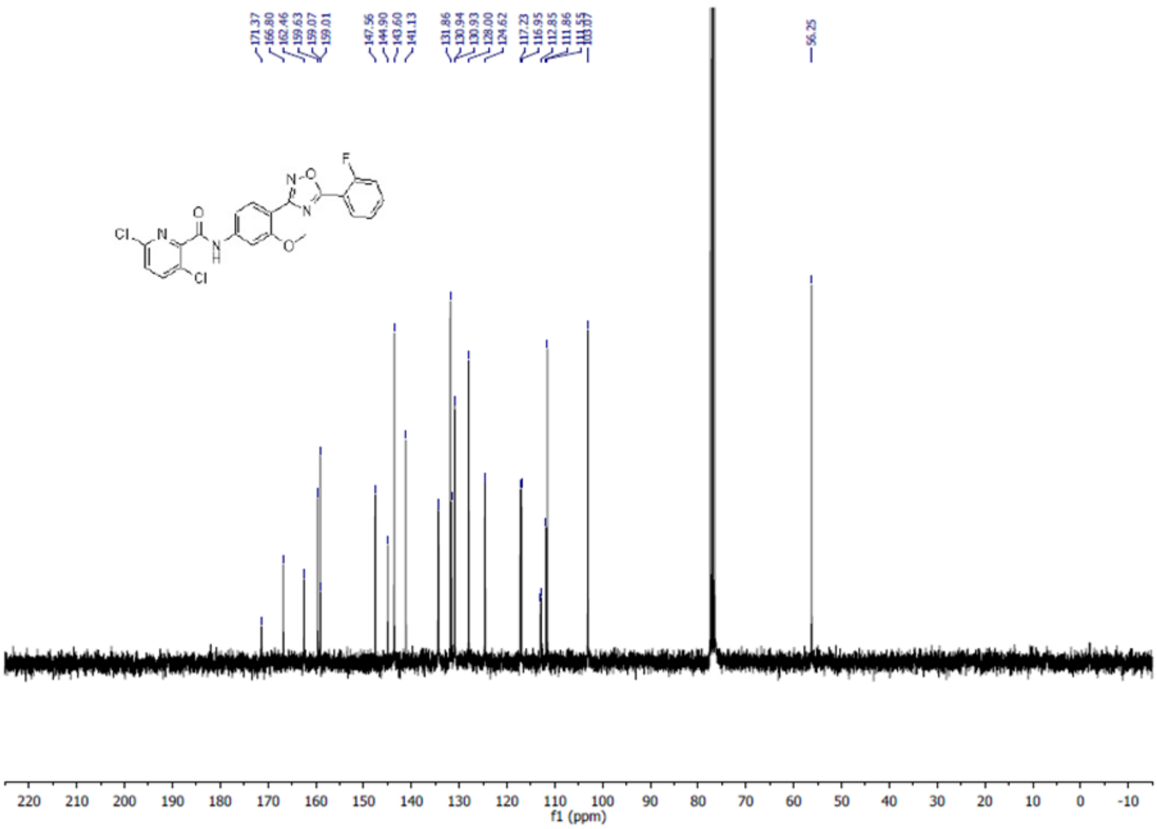

^{13}C NMR (75 MHz, $CDCl_3$), δ ppm: 171.4 (d, *J* = 4.5 Hz), 166.8, 160.8 (d, *J* = 260.4 Hz), 159.6, 159.1, 147.6, 144.9, 143.6, 141.1, 134.4 (d, *J* = 8.6 Hz), 131.9, 131.6, 130.9 (d, *J* = 0.9 Hz), 128.0, 124.6 (d, *J* = 3.8 Hz), 117.0 (d, *J* = 20.9 Hz), 112.9 (d, *J* = 11.4 Hz), 111.9, 111.6, 103.1, 56.3; LC-MS (method A) R_T = 3.52 min (purity: 100%), *m/z* found: 460.62, calc.: 459.035 $[M+H^+]$, HRMS: 459.049, mp=186-188°C.

N-(3-Chloro-4-(5-phenyl-1,2,4-oxadiazol-3-yl)phenyl)picolinamide (49)

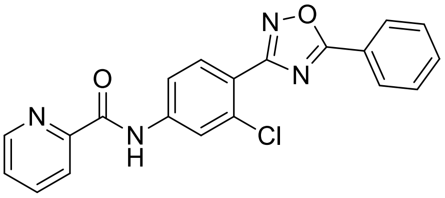

Starting aniline **16j** (0.12 g, 0.442 mmol, 1 eq) was dissolved in THF (5 mL) and TEA (0.174 g, 0.24 mL, 1.77 mmol, 4.0 eq) was added dropwise. 2-Pyridinecarbonyl chloride, hydrochloride (0.12 g, 0.66 mmol, 1.5 eq) was added in few portions over 5 minutes. After stirring overnight in rt, the solvent was removed in vacuo. Water was then added to residual oil, and after stirring, precipitated solid was filtered, dried and purified by column chromatography (SiO_2, $CHCl_3$/MeOH 99:1) followed by maceration (2-PrOH/hexane 1:2); white solid, 0.12 g, 72.3%.

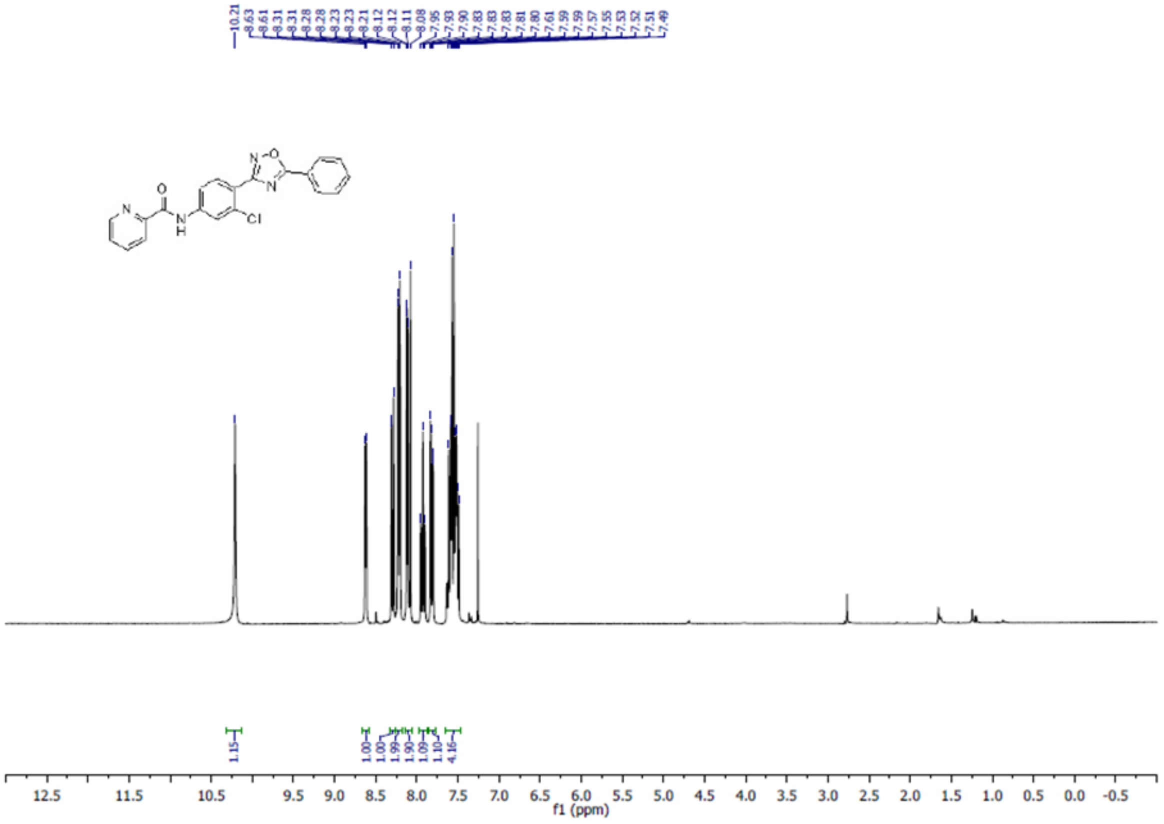

^{1}H NMR (300 MHz, $CDCl_3$), δ ppm: 10.21 (s br. 1H, NH), 8.62 (dt, *J* = 4.8 and 0.8 Hz, 1H), 8.30 (dd, *J* = 7.8 and 0.8 Hz, 1H), 8.23-8.20 (m, 2H), 8.12-8.08 (m, 2H), 7.90 (ddd, *J* = 7.8, 1.6 and 0.6 Hz, 1H), 7.82 (dd, *J* = 8.6 and 2.0 Hz, 1H), 7.64-7.49 (m, 4H);

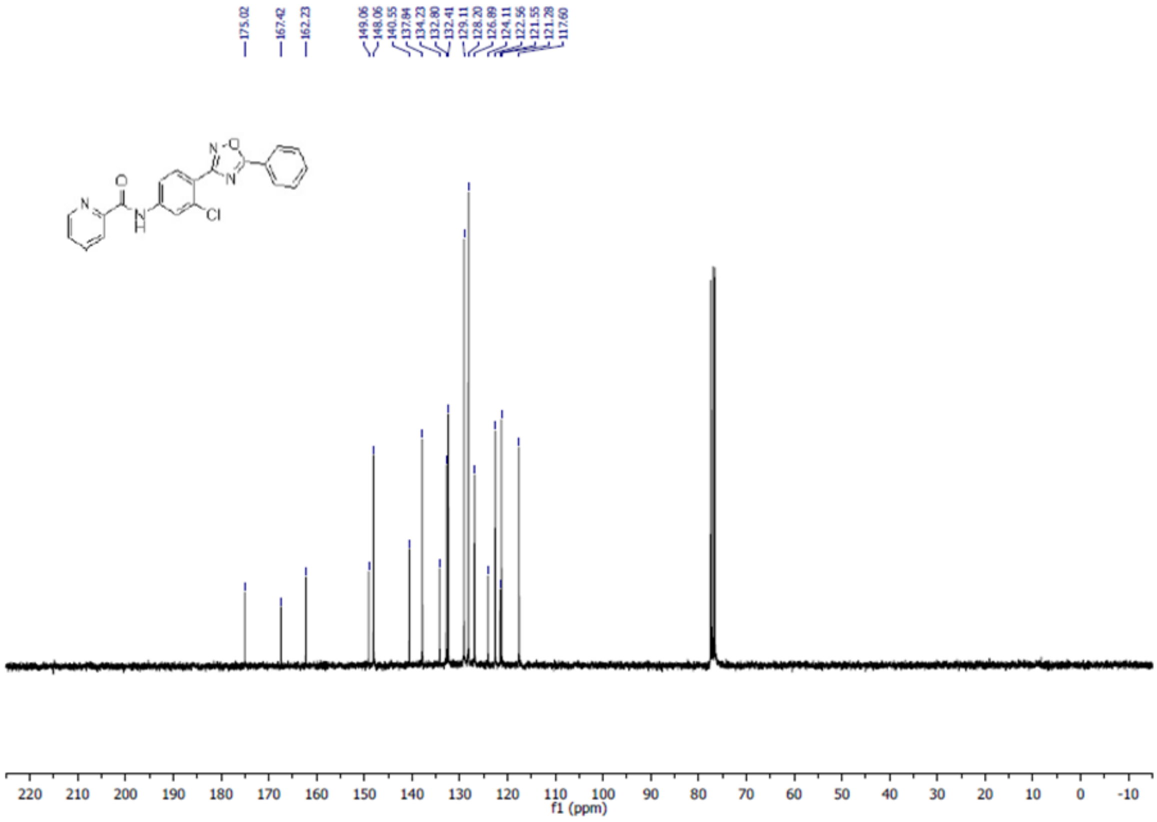

^{13}C NMR (75 MHz, $CDCl_3$), δ ppm: 175.0, 167.4, 162.2, 149.1, 148.1, 140.6, 137.8, 134.2, 132.8, 132.4, 129.1 (2C), 128.2 (2C), 126.9, 124.1, 122.6, 121.6, 121.3, 117.6; LC-MS (method A) R_T = 3.80 min (purity: 97.33%), *m/z* found: 377.4, calc.: 377.073 [M+H$^+$], HRMS: 377.081, mp=159-160˚C.

N-(3-Chloro-4-(5-(2-chlorophenyl)-1,2,4-oxadiazol-3-yl)phenyl)-6-fluoropicolinamide (50)

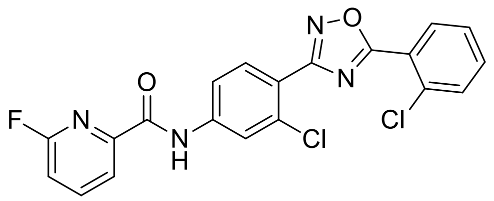

Prepared according to general procedure 5 from **16k** and 6-fluoro-pyridine-2-carboxylic acid. Precipitated solid was filtered directly from the reaction mixture, washed with MeCN, dried, and purified by maceration (2-PrOH/hexane 1:2); white solid, 0.120 g, 85.7%.

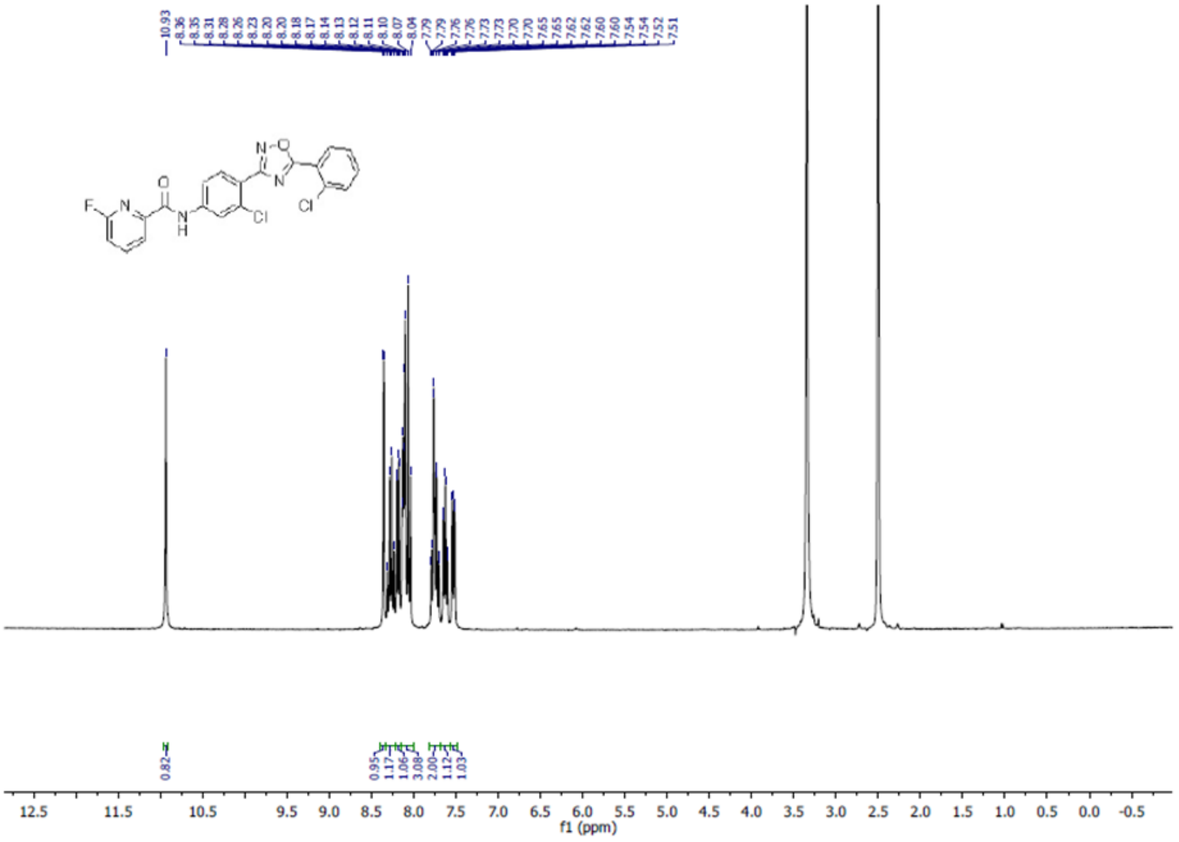

^{1}H NMR (300 MHz, DMSO-d$_6$), δ ppm: 10.93 (s br. 1H, NH), 8.35 (d, J = 1.8 Hz, 1H), 8.25 (q, J = 7.9 Hz, 1H), 8.19 (dd, J = 7.6 and 1.3 Hz, 1H), 8.14-8.04 (m, 3H), 7.79-7.70 (m, 2H), 7.63 (td, J = 7.7 and 1.6 Hz, 1H), 7.53 (dd, J = 8.2 and 1.8 Hz, 1H);

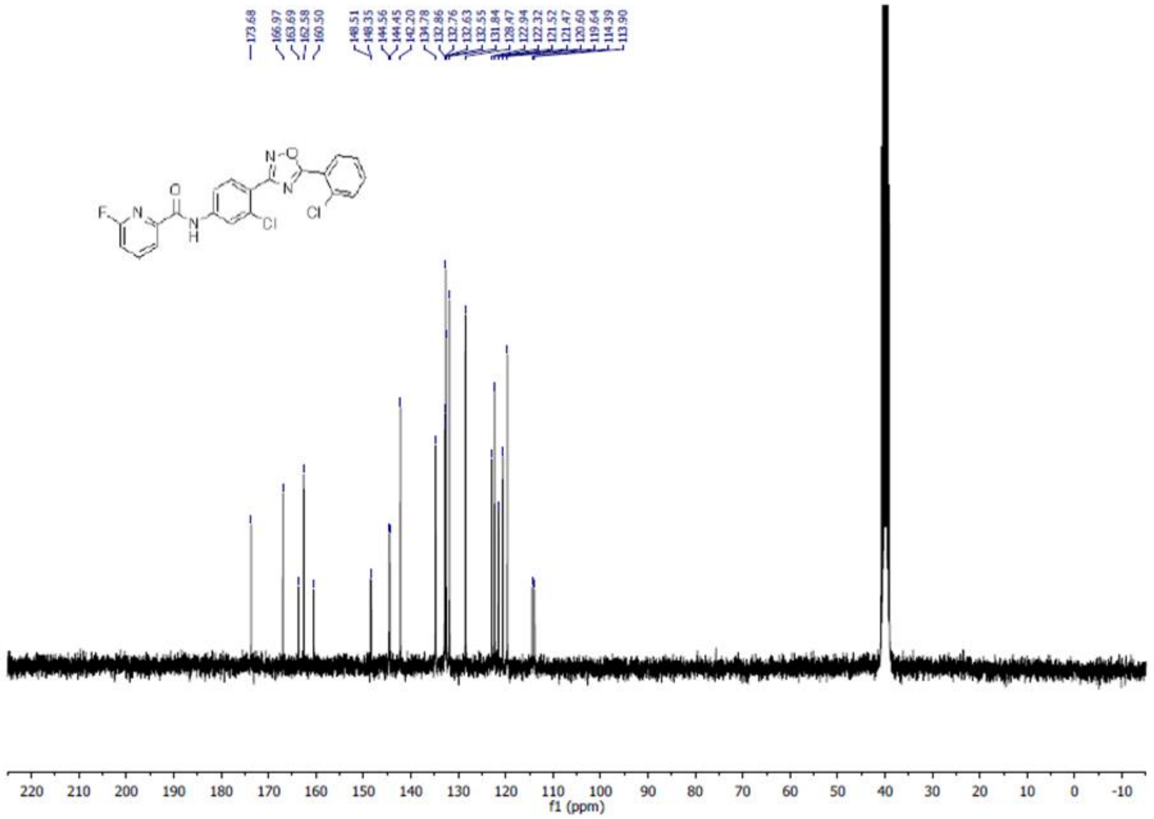

^{13}C NMR (75 MHz, DMSO-d_6), δ ppm: 173.7, 167.0, 162.6, 162.1 (d, *J* = 240.9 Hz), 148.4 (d, *J* = 11.6 Hz), 144.5 (d, *J* = 8.1 Hz), 142.2, 134.8, 132.9, 132.8, 132.6, 132.5, 131.8, 128.5, 122.9, 122.3, 121.5 (d, *J* = 3.5 Hz), 120.6, 119.6, 114.1 (d, *J* = 36.4 Hz); LC-MS (method A) R_T = 3.91 min (purity: 97.54%), *m/z* found: 430.9, calc.: 429.024 [$M+H^+$], HRMS: 429.036, mp=198-200˚C.

6-Chloro-*N*-(3-chloro-4-(5-(2-chlorophenyl)-1,2,4-oxadiazol-3-yl)phenyl)picolinamide (51)

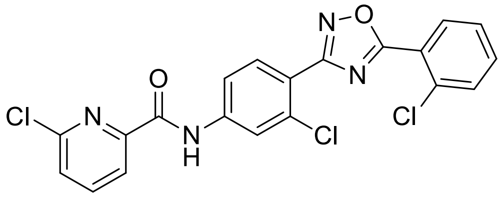

Prepared according to general procedure 5 from **16k** and 6-chloro-pyridine-2-carboxylic acid. Precipitated solid was filtered from the reaction mixture, washed with MeCN, dried, and purified by maceration (2-PrOH/hexane 1:2); white solid, 0.113 g, 77.9%.

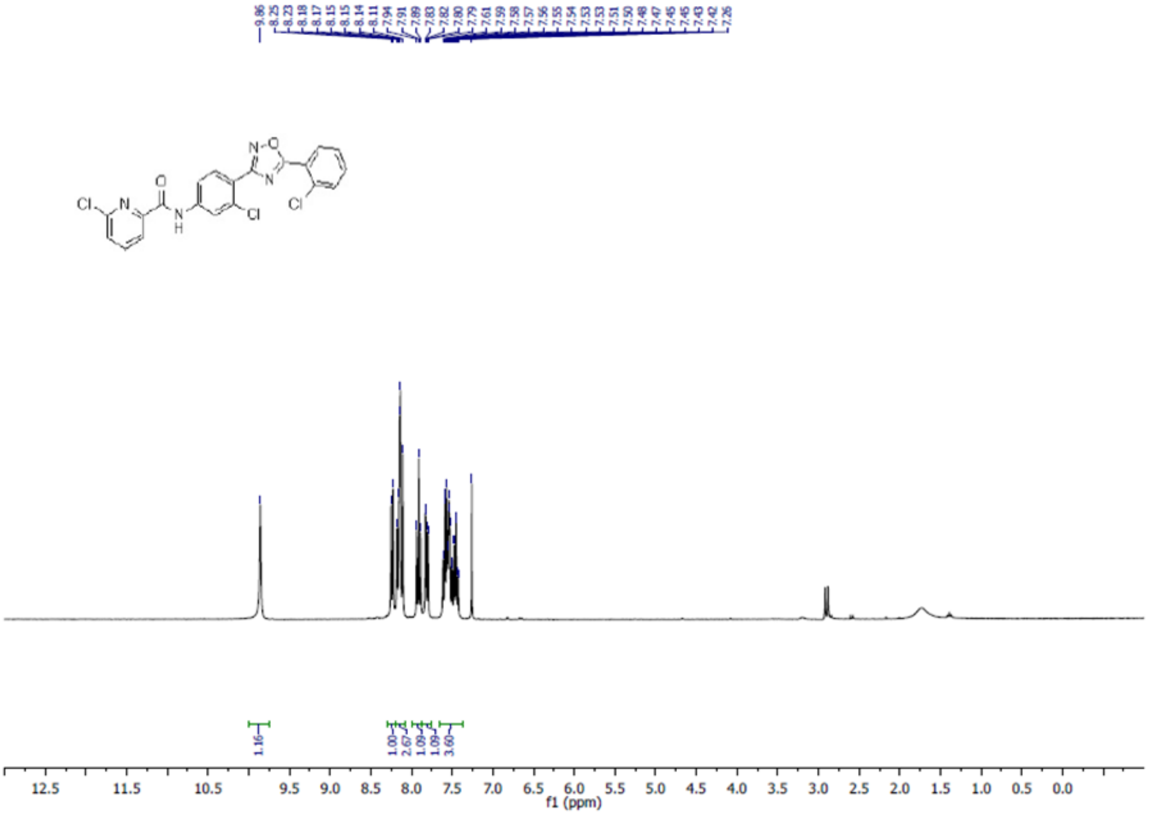

^{1}H NMR (300 MHz, $CDCl_3$), δ ppm: 9.86 (s br. 1H, NH), 8.24 (d, J = 8.2 Hz, 1H), 8.18-8.11 (m, 3H), 7.91 (t, J = 7.8 Hz, 1H), 7.81 (dd, J = 8.6 and 2.1 Hz, 1H), 7.61-7.42 (m, 4H);

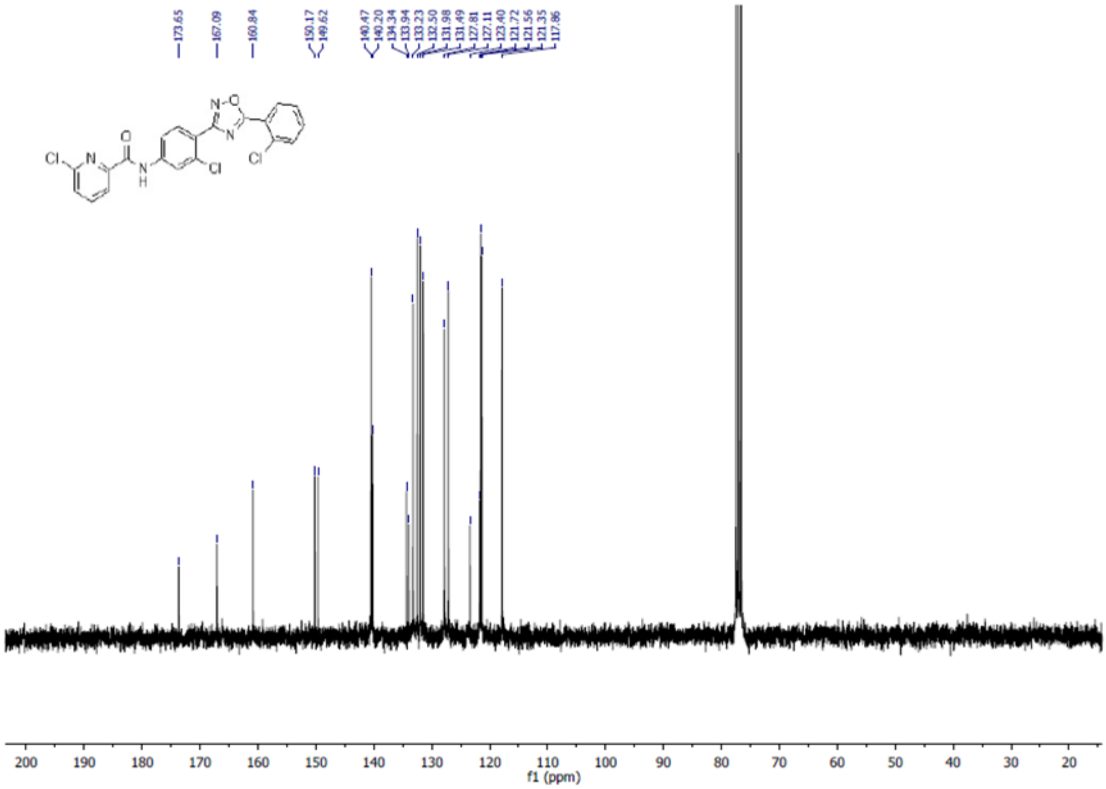

^{13}C NMR (75 MHz, $CDCl_3$), δ ppm: 173.7, 167.1, 160.8, 150.2, 149.6, 140.5, 140.2, 134.3, 133.9, 133.2, 132.5, 132.0, 131.5, 127.8, 127.1, 123.4, 121.7, 121.6, 121.4, 117.9; LC-MS (method A) R_T = 4.07 min (purity: 96.80%), *m/z* found: 445.6, calc.: 444.995 [$M+H^+$], HRMS: 444.994, mp=193-195°C.

N-(3-Chloro-4-(5-(2-chlorophenyl)-1,2,4-oxadiazol-3-yl)phenyl)picolinamide (52)

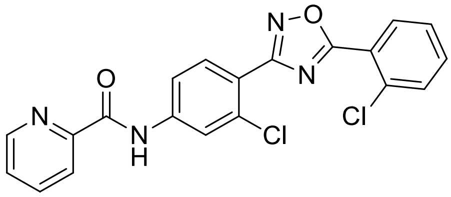

Prepared according to general procedure 2 from **26c** and 2-chlorobenzoyl chloride. Crude product was purified by column chromatography (SiO_2, $CHCl_3$) followed by maceration (2-PrOH/hexane 1:2); white solid, 0.18 g, 85.7%.

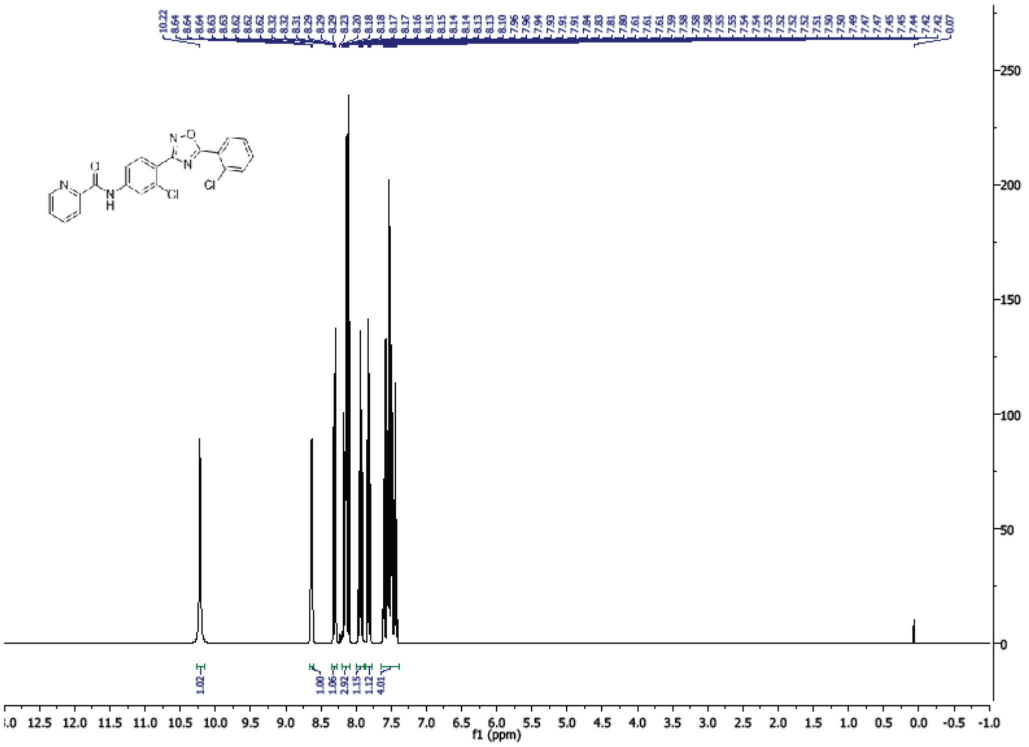

^{1}H NMR (300 MHz, $CDCl_3$), δ ppm: 10.22 (s br. 1H, NH), 8.63 (ddd, J = 4.8, 1.6 and 0.9 Hz, 1H), 8.30 (dt, J = 7.8 and 1.0 Hz, 1H), 8.18-8.10 (m, 3H), 7.94 (td, J = 7.7 and 1.7 Hz, 1H), 7.82 (dd, J = 8.6 and 2.2 Hz, 1H), 7.62-7.42 (m, 4H).

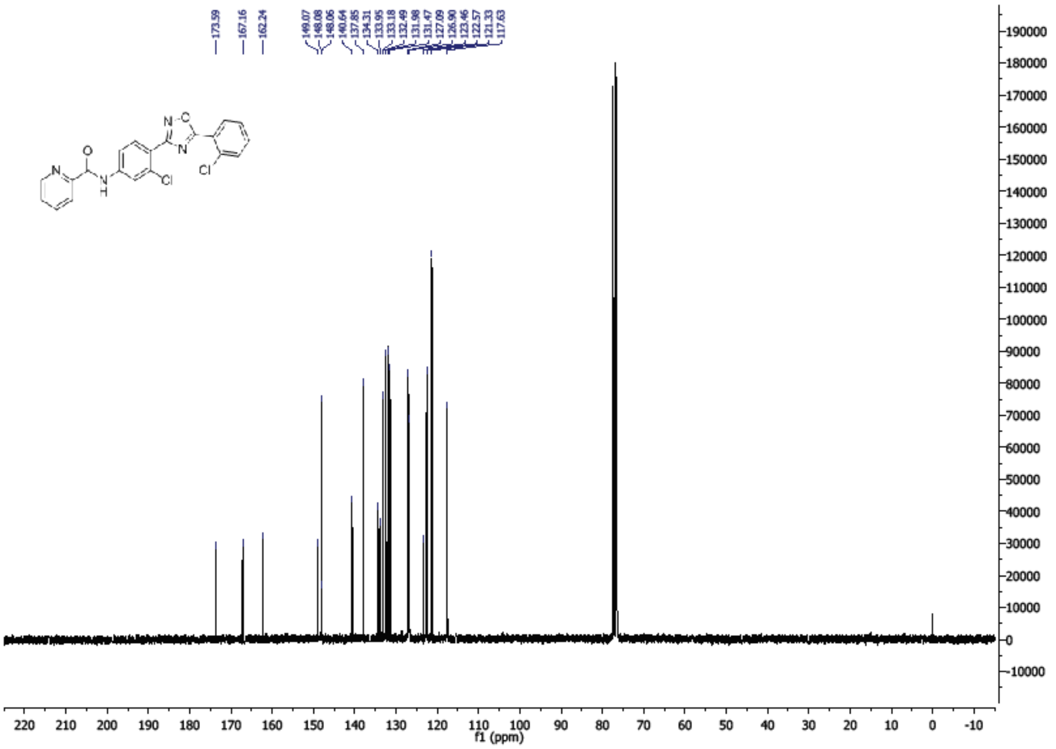

^{13}C NMR (75 MHz, $CDCl_3$), δ ppm: 173.6, 167.2, 162.2, 149.1, 148.1, 140.6, 137.8, 134.3, 133.9, 133.2, 132.5, 132.0, 131.5, 127.1, 126.9, 123.5, 122.6, 121.3, 121.2, 117.6; LC-MS (method A) R_T = 3.89 min (purity: 97.60%), *m/z* found: 411.45, calc.: 411.034 [M+H$^+$], HRMS: 411.035, mp=159-161˚C.

***N*-(3-Chloro-4-(5-(2-chloro-4-fluorophenyl)-1,2,4-oxadiazol-3-yl)phenyl)picolinamide**

(53)

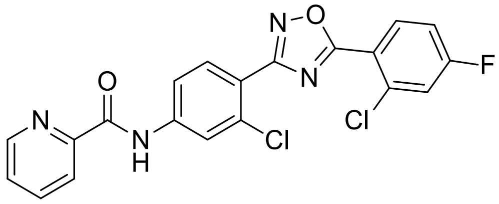

Prepared from **26c** and 2-chloro-4-fluorobenzoyl chloride according to general procedure 2. Crude product was purified by column chromatography (SiO_2, $CHCl_3$) followed by maceration (2-PrOH/hexane 1:2); white solid, 0.15 g, 68.2%.

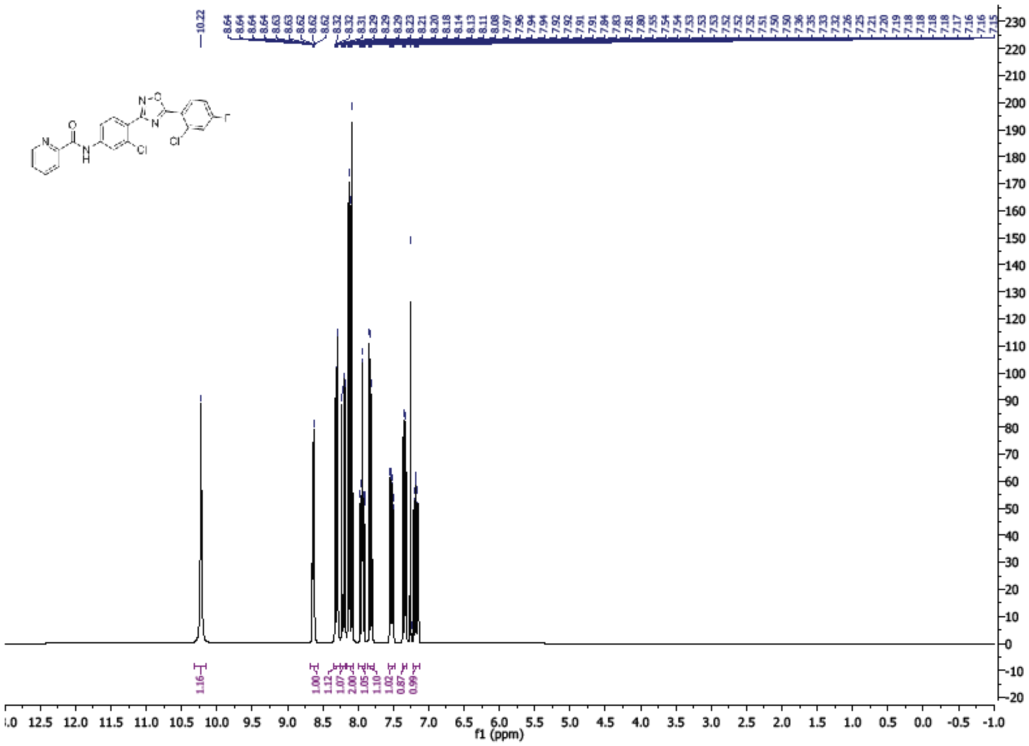

^{1}H NMR (300 MHz, $CDCl_3$), δ ppm: 10.23 (s br. 1H, NH), 8.63 (d br., J = 4.8 Hz, 1H), 8.30 (d br., J = 7.8 Hz, 1H), 8.19 (dd, J = 8.8 and 6.0 Hz, 1H), 8.13 (d, J = 2.0 Hz, 1H), 8.10 (d, J = 8.5 Hz, 1H), 7.94 (td, J = 7.7 and 1.6 Hz, 1H), 7.82 (dd, J = 8.5 Hz, 1H), 7.53 (ddd, J = 7.5, 4.8 and 1.0 Hz, 1H), 7.34 (dd, J = 8.4 and 2.5 Hz, 1H), 7.17 (ddd, J = 8.9, 7.5 and 2.5 Hz, 1H);

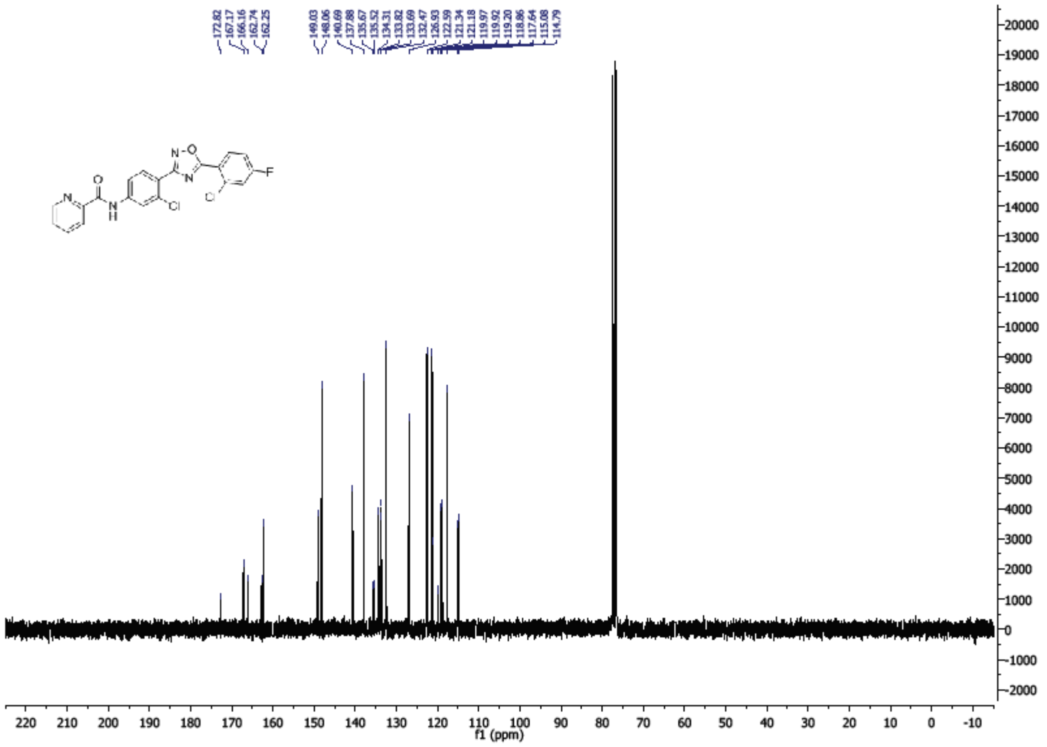

^{13}C NMR (75 MHz, $CDCl_3$), δ ppm: 172.8, 167.2, 164.5 (d, J = 257.9 Hz), 162.3, 149.0, 148.1, 140.7, 137.8, 135.6 (d, J = 10.8 Hz), 134.3, 133.7 (d, J = 9.7 Hz), 132.5, 126.9, 122.6, 121.3, 121.2, 119.9 (d, J = 3.6 Hz), 119.0 (d, J = 25.0 Hz), 117.6, 114.9 (d, J = 21.8 Hz); LC-MS (method A) R_T=3.96 min (purity: 97.80%), *m/z* found: 429.39, calc.: 429.024 $[M+H^+]$, HRMS: 429.019, mp=189-190°C.

***N*-(3-Chloro-4-(5-(2-methoxyphenyl)-1,2,4-oxadiazol-3-yl)phenyl)picolinamide** (**54**)

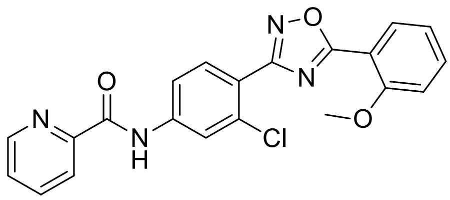

Prepared from **26c** and 2-methoxybenzoyl chloride according to general procedure 2. Crude product was purified by column chromatography (SiO_2, $CHCl_3$/hexane 1:1 to $CHCl_3$/MeOH 99:1 and then Al_2O_3, AcOEt/hexane 1:2) followed by maceration (2-PrOH/hexane/$CHCl_3$ 1:1:1); white solid, 0.13 g, 41.9%.

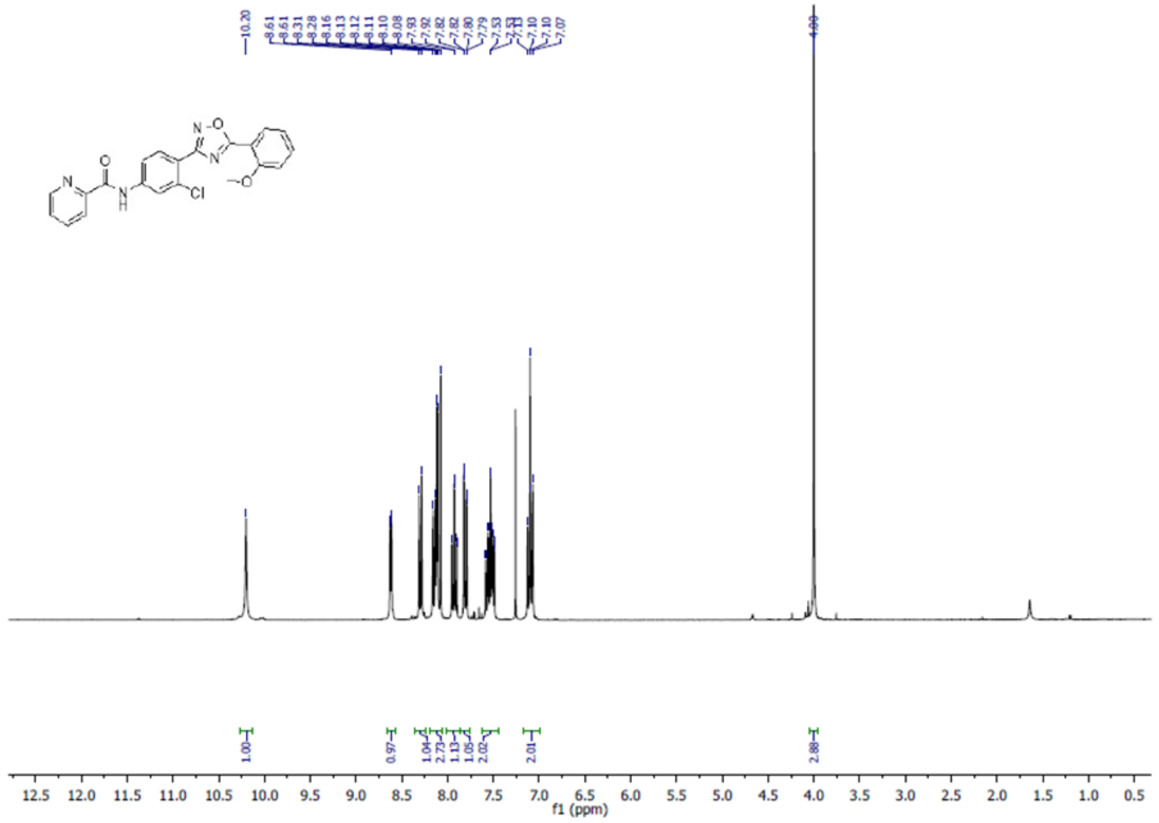

^{1}H NMR (300 MHz, $CDCl_3$), δ ppm: 10.20 (s br. 1H, NH), 8.62 (ddd, *J* = 4.8, 1.6 and 0.9 Hz, 1H), 8.30 (dt, *J* = 7.8 and 1.0 Hz, 1H), 8.16-8.09 (m, 3H), 7.92 (td, *J* = 7.7 and 1.7 Hz, 1H), 7.81 (dd, *J* = 8.6 and 2.1 Hz, 1H), 7.59-7.49 (m, 2H), 7.13-7.07 (m, 2H), 4.00 (s, 3H, CH_3);

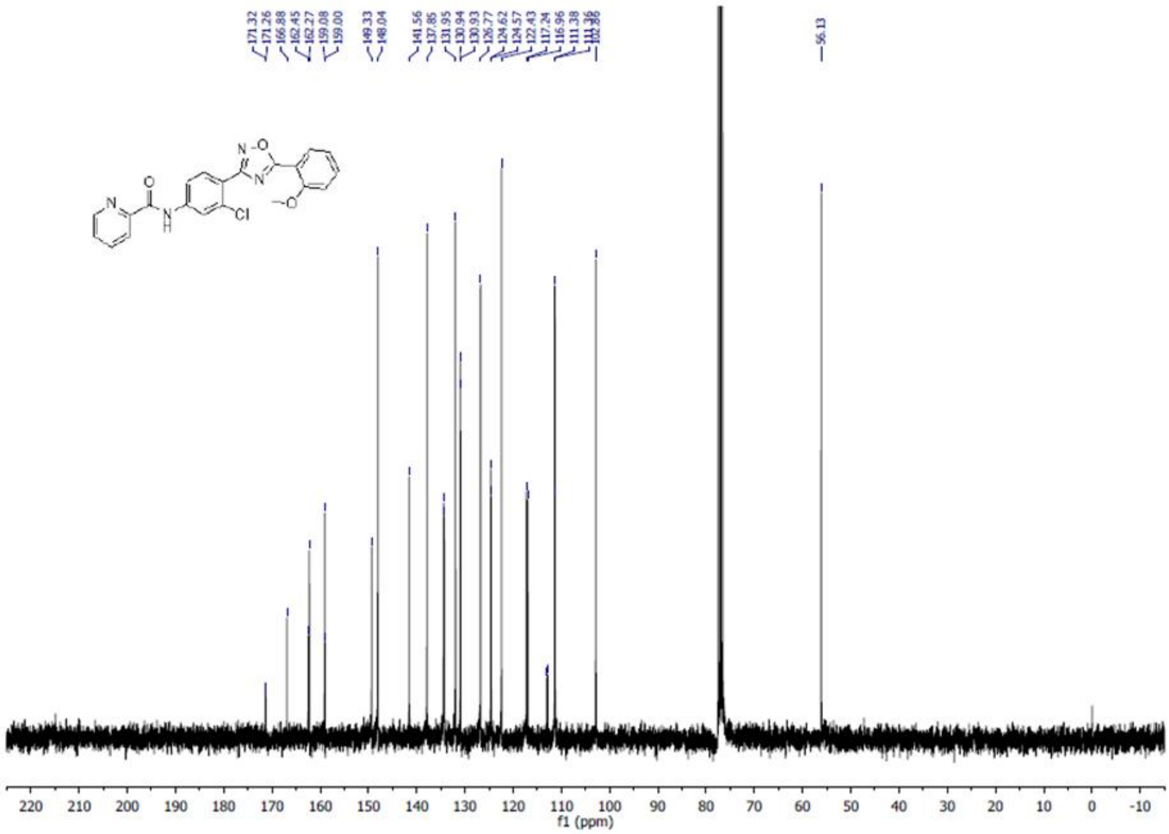

^{13}C NMR (75 MHz, $CDCl_3$), δ ppm: 174.4, 166.8, 162.2, 158.6, 149.1, 148.1, 140.4, 137.8, 134.2, 134.1, 132.4, 131.7, 126.9, 122.6, 121.8, 121.2, 120.8, 117.6, 113.3, 112.1, 56.1; LC-MS (method A) R_T = 3.63 min (purity 94.70%), m/z = 407.89, calc.: 407.084 [M+H$^+$], HRMS: 407.091, mp=171-173°C.

_N_-(3-Fluoro-4-(5-phenyl-1,2,4-oxadiazol-3-yl)phenyl)picolinamide (**55**)

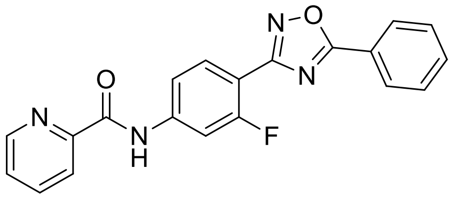

Prepared from **26d** and benzoyl chloride according to general procedure 2. Crude product was purified by column chromatography (SiO_2, $CHCl_3$/MeOH 49:1) followed by maceration (2-PrOH/hexane 1:2); white solid, 0.16 g, 80.4%.

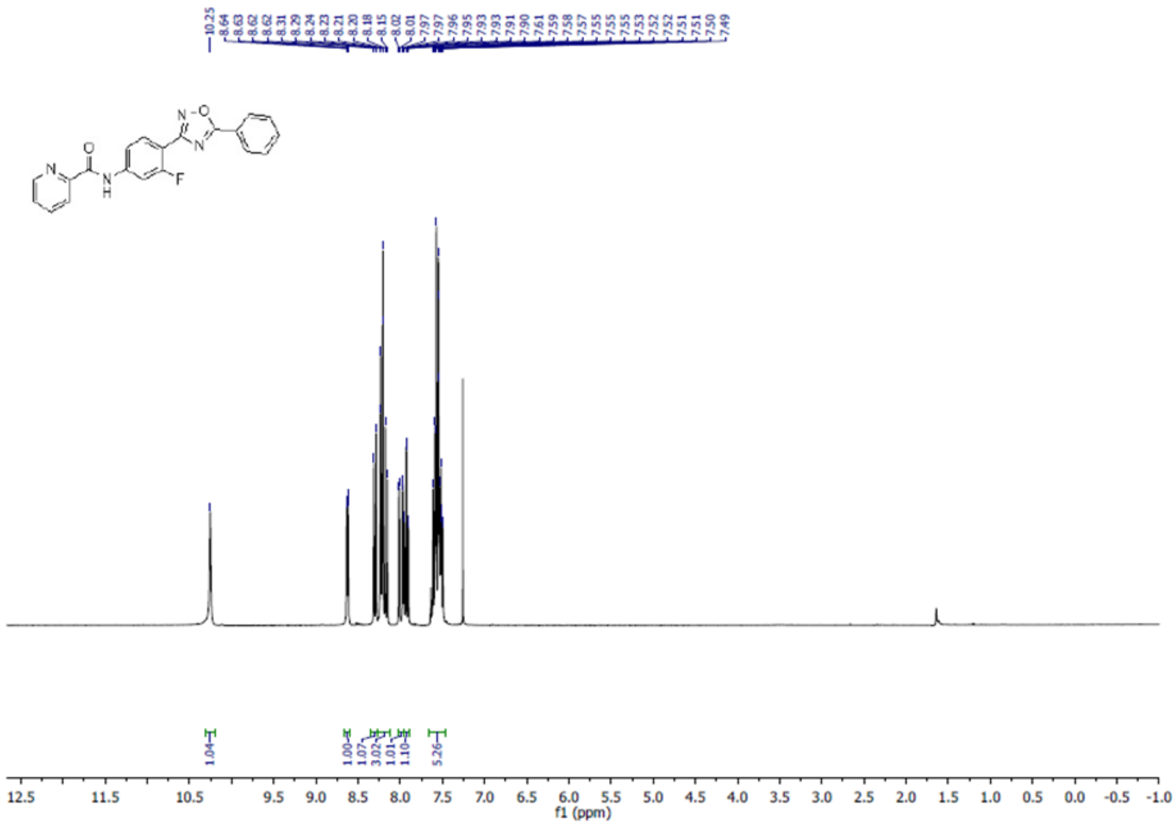

^{1}H NMR (300 MHz, $CDCl_3$), δ ppm: 10.25 (s br. 1H, NH), 8.63 (ddd, J = 4.8, 1.6 and 0.9 Hz, 1H), 8.30 (dt, J = 7.8 and 1.0 Hz, 1H), 8.24-8.15 (m, 3H), 7.99 (dd, J = 12.5 and 2.1 Hz, 1H), 7.93 (td, J = 7.7 and 1.7, 1H), 7.64-7.49 (m, 5H);

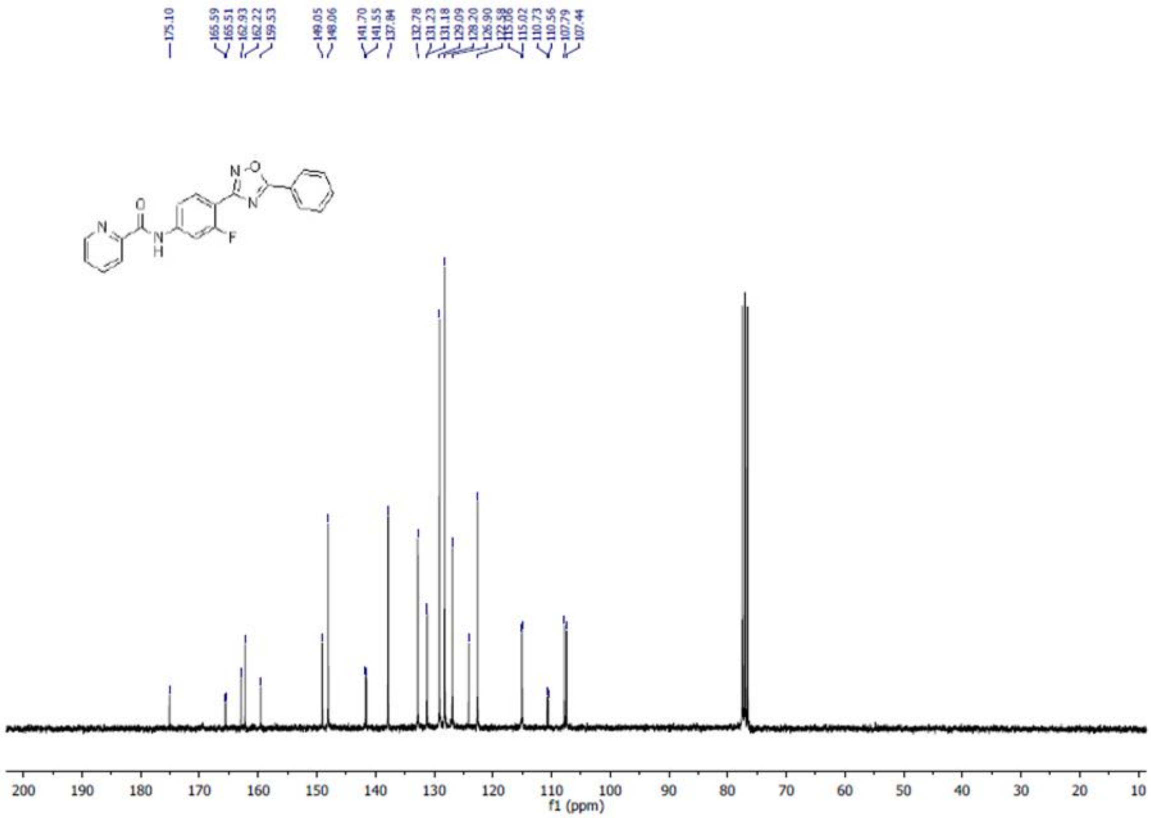

^{13}C NMR (75 MHz, $CDCl_3$), δ ppm: 175.0, 165.5 (d, J = 5.9 Hz), 162.2, 161.2 (d, J = 256.5 Hz), 149.1, 148.1, 141.6 (d, J = 11.3 Hz), 137.8, 132.8, 131.2 (d, J = 3.7 Hz), 129.1 (2C), 128.2 (2C), 126.9, 124.1, 122.6, 115.0 (d, J = 3.2 Hz), 110.6 (d, J = 12.8 Hz), 107.6 (d, J = 26.5 Hz); LC-MS (method A) R_T = 3.65 min (purity: 96.81%), *m/z* found: 361.3, calc.: 361.102 [M+H$^+$], HRMS: 361.111, mp=206-207°C.

***N*-(4-(5-(2-Chlorophenyl)-1,2,4-oxadiazol-3-yl)-3-fluorophenyl)picolinamide** (**56**)

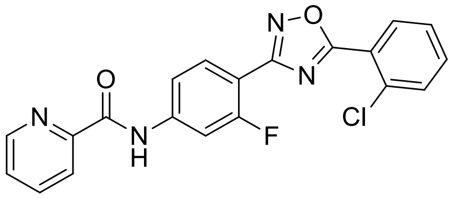

Prepared from **26d** and 2-chlorobenzoyl chloride according to general procedure 2. Crude product was purified by column chromatography (SiO_2, $CHCl_3$/MeOH 49:1) followed by maceration (2-PrOH/hexane 1:2); white solid, 0.17 g, 78.7%.

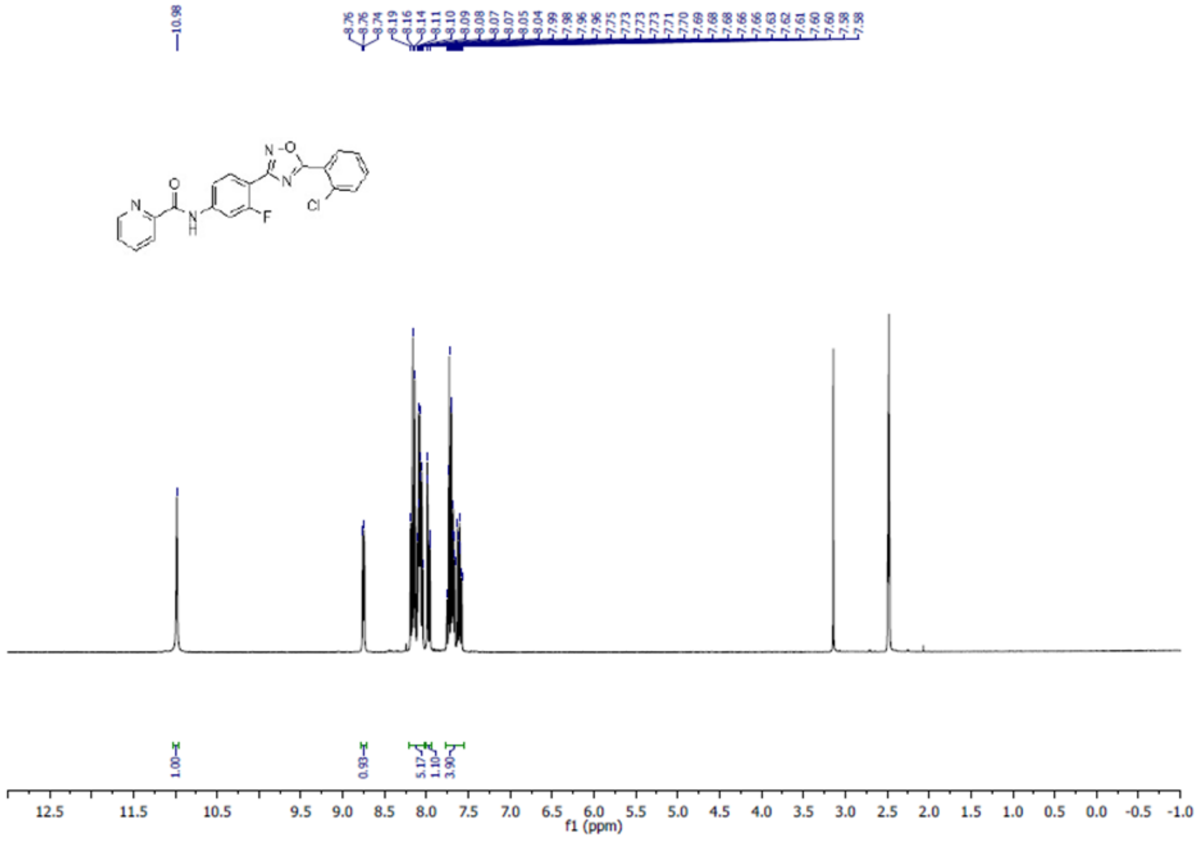

^{1}H NMR (300 MHz, DMSO-d_6) δ 10.98 (s br. 1H, NH), 8.75 (ddd, J = 4.8, 1.6 and 0.9 Hz, 1H), 8.19-8.04 (m, 5H), 7.98 (dd, J = 8.6 and 2.0 Hz, 1H), 7.75-7.58 (m, 4H);

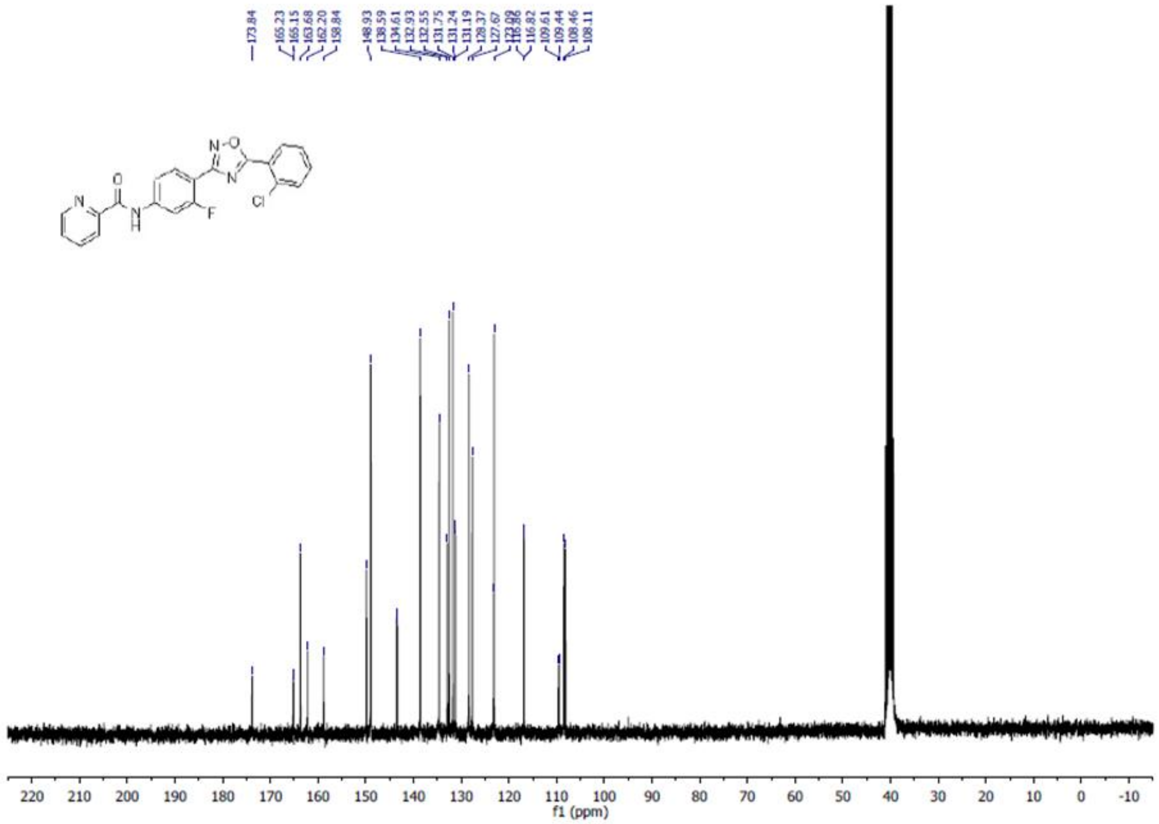

^{13}C NMR (75 MHz, DMSO-d$_6$) δ 172.8, 165.2 (d, *J* = 8.6 Hz), 163.7, 160.5 (d, *J* = 253.6 Hz), 149.9, 148.9, 143.4 (d, *J* = 11.4 Hz), 138.6, 134.6, 132.9, 132.6, 131.8, 131.2 (d, *J* = 3.5 Hz), 128.4, 127.7, 123.2, 123.1, 116.8 (d, *J* = 3.0 Hz), 109.5 (d, *J* = 12.9 Hz), 108.3 (d, *J* = 26.1 Hz); LC-MS (method B) R_T = 3.77 min (purity 97.7%), m/z = 395.1, calc.: 395.063 [M+H$^+$], HRMS: 395.064, mp=200-202°C.

***N*-(3-Fluoro-4-(5-(2-methoxyphenyl)-1,2,4-oxadiazol-3-yl)phenyl)picolinamide** (**57**)

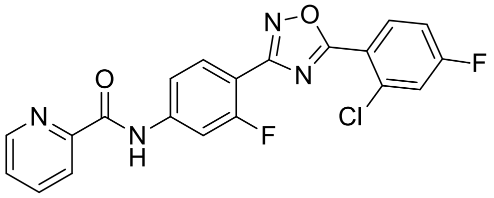

Prepared from **26d** and 2-chloro-4-fluorobenzoyl chloride according to general procedure 2. Crude product was purified by column chromatography (SiO_2, $CHCl_3$/MeOH 99:1) followed by maceration (2-PrOH/hexane 1:2); white solid, 0.18 g, 79.6%.

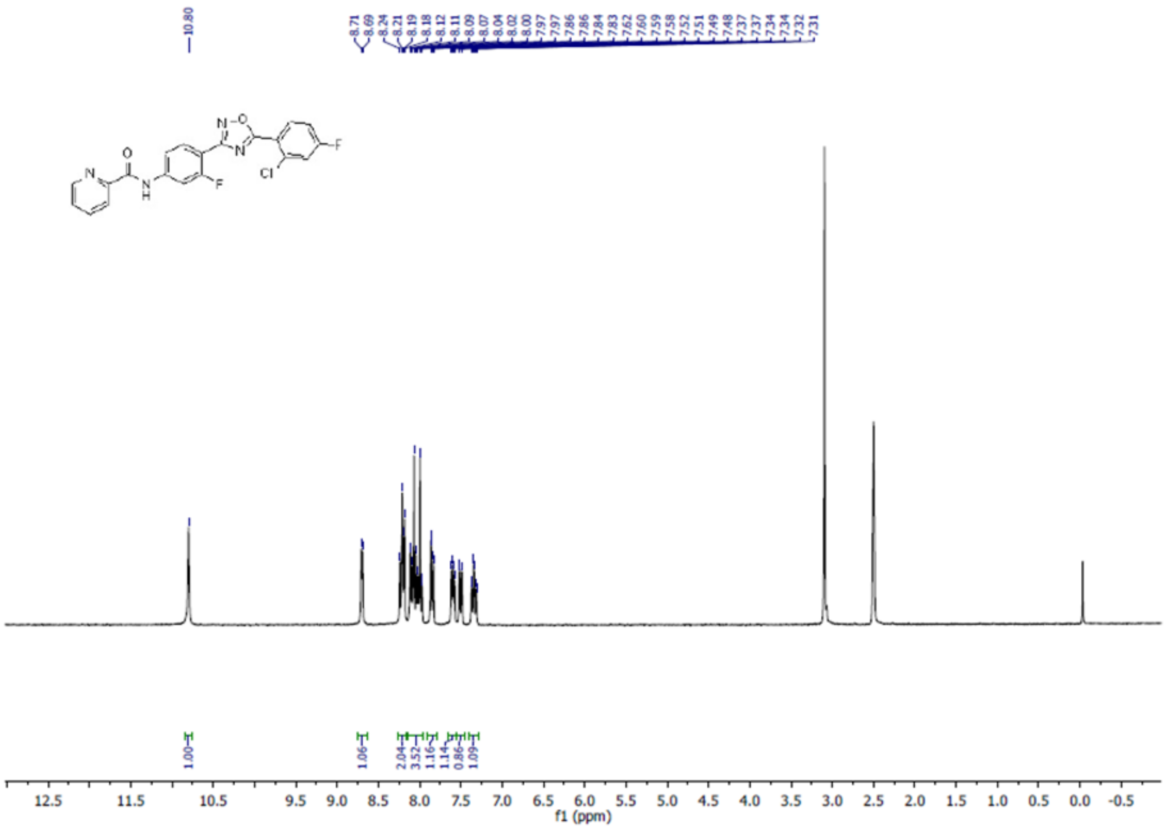

^{1}H NMR (300 MHz, $CDCl_3$ + DMSO-d_6), δ ppm: 10.80 (s br. 1H, NH), 8.70 (d br., J = 4.5 Hz, 1H), 8.24-8.18 (m, 2H), 8.12-7.97 (m, 3H), 7.85 (dd, J = 8.5 and 1.5 Hz, 1H), 7.60 (dd, J = 6.8 and 5.0 Hz, 1H), 7.50 (dd, J = 8.6 and 2.4 Hz, 1H), 7.34 (td, J = 8.5 and 2.4 Hz, 1H);

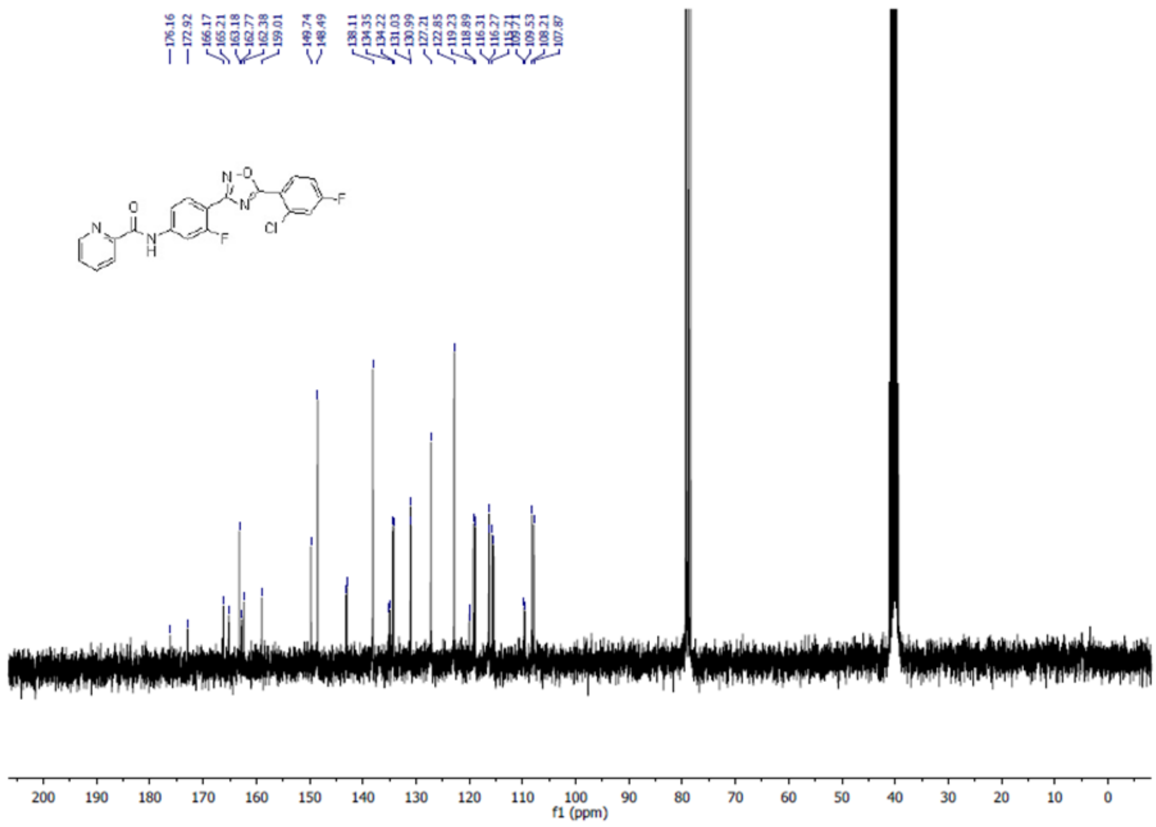

^{13}C NMR (75 MHz, $CDCl_3$ + DMSO-d_6), δ ppm: 176.2, 172.9, 165.2 (d, *J* = 5.9 Hz), 164.5 (d, *J* = 256.6 Hz), 163.2, 160.7 (d, *J* = 254.6 Hz), 149.7, 148.5, 143.1 (d, *J* = 11.4 Hz), 138.1, 135.0 (d, *J* = 10.8 Hz), 131.0 (d, *J* = 3.6 Hz), 127.2, 122.9, 120.0 (d, *J* = 3.7 Hz), 119.1 (d, *J* = 25.4 Hz), 116.3 (d, *J* = 3.1 Hz), 115.5 (d, *J* = 21.9 Hz), 109.6 (d, *J* = 13.3 Hz), 108.0 (d, *J* = 26.2 Hz); LC-MS (method B) R_T = 5.87 min (purity: 97.42%), *m/z* found: 413.06, calc.: 413.054 [M+H$^+$], HRMS: 413.066, mp=217-219°C.

N-(3-Fluoro-4-(5-(2-methoxyphenyl)-1,2,4-oxadiazol-3-yl)phenyl)picolinamide (58)

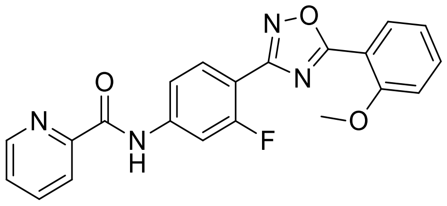

Prepared from **26d** and 2-methoxybenzoyl chloride according to general procedure 2. Crude product was purified by column chromatography (SiO_2, $CHCl_3$/MeOH 99:1) followed by maceration (2-PrOH/hexane 1:2); white solid, 0.16 g, 74.8%.

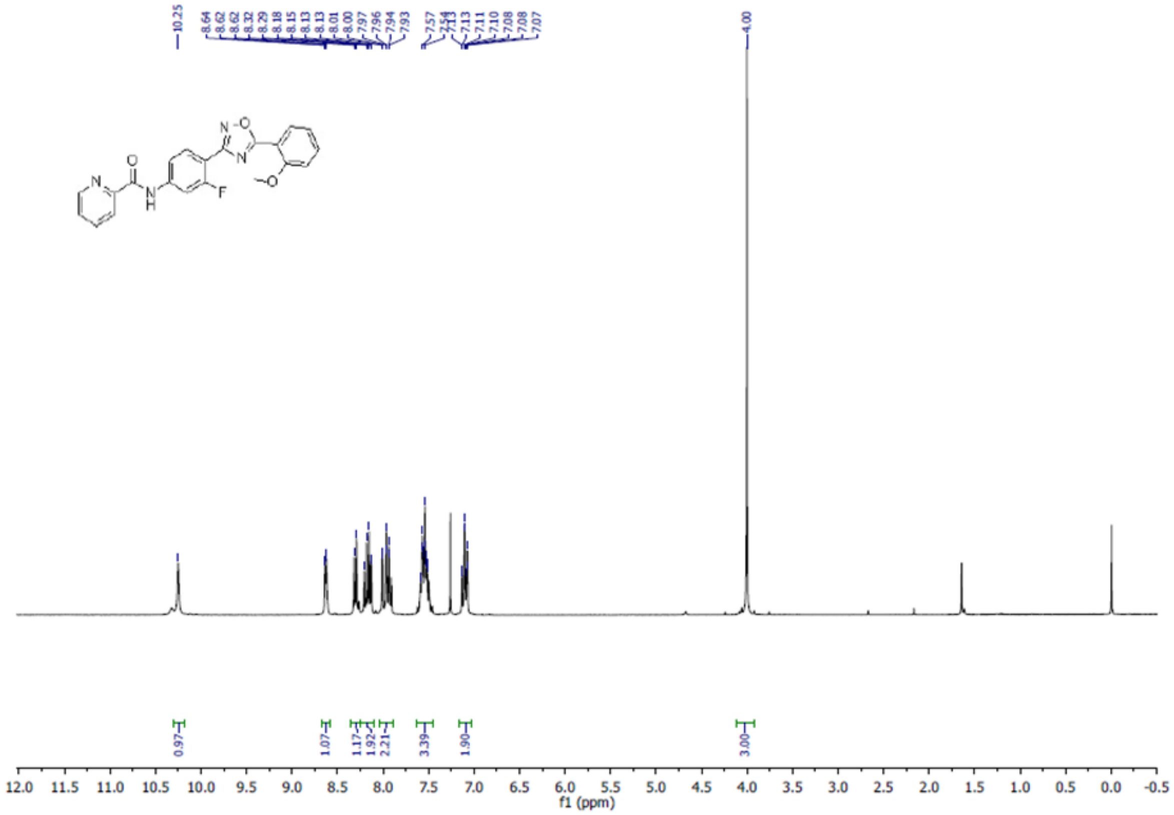

^{1}H NMR (300 MHz, $CDCl_3$), δ ppm: 10.25 (s br. 1H, NH), 8.63 (ddd, J = 4.8, 1.5 and 0.8 Hz, 1H), 8.30 (d br., J = 7.8, 1H), 8.21-8.14 (m, 2H), 8.01-7.91 (m, 2H), 7.62-7.46 (m, 3H), 7.13-7.07 (m, 2H), 4.00 (s, 3H, CH_3);

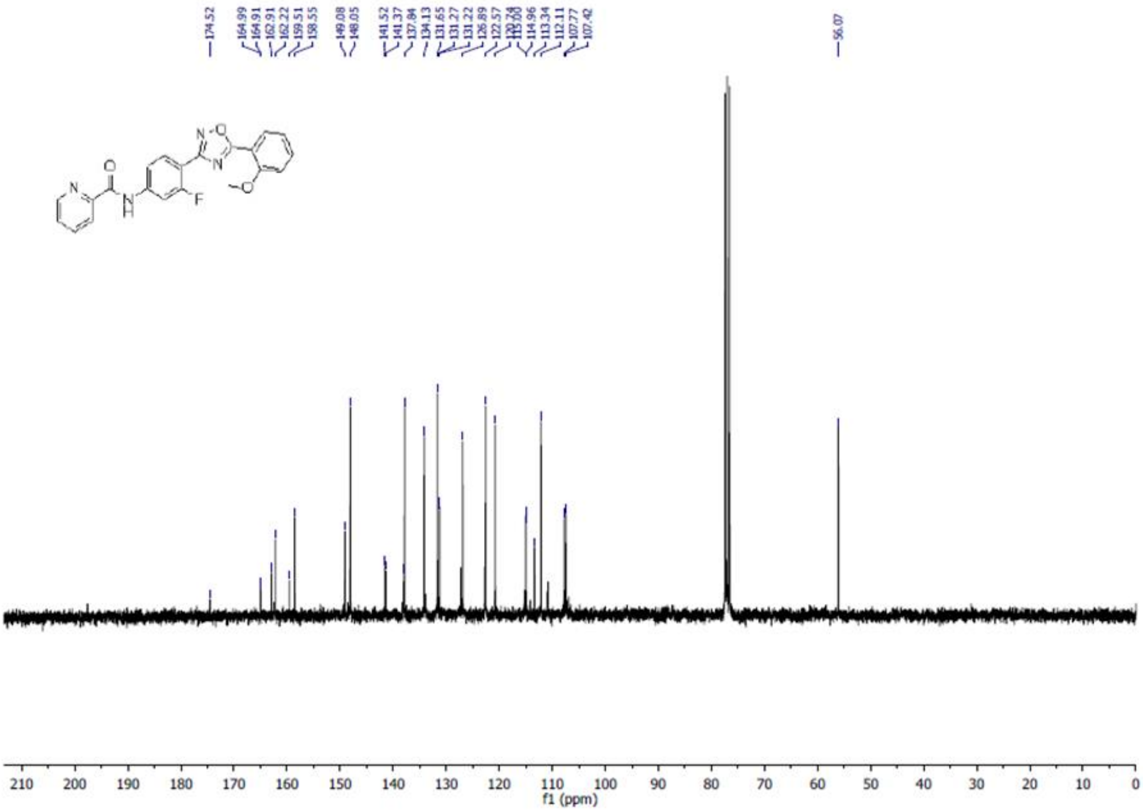

^{13}C NMR (75 MHz, $CDCl_3$), δ ppm: 174.5, 164.9 (d, *J* = 5.9 Hz), 162.2, 161.2 (d, *J* = 256.4 Hz), 158.6, 149.1, 148.1, 141.4 (d, *J* = 11.2 Hz), 137.9, 134.1, 131.7, 131.2 (d, *J* = 3.9 Hz), 126.9, 122.6, 120.8, 115.0 (d, *J* = 3.2 Hz), 113.3, 112.1, 110.9 (d, *J* = 12.7 Hz), 107.6 (d, *J* = 26.5 Hz), 56.1; LC-MS (method B) R_T = 5.27 min (purity: 90.0%), *m/z* found: 391.78, calc.: 391.113 [M+H^+], HRMS: 391.125, mp=214-216°C.

N-(4-(5-Phenyl-1,2,4-oxadiazol-3-yl)-3-(trifluoromethyl)phenyl)picolinamide (59)

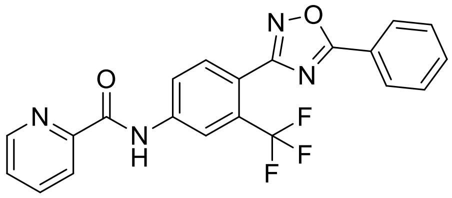

Prepared from **16l** and 2-pyridinecarbonyl chloride, hydrochloride according to general procedure 4. Crude product was purified by column chromatography (SiO_2, $CHCl_3$) followed by maceration (2-PrOH/hexane 1:2); white solid, 0.417 g, 86.2%.

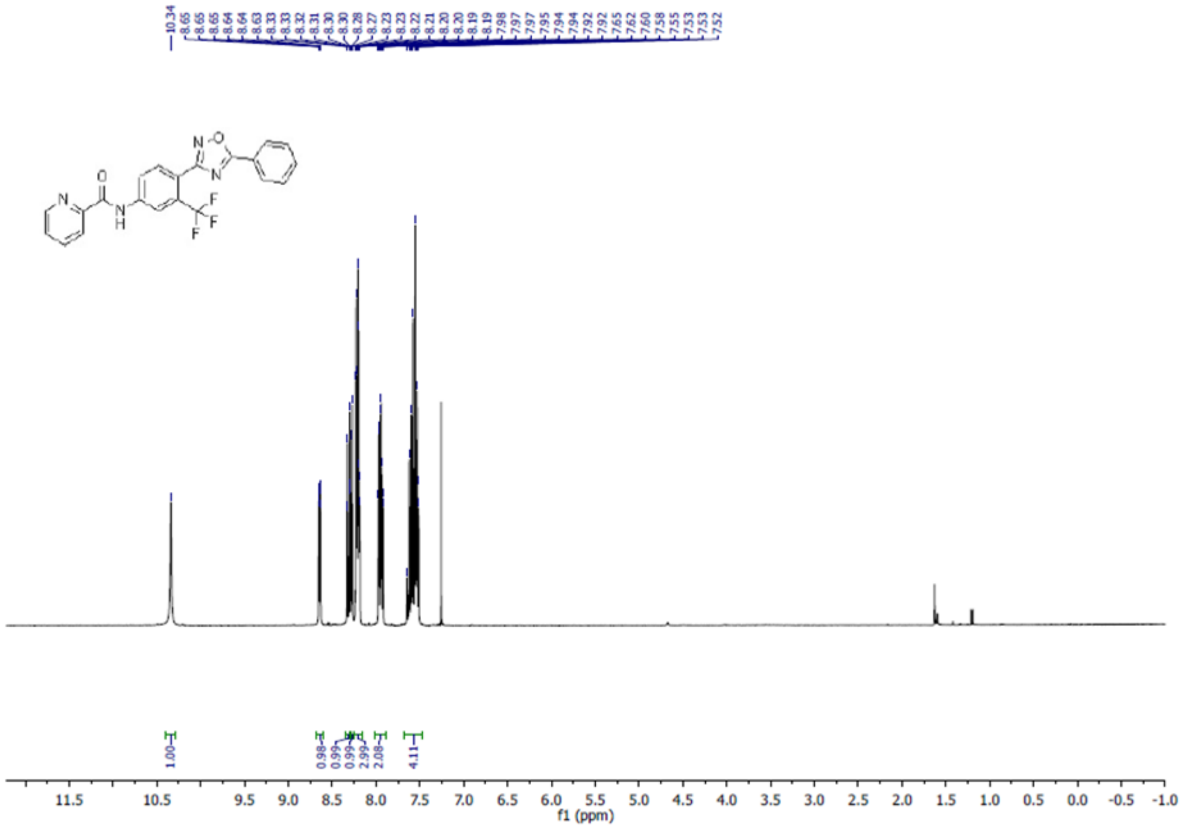

^{1}H NMR (300 MHz, $CDCl_3$), δ ppm: 10.34 (s br. 1H, NH), 8.65 (ddd, J = 4.8, 1.6 and 0.9 Hz, 1H), 8.31 (dt, J =7.9 and 1.1 Hz, 1H), 8.28 (d, J = 7.8 Hz, 1H), 8.24-8.19 (m, 3H), 7.98-7.92 (m, 2H), 7.65-7.51 (m, 4H);

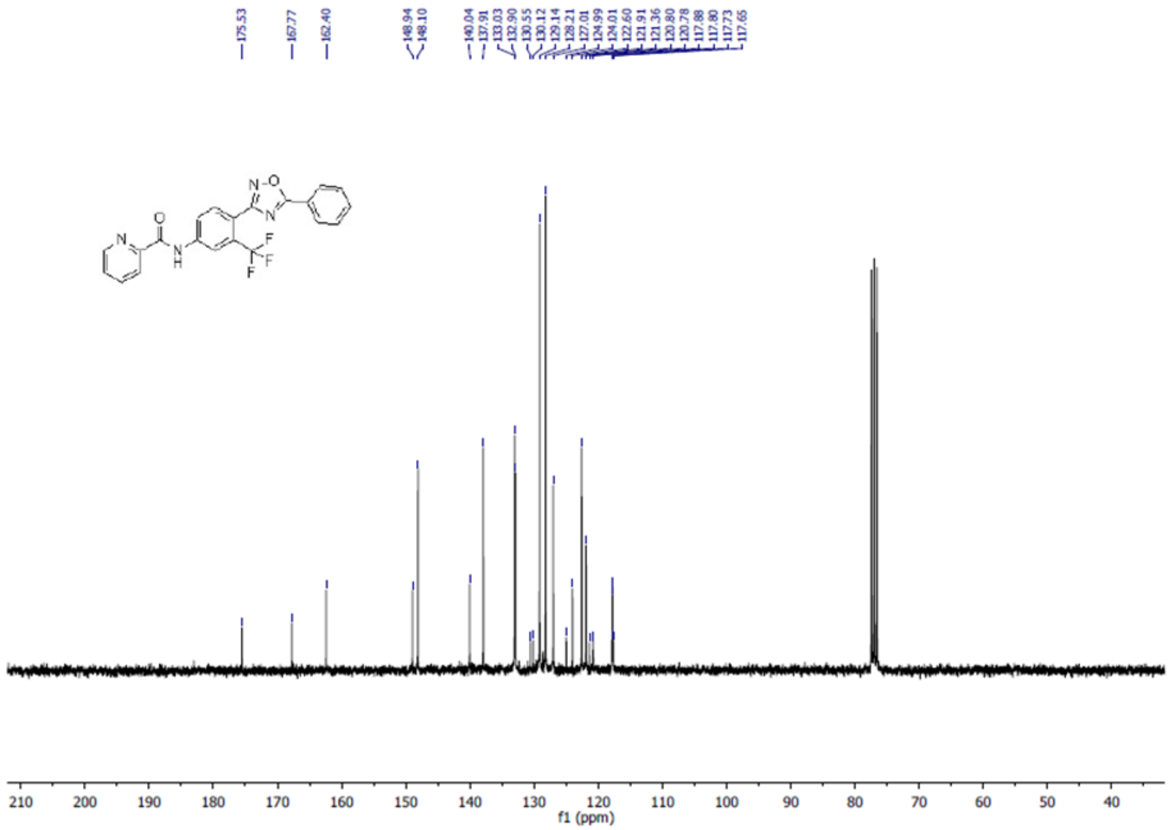

^{13}C NMR (75 MHz, $CDCl_3$), δ ppm: 175.5, 167.8, 162.4, 148.9, 148.1, 140.0, 137.9, 133.0, 132.9, 130.4 (q, J = 32.4 Hz), 129.1 (2C), 128.2 (2C), 127.1, 124.0, 123.2 (d, J = 274.0 Hz), 122.6, 121.9, 120.8 (q, J = 1.7 Hz), 117.7 (q, J = 5.7 Hz); LC-MS (method B) R_T = 3.80 min (purity: 100%), *m/z* found: 411.6, calc.: 411.099 [M+H^+], HRMS: 411.115, mp=141-142°C

***N*-(4-(5-(2-Chlorophenyl)-1,2,4-oxadiazol-3-yl)-3-(trifluoromethyl)phenyl)picolinamide (60)**

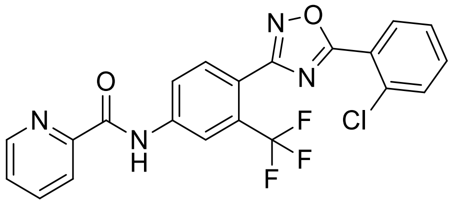

Prepared from **16m** and 2-pyridinecarbonyl chloride, hydrochloride according to general procedure 4. Crude product was purified by column chromatography (SiO_2, $CHCl_3$) followed by maceration (2-PrOH/hexane 1:2); white solid, 0.15 g, 95.5%.

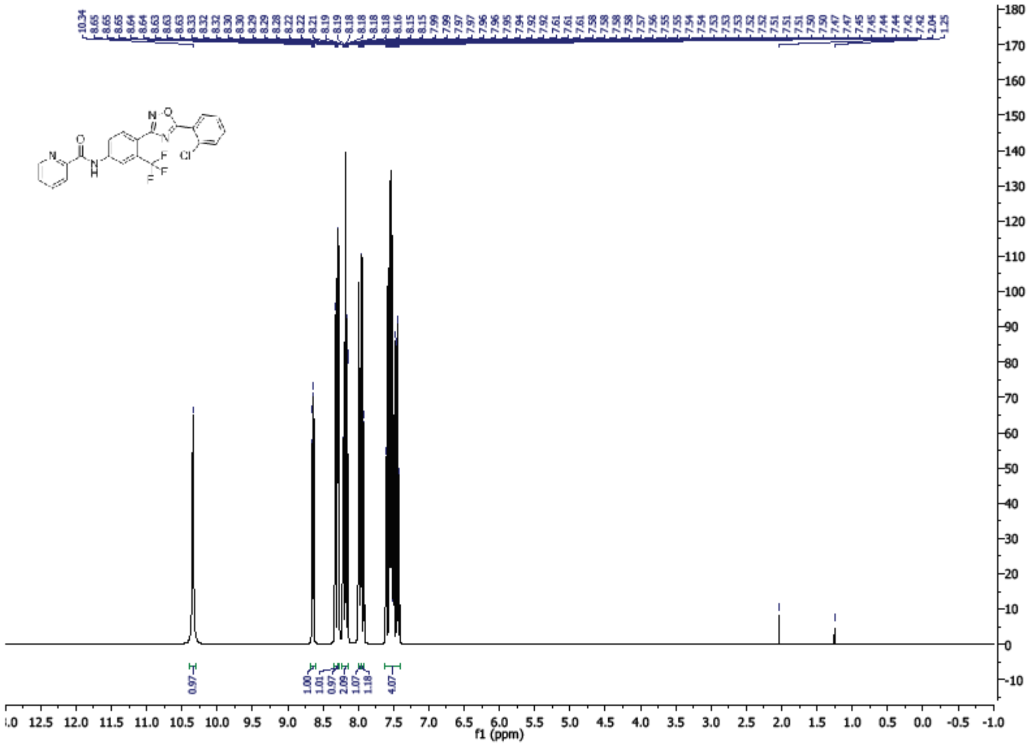

^{1}H NMR (300 MHz, $CDCl_3$), δ ppm: 10.35 (s br. 1H, NH), 8.65 (ddd, J = 4.8, 1.6 and 0.9 Hz, 1H), 8.30 (dt, J = 7.8 and 1.1 Hz, 1H), 8.29 (d br., J =2.2 Hz, 1H), 8.22-8.15 (m, 2H), 7.98-7.92 (m, 2H), 7.61-7.42 (m, 4H);

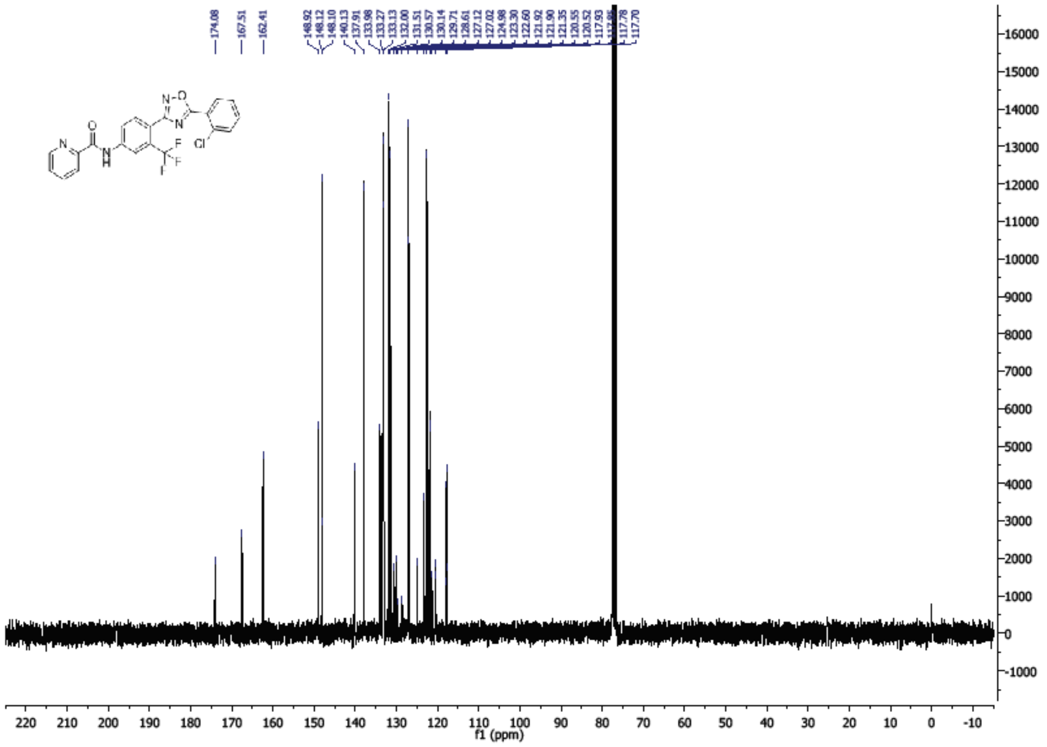

^{13}C NMR (75 MHz, $CDCl_3$) δ 174.1, 167.5, 162.4, 148.9, 148.1, 140.1, 137.9, 134.0, 133.3, 133.1, 132.0, 131.5, 130.4 (q, J = 32.5 Hz), 127.1, 127.0, 123.3, 123.2 (d, J = 123.2 Hz), 122.6, 121.9, 120.5 (q, J = 1.9 Hz), 117.7 (q, J = 5.7 Hz); LC-MS (method A) R_T = 3.85 min (purity: 100%), *m/z* found: 445.0, calc.: 445.060 [M+H$^+$], HRMS: 445.061, mp=140-141°C

N-(3-Methyl-4-(5-phenyl-1,2,4-oxadiazol-3-yl)phenyl)picolinamide (61)

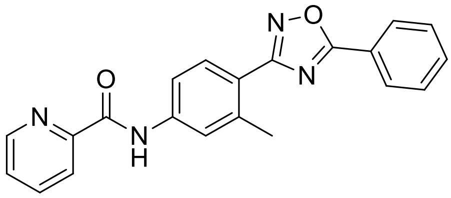

Prepared from **16n** and 2-pyridinecarbonyl chloride, hydrochloride according to general procedure 4. Crude product was purified by column chromatography (SiO_2, $CHCl_3$/MeOH 99:1) followed by maceration (2-PrOH/hexane 1:2); white solid, 0.21 g, 75.0%.

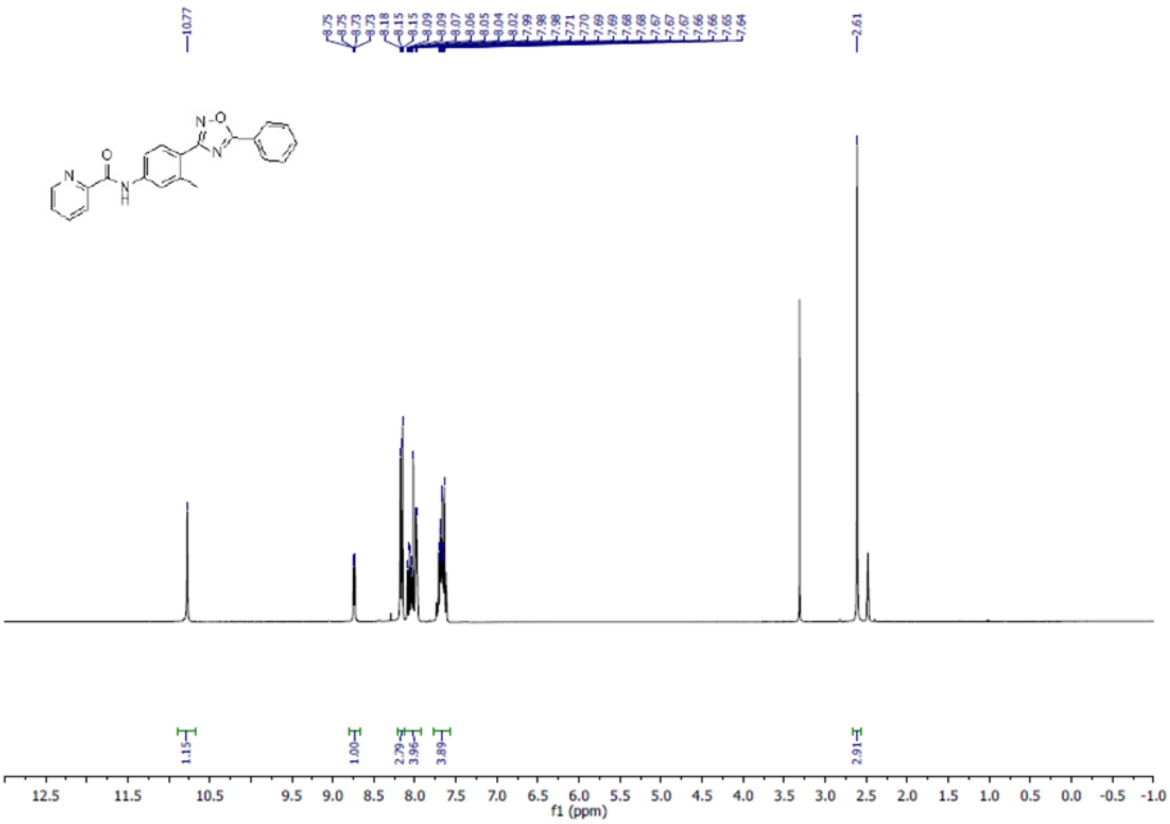

^{1}H NMR (300 MHz, DMSO-d_6), δ ppm: 10.77 (s br. 1H, NH), 8.75 (ddd, J = 4.8, 1.6 and 0.9 Hz, 1H), 8.18-8.15 (m, 3H), 8.09-7.97 (m, 4H), 7.74-7.61 (m, 4H), 2.61 (s, 3H, CH_3);

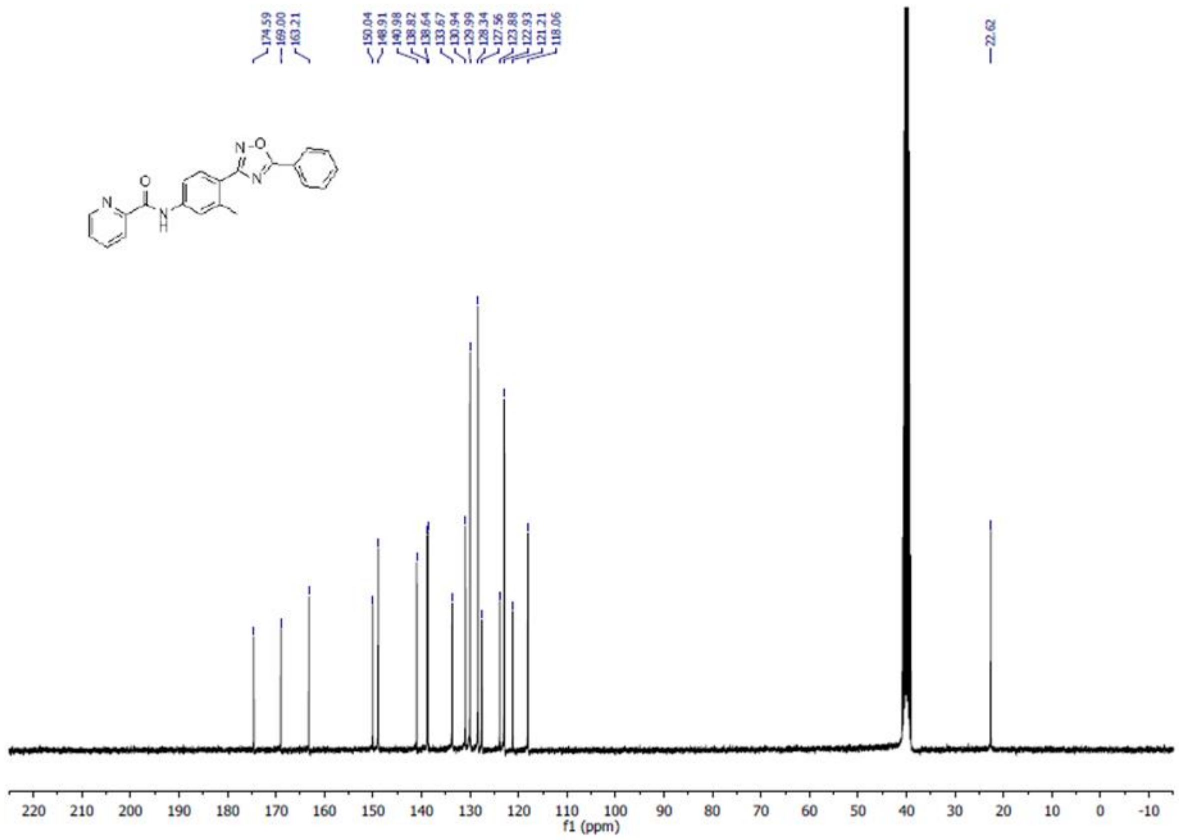

^{13}C NMR (75 MHz, DMSO-d_6), δ ppm: δ 174.6, 169.0, 163.2, 150.0, 148.9, 141.0, 138.8, 138.6, 133.7, 130.9, 130.0 (2C), 128.3 (2C), 127.6, 123.9, 122.9 (2C), 121.2, 118.1, 22.6; LC-MS (method B) R_T = 3.89 min (purity: 98.5%), *m/z* found: 357.0, calc.: 357.127 [$M+H^+$], HRMS: 357.134, mp=130-131°C.

N-(4-(5-(2-Chlorophenyl)-1,2,4-oxadiazol-3-yl)-3-methylphenyl)picolinamide (62)

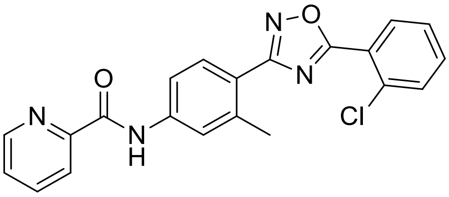

Prepared from **16o** and 2-pyridinecarbonyl chloride, hydrochloride according to general procedure 4. Crude product was purified by column chromatography (SiO_2, $CHCl_3$/MeOH 99:1) followed by maceration (2-PrOH/hexane 1:2); white solid, 0.41 g, 93.6%.

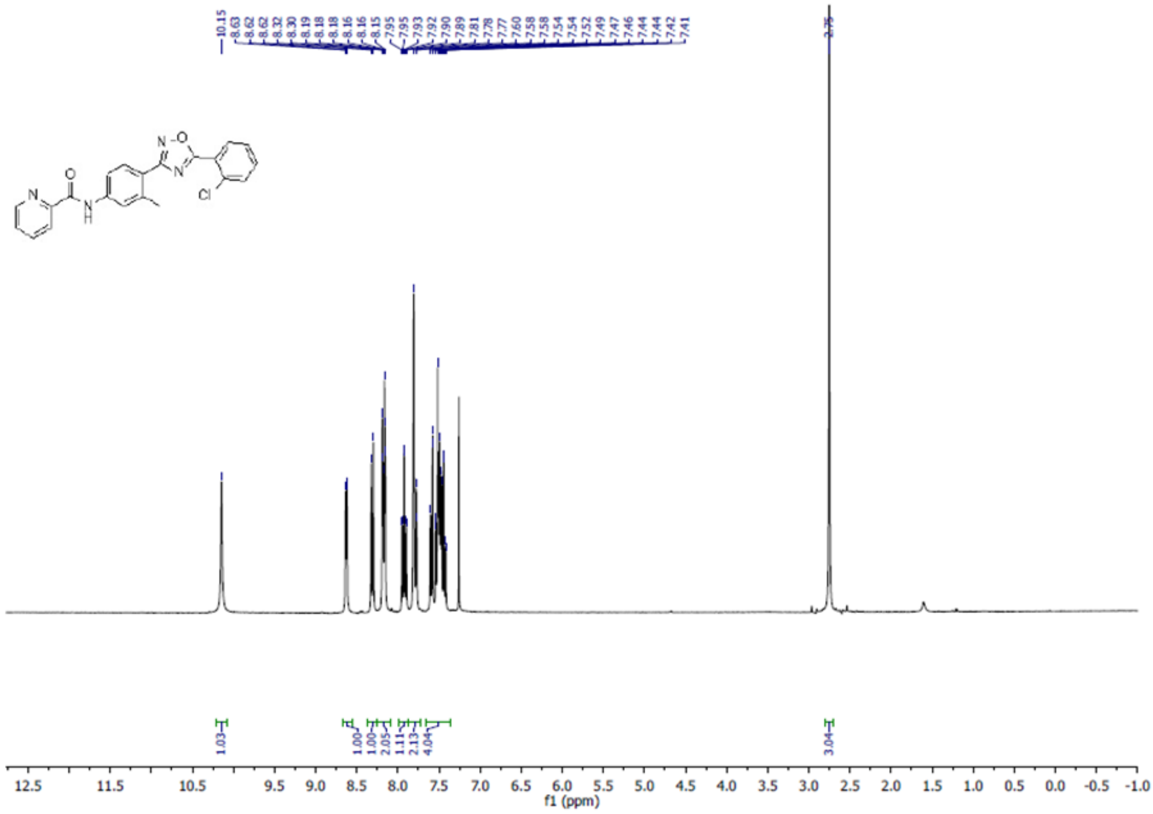

^{1}H NMR (300 MHz, $CDCl_3$), δ ppm: 10.15 (s br. 1H, NH), 8.63 (ddd, J = 4.8, 1.6 and 0.9 Hz, 1H), 8.31 (d br., J = 7.8 Hz, 1H), 8.20-8.14 (m, 2H), 7.93 (td, J = 7.7 and 1.7 Hz, 1H), 7.81-7.77 (m, 2H), 7.61-7.41 (m, 4H), 2.75 (s, 3H, CH_3);

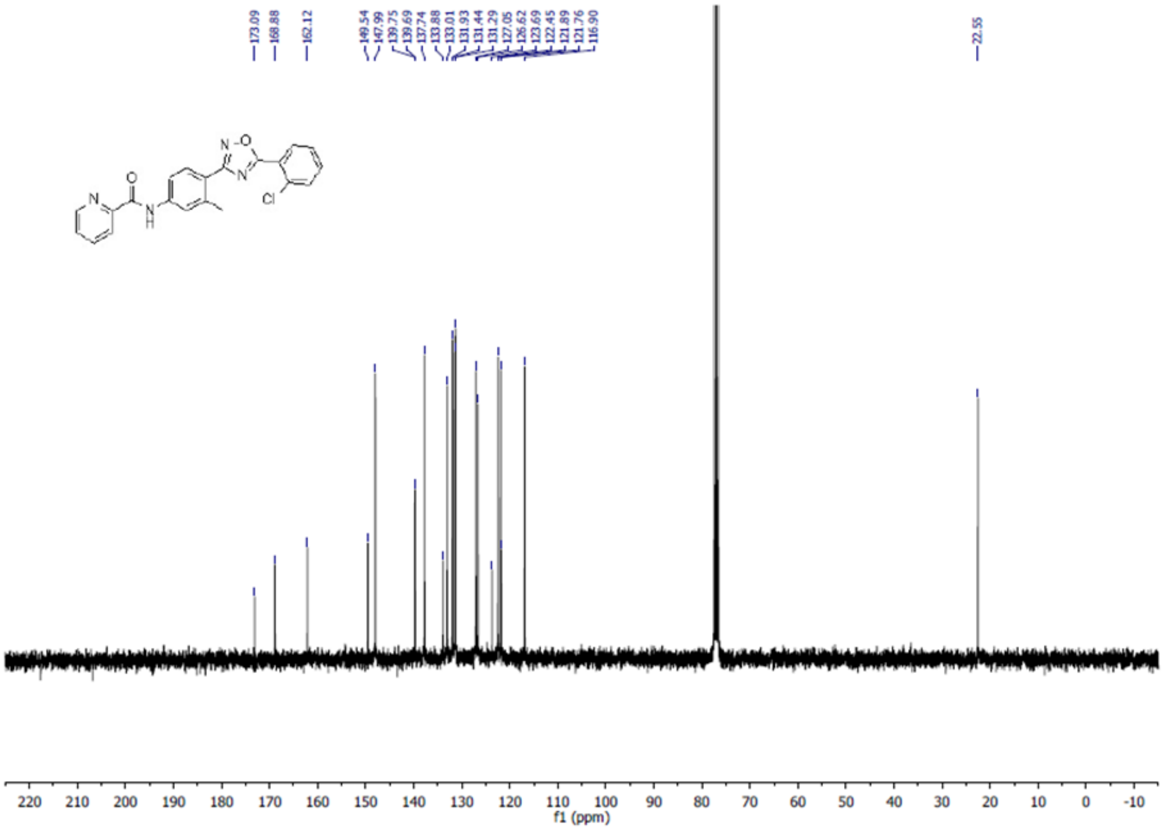

^{13}C NMR (75 MHz, $CDCl_3$), δ ppm: 173.1 168.9, 162.1, 149.5, 148.0, 139.8, 139.7, 137.8, 133.9, 133.0, 131.9, 131.4, 131.3, 127.1, 126.6, 123.7, 122.5, 121.9, 121.8, 116.9, 22.6; LC-MS (method A) R_T = 3.94 min (purity 96.7%), *m/z* found: 391.4, calc.: 391.088 [$M+H^+$], HRMS: 391.099, mp=147-149°C.

.

2. In vitro mGlu receptor selectivity profile

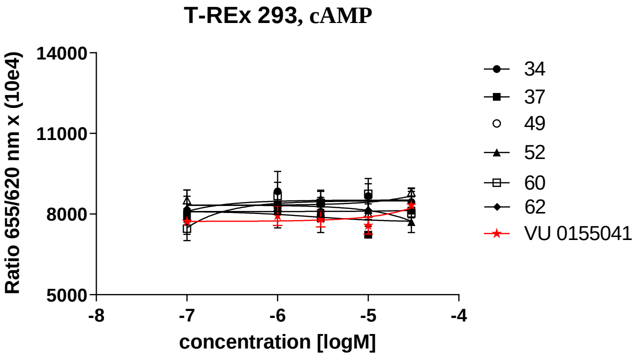

Figure S1. Activity of compounds **34**, **37**, **49**, **52**, **60** and **62** in forskolin-induced cAMP accumulation in untransfected cells of the T-REx 293 line. Similar to reference $mGlu_4$ receptor PAM VU155041, no effects of the tested 1,2,4-oxadiazole derivatives were observed in the cAMP assay in the mock T-REx 293 cell line.

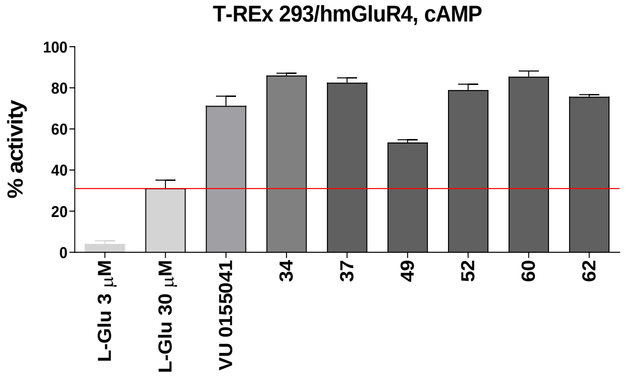

Figure S2. Activity of compounds **34**, **37**, **49**, **52**, **60**, **62** and VU155041 (ago-PAM of $mGlu_4$ receptor used as reference drug) tested at a concentration of 10 μM in NAM mode (in the presence of EC_{80} concentration of L-Glu (30 μM)) by the cAMP accumulation assay in cells expressing $mGlu_4$ receptor.

A

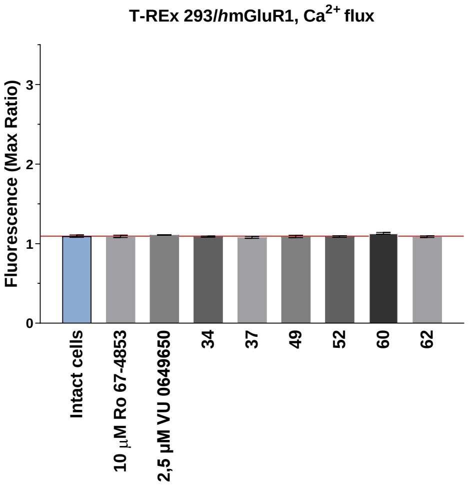
T-REx 293/hmGluR1, Ca2+ flux
Fluorescence (Max Ratio)
0
1
2
3
Intact cells
10 μM Ro 67-4853
2,5 μM VU 0649650
34
37
49
52
60
62

D

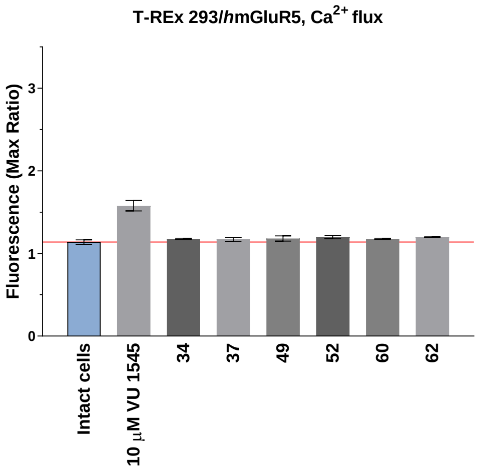
T-REx 293/hmGluR5, Ca2+ flux
Fluorescence (Max Ratio)
0
1
2
3
Intact cells
10 μM VU 1545
34
37
49
52
60
62

B

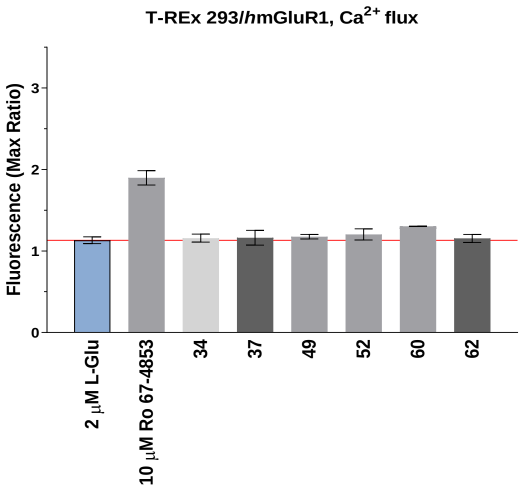
T-REx 293/hmGluR1, Ca2+ flux
Fluorescence (Max Ratio)
0
1
2
3
2 μM L-Glu
10 μM Ro 67-4853
34
37
49
52
60
62

E

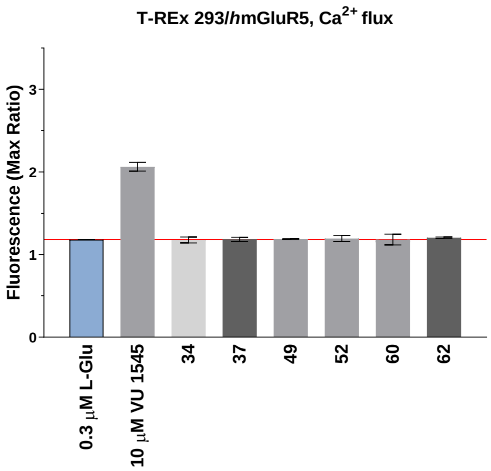
T-REx 293/hmGluR5, Ca2+ flux
Fluorescence (Max Ratio)
0
1
2
3
0.3 μM L-Glu
10 μM VU 1545
34
37
49
52
60
62

C

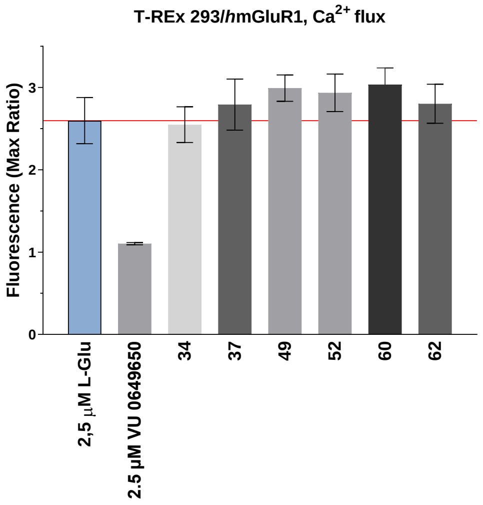

F

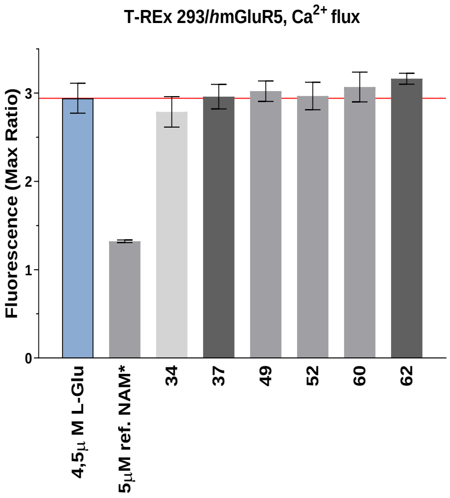

Figure S3. Activity of compounds **34**, **37**, **49**, **52**, **60**, **62** and reference drugs (VU 0469650, the NAM of $mGlu_1$ receptor; Ro 67-4853, the PAM of $mGlu_1$ receptor; VU 1545, the PAM of $mGlu_5$ receptor; and the 3-(2-pyridyl)-5-(2-chlorophenyl)-1,2,4-oxadiazole, the NAM of $mGlu_5$ receptor (PCT/US00/22618)) tested at a concentration of 10 μM by the Ca^{2+} flux method (by HAMAMATSU FDSS/μcell) in cells expressing $mGlu_1$ receptor and $mGlu_5$ receptor.

A/D – Agonistic activity of the compounds in cells expressing $mGlu_1$ receptor and $mGlu_5$ receptor.

B/E – PAM activity of the compounds in the presence of L-Glu at the EC_{20} concentration (2 μM for $mGlu_1$ receptor and 0.3 μM for $mGlu_5$ receptor).

C/F – NAM activity of the compounds in the presence of L-Glu at the EC_{80} concentration (2.5 μM for $mGlu_1$ receptor and 4.5 μM for $mGlu_5$ receptor).

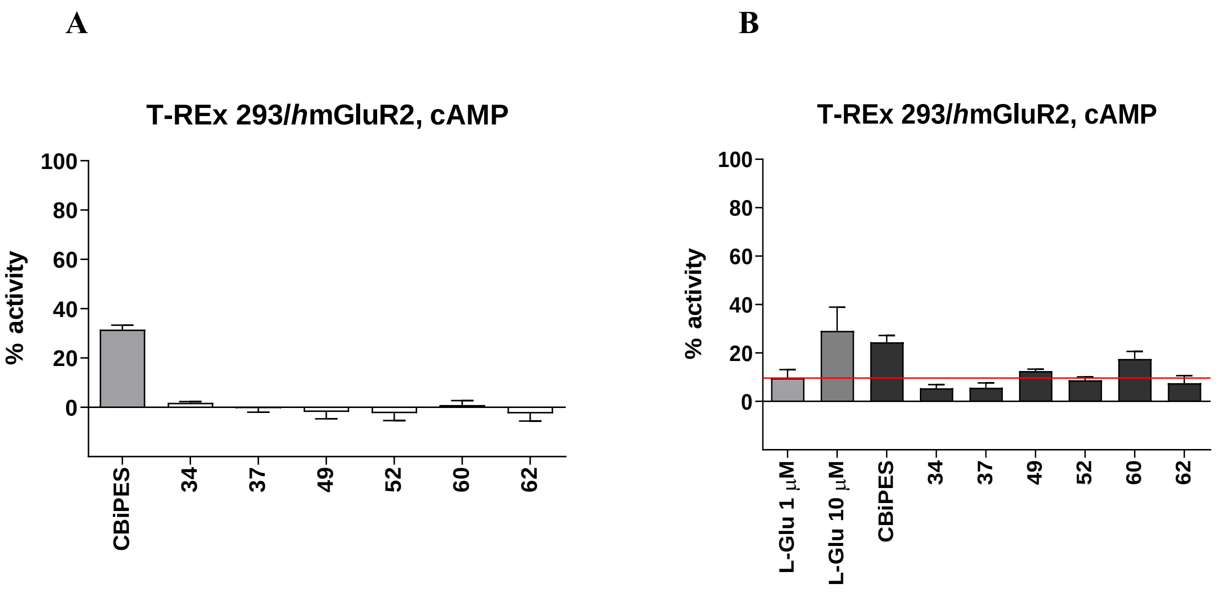

Figure S4. Activity of compounds **34**, **37**, **49**, **52**, **60**, **62** and CBiPES (PAM of $mGlu_2$ receptor, used as reference drug) at a concentration of 10 μM verified by cAMP accumulation assay in cells expressing human $mGlu_2$ receptor. (**A**) Agonist activity of the compounds on the cAMP level.
(B) PAM activity of the compounds in the presence of the EC_{20} (1 μM) of L-glutamic acid

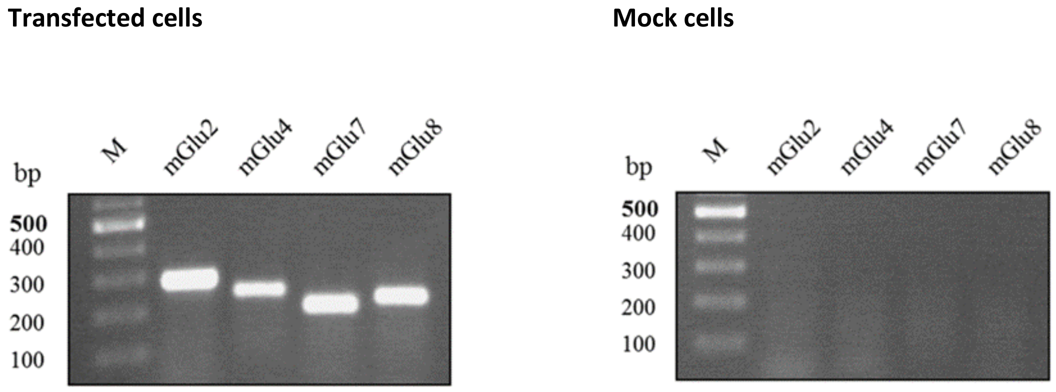

Figure S5. mRNA expression of mGluR 2, 4, 7 and 8 analysed by RT-PCR in transfected or not T-REx 293 cell line after 24h Tet induction. M – standard DNA marker (O'GeneRuler^{TM} DNA ladder Mix).

Table 1 RT-PCR primer sequences.

Gen	Starter Forward 5'->3'	Starter Reverse 5'->3'
GRM2	CCTACAATGTGCTCCTCATC	GCTAACCACGTTCTTCTGC
GRM4	CGACAAGCTGTACATCCAG	GTTCTCGCAGAGCTCAGAC
GRM7	GTGTATGCCATCAAGACTCG	CCACTGATGCACTTAGGTTC
GRM8	GTGCTTCAGTATCTCTGG	GTCTTGGTAGAGGAAGTG

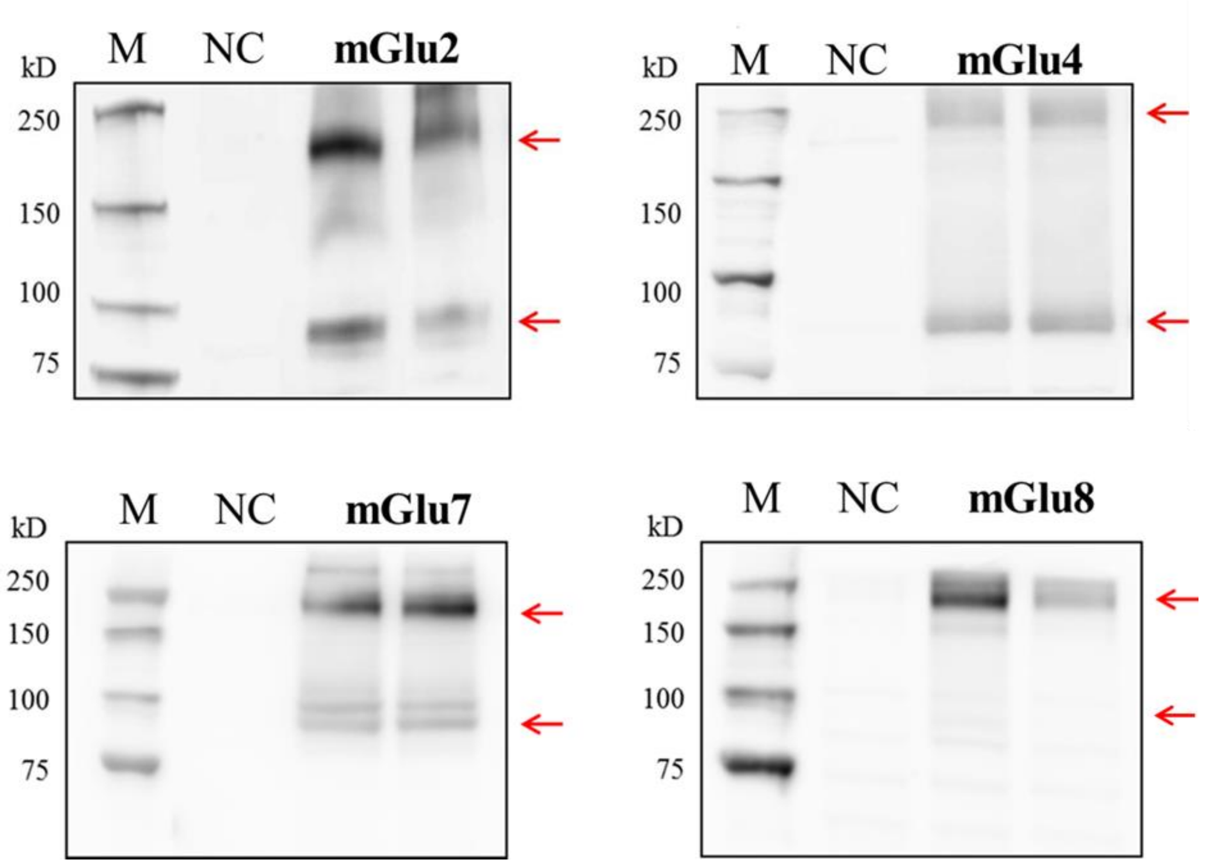

Figure S6. Analysis of the expression of mGlu$_2$, mGlu$_4$, mGlu$_7$, and mGlu$_8$ by the Western blot method, in the T-REx 293 cell lines. M - Mass standard (Precision Plus Protein WesternC Standard); NC – non transfeced T-REx cells 293; mGlu$_{2,4,7,8}$ – cells transfected as indicated after Tet treatment. Arrows indicated the receptor protein as monomer or dimmer. Primary Ig mGluR2: R&D Systems MAB4676; mGluR4: Abcam ab53088; mGluR7: Abcam ab53705; mGluR8: Abcam ab53094.

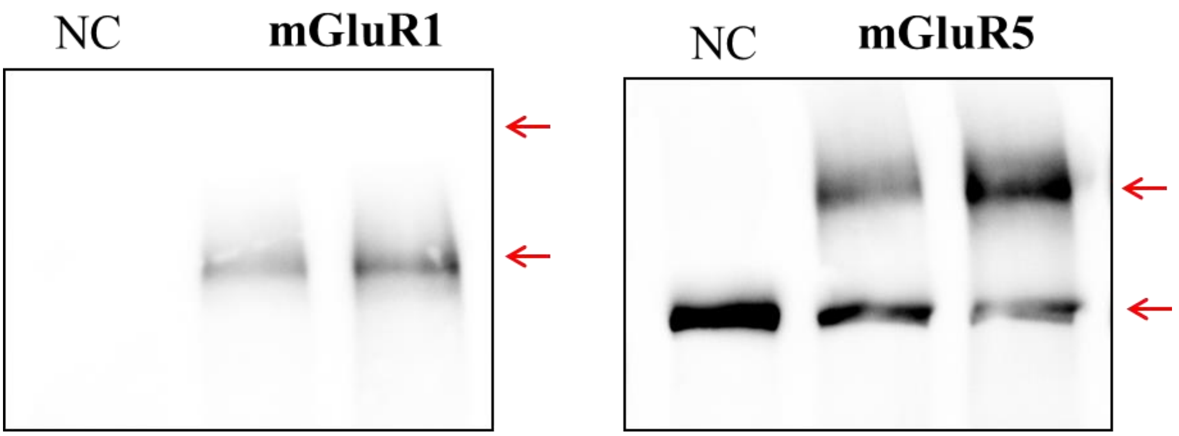

Figure S7. Analysis of the expression of $mGlu_1$ and $mGlu_5$ by the Western blot method, in the T-REx 293 cell lines. NC – non-transferred T-REx 293 cells, $mGlu_{1,5}$ – transfected cell after Tet treatment. Arrows indicated the receptor protein as monomer or dimmer. Primary Ig mGluR1: R&D Systems MAB4836; mGluR5: R&D Systems MAB4514.

3. Preliminary safety

3.1 In vitro binding to hERG assay

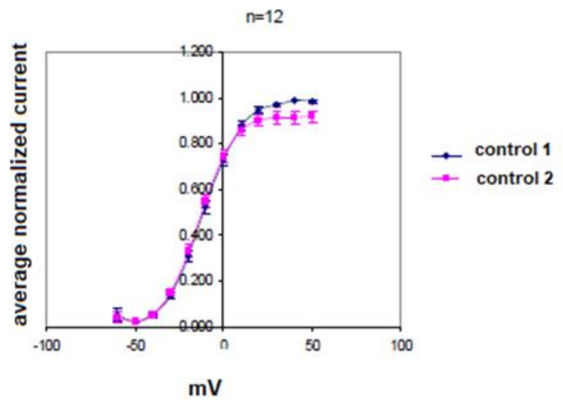

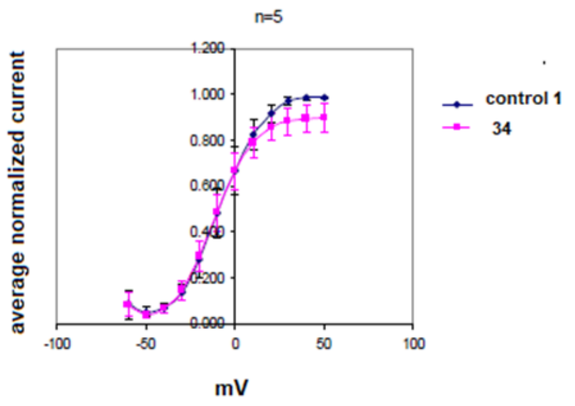

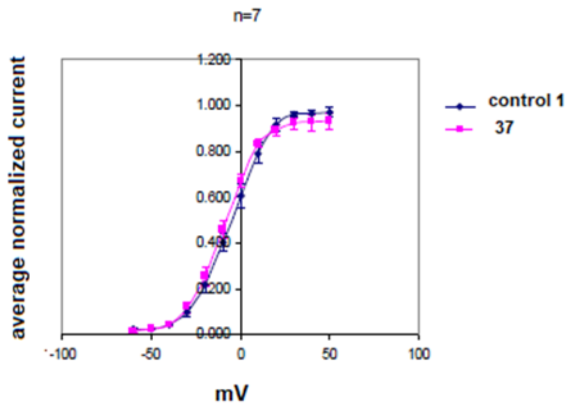

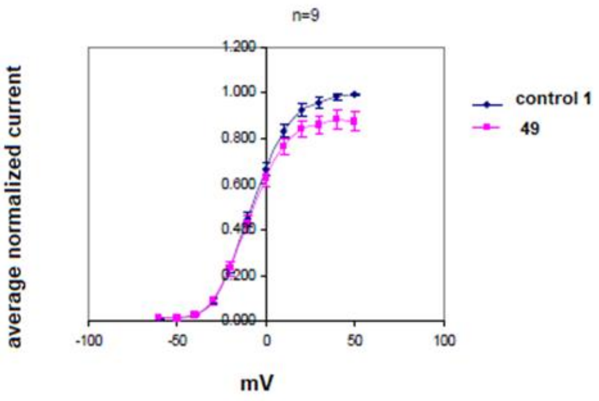

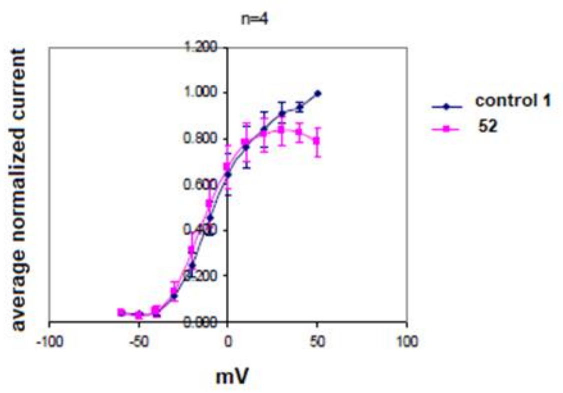

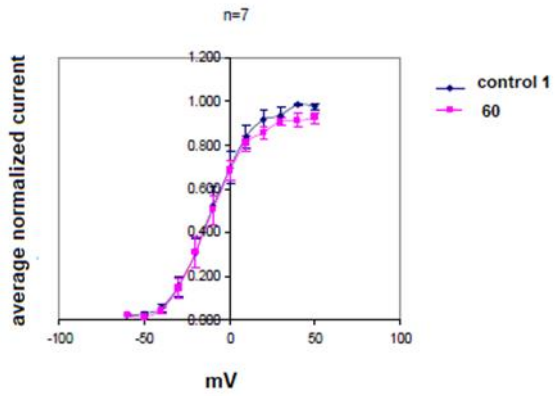

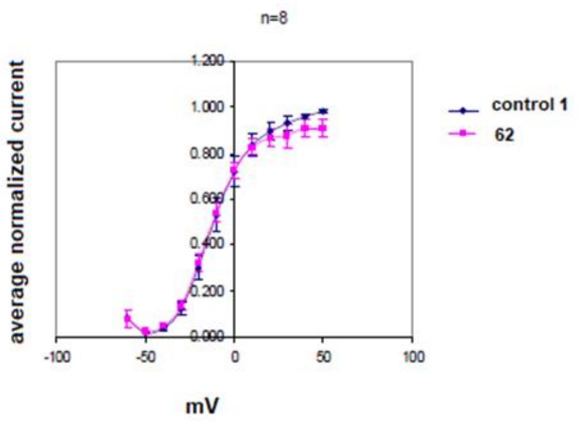

Figure S8 . Voltage-activation curves of compounds **34**, **37**, **49**, **52**, **60**, **62**, normalized peak tail current dentisity at –50 mV was plotted against tested potential, n-number of experiments.

3.2 Mutagenicity potential-mini-AMES

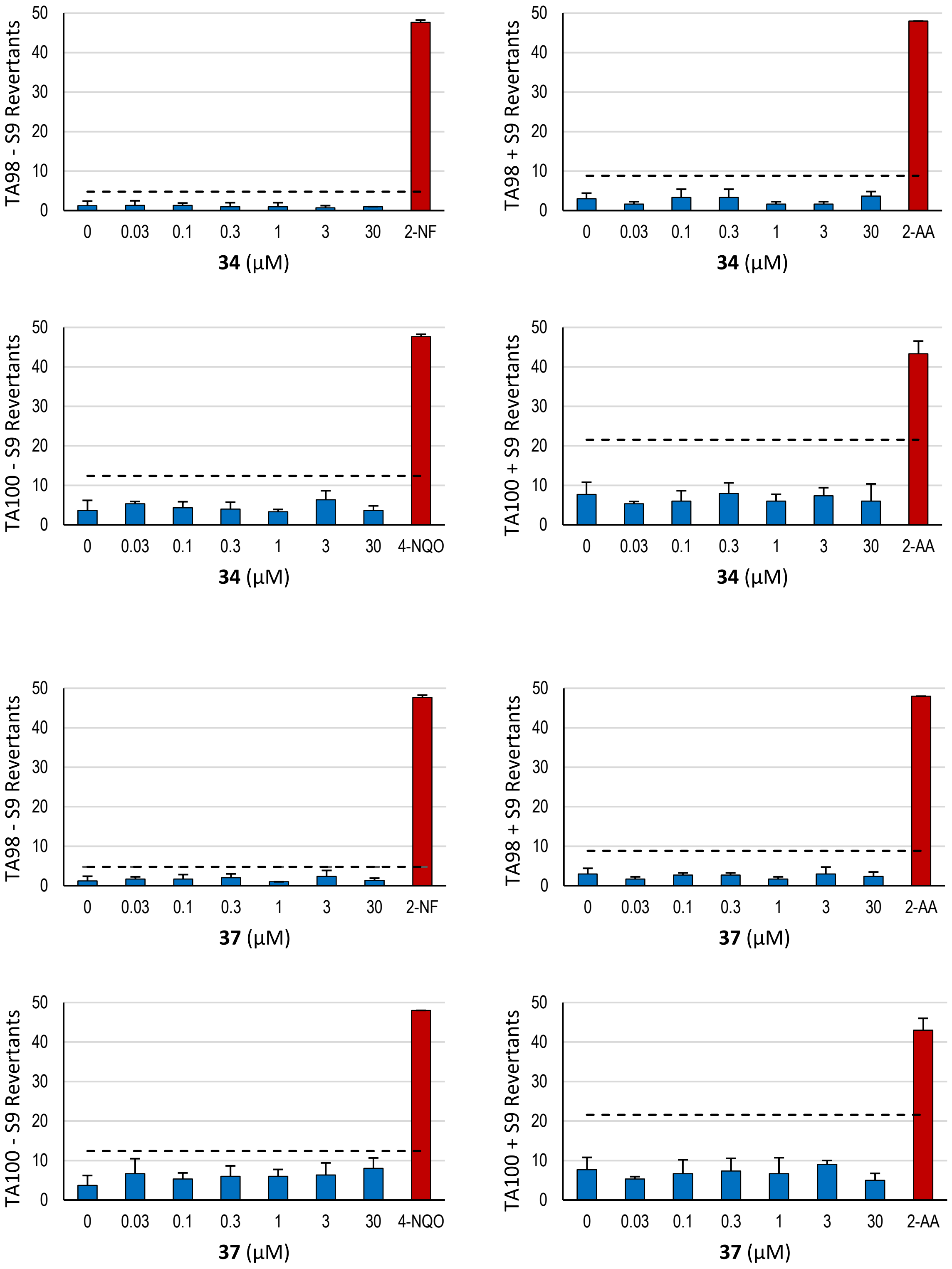

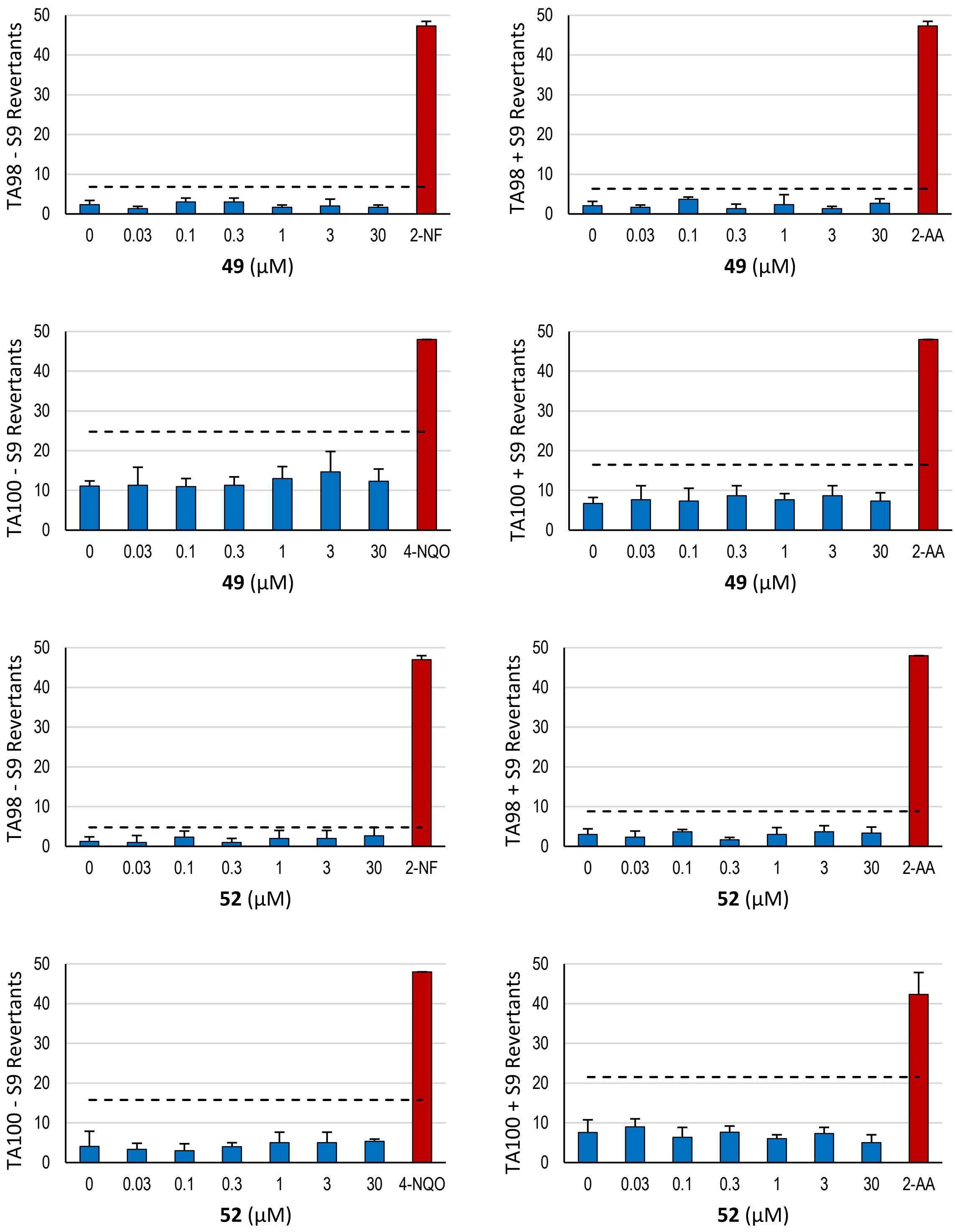
TA98 - S9 Revertants
0 0.03 0.1 0.3 1 3 30 2-NF
49 (µM)
TA98 + S9 Revertants
0 0.03 0.1 0.3 1 3 30 2-AA
49 (µM)
TA100 - S9 Revertants
0 0.03 0.1 0.3 1 3 30 4-NQO
49 (µM)
TA100 + S9 Revertants
0 0.03 0.1 0.3 1 3 30 2-AA
49 (µM)
TA98 - S9 Revertants
0 0.03 0.1 0.3 1 3 30 2-NF
52 (µM)
TA98 + S9 Revertants
0 0.03 0.1 0.3 1 3 30 2-AA
52 (µM)
TA100 - S9 Revertants
0 0.03 0.1 0.3 1 3 30 4-NQO
52 (µM)
TA100 + S9 Revertants
0 0.03 0.1 0.3 1 3 30 2-AA
52 (µM)

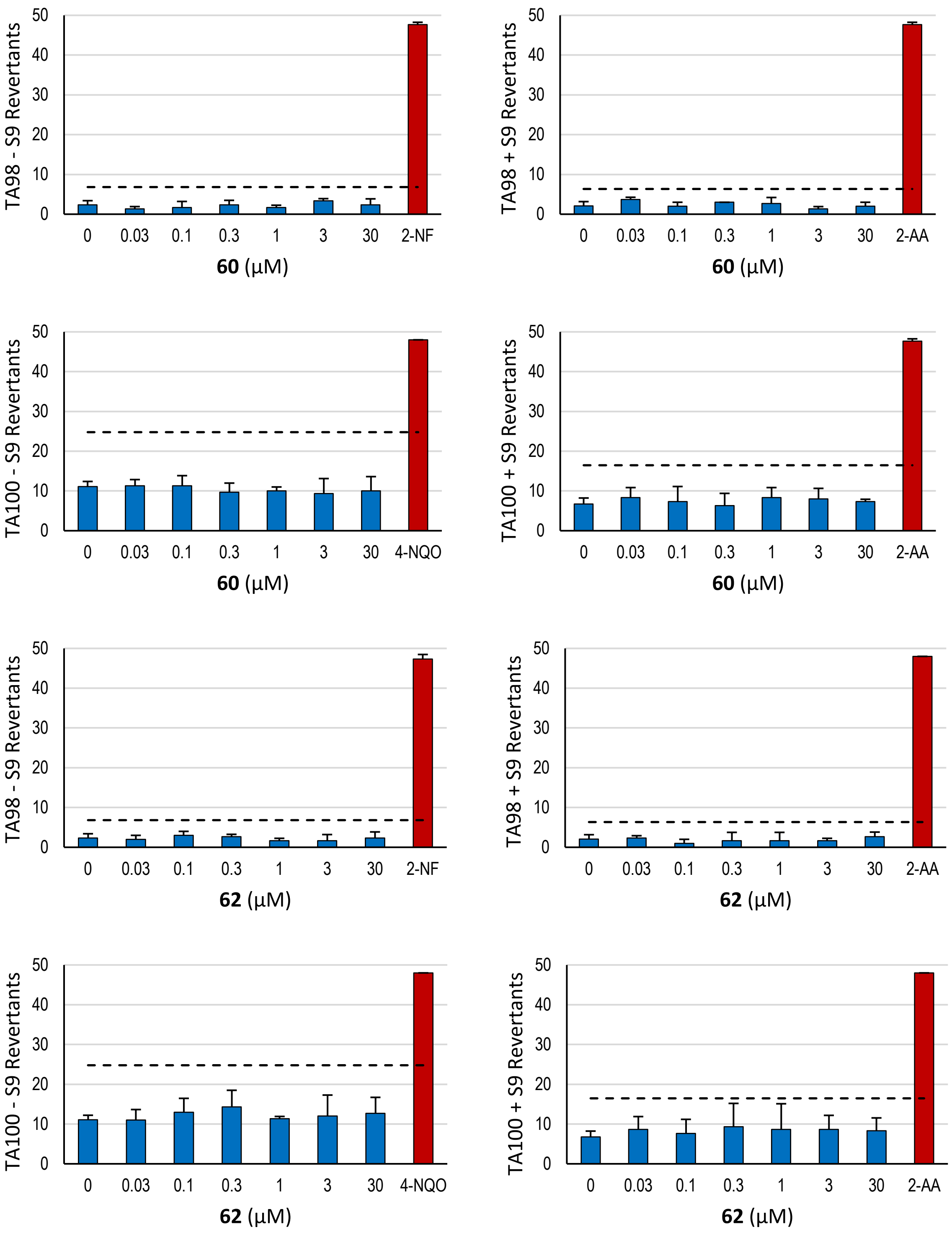

Figure S9. The AMES test revealed no mutagenic potential for **34**, **37**, **49**, **52**, **60** and **62**. The AMES test was conducted with two Salmonella typhimurium strains TA98 and TA100 in the presence or absence of liver homogenate S9 to simulate the metabolic conversions of tested compounds with liver enzymes. The AMES MPF 98/100 assay from Xenometrix was performed as described by the supplier. Data were analysed with the AMES MPF calculation

sheet provided by Xenometrix. The experiment was performed once in triplicates as suggested by the supplier. The dashed line indicates 2-fold increase over baseline. Positive control Abb.: 2-AA, 2-aminoanthracene; 2-NF, 2-nitrofuorene; 4-NQO: 4-nitroquinoline-N-oxide.

4. In vivo tests

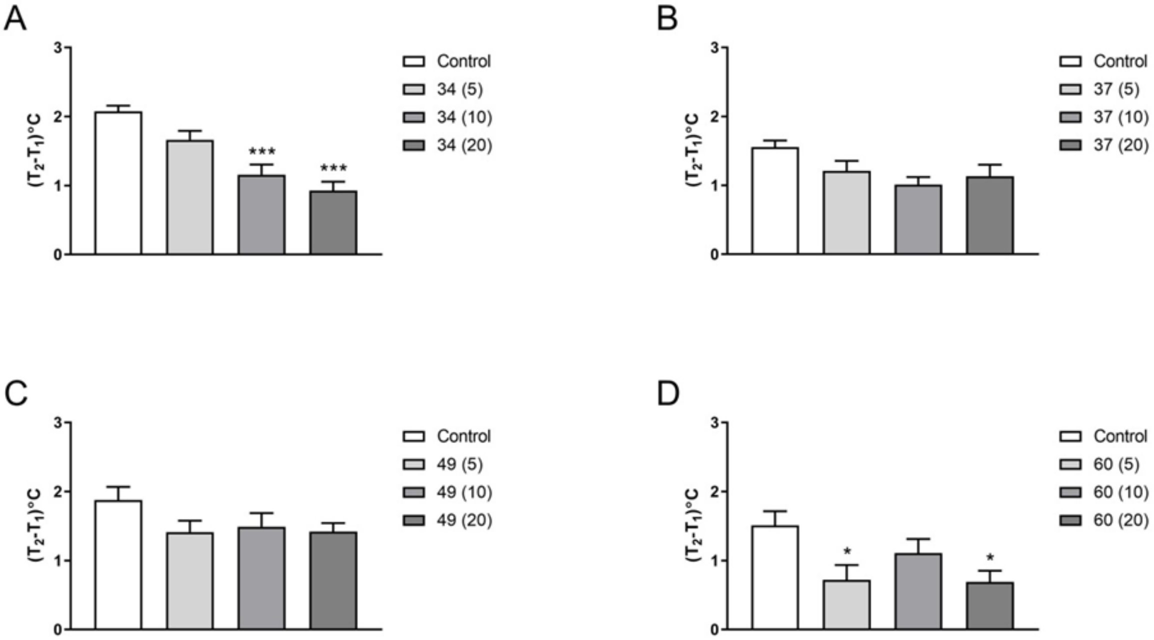

Figure S10. The effects of compounds **34**, **37**, **49**, and **60** on stress-induced hyperthermia (SIH) in mice. Compound **34** induced a dose-dependent reduction in SIH (effective doses of 10 and 20 mg/kg [$F(3.29)=15.24$; $p < 0.0001$], but the 5 mg/kg dose was not effective); compound **37** was effective at a dose of 10 mg/kg [$F(3.34)=3{,}35$; $p < 0.03$]; compound **49** was not effective; and compound **60** reduced the temperature increase caused by stress at the doses of 5 and 20 mg/kg [$F\ (3.35) = 3.654$; $p < 0.05$].

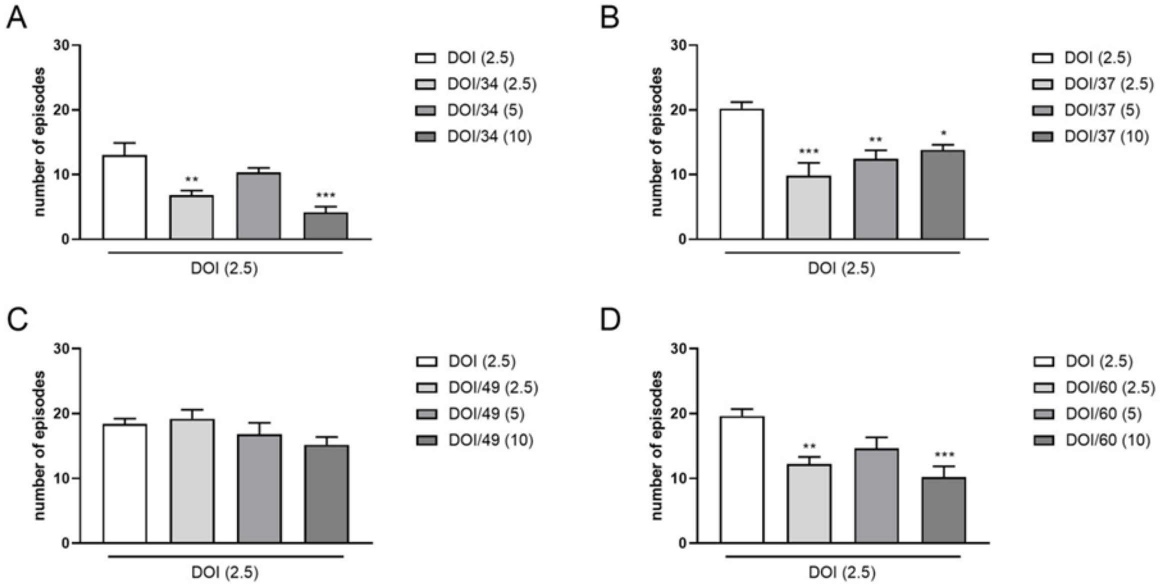

Figure S11. The effects of compounds **34**, **37**, **49**, and **60** on the DOI-induced HTR: Compound **34** revealed a statistically significant effect at the two doses (2.5 and 10 mg/kg) [F(3.20) = 11.21; $p < 0.05$]; compound **37** reversed the DOI-induced effect at all investigated doses [F(3.16) =10.32; $p < 0.005$]; compound **49** was not effective; and compound **60** reduced the number of episodes at doses of 2.5 and 10 mg/kg [F (3.16) = 8.018; $p < 0.05$], while at the 5 mg/kg dose, the effect was not statistically significant.

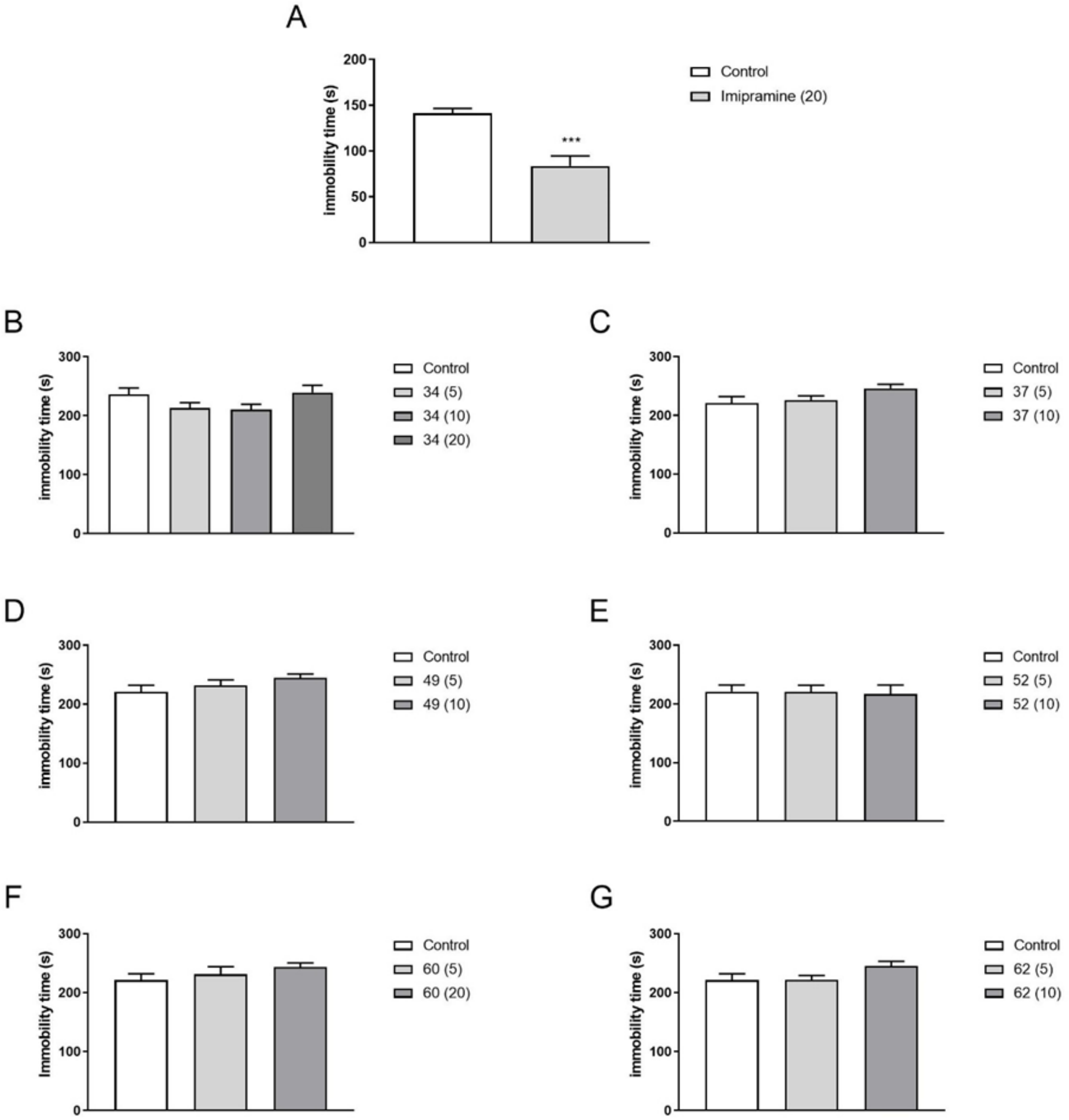

Figure S12. The effects of (A): imipramine [$t_{(14)} = 6.4$; $p < 0.0001$] and 1,2,4-oxadiazole derivatives: (B) **34**, (C), **37**, (D) **49**, (E) **52**, (F) **60** and (G) **62**, on the duration of immobility in the TST in mice. Values are the mean ± S.E.M. with 7 mice in each group (except of imipramine for which n = 8).

References

[1] A. Pilc, A.J. Bojarski, A. Stankiewicz, P. Brański, G. Burnat, R. Bugno, 1,2,4-oxadiazole derivatives as allosteric modulators of metabotropic glutamate receptors belonging to group III, EP2853532A1, 2013.

[2] S.K. Pathak, S. Nath, J. De, S.K. Pal, A.S. Achalkumar, The effect of regioisomerism on the mesomorphic and photophysical behavior of oxadiazole-based tris(N-salicylideneaniline)s: synthesis and characterization, New J. Chem. 41 (2017) 9908–9917. https://doi.org/10.1039/C7NJ01766A.

[3] E. Vieira, A. Binggeli, V. Breu, D. Bur, W. Fischli, R. Güller, G. Hirth, H.P. Märki, M. Müller, C. Oefner, M. Scalone, H. Stadler, M. Wilhelm, W. Wostl, Substituted piperidines - Highly potent renin inhibitors due to induced fit adaptation of tile active site, Bioorganic Med. Chem. Lett. 9 (1999) 1397–1402. https://doi.org/10.1016/S0960-894X(99)00195-X.

[4] B. Chruścicka, G. Burnat, P. Brański, P. Chorobik, T. Lenda, M. Marciniak, A. Pilc, Tetracycline-based system for controlled inducible expression of group III metabotropic glutamate receptors, J. Biomol. Screen. 20 (2015) 350–358. https://doi.org/10.1177/1087057114559183.
